# The D-amino acid oxidase-carbon nanotubes: evaluation of cytotoxicity and biocompatibility of a potential anticancer nanosystem

**DOI:** 10.1007/s13205-023-03568-1

**Published:** 2023-06-19

**Authors:** Elena Rosini, Marta Boreggio, Matteo Verga, Laura Caldinelli, Loredano Pollegioni, Elisa Fasoli

**Affiliations:** 1 Department of Biotechnology and Life Sciences, University of Insubria, Via J.H. Dunant 3, 21100 Varese, Italy; 2 Department of Chemistry, Materials and Chemical Engineering “Giulio Natta”, Politecnico di Milano, Piazza Leonardo da Vinci 32, 20133 Milan, Italy

**Keywords:** Oxidation stress, Drug delivery, Mass spectrometry, Protein corona, Proteomic analysis

## Abstract

The ‘enzyme prodrug therapy’ represents a promising strategy to overcome limitations of current cancer treatments by the systemic administration of prodrugs, converted by a foreign enzyme into an active anticancer compound directly in tumor sites. One example is D-amino acid oxidase (DAAO), a dimeric flavoenzyme able to catalyze the oxidative deamination of D-amino acids with production of hydrogen peroxide, a reactive oxygen species (ROS), able to favor cancer cells death. A DAAO variant containing five aminoacidic substitutions (mDAAO) was demonstrated to possess a better therapeutic efficacy under low O_2_ concentration than wild-type DAAO (wtDAAO). Recently, aiming to design promising nanocarriers for DAAO, multi-walled carbon nanotubes (MWCNTs) were functionalized with polyethylene glycol (PEG) to reduce their tendency to aggregation and to improve their biocompatibility. Here, wtDAAO and mDAAO were adsorbed on PEGylated MWCNTs and their activity and cytotoxicity were tested. While PEG-MWCNTs-DAAOs have shown a higher activity than pristine MWCNTs-DAAO (independently on the DAAO variant used), PEG-MWCNTs-mDAAO showed a higher cytotoxicity than PEG-MWCNTs-wtDAAO at low O_2_ concentration. In order to evaluate the nanocarriers’ biocompatibility, PEG-MWCNTs-DAAOs were incubated in human serum and the composition of protein corona was investigated via nLC-MS/MS, aiming to characterize both soft and hard coronas. The mDAAO variant has influenced the bio-corona composition in both number of proteins and presence of opsonins and dysopsonins: notably, the soft corona of PEG-MWCNTs-mDAAO contained less proteins and was more enriched in proteins able to inhibit the immune response than PEG-MWCNTs-wtDAAO. Considering the obtained results, the PEGylated MWCNTs conjugated with the mDAAO variant seems a promising candidate for a selective antitumor oxidative therapy: under anoxic-like conditions, this novel drug delivery system showed a remarkable cytotoxic effect controlled by the substrate addition, against different tumor cell lines, and a bio-corona composition devoted to prolong its blood circulation time, thus improving the drug’s biodistribution.

**Supplementary Information:**

The online version contains supplementary material available at 10.1007/s13205-023-03568-1.

## Introduction

Cancer represents the second leading cause of mortality worldwide: the well-known limitations of current cancer treatments, chemotherapy and radiotherapy, particularly their low specificity for tumor cells, ask to develop new efficient alternatives (Hassanpour and Dehghani 2017; Abbas and Rehman 2018; Siegel et al. 2018; Schirrmacher 2019; Sung et al. 2021). In this contest the ‘enzyme prodrug therapy’ (EPT) represents a very attractive strategy: it consists in the systemically administration of prodrugs, locally converted into active anticancer compounds by an enzyme targeted into tumor sites (Bashraheel et al. 2020; Sharifi et al. 2020; Japir et al. 2021).

On this side, a promising enzyme is D-amino acid oxidase (EC 1.4.3.3., DAAO), a dimeric flavoenzyme, belonging to the dehydrogenase-oxidase class (Pollegioni et al. 2018; Rosini et al. 2018, 2021; Fuentes-Baile et al. 2021; Rosini and Pollegioni 2022). In particular, the enzyme from *Rhodotorula gracilis* is characterized by high catalytic activity and by stable interaction with the FAD cofactor (Kuan et al. 2008; Pollegioni et al. 2008; Lee et al. 2021). Its ability to catalyze the oxidative deamination of D-amino acids to the corresponding α-keto acids with the production of hydrogen peroxide allows the coupling of EPT benefits with the ‘oxidation therapy’, devoted to selectively generate reactive oxygen species (ROS) in tumor, thus causing cancer cells death via induction of oxidative stress (Jo et al. 2020; Wu et al. 2020; Yu et al. 2020; Rosini and Pollegioni 2022). The formation of hydrogen peroxide by DAAO is heavily influenced by the molecular oxygen concentration in tissues (Rosini et al. 2009, 2011; Rosini and Pollegioni 2022). Actually, an efficient therapeutic index is evident when the oxygen affinity of the enzyme is in the micromolar range, which corresponds to the oxygen concentration found in the microenvironment of solid tumors. Unfortunately, the wild-type DAAO (wtDAAO) shows a K_m_ value for oxygen in the millimolar range, thus rendering the enzyme not suitable for this therapeutic application (Pollegioni et al. 2007; Muz et al. 2015; Tam et al. 2019; Nejad et al. 2021). To overcome this limitation, the mDAAO variant has been developed by a protein engineering approach: this variant, harboring five aminoacidic substitutions (S19G, S120P, Q144R, K321M and A345V), shows a tenfold lower *K*_*m*_ for oxygen (from 1.9 mM to 220 μM) and a better therapeutic efficacy under low O_2_ concentration (Pollegioni et al. 2007; Rosini et al. 2009).

In literature, many studies have reported the combination of DAAO with drug delivery systems, to directly transport the enzyme into tumor sites and to increase its therapeutic efficacy (Rosini and Pollegioni 2022). Particularly, the main tested nanosystems were magnetic nanoparticles, such as iron oxide nanoparticles (Bava et al. 2013; Cappellini et al. 2015; Balzaretti et al. 2017; Fuentes-Baile et al. 2020). Among magnetic nanoparticles used for theragnostic purposes, carbon nanotubes and, in particular, multi-walled carbon nanotubes (MWCNTs) seemed promising nanocarriers. Their ability to cross the plasma membrane and their high surface area made them suitable to carry various bioactive agents, such as chemotherapeutic drugs (Morais et al. 2020; Lyra et al. 2021; Zhang et al. 2021). Many studies were focused on their covalent functionalization with biocompatible polymers, such as polyethylene glycol (PEG), to overcome MWCNTs limitations due to their toxicity and their tendency to aggregate in biological fluids (Tan et al. 2020; Yumita et al. 2020; Solhjoo et al. 2021). Furthermore, it was demonstrated that the coating of DAAO with PEG enhances the enzyme stability, favoring the production of hydrogen peroxide over time (Rosini and Pollegioni 2020; Nakamura et al. 2021).

Another important aspect, to evaluate after administration of a nanocarrier into the human body bloodstream, is the interaction between the nanosystem and biological fluids components, essential to the formation of the ‘protein corona’ (PC), also called ‘bio-corona’, around nanoparticles. The formation of PC is a dynamic process, that involves many weak forces between physiological proteins and nanoparticles’ surface (Fasoli 2021; Kopac 2021; Li et al. 2021). Protein corona composition and thickness are influenced by both nanoparticles features (such as dimension, morphology and surface properties) and environmental features (like biological fluid composition, pH and temperature). The bio-corona is able to affect nanoparticles properties, influencing their biodistribution and their targeting capacity (Nicoletti et al. 2018; Huang et al. 2021; Tengjisi et al. 2022). PC could be divided into two main layers: ‘soft corona’ (SC) and ‘hard corona’ (HC). Proteins belonging to HC directly bind nanoparticles surface with high affinity, generating a stable and nonexchangeable layer. HC is responsible of nanoparticle fate by controlling membrane adhesion, cellular signaling pathways, biodistribution and interactions with surrounding cells. SC proteins indirectly bind nanoparticles, interacting with HC components: it is an exchangeable layer, highly dependent on the type of biological environment (Galdino et al. 2021; García-Álvarez and Vallet-Regí 2021; Nicoletti et al. 2021a, b).

The current research aimed not only to investigate the composition and the role of protein corona formed onto potential anticancer nanosystems, as in our previous study (Boreggio et al. 2022), but also to evaluate and to compare the cytotoxic effect against selected tumor cells lines and the biocompatibility of wtDAAO and mDAAO variant adsorbed on PEGylated MWCNTs. Notably, the functionalization of nanotubes with the mDAAO variant active at low oxygen concentration, a condition resembling the microenvironment found in the central part of tumors, makes the new developed drug delivery system a promising candidate for a cancer oxidative therapy.

## Materials and methods

### Design of MWCNTs

MWCNTs, prepared by the typical chemical vapour deposition (CVD) protocol, were oxidized to obtain carboxylic functions on their surface, as reported by Nicoletti et al. (2021b). MWCNTs were subsequently functionalized with methoxypolyethylene glycol amine 5000 (PEG 5 kDa), following the protocol reported by Boreggio et al. (2022). In particular, 200 mg of MWCNTs, 50 mL of dry tetrahydrofuran, 500 mg of PEG, 200 mg of *N,N′*-dicyclohexylcarbodiimide and 60 mg of 4-(dimethylamino)pyridine were used. The obtained PEGylated MWCNTs (PEG-MWCNTs) were characterized with thermal gravimetric analysis (TGA) (heating from 35 °C to 900 °C, in air), to evaluate the functionalization degree. Scanning Electron Microscopy (SEM) analysis was performed using an Evo 50 Zeiss scanning electron microscope under vacuum after gold sample treatment. Fourier Transform Infrared Spectroscopy (FTIR) was performed using Nicolet iS5 FT-IR Spectrometer (Nicolet Instrument Corporation, USA): the sample was prepared by grinding potassium bromide (KBr) with a very small amount of dried PEG-MWCNTs and the produced powder was compressed to form a thin pellet. All reagents were purchased from Merck Millipore® and were used without any further purification.

### DAAO production and incubation with MWCNTs

Both the recombinant wtDAAO and mDAAO variant were expressed and purified from BL21(DE3)pLysS *Escherichia coli* cells (Fantinato et al. 2001). Pure enzymes had a specific activity on D-alanine (D-Ala) of 115 U/mg protein at 25 °C, 21% O_2_. At lower oxygen and D-Ala concentrations (2.5% and 0.1 mM, respectively), the specific activity was close to zero for wtDAAO and 6.5 U/mg for mDAAO (Rosini et al. 2009).

2 mg/mL wtDAAO solution was incubated with 2 mg of both MWCNTs and PEG-MWCNTs, to obtain MWCNTs-wtDAAO and PEG-MWCNTs-wtDAAO, respectively, following the protocol reported by Boreggio et al. (2022). The same procedure was repeated to incubate PEG-MWCNTs with mDAAO, obtaining PEG-MWCNTs-mDAAO.

### DAAO’s activity and stability assay

The activity of both wtDAAO and mDAAO associated with MWCNTs was determined using the horseradish peroxidase (EC 1.11.1.7; Roche^®^) and *o*-dianisidine (Merck Millipore^®^) coupled assay (Rosini et al. 2018). Particularly, starting from a concentration of 0.6 mg/mL for MWCNTs-wtDAAO, and of 0.4 mg/mL for both PEG-MWCNTs-wtDAAO and PEG-MWCNTs-mDAAO, the assay was performed at the final concentration of 0.2 mg/mL for all samples. As control, the same procedure was applied to free (without DAAO) carboxylated MWCNTs at the same final concentration of 0.2 mg/mL.

The stability of different conjugates was assayed measuring the activity of the enzyme, as described above, every hour for 6 h on samples maintained under gentle stirring at 37 °C.

### In vitro cytotoxicity assay

The cytotoxicity of different MWCNTs-DAAOs was assessed by performing the thiazolyl blue tetrazolium bromide (MTT, Merck Millipore^®^) assay on mouse CT26 (colon carcinoma) and human U87 (glioblastoma) cancer cell lines, as well as on monkey fibroblast COS-7 (kidney) cell as control. Cells were plated in 96-well culture plates at a density of 3000 cells per well, cultured overnight at 37 °C in a 5% CO_2_ incubator in Dulbecco’s Modified Eagle Medium (DMEM) (Euroclone) supplemented with 10% fetal bovine serum, 4.5 g/L glucose, 1 mM L-glutamine, 1 mM sodium pyruvate, and penicillin–streptomycin and then exposed to 10 mU enzyme and different D-Ala concentrations (1–20 mM) for 24 h (Rosini et al. 2009; Rosini and Pollegioni 2020; Boreggio et al. 2022). Toxicity was quantified as the fraction of surviving cells relative to the untreated sample as control (i.e., cells incubated without enzyme or D-Ala) taken as 100% of survival. The cytotoxic assay was also performed at 2.5% (30 µM) O_2_ using the Atmosbag incubation system (Merck Millipore^®^) (Rosini et al. 2009; Rosini and Pollegioni 2020).

### Protein corona investigation by SDS-PAGE analysis

MWCNTs-wtDAAO and PEG-MWCNTs-DAAO (both wtDAAO and mDAAO) were incubated with human serum (HS) (purchased from Merk Millipore^®^), following the protocol reported by Boreggio et al. (2022). Protein corona were then eluted four times using 150 µL phosphate-buffered saline (PBS) solution at 37 °C (E1), 150 µL PBS solution at 99 °C (E2), 150 µL 4% sodium dodecyl sulfate (SDS) solution at 99 °C (E3) and 150 µL 20 mM dithiothreitol (DTT) solution at 99 °C (E4). Every elution step was performed for 10 min under vigorous stirring. The same procedure was applied also to both MWCNTs and PEG-MWCNTs without DAAO, as control, to evaluate the affinity of free MWCNTs for human proteins.

The protein concentration of eluates E1, E2 and E3 was evaluated with the Thermo Scientific Pierce™ Bicinchoninic Protein Assay kit (BCA), using bovine serum albumin as standard. The last eluate, E4, was not quantified because the amount of DTT in the sample was not compatible with the used kit. All eluates were loaded onto SDS-PAGE gels as following. In the first gel, PEG-MWCNTs and PEG-MWCNTs-wtDAAO were compared: 20 μg of proteins from E1, 18 μg of proteins from E2 and 5 μg of proteins from E3 of both samples were loaded. In a second gel PEG-MWCNTs and PEG-MWCNTs-mDAAO were compared: 6 μg of proteins from E1, 6 μg of proteins from E2 and 6 μg of proteins from E3 of both samples were loaded. In all gels, 15 μL of E4 of both samples were loaded.

### Mass spectrometry and data analysis

SDS-PAGE gels were cut and treated following the standardized protocols, using trypsin as digestive enzyme (Boreggio et al. 2022). 8 μL of tryptic-digested samples was injected in a nanochromatography system (UltiMate 3000 RSLCnano System, Thermo Scientific), coupled with a mass spectrometer (LTQ XL, Thermo Scientific). The MS data were analyzed by the Mascot search engine (Version 2.3.01), using the Proteome Discoverer software (v. 1.2.0 Thermo) and consulting specific UniProtKB/Swiss-Prot protein database (Swiss-Prot_Human_Reviewed 42,372 sequences and 24,330,934 residues). A preliminary subtraction of common contaminants was performed using definite Contaminants database (262 sequence, 133,770 residues). The identified proteins were classified by molecular function using Gene Ontology (GO) analysis (https://www.ebi.ac.uk/QuickGO) and a comparison between all identified plasma proteins was conducted by Venn diagram (https://bioinfogp.cnb.csic.es/tools/venny/).

### Statistical analyses

The cytotoxicity assays were replicated four times for each condition and data were analyzed for statistical significance using two-way ANOVA followed by a Turkey’s multiple comparison test using GraphPad Prism software (GraphPad Software Inc.). Significance was assessed at *p* < 0.05. As regards mass spectrometry analysis, all samples were injected twice to obtain technical duplicates (Supplementary Table S2). The data validation was performed considering only proteins recognized in both replicates as reliable identifications (Supplementary Table S3).

## Results

### MWCNTs functionalization

MWCNTs were synthesized by the CVD technique and functionalized with PEG (PEG-MWCNTs), according to the published protocols (Boreggio et al. 2022). TGA, a method of thermal analysis frequently used to quantitatively determine the composition of a sample without giving information on the nature of its constituents, was applied to evaluate the PEGylation percentage of carbon nanotubes. A 12% PEGylation degree of MWCNTs was extrapolated: starting from 2 mg of MWCNTs, TGA showed the same functionalization for both wtDAAO and mDAAO variant (0.24 mg) (Supplementary Fig. S1). A detailed characterization of PEG-MWCNTs was performed by SEM and FTIR analyses. Supplementary Fig. S2 depicts about the morphology and the organization of PEG-MWCNTs: four images showed the typical agglomerations of nanotubes (Fig. S2a) and some details of tubular structures (Fig. S2b), increasing instrument’s resolution. Supplementary Fig. S3 reports FTIR spectrum, showing the peaks related to graphitic structure: in particular the free -OH groups at 3464 cm^−1^ and the graphitic C=C bonds at 1639 cm^−1^. Both SEM and FTIR results were not so exhaustive as well as TGA ones to reveal the functionalization on MWCNTs, probably due to the fact that SEM is more suitable for large particles (above 50 nm in diameter) and to the low percentage of PEGylation, enable to produce evident peak in the 1300–1000 cm^−1^ range in FTIR spectrum associated with C-O bonds in PEG structure (Komane et al. 2018; Falank et al. 2019). Considering the enzyme’s adsorption onto PEGylated nanotubes’ surface, similar figures were apparent: ≈ 0.68 mg and ≈ 0.79 mg for wtDAAO and mDAAO variant, respectively, after incubation of 2 mg of functionalized MWCNTs with 3 mg of enzyme (Table 1). The amino acid substitutions introduced in the mDAAO seems to not affect the adsorption onto MWCNTs surface.

**Table 1:** Quantification of PEG and of enzyme for each type of designed carbon nanotubes: MWCNTs-wtDAAO, PEG-MWCNTs-wtDAAO and PEG-MWCNTs-mDAAO

	MWCNTs-wtDAAO	PEG-MWCNTs-wtDAAO	PEG-MWCNTs-mDAAO
mg of functionalization ^a^		0.24	0.24
mg of DAAO ^a^	1.10	0.68	0.79

^a^on 2 mg of MWCNTs

### Activity and cytotoxicity of PEG-MWCNTs-DAAO conjugates

To verify the effect of conjugation on the enzyme functionality, the enzymatic activity of PEG-MWCNTs-DAAO conjugates (i.e. PEG-MWCNTs-wtDAAO and PEG-MWCNTs-mDAAO) on D-Ala as substrate was evaluated via a colorimetric assay in which a chromogenic substrate (*o*-dianisidine) was used to detect the amount of the produced hydrogen peroxide. The apparent enzymatic activity of both PEG-MWCNTs-DAAO conjugates was lower than the value obtained for the free enzyme: a specific activity of 53, 38, and 80 U/mg was determined for PEG-MWCNTs-wtDAAO, PEG-MWCNTs-mDAAO, and free wtDAAO, respectively. Interestingly, the functionalization with PEG has resulted in an increase of the specific activity of PEG-MWCNTS-wtDAAO conjugate of ≈ 5-fold in comparison to MWCNTs-wtDAAO (≈ 10 U/mg).

**Fig. 1:**
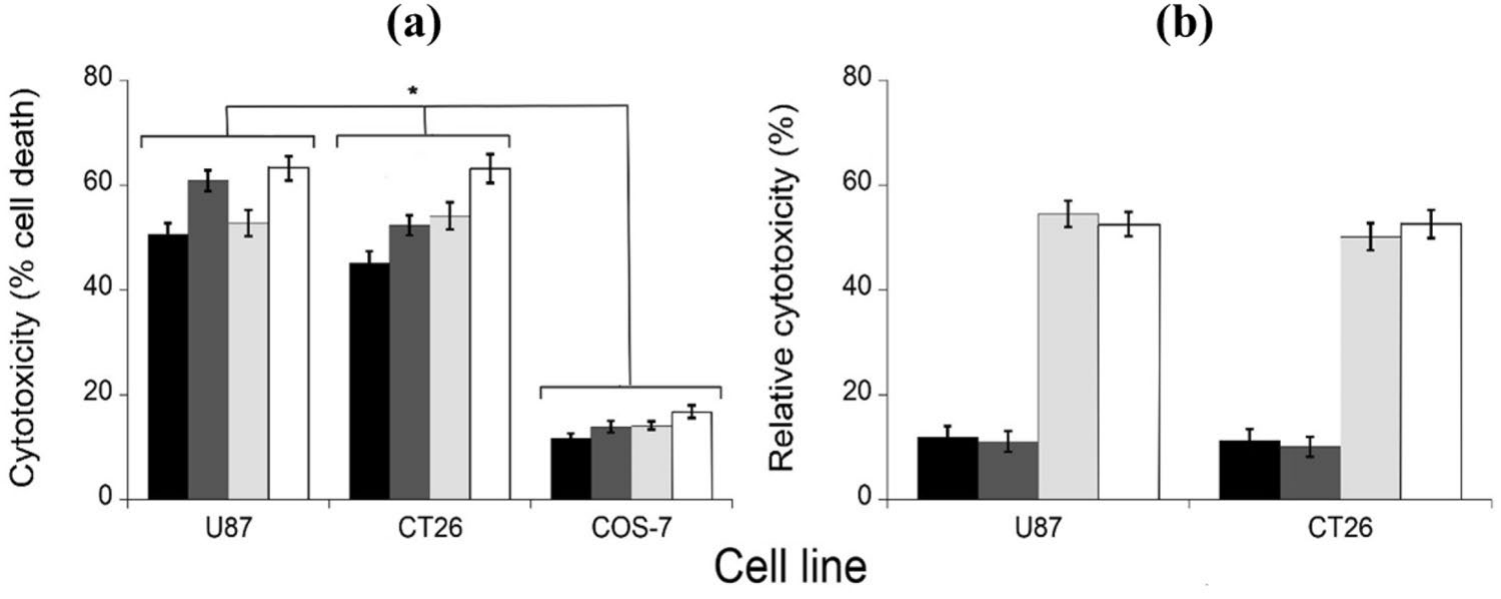
Cytotoxicity of PEG-MWCNTs-wtDAAO (black bars), PEG-MWCNTs-wtDAAO incubated in human plasma (dark grey bars), PEG-MWCNTs-mDAAO (light grey bars), and PEG-MWCNTs-mDAAO incubated in human plasma (white bars), on U87 and CT26 tumor cell lines and COS-7 control cell line. The cytotoxic effect was assayed after 24 h of incubation with 10 mU enzyme, 20 mM D-alanine as substrate at **a** 21% O_2_ and **b** 2.5% O_2_. Toxicity was quantified as the fraction of surviving cells relative to the untreated ones (i.e., the cells incubated without DAAO or D-Ala) taken as 100% of survival. The values are reported as mean ± standard deviation (*n = *4). The results were evaluated by statistical analysis using two‐way ANOVA followed by a Tukey's multiple comparison test. *p < 0.0001

The in vitro cytotoxicity of different PEG-MWCNTs-DAAOs (free and covered with own protein corona), on mouse CT26 and human U87 cancer cell lines, as well as on monkey fibroblast COS-7 cells as control, using D-Ala as the optimal substrate, was evaluated via the MTT test. The effective capability of PEG-MWCNTs-DAAOs nanosystems to selectively kill cancer cells, instead of control ones, following the substrate (D-Ala) addition was investigated. Both free MWCNTs and functionalized MWCNTs-DAAOs alone or D-Ala alone did not induce cytotoxicity against tumor cells (not shown). On the other hand, a remarkable D-Ala dependent cytotoxicity was evident on tumor cells in comparison to control cells (see Fig. 1 and Supplementary Fig. S4). Noteworthy, the presence of protein corona, formed after incubation of functionalized PEG-MWCNTs-DAAOs in human plasma, induced greater cytotoxicity (up to 60% cell death was apparent for the conjugates with corona compared to a 50% value observed for the uncovered free form) (Fig. 1a). We hypothesize that its presence may increase the stability of the enzyme over time, as already observed for the PEG-mDAAO conjugate (Rosini and Pollegioni 2020).

To verify the possible therapeutic application under microaerobic conditions found in tumor tissues, the cytotoxic assay was performed at a low O_2_ concentration (2.5%, 30 µM). The cytotoxicity was most evident for the functionalized PEG-MWCNTs-mDAAO variant as compared to the wild-type conjugate: a 50% of the value measured at air saturation in comparison to a figure of 10% measured for the wild-type form (see Fig. 1b), a result resembling the relative activity measured at low substrate concentrations (Rosini et al. 2009). The antitumor effect was suppressed by the addition of excess bovine catalase, indicating that hydrogen peroxide largely contribute to the observed cytotoxicity (Stegman et al. 1998; Rosini and Pollegioni 2022).

### Proteomic investigation of protein corona

**Fig. 2:**
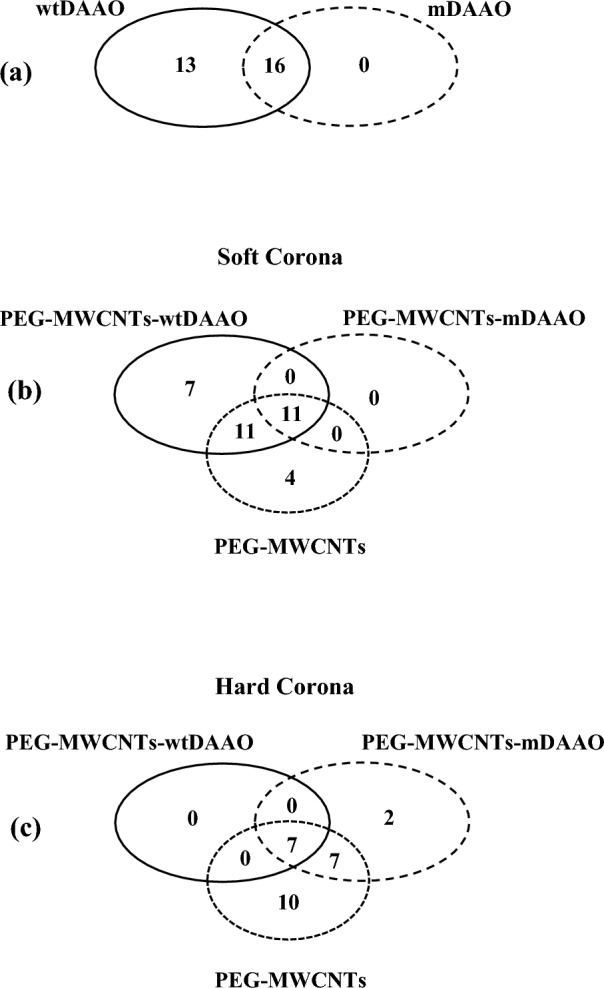
Venn diagrams reported the numbers of proteins in bio-corona around PEGylated carbon nanotubes, identified by mass spectrometry analysis. **a** The protein corona around PEG-MWCNTs-wtDAAO was formed by 13 more proteins than around PEG-MWCNTs-mDAAO. **b** Comparison between soft corona of all three functionalized carbon nanotubes. In absence of enzyme and in presence of wtDAAO, 4 and 7 specific proteins were identified in PEG-MWCNTs and in PEG-MWCNTs-wtDAAO, respectively. **c** Comparison between hard corona of all three functionalized carbon nanotubes. The hard corona in PEG-MWCNTs and in PEG-MWCNTs-mDAAO was enriched with 10 and 2 specific proteins, respectively

To investigate the in vitro formation of protein corona around PEG-MWCNTs-DAAOs, useful to predict nanoparticles’ biodistribution and targeting capacity, proteins belonging to both soft and hard coronas were collected. A protocol of differential elution, based on the application of solutions with different ionic strengths and/or temperature to collect soft and hard corona proteins separately, was applied after the in vitro incubation of PEG-MWCNTs-DAAOs in human serum. Soft corona proteins were eluted by native buffers, like E1 and E2, while hard corona proteins by denaturant buffers, like E3 and E4. To evaluate the protein concentration of the obtained eluates, the Bicinchoninic acid assay was applied only on the eluates compatible with BCA conditions. A much higher amount of proteins in the E1 (PBS at 37 °C) than in E2 (PBS at 99 °C) and traces of proteins in E3 (4% SDS at 99 °C) were revealed (Supplementary Table S1). Such result supported the evidence of abundance of proteins in the soft corona, a dynamic layer involved in the biocompatibility of nanocarriers. Also E4 (eluted at 20 mM DTT at 99 °C) was characterized by many and intense bands, demonstrating the buffer’s efficiency in detaching proteins belonging to hard corona. Supplementary Fig. S5 depicts the role of wtDAAO in the formation of protein corona and Fig. 2a shows the number of validated proteins belonging to the bio-corona around PEGylated carbon nanotubes with both enzymes. The bio-corona around nanostructures with wtDAAO seemed to be more enriched in proteins than the correspondent one around mDAAO. Figure 2b and c, respectively, reported the number of validated proteins in both soft and hard corona, identified in all PEGylated MWCNTs. Again, the presence of enzyme variant seemed to influence the composition of both soft and hard corona, playing a possible role in the biocompatibility of nanocarriers.

**Fig. 3:**
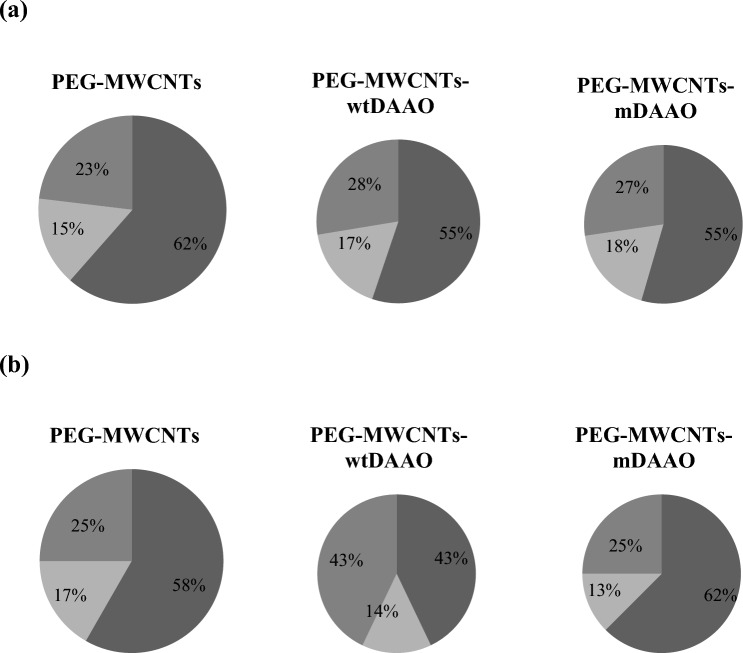
Pie Charts of proteins identified in **a** soft and **b** hard corona around PEGylated carbon nanotubes, PEG-MWCNTs-wtDAAO and PEG-MWCNTs-mDAAO. In all Pie Charts, dark grey sector contained proteins able to activate immune response, the light grey one reported proteins devoted to inhibit the defensive processes and the grey one was characterized by proteins with different functions

The capability of the nanosystem to circulate in biological fluids, reducing the immunogenicity up to reach the target, is strictly related to the number and the type of proteins present in the bio-corona. To investigate the biocompatibility of designed carbon nanotubes, Table 2 compared the number of proteins able to enhance or to inhibit the immune response, present in both soft and hard corona of all functionalized MWCNTs (the full list is reported in Supplementary Table S2). Both PEG-MWCNTs-wtDAAO and PEG-MWCNTs-mDAAO have interacted with immunoglobulins and immune system’s activators like complement C3, but also with inhibitors of immune response, like apolipoprotein B-100, contrasting the previous ones. As reported in Fig. 3, the presence of both DAAO forms has reduced the percentage of immune system’s activators and contemporary increased the amount of inhibitors in soft corona, probably improving the behaviour of nanostructures in physiological conditions.

**Table 2:** Number of proteins able to activate or to inhibit the immune response, identified in soft corona and hard corona of PEG-MWCNTs, PEG-MWCNTs-wtDAAO and PEG-MWCNTs-mDAAO

Total proteins	Pro immune response	Cos immune response
Soft protein corona		
PEG-MWCNTs (26)	16	4
PEG-MWCNTs-wtDAAO (29)	14	5
PEG-MWCNTs-mDAAO (11)	6	2
Hard protein corona		
PEG-MWCNTs (24)	14	4
PEG-MWCNTs-wtDAAO (7)	3	1
PEG-MWCNTs-mDAAO (16)	10	2

The major stealth is observed for PEG-MWCNTs-mDAAO, considering soft corona, and for PEG-MWCNTs-wtDAAO, considering hard corona

## Discussion

To design a promising nanocarrier for enzyme cancer therapy, multi-walled carbon nanotubes were functionalized with PEG chains of 5000 Da, widely used to prepare polymeric drugs. It is well-known that PEGylation is essential in preventing aggregation of nanoparticles, internalization, and phagocytosis (Suk et al. 2016). PEG has the capability to increase the blood circulation time of nanoparticles, preventing their opsonization and reducing the clearance. The molecular weight was selected according to the recent published data about its possibility to reduce of 50% the amount of adsorbed proteins (Gref et al. 2000). It was also demonstrated that surface modification with polysaccharides combined with PEG could favor the interaction with clusterin, enriching the bio-corona in this dysopsonin protein and reducing the clearance by macrophages (Kang et al. 2015; Papini et al. 2020). The molecular weight and the grafting density for PEG could play a crucial role in the stealth properties of nanostructures: nanoparticles’ PEGylation with 5000 Da PEG have more effectively reduced levels of complement-mediated anaphylatoxin C5a than PEGylated nanoparticles with 3400 Da PEG (Maisha et al. 2021). Considering all these features, MWCNTs were functionalized using 5000 Da PEG reaching the 12% PEGylation percentage according to our previous research, as shown in Table 1 and in Supplementary Fig. S1 (Boreggio et al. 2022).

Here, the flavoenzyme DAAO from the yeast *R. gracilis* was selected because of its properties and because the enzyme is active on substrates (i.e., the neutral D-amino acids) present in the micromolar range in mammalian organisms, thus allowing a fine regulation of ROS production following a controlled substrate administration, in the so called “activity on demand” to prevent damage to normal tissue (Rosini et al. 2021; Rosini and Pollegioni 2022). Indeed, to improve the therapeutic index, the mDAAO enzyme variant obtained through a protein engineering approach was used (Rosini et al. 2009), showing an increased activity at low oxygen saturation (30 µM), a condition resembling the microenvironment in the central part of tumors.

In a previous work, the PEGylation of mDAAO variant resulted in a conjugate showing an increased enzymatic activity, thermal stability and half-life in plasma (Rosini and Pollegioni 2020). The PEG-MWCNTs-DAAOs conjugates display a higher activity than the DAAO adsorbed on free MWCNTs (up to 5-fold), confirming the positive effect of PEG conjugation. Noteworthy, at a low O_2_ concentration (30 μM), a significant higher cytotoxicity is evident for the PEG-MWCNTs-mDAAO conjugate as compared to wild-type enzyme form (see Fig. 1b). Under these microaerobic/anoxic conditions, that mimic the central area of the tumor tissue, a 60% cell death was apparent for the treatment with mDAAO variant compared to a 15% value observed for the corresponding wild-type form. This result agrees with the comparatively higher specific activity measured at a low oxygen concentration: a 50% of the activity assayed at air saturation was apparent for mDAAO, whereas the wild-type enzyme was practically inactive (Rosini et al. 2009).

Concerning the PEG-MWCNTs-DAAOs biocompatibility, required to evaluate their potential impact on immune system and on inflammation processes after their intravenous administration, the characterization of protein corona composition was performed by mass spectrometry analysis, focusing the attention on both components of bio-corona: hard and soft corona. As regards the validated number of PC proteins, PEG-MWCNTs-wtDAAO was characterized by a higher figure than PEG-MWCNTs-mDAAO, pointing to an effect of amino acidic substitutions introduced in the mDAAO variant mainly on the protein surface (Rosini et al. 2009) on the interaction with physiological proteins, reducing the layer’s complexity. Considering that PC composition and thickness may affect the properties and the fate of nanoparticles (Lai et al 2017; Senapati et al. 2017), both the type and the biological function of proteins are crucial to evaluate the biocompatibility of designed nanostructures. Both PEG-MWCNTs-wtDAAO and PEG-MWCNTs-mDAAO have interacted with opsonin proteins, like immunoglobulins and complement’s system components, that could act as immunostimulants, enhancing the immune uptake, the inflammation and the clearance. On the contrary, PC of all designed nanocarriers were composed by apolipoprotein B-100 and α-2-macroglobulin, able to improve their biocompatibility, promoting anti-inflammatory activities and inhibiting complement’s system and lectin pathway (Lee et al. 2014; Guan et al. 2019). In general, the enzyme conjugation has reduced interactions with physiological proteins, as shown in Supplementary Fig. S5, probably due to steric hindrance and, interestingly, to substitutions introduced in mDAAO affected the composition of protein corona. The wtDAAO has favoured the binding of 13 specific proteins, mostly belonging to immune system’s activator classes like immunoglobulins (*α* and *k*) and complement factor B. Among proteins belonging to PC of both PEG-MWCNTs-wtDAAO and PEG-MWCNTs-mDAAO, two were particularly interesting: serum albumin (P02768) and serum transferrin (P02787), as reported in Supplementary Table S3. Albumin is a well-known protein able to improve biocompatibility and availability of nanoparticles: it was demonstrated that preformed albumin corona on nanostructures’ surface reduced the plasma proteins adsorption and the complement activation, prolonging the blood circulation (Ruh et al. 2012; Peng et al. 2013). Moreover, serotransferrin is a very promising endogenous protein in the field of nanomedicine because several tumor cell membranes overexpress transferrin receptors as well as the blood–brain barrier. So, transferrin is one of the most investigated ligands for nanoparticles functionalization, to achieve a targeted drug delivery: the possibility to enrich protein corona in transferrin could be crucial to efficiently target nanoparticles into tumor sites or to promote the crossing of the blood–brain barrier for the treatment of brain disorders (Choudhury et al. 2018; Sepand et al. 2020).

To predict the role of bio-corona on the fate of designed nanoparticles, the investigation of HC protein composition seems crucial since it could control the nanostructures’ biodistribution and the interaction with surrounding cells, modulating the membrane adhesion and the cellular signalling pathways (Lai et al. 2017; Pisani et al. 2017). As reported in Table 2 and in Fig. 3, the HC of all functionalized carbon nanotubes were characterized by a prevalence of immune response activators than inhibitors, even if the discrepancy between these classes was most evident in PEG-MWCNTs than in PEG-MWCNTs-wtDAAO and PEG-MWCNTs-mDAAO. The mDAAO variant specifically interacted with 2 immune response activators while PEG-MWCNTs have enriched the pool of 14 HC proteins in 6 new activators of immune system.

Concerning the protein composition of SC, the mDAAO variant has played an important role in its formation, reducing the number of interactions and, probably, also controlling the dimension of nanocarriers. No unique proteins were identified around PEG-MWCNTs-mDAAO surface, probably reducing the SC layer thickness. In general, the formation of a nanometric discontinuous layer around nanoparticles could improve their new biological identity, biodistribution and targeting capacity (Marichal et al. 2019). A higher number of proteins were found in SC around PEG-MWCNTs and PEG-MWCNTs-wtDAAO (Fig. 2b), enriching in biomolecules able to reduce the biocompatibility and to favour inflammation processes, such as immunoglobulins and complement factors, as reported in Table 2 and in Fig. 3. Anyway, the SC of PEG-MWCNTs-wtDAAO was also characterized by C4b-binding protein (P04003) able to control the pathway of complement activation, contrasting the effect of opsonins.

In conclusion, the conjugation of functionalized PEG-MWCNTs with mDAAO allows to induce a cytotoxic effect to specific tumor cells under conditions resembling the anoxic microenvironment found in the central part of solid tumors and to modulate the protein composition of bio-corona that has appeared to be less abundant in proteins and more enriched in dysopsonins proteins, thus allowing a prolonged blood circulation time and a reduced clearance.

Noteworthy, the higher cytotoxic effect observed in vitro on tumor cells in comparison to the control one, could further be enhanced in vivo by (i) the selective accumulation in the tumor tissue following intravenous administration, and (ii) the subsequent administration of the safe substrate D-Ala, thus providing a promising passive targeting approach for a selective antitumor oxidative therapy.

## Supplementary Information

Below is the link to the electronic supplementary material.

Supplementary file1 (DOCX 2245 KB)

Supplementary file2 (DOCX 699 KB)

Supplementary file3 (DOCX 40 KB)
