# Supplementary material for: The D-amino acid oxidase-carbon nanotubes: evaluation of cytotoxicity and biocompatibility of a potential anticancer nanosystem
Source: 3 Biotech. 2023 Jun 19;13(7):243. doi: 10.1007/s13205-023-03568-1 (PMC10279611; doi:10.1007/s13205-023-03568-1)
Supplement: Supplementary file 1 — Supplementary file1 (DOCX 2245 KB) [file 13205_2023_3568_MOESM1_ESM.docx]

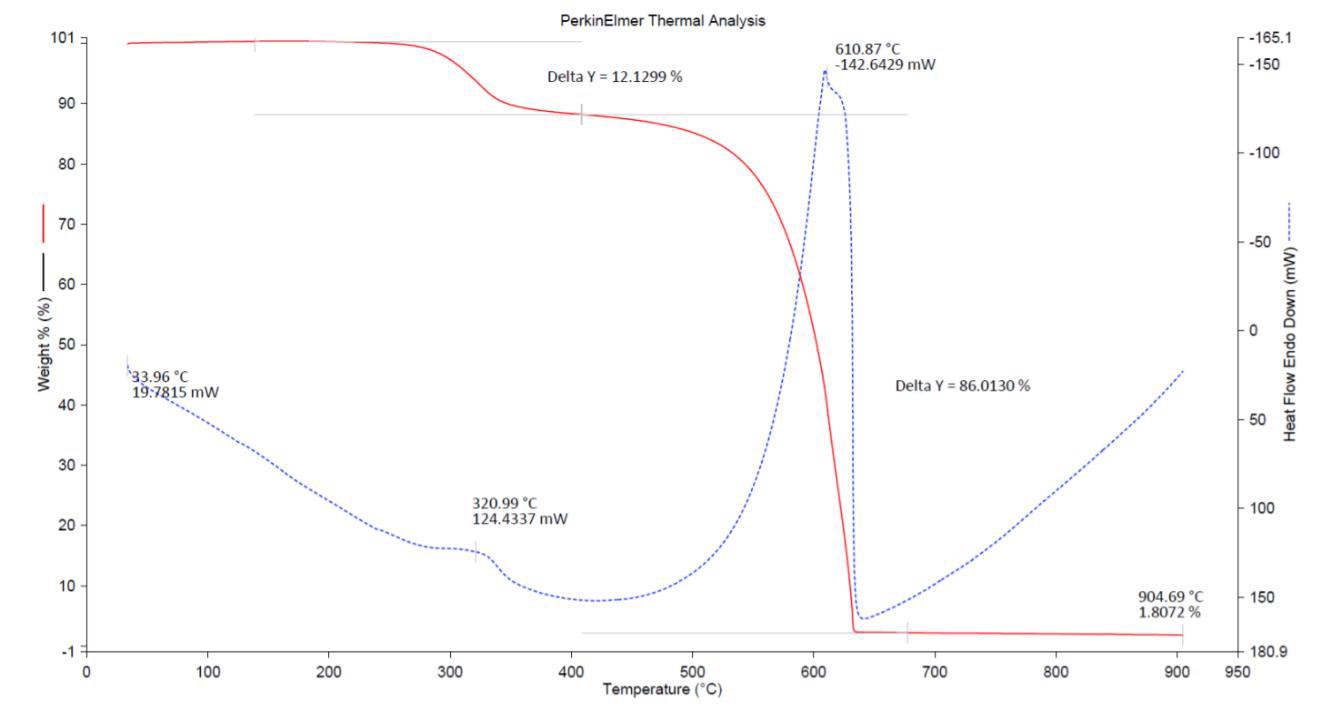


**Figure S1** Spectrum of TGA analysis related to PEG-MWCNTs


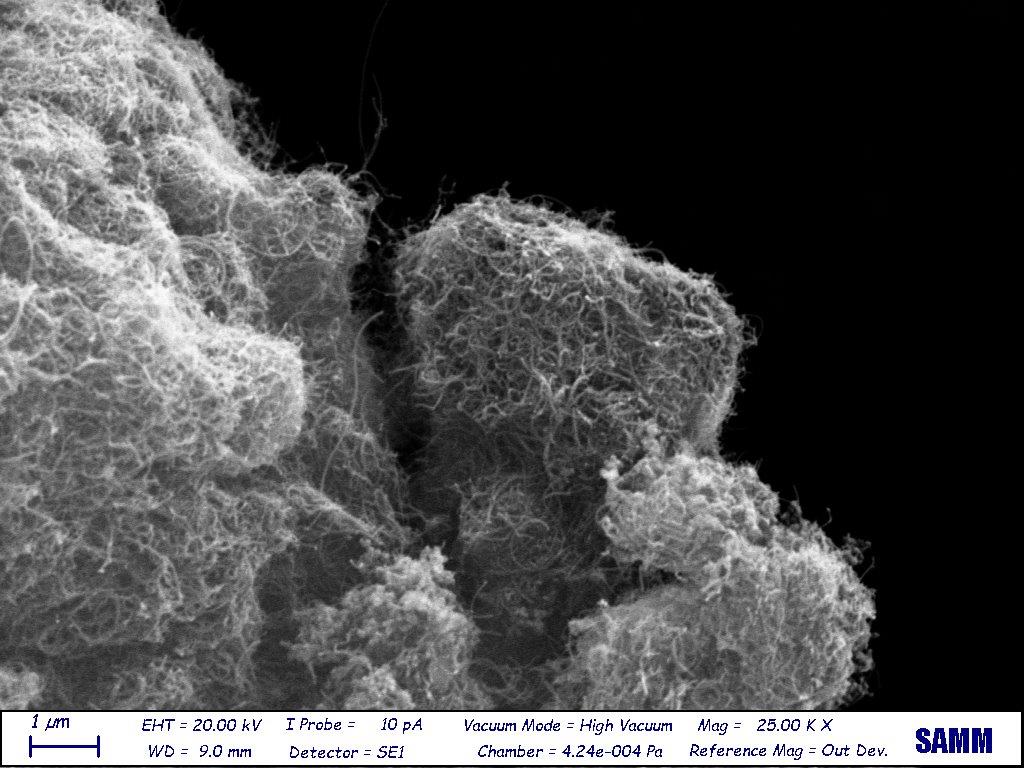

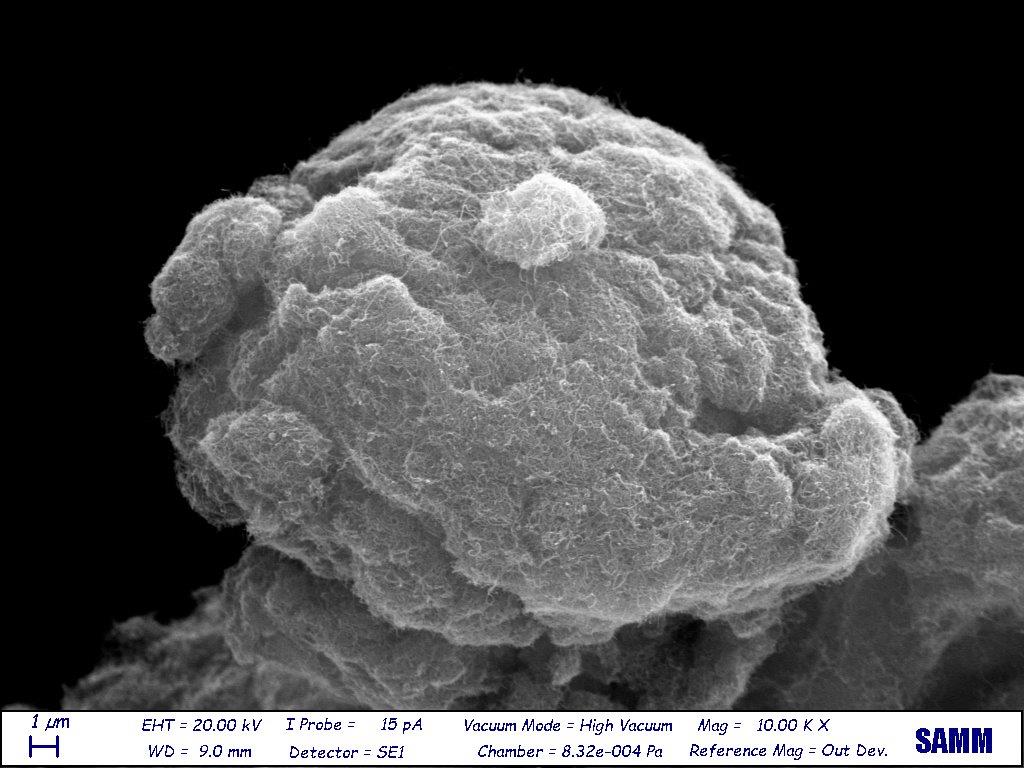

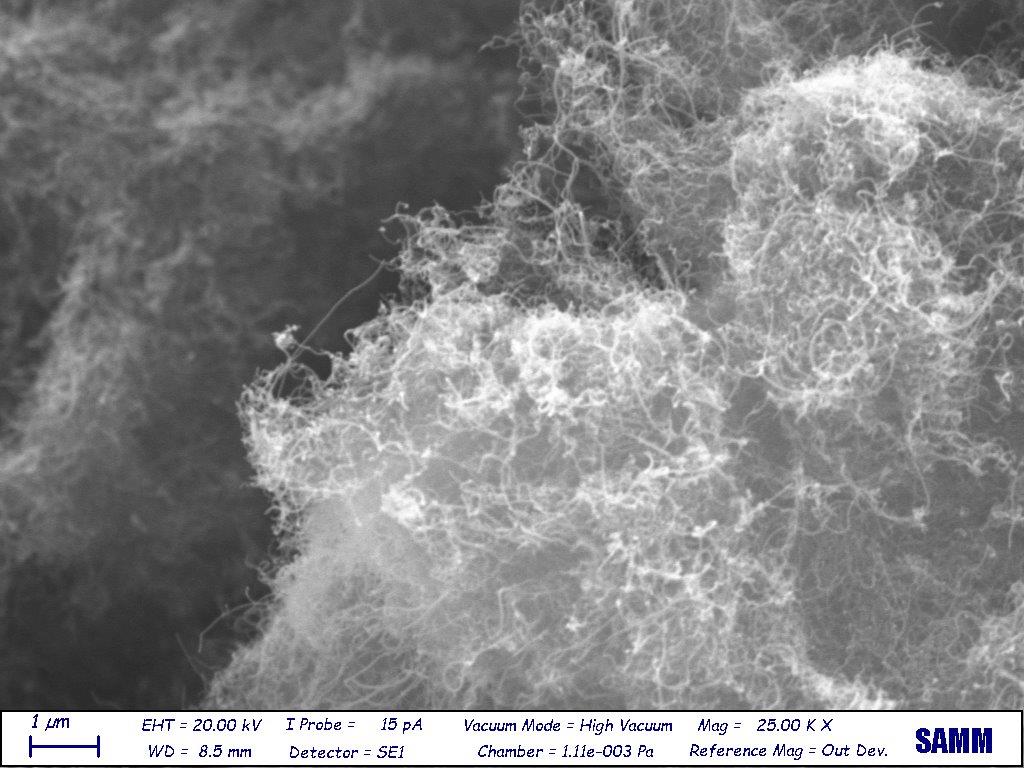

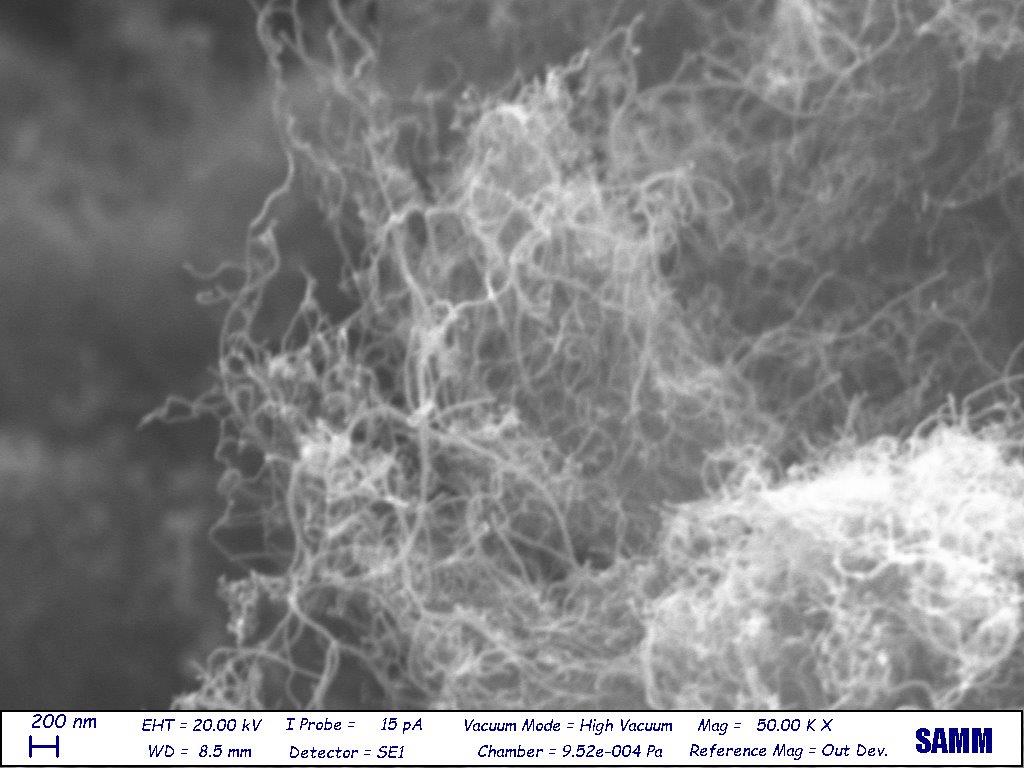


**(b)**

**(a)**

**Figure S2** SEM images of PEG-MWCNTs: agglomerations and detailed tubular structures were respectively showed in both pictures a and b.


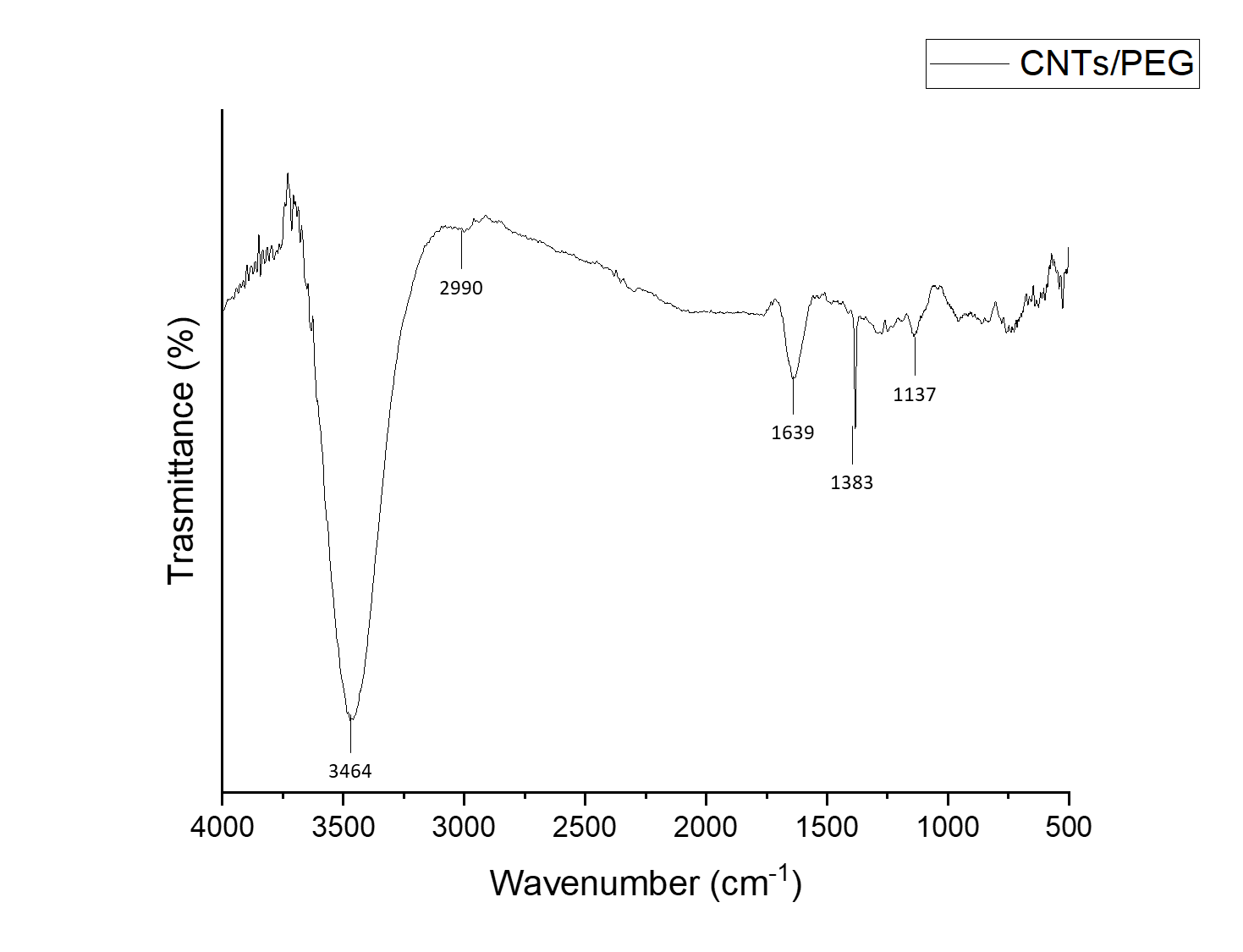


**Figure S3** FTIR spectrum of PEG-MWCNTs reported a peak at 3464 cm^-1^, probably referred to moisture traces and to free -OH groups present on nanotubes surface, and a peak at 1639 cm^-1^, due to stretching vibration of the graphitic C=C bonds on MWCNTs.

**
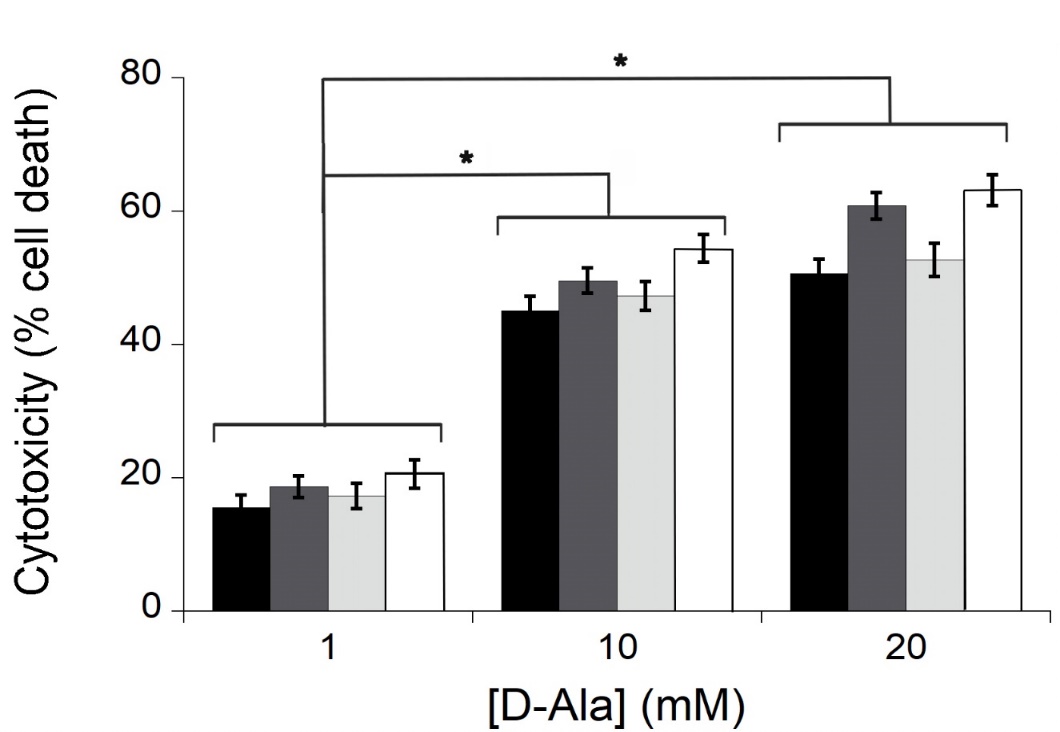
**

**Figure S4** Dependence on D-Ala concentration of cytotoxicity induced by PEG-MWCNTs-wtDAAO (black bars), PEG-MWCNTs-wtDAAO incubated in human plasma (dark grey bars), PEG-MWCNTs-mDAAO (light grey bars), and PEG-MWCNTs-mDAAO incubated in human plasma (white bars) on U87 cells. Toxicity was quantified as the fraction of surviving cells relative to the untreated cells as control (i.e., the cells incubated without DAAO or D-Ala) taken as 100% of survival. The values are reported as mean ± standard deviation (n = 4). The results were evaluated by statistical analysis using two‐way ANOVA followed by a Tukey's multiple comparison test. *p < 0.001

**PEG-MWCNT PEG-MWCNT-wtDAAO PEG-MWCNT PEG-MWCNT-mDAAO**


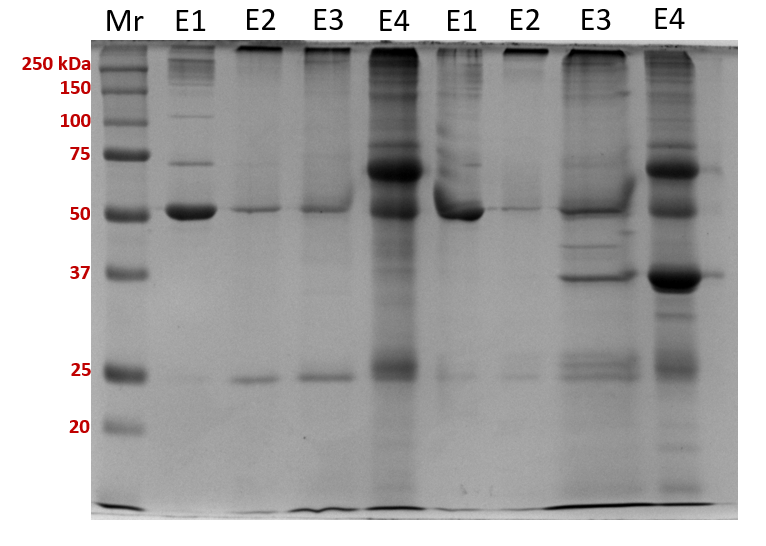


20

25

37

50

75

100

150

250kDa


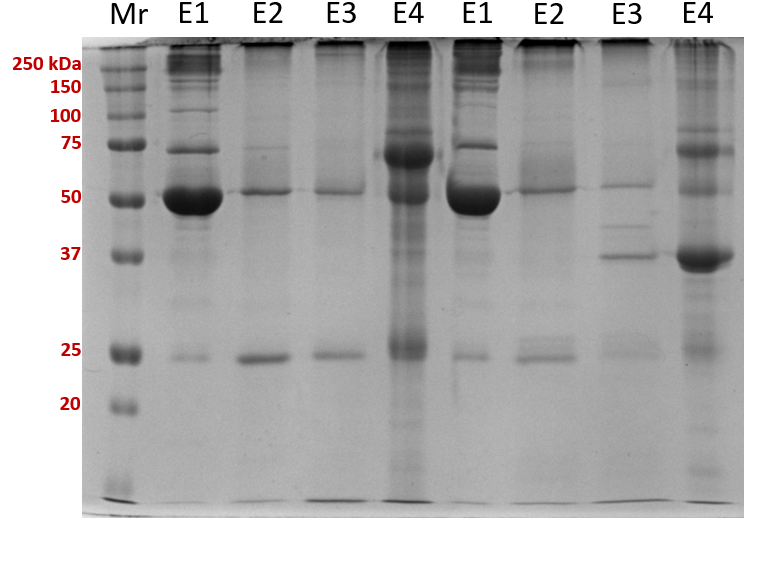


20

25

37

50

75

100

250kDa

150

**(b)**

**(a)**

**PEG-MWCNTs**

**23**

**Effect of DAAO in the Protein Corona**

**PEG-MWCNT-wtDAAO**

**10**

**6**

**(c)**

**Figure S5** SDS-PAGE gels of bio-corona proteins, eluted from PEG-MWCNTs, PEG-MWCNTs-wtDAAO and PEG-MWCNTs-mDAAO. In **a**, eluates of PEG-MWCNTs and of PEG-MWCNTs-wtDAAO were compared, loading 20 μg of proteins from E1, 18 μg of proteins from E2, 5 μg of proteins from E3 and 15 μL of E4. In **b**, eluates of PEG-MWCNTs and of PEG-MWCNTs-mDAAO were compared, loading 6 μg of proteins from E1, 6 μg of proteins from E2, 6 μg of proteins from E3 and 15 μL of E4. In **c**, Venn diagram showed the identified proteins, present in the layer around PEG-MWCNTs and PEG-MWCNTs-wtDAAO, in order to investigate the effect of enzyme in the formation of bio-corona

|  | **Protein Concentration (μg/μl)** | | |
| --- | --- | --- | --- |
|  | **E1** | **E2** | **E3** |
| **PEG-MWCNTs** | 5,0 | 2,3 | 0,7 |
| **PEG-MWCNTs-wtDAAO** | 7,6 | 1,2 | 0,3 |
| **PEG-MWCNTs-mDAAO** | 7,6 | 2,1 | 0,3 |

**Table S1** For all functionalized carbon nanotubes, concentration (μg/μl) of bio-corona proteins was measured by BCA assay in 1X PBS solution at 37°C (E1), in 1X PBS solution at 99°C (E2) and in 4% SDS at 99°C (E3). The presence of 20mM DTT, in E4, was incompatible with BCA assay
