# Supplementary material for: The D-amino acid oxidase-carbon nanotubes: evaluation of cytotoxicity and biocompatibility of a potential anticancer nanosystem
Source: 3 Biotech. 2023 Jun 19;13(7):243. doi: 10.1007/s13205-023-03568-1 (PMC10279611; doi:10.1007/s13205-023-03568-1)
Supplement: Supplementary file 2 — Supplementary file2 (DOCX 699 KB) [file 13205_2023_3568_MOESM2_ESM.docx]

**Table S2.1** PEG-MWCNTs Soft Corona R1

| **Prot. Number** | **Accession Number** | **Protein Name** | **Mascot Score** | **Mr** | **N° pep** | **m/z** | **z** | **Pep. error** | **Pep. Score** | **Pep. sequence** | **Pep. Modification** |
| --- | --- | --- | --- | --- | --- | --- | --- | --- | --- | --- | --- |
| 1 | P02768 | Serum albumin OS=Homo sapiens OX=9606 GN=ALB PE=1 SV=2 | 11758 | 71317 | 42 | 673,361 | 1 | -0,017 | 22,08 | AWAVAR |  |
|  |  |  |  |  |  | 772,1957 | 1 | -0,2428 | 22,98 | AACLLPK |  |
|  |  |  |  |  |  | 395,2374 | 2 | -0,0041 | 39,63 | LVTDLTK |  |
|  |  |  |  |  |  | 880,3468 | 1 | -0,0943 | 44,86 | AEFAEVSK |  |
|  |  |  |  |  |  | 927,4325 | 1 | -0,0609 | 28,6 | YLYEIAR |  |
|  |  |  |  |  |  | 467,2592 | 2 | -0,0074 | 28,07 | LCTVATLR |  |
|  |  |  |  |  |  | 940,3303 | 1 | -0,118 | 66,99 | DDNPNLPR |  |
|  |  |  |  |  |  | 951,3929 | 1 | -0,0488 | 36,86 | DLGEENFK |  |
|  |  |  |  |  |  | 480,6688 | 2 | -0,2322 | 28 | FQNALLVR |  |
|  |  |  |  |  |  | 984,295 | 1 | -0,1934 | 25,85 | TYETTLEK |  |
|  |  |  |  |  |  | 500,7454 | 2 | -0,1201 | 44,9 | QTALVELVK |  |
|  |  |  |  |  |  | 1013,315 | 1 | -0,2838 | 20,75 | LVAASQAALGL |  |
|  |  |  |  |  |  | 1017,5 | 1 | -0,0367 | 59,38 | SLHTLFGDK |  |
|  |  |  |  |  |  | 528,6869 | 2 | 0,7781 | 44,78 | KYLYEIAR |  |
|  |  |  |  |  |  | 536,2054 | 2 | 0,9577 | 22,8 | ETCFAEEGK |  |
|  |  |  |  |  |  | 537,7919 | 2 | 0,034 | 33,51 | LDELRDEGK |  |
|  |  |  |  |  |  | 538,2939 | 2 | 0,0816 | 24,56 | NECFLQHK |  |
|  |  |  |  |  |  | 564,8518 | 2 | -0,0023 | 25,25 | KQTALVELVK |  |
|  |  |  |  |  |  | 569,6988 | 2 | -0,1075 | 48,55 | CCTESLVNR |  |
|  |  |  |  |  |  | 571,3046 | 2 | -0,0919 | 47,22 | KLVAASQAALGL |  |
|  |  |  |  |  |  | 574,8258 | 2 | -0,9707 | 76,1 | LVNEVTEFAK |  |
|  |  |  |  |  |  | 409,2865 | 3 | -0,7601 | 24,35 | FKDLGEENFK |  |
|  |  |  |  |  |  | 653,5858 | 2 | 0,5467 | 34,49 | ECCEKPLLEK |  |
|  |  |  |  |  |  | 656,4768 | 2 | 0,2044 | 21,2 | HPDYSVVLLLR |  |
|  |  |  |  |  |  | 679,5746 | 2 | -0,4878 | 45,51 | AVMDDFAAFVEK | Oxidation (M) |
|  |  |  |  |  |  | 686,2488 | 2 | -0,0765 | 53,79 | AAFTECCQAADK |  |
|  |  |  |  |  |  | 717,8455 | 2 | 0,1502 | 52,42 | ETYGEMADCCAK |  |
|  |  |  |  |  |  | 1443,471 | 1 | -0,1712 | 47,1 | YICENQDSISSK |  |
|  |  |  |  |  |  | 725,6942 | 2 | -0,1473 | 48,93 | ETYGEMADCCAK | Oxidation (M) |
|  |  |  |  |  |  | 489,5328 | 3 | -0,1253 | 33,91 | VTKCCTESLVNR |  |
|  |  |  |  |  |  | 749,5402 | 2 | -0,5053 | 29,69 | TCVADESAENCDK |  |
|  |  |  |  |  |  | 750,561 | 2 | 0,483 | 44,49 | ADDKETCFAEEGK |  |
|  |  |  |  |  |  | 1511,795 | 1 | -0,0474 | 48,53 | VPQVSTPTLVEVSR |  |
|  |  |  |  |  |  | 516,2498 | 3 | -0,062 | 26,48 | LKECCEKPLLEK |  |
|  |  |  |  |  |  | 819,9954 | 2 | -0,799 | 43,87 | DVFLGMFLYEYAR | Oxidation (M) |
|  |  |  |  |  |  | 547,2965 | 3 | -0,0628 | 28,27 | KVPQVSTPTLVEVSR |  |
|  |  |  |  |  |  | 820,4447 | 2 | 0,0996 | 79,79 | DVFLGMFLYEYAR | Oxidation (M) |
|  |  |  |  |  |  | 820,4548 | 2 | -0,0354 | 79,34 | KVPQVSTPTLVEVSR |  |
|  |  |  |  |  |  | 820,4919 | 2 | 0,194 | 76,35 | DVFLGMFLYEYAR | Oxidation (M) |
|  |  |  |  |  |  | 547,3323 | 3 | 0,0447 | 60,32 | KVPQVSTPTLVEVSR |  |
|  |  |  |  |  |  | 820,5372 | 2 | 0,2846 | 47,23 | DVFLGMFLYEYAR | Oxidation (M) |
|  |  |  |  |  |  | 547,3635 | 3 | 0,1383 | 30,82 | KVPQVSTPTLVEVSR |  |
|  |  |  |  |  |  | 820,5734 | 2 | 0,357 | 54,09 | DVFLGMFLYEYAR | Oxidation (M) |
|  |  |  |  |  |  | 547,4 | 3 | 0,2476 | 48,81 | KVPQVSTPTLVEVSR |  |
|  |  |  |  |  |  | 820,692 | 2 | 0,5943 | 45,6 | DVFLGMFLYEYAR | Oxidation (M) |
|  |  |  |  |  |  | 547,4827 | 3 | 0,4957 | 60,96 | KVPQVSTPTLVEVSR |  |
|  |  |  |  |  |  | 820,7484 | 2 | 0,7071 | 45,09 | DVFLGMFLYEYAR | Oxidation (M) |
|  |  |  |  |  |  | 547,5113 | 3 | 0,5816 | 66,35 | KVPQVSTPTLVEVSR |  |
|  |  |  |  |  |  | 829,1652 | 2 | -0,4295 | 40,79 | QNCELFEQLGEYK |  |
|  |  |  |  |  |  | 581,6854 | 3 | 0,1476 | 24,23 | HPYFYAPELLFFAK |  |
|  |  |  |  |  |  | 633,952 | 3 | 0,8464 | 49 | RHPYFYAPELLFFAK |  |
|  |  |  |  |  |  | 637,5635 | 3 | -0,2558 | 21,54 | RPCFSALEVDETYVPK |  |
|  |  |  |  |  |  | 1023,285 | 2 | 0,4679 | 81,09 | VFDEFKPLVEEPQNLIK |  |
|  |  |  |  |  |  | 696,4146 | 3 | 0,3917 | 40,46 | VHTECCHGDLLECADDR |  |
|  |  |  |  |  |  | 997,3568 | 3 | -0,2835 | 35,29 | SHCIAEVENDEMPADLPSLAADFVESK | Oxidation (M) |
| 2 | P02647 | Apolipoprotein A-I OS=Homo sapiens OX=9606 GN=APOA1 PE=1 SV=1 | 2012 | 30759 | 20 | 732,3397 | 1 | -0,0377 | 30,3 | DLEEVK |  |
|  |  |  |  |  |  | 416,3301 | 2 | 0,217 | 24,37 | LAEYHAK |  |
|  |  |  |  |  |  | 437,3358 | 2 | 0,2218 | 45,71 | AELQEGAR |  |
|  |  |  |  |  |  | 448,641 | 2 | -0,2089 | 32,93 | LHELQEK |  |
|  |  |  |  |  |  | 506,847 | 2 | 0,1081 | 33,57 | AKPALEDLR |  |
|  |  |  |  |  |  | 524,3329 | 2 | 0,1446 | 52,72 | LSPLGEEMR | Oxidation (M) |
|  |  |  |  |  |  | 579,7735 | 2 | 0,9125 | 31,82 | LEALKENGGAR |  |
|  |  |  |  |  |  | 608,3962 | 2 | 0,1637 | 42,72 | ATEHLSTLSEK |  |
|  |  |  |  |  |  | 626,5826 | 2 | -0,4628 | 30,97 | VQPYLDDFQK |  |
|  |  |  |  |  |  | 650,3053 | 2 | 0,0359 | 46,84 | WQEEMELYR | Oxidation (M) |
|  |  |  |  |  |  | 651,2352 | 2 | -0,1854 | 42,42 | THLAPYSDELR |  |
|  |  |  |  |  |  | 660,1794 | 2 | 0,7097 | 24,14 | LSPLGEEMRDR | Oxidation (M) |
|  |  |  |  |  |  | 668,1984 | 2 | -0,2566 | 21,07 | QEMSKDLEEVK |  |
|  |  |  |  |  |  | 691,0372 | 2 | 0,3514 | 43,11 | VQPYLDDFQKK |  |
|  |  |  |  |  |  | 693,7983 | 2 | -0,1257 | 89,94 | VSFLSALEEYTK |  |
|  |  |  |  |  |  | 700,5828 | 2 | -0,511 | 115,87 | DYVSQFEGSALGK |  |
|  |  |  |  |  |  | 732,2213 | 2 | 0,5838 | 60,15 | VKDLATVYVDVLK |  |
|  |  |  |  |  |  | 806,7983 | 2 | -0,1959 | 98,98 | LLDNWDSVTSTFSK |  |
|  |  |  |  |  |  | 605,8981 | 3 | -0,1709 | 42,88 | DSGRDYVSQFEGSALGK |  |
|  |  |  |  |  |  | 967,0326 | 2 | 0,1241 | 84,15 | EQLGPVTQEFWDNLEK |  |
| 3 | P02787 | Serotransferrin OS=Homo sapiens OX=9606 GN=TF PE=1 SV=3 | 1743 | 79294 | 27 | 635,4738 | 1 | 0,0975 | 30,07 | DLLFK |  |
|  |  |  |  |  |  | 663,5623 | 1 | 0,1798 | 20,16 | DLLFR |  |
|  |  |  |  |  |  | 735,4327 | 1 | 0,0292 | 24,86 | GDVAFVK |  |
|  |  |  |  |  |  | 827,5113 | 1 | 0,1067 | 33,43 | NPDPWAK |  |
|  |  |  |  |  |  | 437,7333 | 2 | 0,0177 | 52,48 | DSAHGFLK |  |
|  |  |  |  |  |  | 921,4154 | 1 | -0,0192 | 23,57 | DDTVCLAK |  |
|  |  |  |  |  |  | 489,6951 | 2 | -0,1061 | 60,52 | DGAGDVAFVK |  |
|  |  |  |  |  |  | 499,3304 | 2 | 0,1765 | 39,49 | ASYLDCIR |  |
|  |  |  |  |  |  | 500,8413 | 2 | 0,1767 | 42,63 | YLGEEYVK |  |
|  |  |  |  |  |  | 563,6289 | 2 | 0,6785 | 45,18 | KASYLDCIR |  |
|  |  |  |  |  |  | 584,1201 | 2 | 0,6417 | 34,32 | HQTVPQNTGGK |  |
|  |  |  |  |  |  | 606,5273 | 2 | 0,5001 | 44,28 | DSGFQMNQLR | Oxidation (M) |
|  |  |  |  |  |  | 625,4487 | 2 | 0,2843 | 51,68 | SASDLTWDNLK |  |
|  |  |  |  |  |  | 637,5126 | 2 | 0,3644 | 20,08 | HSTIFENLANK |  |
|  |  |  |  |  |  | 642,312 | 2 | 0,0476 | 27,56 | EGYYGYTGAFR |  |
|  |  |  |  |  |  | 659,2008 | 2 | -0,1949 | 62 | WCAVSEHEATK |  |
|  |  |  |  |  |  | 677,7242 | 2 | -0,1895 | 79,37 | DYELLCLDGTR |  |
|  |  |  |  |  |  | 708,2275 | 2 | -0,2721 | 71,66 | SVIPSDGPSVACVK |  |
|  |  |  |  |  |  | 748,226 | 2 | 0,7149 | 56,54 | MYLGYEYVTAIR | Oxidation (M) |
|  |  |  |  |  |  | 766,1133 | 2 | -0,4685 | 27,31 | CSTSSLLEACTFR |  |
|  |  |  |  |  |  | 770,6679 | 2 | 0,6177 | 70,65 | DQYELLCLDNTR |  |
|  |  |  |  |  |  | 789,4953 | 2 | 0,3256 | 97,58 | FDEFFSEGCAPGSK |  |
|  |  |  |  |  |  | 797,531 | 2 | 0,2454 | 50,37 | TAGWNIPMGLLYNK | Oxidation (M) |
|  |  |  |  |  |  | 815,4551 | 2 | 0,087 | 66,17 | EDPQTFYYAVAVVK |  |
|  |  |  |  |  |  | 862,314 | 2 | 0,8599 | 85,79 | LCMGSGLNLCEPNNK | Oxidation (M) |
|  |  |  |  |  |  | 863,0233 | 2 | -0,7276 | 23,02 | IECVSAETTEDCIAK |  |
|  |  |  |  |  |  | 909,7462 | 2 | 0,6807 | 57,86 | EGTCPEAPTDECKPVK |  |
| 4 | P01009 | Alpha-1-antitrypsin OS=Homo sapiens OX=9606 GN=SERPINA1 PE=1 SV=3 | 1327 | 46878 | 9 | 393,4328 | 2 | 0,4067 | 20,3 | VVNPTQK |  |
|  |  |  |  |  |  | 444,9685 | 2 | 0,4261 | 29,05 | AVLTIDEK |  |
|  |  |  |  |  |  | 461,9602 | 2 | 0,4867 | 27,07 | FLENEDR |  |
|  |  |  |  |  |  | 504,9977 | 2 | 0,4885 | 31,03 | QINDYVEK |  |
|  |  |  |  |  |  | 508,2964 | 2 | -0,0291 | 35,25 | SVLGQLGITK |  |
|  |  |  |  |  |  | 555,8446 | 2 | 0,0778 | 55,91 | LSITGTYDLK |  |
|  |  |  |  |  |  | 917,3938 | 2 | -0,1426 | 112,56 | VFSNGADLSGVTEEAPLK |  |
|  |  |  |  |  |  | 946,3059 | 2 | -0,2511 | 90,74 | DTEEEDFHVDQVTTVK |  |
|  |  |  |  |  |  | 1146,23 | 2 | 0,3227 | 72,88 | GTEAAGAMFLEAIPMSIPPEVK | 2 Oxidation (M) |
| 5 | P00738 | Haptoglobin OS=Homo sapiens OX=9606 GN=HP PE=1 SV=1 | 1240 | 45861 | 11 | 429,9578 | 2 | 0,4152 | 32,46 | QLVEIEK |  |
|  |  |  |  |  |  | 460,7876 | 2 | 0,1054 | 53,1 | GSFPWQAK |  |
|  |  |  |  |  |  | 490,5554 | 2 | -0,3913 | 32,58 | VGYVSGWGR |  |
|  |  |  |  |  |  | 530,8822 | 2 | 0,2042 | 40,58 | VMPICLPSK | Oxidation (M) |
|  |  |  |  |  |  | 602,492 | 2 | 0,3399 | 65,14 | VTSIQDWVQK |  |
|  |  |  |  |  |  | 645,8662 | 2 | -0,0052 | 60,77 | DIAPTLTLYVGK |  |
|  |  |  |  |  |  | 656,7899 | 2 | 0,9663 | 46,9 | TEGDGVYTLNDK |  |
|  |  |  |  |  |  | 673,1028 | 2 | -0,4473 | 48,64 | SCAVAEYGVYVK |  |
|  |  |  |  |  |  | 720,6407 | 2 | 0,6092 | 63,37 | TEGDGVYTLNNEK |  |
|  |  |  |  |  |  | 862,697 | 2 | 0,5726 | 91,09 | YVMLPVADQDQCIR | Oxidation (M) |
|  |  |  |  |  |  | 730,3071 | 3 | 0,8543 | 28,81 | SPVGVQPILNEHTFCAGMSK | Oxidation (M) |
| 6 | P01024 | Complement C3 OS=Homo sapiens OX=9606 GN=C3 PE=1 SV=2 | 1118 | 188569 | 34 | 385,3159 | 2 | 0,168 | 20,96 | VVPEGIR |  |
|  |  |  |  |  |  | 388,6837 | 2 | -0,1063 | 30,54 | GVFVLNK |  |
|  |  |  |  |  |  | 805,6079 | 1 | 0,1261 | 31,86 | TFISPIK |  |
|  |  |  |  |  |  | 417,3546 | 2 | 0,2139 | 52,43 | LPYSVVR |  |
|  |  |  |  |  |  | 842,5564 | 1 | 0,0218 | 26,15 | VVLVAVDK |  |
|  |  |  |  |  |  | 443,832 | 2 | 0,1323 | 26,59 | ISLPESLK |  |
|  |  |  |  |  |  | 480,5388 | 2 | 0,5295 | 20,9 | EALKLEEK |  |
|  |  |  |  |  |  | 502,021 | 2 | 0,4881 | 59,36 | TGLQEVEVK |  |
|  |  |  |  |  |  | 531,952 | 2 | 0,4082 | 62,09 | ADIGCTPGSGK |  |
|  |  |  |  |  |  | 532,6218 | 2 | 0,691 | 30,13 | DSCVGSLVVK |  |
|  |  |  |  |  |  | 542,1552 | 2 | -0,2551 | 51,93 | GYTQQLAFR |  |
|  |  |  |  |  |  | 547,1476 | 2 | 0,658 | 45,41 | NTLIIYLDK |  |
|  |  |  |  |  |  | 555,9853 | 2 | 0,3367 | 79,17 | VLLDGVQNPR |  |
|  |  |  |  |  |  | 570,3321 | 2 | 0,1162 | 27,09 | FYYIYNEK |  |
|  |  |  |  |  |  | 575,0485 | 2 | 0,4474 | 42,02 | HQQTVTIPPK |  |
|  |  |  |  |  |  | 576,9205 | 2 | 0,2289 | 27,65 | QPSSAFAAFVK |  |
|  |  |  |  |  |  | 612,9927 | 2 | 0,3813 | 53,6 | YYTYLIMNK | Oxidation (M) |
|  |  |  |  |  |  | 413,6427 | 3 | 0,1919 | 32,3 | KVLLDGVQNPR |  |
|  |  |  |  |  |  | 631,5205 | 2 | 0,4319 | 43,09 | QELSEAEQATR |  |
|  |  |  |  |  |  | 633,9475 | 2 | 0,2697 | 62,96 | NTMILEICTR | Oxidation (M) |
|  |  |  |  |  |  | 645,4789 | 2 | 0,3424 | 71,52 | SGSDEVQVGQQR |  |
|  |  |  |  |  |  | 651,1717 | 2 | 0,7484 | 51,87 | ACEPGVDYVYK |  |
|  |  |  |  |  |  | 668,5467 | 2 | 0,3555 | 45,08 | APSTWLTAYVVK |  |
|  |  |  |  |  |  | 686,1078 | 2 | 0,477 | 58,3 | TIYTPGSTVLYR |  |
|  |  |  |  |  |  | 464,1577 | 3 | 0,7618 | 23,35 | KQELSEAEQATR |  |
|  |  |  |  |  |  | 701,3108 | 2 | -0,2209 | 30,07 | SSLSVPYVIVPLK |  |
|  |  |  |  |  |  | 736,1212 | 2 | 0,4553 | 85,55 | IPIEDGSGEVVLSR |  |
|  |  |  |  |  |  | 754,7863 | 2 | 0,8079 | 36,18 | GQGTLSVVTMYHAK | Oxidation (M) |
|  |  |  |  |  |  | 504,556 | 3 | -0,1681 | 29,68 | LVAYYTLIGASGQR |  |
|  |  |  |  |  |  | 828,6285 | 2 | 0,3825 | 79,46 | TVMVNIENPEGIPVK | Oxidation (M) |
|  |  |  |  |  |  | 834,7148 | 2 | 0,6854 | 66,88 | VYAYYNLEESCTR |  |
|  |  |  |  |  |  | 619,3966 | 3 | 0,3197 | 29,08 | SEETKENEGFTVTAEGK |  |
|  |  |  |  |  |  | 1083,761 | 2 | 0,5128 | 64,67 | AYYENSPQQVFSTEFEVK |  |
|  |  |  |  |  |  | 1099,722 | 2 | 0,3061 | 126,74 | VPVAVQGEDTVQSLTQGDGVAK |  |
| 7 | P01834 | Immunoglobulin kappa constant OS=Homo sapiens OX=9606 GN=IGKC PE=1 SV=2 | 1014 | 11929 | 5 | 751,4923 | 2 | -0,7811 | 36,84 | DSTYSLSSTLTLSK |  |
|  |  |  |  |  |  | 899,7235 | 2 | 0,5445 | 67,72 | SGTASVVCLLNNFYPR |  |
|  |  |  |  |  |  | 626,0795 | 3 | 0,2971 | 35,58 | VYACEVTHQGLSSPVTK |  |
|  |  |  |  |  |  | 973,6169 | 2 | 0,1996 | 79,74 | TVAAPSVFIFPPSDEQLK |  |
|  |  |  |  |  |  | 1068,23 | 2 | -0,5163 | 83,7 | VDNALQSGNSQESVTEQDSK |  |
| 8 | P0DOX5 | Immunoglobulin gamma-1 heavy chain OS=Homo sapiens OX=9606 PE=1 SV=2 | 973 | 49925 | 8 | 838,4902 | 1 | -0,013 | 32,9 | ALPAPIEK |  |
|  |  |  |  |  |  | 851,5325 | 1 | 0,1034 | 42,63 | DTLMISR | Oxidation (M) |
|  |  |  |  |  |  | 581,3257 | 2 | 0,0145 | 44,65 | NQVSLTCLVK |  |
|  |  |  |  |  |  | 593,8447 | 2 | 0,0354 | 35,16 | GPSVFPLAPSSK |  |
|  |  |  |  |  |  | 644,0005 | 2 | 0,3198 | 37,48 | EPQVYTLPPSR |  |
|  |  |  |  |  |  | 661,1959 | 2 | -0,2935 | 76,77 | STSGGTAALGCLVK |  |
|  |  |  |  |  |  | 937,4071 | 2 | -0,1149 | 67,36 | TTPPVLDSDGSFFLYSK |  |
|  |  |  |  |  |  | 713,9276 | 3 | 0,7408 | 34,27 | TPEVTCVVVDVSHEDPEVK |  |
| 9 | P0DOX7 | Immunoglobulin kappa light chain OS=Homo sapiens OX=9606 PE=1 SV=1 | 948 | 23650 | 7 | 375,4402 | 2 | 0,4757 | 25,29 | VTITCR |  |
|  |  |  |  |  |  | 751,4923 | 2 | -0,7811 | 36,84 | DSTYSLSSTLTLSK |  |
|  |  |  |  |  |  | 899,7235 | 2 | 0,5445 | 67,72 | SGTASVVCLLNNFYPR |  |
|  |  |  |  |  |  | 626,0795 | 3 | 0,2971 | 35,58 | VYACEVTHQGLSSPVTK |  |
|  |  |  |  |  |  | 955,2306 | 2 | 0,5571 | 127,14 | DIQMTQSPSTLSASVGDR | Oxidation (M) |
|  |  |  |  |  |  | 1002,443 | 2 | 0,83 | 29,38 | GTVAAPSVFIFPPSDEQLK |  |
|  |  |  |  |  |  | 1068,23 | 2 | -0,5163 | 83,7 | VDNALQSGNSQESVTEQDSK |  |
| 10 | P04114 | Apolipoprotein B-100 OS=Homo sapiens OX=9606 GN=APOB PE=1 SV=2 | 848 | 516651 | 31 | 394,695 | 2 | 0,8751 | 22,49 | FIIPGLK |  |
|  |  |  |  |  |  | 481,5131 | 2 | 0,4876 | 60,68 | LDVTTSIGR |  |
|  |  |  |  |  |  | 506,8318 | 2 | 0,0162 | 58,66 | TGISPLALIK |  |
|  |  |  |  |  |  | 508,2141 | 2 | -0,1249 | 53,58 | VSTAFVYTK |  |
|  |  |  |  |  |  | 514,7147 | 2 | -0,1877 | 55,02 | LSNVLQQVK |  |
|  |  |  |  |  |  | 1039,386 | 1 | -0,2535 | 20,94 | LAPGELTIIL |  |
|  |  |  |  |  |  | 523,4959 | 2 | 0,3805 | 31,12 | IPSVQINFK |  |
|  |  |  |  |  |  | 555,8907 | 2 | 0,2493 | 54,17 | VPQTDMTFR | Oxidation (M) |
|  |  |  |  |  |  | 557,2575 | 2 | -0,0717 | 41,53 | LAAYLMLMR | 2 Oxidation (M) |
|  |  |  |  |  |  | 563,2884 | 2 | -0,0277 | 52,86 | LIDVISMYR | Oxidation (M) |
|  |  |  |  |  |  | 576,6814 | 2 | 0,7864 | 64,95 | LDFSSQADLR |  |
|  |  |  |  |  |  | 598,7434 | 2 | -0,1032 | 37,75 | NMEVSVATTTK | Oxidation (M) |
|  |  |  |  |  |  | 636,4526 | 2 | 0,2139 | 60,35 | SVSLPSLDPASAK |  |
|  |  |  |  |  |  | 640,9408 | 2 | 0,1535 | 51,98 | TEVIPPLIENR |  |
|  |  |  |  |  |  | 644,2882 | 2 | 0,8377 | 78,86 | NTLELSNGVIVK |  |
|  |  |  |  |  |  | 654,1408 | 2 | 0,57 | 29,85 | EFQVPTFTIPK |  |
|  |  |  |  |  |  | 655,6213 | 2 | 0,5355 | 27,03 | EVGTVLSQVYSK |  |
|  |  |  |  |  |  | 677,7595 | 2 | -0,1918 | 54,65 | YGMVAQVTQTLK | Oxidation (M) |
|  |  |  |  |  |  | 679,0369 | 2 | 0,2528 | 53,96 | LPYTIITTPPLK |  |
|  |  |  |  |  |  | 694,3622 | 2 | 0,9333 | 71,8 | IAELSATAQEIIK |  |
|  |  |  |  |  |  | 700,6179 | 2 | 0,4243 | 20,69 | TLADLTLLDSPIK |  |
|  |  |  |  |  |  | 722,682 | 2 | -0,3244 | 27,36 | LQSTTVMNPYMK | 2 Oxidation (M) |
|  |  |  |  |  |  | 753,6007 | 2 | 0,4136 | 75,27 | IGQDGISTSATTNLK |  |
|  |  |  |  |  |  | 791,6558 | 2 | 0,5101 | 88,51 | AVSMPSFSILGSDVR | Oxidation (M) |
|  |  |  |  |  |  | 796,5925 | 2 | 0,3088 | 60,81 | VLLDQLGTTISFER |  |
|  |  |  |  |  |  | 808,2161 | 2 | 0,6998 | 44,09 | MYQMDIQQELQR | 2 Oxidation (M) |
|  |  |  |  |  |  | 809,1951 | 2 | 0,4936 | 51,27 | TSSFALNLPTLPEVK |  |
|  |  |  |  |  |  | 815,7541 | 2 | 0,7545 | 91,01 | MTSNFPVDLSDYPK | Oxidation (M) |
|  |  |  |  |  |  | 829,8442 | 2 | 0,8057 | 23,76 | SVSDGIAALDLNAVANK |  |
|  |  |  |  |  |  | 961,2305 | 2 | 0,5131 | 80,07 | VIGNMGQTMEQLTPELK | 2 Oxidation (M) |
|  |  |  |  |  |  | 982,5064 | 2 | 0,9486 | 49,2 | TILGTMPAFEVSLQALQK | Oxidation (M) |
| 11 | P01860 | Immunoglobulin heavy constant gamma 3 OS=Homo sapiens OX=9606 GN=IGHG3 PE=1 SV=2 | 722 | 42287 | 8 | 838,4902 | 1 | -0,013 | 32,9 | ALPAPIEK |  |
|  |  |  |  |  |  | 851,5325 | 1 | 0,1034 | 42,63 | DTLMISR | Oxidation (M) |
|  |  |  |  |  |  | 581,3257 | 2 | 0,0145 | 44,65 | NQVSLTCLVK |  |
|  |  |  |  |  |  | 593,7744 | 2 | 0,0436 | 35,33 | SCDTPPPCPR |  |
|  |  |  |  |  |  | 644,0005 | 2 | 0,3198 | 37,48 | EPQVYTLPPSR |  |
|  |  |  |  |  |  | 644,2468 | 2 | -0,165 | 44,61 | GPSVFPLAPCSR |  |
|  |  |  |  |  |  | 661,1959 | 2 | -0,2935 | 76,77 | STSGGTAALGCLVK |  |
|  |  |  |  |  |  | 452,8134 | 3 | 0,7882 | 27,4 | TPLGDTTHTCPR |  |
| 12 | P0DOY2 | Immunoglobulin lambda constant 2 OS=Homo sapiens OX=9606 GN=IGLC2 PE=1 SV=1 | 585 | 11458 | 5 | 864,2336 | 1 | -0,1431 | 26,18 | TVAPTECS |  |
|  |  |  |  |  |  | 495,8846 | 2 | 0,2518 | 65,02 | AGVETTTPSK |  |
|  |  |  |  |  |  | 856,801 | 2 | 0,8356 | 77,09 | SYSCQVTHEGSTVEK |  |
|  |  |  |  |  |  | 872,3684 | 2 | -0,1292 | 104,25 | YAASSYLSLTPEQWK |  |
|  |  |  |  |  |  | 993,6935 | 2 | 0,3619 | 85,86 | AAPSVTLFPPSSEELQANK |  |
| 13 | P01859 | Immunoglobulin heavy constant gamma 2 OS=Homo sapiens OX=9606 GN=IGHG2 PE=1 SV=2 | 549 | 36505 | 7 | 412,9395 | 2 | 0,3841 | 20,58 | GLPAPIEK |  |
|  |  |  |  |  |  | 851,5325 | 1 | 0,1034 | 42,63 | DTLMISR | Oxidation (M) |
|  |  |  |  |  |  | 581,3257 | 2 | 0,0145 | 44,65 | NQVSLTCLVK |  |
|  |  |  |  |  |  | 644,0005 | 2 | 0,3198 | 37,48 | EPQVYTLPPSR |  |
|  |  |  |  |  |  | 644,2468 | 2 | -0,165 | 44,61 | GPSVFPLAPCSR |  |
|  |  |  |  |  |  | 712,3768 | 2 | 0,0365 | 76,05 | STSESTAALGCLVK |  |
|  |  |  |  |  |  | 961,6328 | 2 | 0,3695 | 58,16 | TTPPMLDSDGSFFLYSK | Oxidation (M) |
| 14 | P01023 | Alpha-2-macroglobulin OS=Homo sapiens OX=9606 GN=A2M PE=1 SV=3 | 519 | 164613 | 17 | 414,9746 | 2 | 0,4846 | 45,33 | SDIAPVAR |  |
|  |  |  |  |  |  | 503,8532 | 2 | 0,2291 | 20,37 | FQVDNNNR |  |
|  |  |  |  |  |  | 510,1324 | 2 | 0,6644 | 48,79 | ATVLNYLPK |  |
|  |  |  |  |  |  | 523,9929 | 2 | 0,3905 | 62,51 | FEVQVTVPK |  |
|  |  |  |  |  |  | 552,3117 | 2 | 0,0107 | 55,89 | SSGSLLNNAIK |  |
|  |  |  |  |  |  | 574,8359 | 2 | 0,0435 | 45,74 | QGIPFFGQVR |  |
|  |  |  |  |  |  | 575,6915 | 2 | -0,2015 | 40,99 | SASNMAIVDVK | Oxidation (M) |
|  |  |  |  |  |  | 605,8143 | 2 | -0,0213 | 59,69 | LPPNVVEESAR |  |
|  |  |  |  |  |  | 628,3644 | 2 | 0,0785 | 53,15 | AIGYLNTGYQR |  |
|  |  |  |  |  |  | 637,16 | 2 | 0,6399 | 64,27 | VTAAPQSVCALR |  |
|  |  |  |  |  |  | 638,4788 | 2 | 0,3828 | 70,4 | VGFYESDVMGR | Oxidation (M) |
|  |  |  |  |  |  | 697,9068 | 2 | 0,1265 | 94,34 | NEDSLVFVQTDK |  |
|  |  |  |  |  |  | 717,2923 | 2 | 0,7541 | 57,46 | MVSGFIPLKPTVK | Oxidation (M) |
|  |  |  |  |  |  | 783,7125 | 2 | 0,5856 | 78,89 | ALLAYAFALAGNQDK |  |
|  |  |  |  |  |  | 857,8801 | 2 | 0,9166 | 81,85 | SSSNEEVMFLTVQVK | Oxidation (M) |
|  |  |  |  |  |  | 1031,22 | 2 | 0,3398 | 68,57 | LLLQQVSLPELPGEYSMK | Oxidation (M) |
|  |  |  |  |  |  | 796,6082 | 3 | 0,6149 | 47,62 | QQNAQGGFSSTQDTVVALHALSK |  |
| 15 | P01861 | Immunoglobulin heavy constant gamma 4 OS=Homo sapiens OX=9606 GN=IGHG4 PE=1 SV=1 | 512 | 36431 | 6 | 851,5325 | 1 | 0,1034 | 42,63 | DTLMISR | Oxidation (M) |
|  |  |  |  |  |  | 581,3257 | 2 | 0,0145 | 44,65 | NQVSLTCLVK |  |
|  |  |  |  |  |  | 644,2468 | 2 | -0,165 | 44,61 | GPSVFPLAPCSR |  |
|  |  |  |  |  |  | 712,3768 | 2 | 0,0365 | 76,05 | STSESTAALGCLVK |  |
|  |  |  |  |  |  | 947,3667 | 2 | 0,8315 | 31,71 | EPQVYTLPPSQEEMTK | Oxidation (M) |
|  |  |  |  |  |  | 951,7397 | 2 | 0,5442 | 80,52 | TTPPVLDSDGSFFLYSR |  |
| 16 | P01042 | Kininogen-1 OS=Homo sapiens OX=9606 GN=KNG1 PE=1 SV=2 | 467 | 72996 | 10 | 502,6301 | 2 | 0,6845 | 38,36 | QVVAGLNFR |  |
|  |  |  |  |  |  | 565,5715 | 2 | 0,6739 | 23,13 | TWQDCEYK |  |
|  |  |  |  |  |  | 582,9334 | 2 | -0,6923 | 27,06 | AATGECTATVGK |  |
|  |  |  |  |  |  | 626,3429 | 2 | 0,0893 | 46,11 | TVGSDTFYSFK |  |
|  |  |  |  |  |  | 692,6337 | 2 | 0,5988 | 64,44 | ENFLFLTPDCK |  |
|  |  |  |  |  |  | 800,6763 | 2 | 0,5981 | 49,14 | IASFSQNCDIYPGK |  |
|  |  |  |  |  |  | 938,2996 | 2 | 0,7139 | 43,58 | YNSQNQSNNQFVLYR |  |
|  |  |  |  |  |  | 1054,089 | 2 | 0,154 | 88,37 | LGQSLDCNAEVYVVPWEK |  |
|  |  |  |  |  |  | 1070,233 | 2 | 0,377 | 70,08 | DIPTNSPELEETLTHTITK |  |
|  |  |  |  |  |  | 838,2758 | 3 | 0,595 | 53,42 | IASFSQNCDIYPGKDFVQPPTK |  |
| 17 | P01876 | Immunoglobulin heavy constant alpha 1 OS=Homo sapiens OX=9606 GN=IGHA1 PE=1 SV=2 | 455 | 38486 | 8 | 410,0091 | 2 | 0,6066 | 33,66 | VAAEDWK |  |
|  |  |  |  |  |  | 449,0389 | 2 | 0,608 | 28,56 | YLTWASR |  |
|  |  |  |  |  |  | 466,3847 | 2 | 0,2163 | 33,62 | TPLTATLSK |  |
|  |  |  |  |  |  | 470,7053 | 2 | -0,0814 | 40,34 | SAVQGPPER |  |
|  |  |  |  |  |  | 607,3746 | 2 | 0,1096 | 53,68 | WLQGSQELPR |  |
|  |  |  |  |  |  | 688,3962 | 2 | 0,1652 | 58,14 | TFTCTAAYPESK |  |
|  |  |  |  |  |  | 771,1564 | 2 | 0,5776 | 70,69 | DASGVTFTWTPSSGK |  |
|  |  |  |  |  |  | 918,3874 | 2 | -0,1822 | 111,81 | QEPSQGTTTFAVTSILR |  |
| 18 | P0CG04 | Immunoglobulin lambda constant 1 OS=Homo sapiens OX=9606 GN=IGLC1 PE=1 SV=1 | 366 | 11512 | 4 | 864,2336 | 1 | -0,1431 | 26,18 | TVAPTECS |  |
|  |  |  |  |  |  | 856,801 | 2 | 0,8356 | 77,09 | SYSCQVTHEGSTVEK |  |
|  |  |  |  |  |  | 872,3684 | 2 | -0,1292 | 104,25 | YAASSYLSLTPEQWK |  |
|  |  |  |  |  |  | 1022,179 | 2 | 0,3106 | 55,78 | ANPTVTLFPPSSEELQANK |  |
| 19 | P05155 | Plasma protease C1 inhibitor OS=Homo sapiens OX=9606 GN=SERPING1 PE=1 SV=2 | 308 | 55347 | 7 | 455,771 | 2 | 0,0718 | 21,37 | TLYSSSPR |  |
|  |  |  |  |  |  | 558,7878 | 2 | -0,0211 | 56,99 | LLDSLPSDTR |  |
|  |  |  |  |  |  | 593,3234 | 2 | -0,0594 | 26,77 | FQPTLLTLPR |  |
|  |  |  |  |  |  | 610,1204 | 2 | 0,64 | 46,79 | DFTCVHQALK |  |
|  |  |  |  |  |  | 632,9967 | 2 | 0,3078 | 53,32 | TNLESILSYPK |  |
|  |  |  |  |  |  | 758,2023 | 2 | 0,6896 | 65,48 | VTTSQDMLSIMEK | 2 Oxidation (M) |
|  |  |  |  |  |  | 805,6705 | 2 | 0,556 | 38,22 | LEDMEQALSPSVFK | Oxidation (M) |
| 20 | P00450 | Ceruloplasmin OS=Homo sapiens OX=9606 GN=CP PE=1 SV=1 | 200 | 122983 | 5 | 509,5559 | 2 | 0,641 | 39,75 | QYTDSTFR |  |
|  |  |  |  |  |  | 602,5194 | 2 | 0,5039 | 56,81 | EYTDASFTNR |  |
|  |  |  |  |  |  | 686,3762 | 2 | -0,0179 | 79,04 | GAYPLSIEPIGVR |  |
|  |  |  |  |  |  | 735,8518 | 2 | -0,0295 | 71,81 | EVGPTNADPVCLAK |  |
|  |  |  |  |  |  | 760,4222 | 2 | 0,0944 | 59,19 | ALYLQYTDETFR |  |
| 21 | P0C0L4 | Complement C4-A OS=Homo sapiens OX=9606 GN=C4A PE=1 SV=2 | 189 | 194261 | 3 | 558,2383 | 2 | 0,8479 | 74,31 | VGDTLNLNLR |  |
|  |  |  |  |  |  | 771,3214 | 2 | -0,1814 | 103,43 | VLSLAQEQVGGSPEK |  |
|  |  |  |  |  |  | 782,7243 | 2 | 0,5924 | 76,17 | TTNIQGINLLFSSR |  |
| 22 | P10909 | Clusterin OS=Homo sapiens OX=9606 GN=CLU PE=1 SV=1 | 175 | 53031 | 4 | 538,2579 | 2 | 0,966 | 23,72 | IDSLLENDR |  |
|  |  |  |  |  |  | 645,1609 | 2 | 0,6766 | 64,4 | ELDESLQVAER |  |
|  |  |  |  |  |  | 882,1289 | 2 | 0,423 | 96,66 | EILSVDCSTNNPSQAK |  |
|  |  |  |  |  |  | 772,2631 | 3 | 0,5973 | 35,93 | VTTVASHTSDSDVPSGVTEVVVK |  |
| 23 | P05154 | Plasma serine protease inhibitor OS=Homo sapiens OX=9606 GN=SERPINA5 PE=1 SV=3 | 131 | 45760 | 3 | 581,6715 | 2 | 0,761 | 28,62 | AVVEVDESGTR |  |
|  |  |  |  |  |  | 613,4887 | 2 | -0,6828 | 73,75 | AAAATGTIFTFR |  |
|  |  |  |  |  |  | 651,1781 | 2 | 0,707 | 37,7 | FSIEGSYQLEK |  |
| 24 | P08603 | Complement factor H OS=Homo sapiens OX=9606 GN=CFH PE=1 SV=4 | 127 | 143680 | 6 | 488,1506 | 2 | 0,854 | 25,29 | FVCNSGYK |  |
|  |  |  |  |  |  | 595,1306 | 2 | -0,2767 | 63,4 | LSYTCEGGFR |  |
|  |  |  |  |  |  | 630,6615 | 2 | 0,6705 | 40,57 | WQSIPLCVEK |  |
|  |  |  |  |  |  | 632,1446 | 2 | 0,6986 | 40,03 | DGWSAQPTCIK |  |
|  |  |  |  |  |  | 773,8091 | 2 | 0,9284 | 48,94 | WSSPPQCEGLPCK |  |
|  |  |  |  |  |  | 828,1628 | 2 | 0,5765 | 59,55 | AGEQVTYTCATYYK |  |
| 25 | P06727 | Apolipoprotein A-IV OS=Homo sapiens OX=9606 GN=APOA4 PE=1 SV=3 | 119 | 45371 | 4 | 488,861 | 2 | 0,1937 | 20,36 | QLTPYAQR |  |
|  |  |  |  |  |  | 616,6002 | 2 | 0,5468 | 56,73 | ALVQQMEQLR | Oxidation (M) |
|  |  |  |  |  |  | 675,5922 | 2 | -0,4765 | 22,14 | SLAPYAQDTQEK |  |
|  |  |  |  |  |  | 704,4782 | 2 | 0,2377 | 47,96 | LGEVNTYAGDLQK |  |
| 26 | P02790 | Hemopexin OS=Homo sapiens OX=9606 GN=HPX PE=1 SV=2 | 102 | 52385 | 3 | 1141,381 | 1 | -0,2078 | 20,5 | GGYTLVSGYPK |  |
|  |  |  |  |  |  | 580,0963 | 2 | 0,7147 | 26,3 | DYFMPCPGR | Oxidation (M) |
|  |  |  |  |  |  | 748,3497 | 2 | 0,0135 | 43,02 | YYCFQGNQFLR |  |
| 27 | P00747 | Plasminogen OS=Homo sapiens OX=9606 GN=PLG PE=1 SV=2 | 97 | 93247 | 4 | 496,8924 | 2 | 0,3269 | 47,19 | TPENFPCK |  |
|  |  |  |  |  |  | 515,9652 | 2 | 0,3453 | 48,17 | LSSPAVITDK |  |
|  |  |  |  |  |  | 571,0955 | 2 | 0,5578 | 37,27 | EAQLPVIENK |  |
|  |  |  |  |  |  | 667,7328 | 2 | 0,8167 | 53,96 | QLGAGSIEECAAK |  |
| 28 | P01011 | Alpha-1-antichymotrypsin OS=Homo sapiens OX=9606 GN=SERPINA3 PE=1 SV=2 | 95 | 47792 | 2 | 481,1453 | 2 | 0,7724 | 51,2 | ADLSGITGAR |  |
|  |  |  |  |  |  | 608,4095 | 2 | 0,0811 | 77,92 | ITLLSALVETR |  |
| 29 | P01871 | Immunoglobulin heavy constant mu OS=Homo sapiens OX=9606 GN=IGHM PE=1 SV=4 | 92 | 50093 | 3 | 431,9533 | 2 | 0,3749 | 30,92 | VTSTLTIK |  |
|  |  |  |  |  |  | 625,5894 | 2 | 0,5357 | 55,67 | LICQATGFSPR |  |
|  |  |  |  |  |  | 809,71 | 2 | 0,6048 | 63,15 | QVGSGVTTDQVQAEAK |  |
| 30 | P02749 | Beta-2-glycoprotein 1 OS=Homo sapiens OX=9606 GN=APOH PE=1 SV=3 | 77 | 39584 | 3 | 496,9805 | 2 | 0,5183 | 60,99 | TDASDVKPC |  |
|  |  |  |  |  |  | 511,992 | 2 | 0,4502 | 52,89 | ATVVYQGER |  |
|  |  |  |  |  |  | 752,3378 | 2 | 0,8899 | 21,06 | VCPFAGILENGAVR |  |
| 31 | P0DP04 | Immunoglobulin heavy variable 3-43D OS=Homo sapiens OX=9606 GN=IGHV3-43D PE=3 SV=1 | 68 | 13179 | 2 | 653,2578 | 2 | 0,9256 | 47,88 | AEDTALYYCAK |  |
|  |  |  |  |  |  | 678,1537 | 2 | 0,6219 | 51,06 | NSLYLQMNSLR | Oxidation (M) |
| 32 | P02774 | Vitamin D-binding protein OS=Homo sapiens OX=9606 GN=GC PE=1 SV=2 | 63 | 54480 | 2 | 638,5479 | 2 | 0,521 | 54,76 | VCSQYAAYGEK |  |
|  |  |  |  |  |  | 755,8425 | 3 | 0,5761 | 36,8 | SCESNSPFPVHPGTAECCTK |  |
| 33 | P01008 | Antithrombin-III OS=Homo sapiens OX=9606 GN=SERPINC1 PE=1 SV=1 | 61 | 53025 | 2 | 715,7194 | 2 | -0,3574 | 59,99 | VAEGTQVLELPFK |  |
|  |  |  |  |  |  | 721,169 | 2 | -0,2966 | 28,77 | DIPMNPMCIYR | 2 Oxidation (M) |
| 34 | P02765 | Alpha-2-HS-glycoprotein OS=Homo sapiens OX=9606 GN=AHSG PE=1 SV=2 | 52 | 40114 | 3 | 407,3494 | 2 | 0,241 | 35,93 | FSVVYAK |  |
|  |  |  |  |  |  | 424,1511 | 2 | -0,1393 | 25,36 | CNLLAEK |  |
|  |  |  |  |  |  | 599,0807 | 2 | 0,5271 | 39,38 | HTLNQIDEVK |  |
| 35 | P01619 | Immunoglobulin kappa variable 3-20 OS=Homo sapiens OX=9606 GN=IGKV3-20 PE=1 SV=2 | 50 | 12663 | 2 | 365,3885 | 2 | 0,3807 | 21,33 | ATGIPDR |  |
|  |  |  |  |  |  | 490,5554 | 2 | 0,5465 | 49,95 | LLIYGASSR |  |

**Table S2.2** PEG-MWCNTs Soft Corona R2

| **Prot. Number** | **Accession Number** | **Protein Name** | **Mascot Score** | **Mr** | **N° pep** | **m/z** | **z** | **Pep. error** | **Pep. Score** | **Pep. sequence** | **Pep. Modification** |
| --- | --- | --- | --- | --- | --- | --- | --- | --- | --- | --- | --- |
| 1 | P02768 | Serum albumin OS=Homo sapiens OX=9606 GN=ALB PE=1 SV=2 | 8629 | 71317 | 42 | 386,3874 | 2 | -0,6711 | 28,55 | AACLLPK |  |
|  |  |  |  |  |  | 789,2093 | 1 | -0,2624 | 23,78 | LVTDLTK |  |
|  |  |  |  |  |  | 440,5605 | 2 | -0,3273 | 35,12 | AEFAEVSK |  |
|  |  |  |  |  |  | 927,181 | 1 | -0,3124 | 27,9 | YLYEIAR |  |
|  |  |  |  |  |  | 467,1303 | 2 | -0,2652 | 44,39 | LCTVATLR |  |
|  |  |  |  |  |  | 470,576 | 2 | -0,3036 | 24,92 | DDNPNLPR |  |
|  |  |  |  |  |  | 480,5412 | 2 | -0,4874 | 37,66 | FQNALLVR |  |
|  |  |  |  |  |  | 492,6318 | 2 | -0,2321 | 28,2 | TYETTLEK |  |
|  |  |  |  |  |  | 500,5115 | 2 | -0,5879 | 38,03 | QTALVELVK |  |
|  |  |  |  |  |  | 506,8931 | 2 | -0,8201 | 89,04 | LVAASQAALGL |  |
|  |  |  |  |  |  | 509,2295 | 2 | -0,0847 | 34,6 | SLHTLFGDK |  |
|  |  |  |  |  |  | 528,5951 | 2 | 0,5945 | 43,53 | KYLYEIAR |  |
|  |  |  |  |  |  | 537,7461 | 2 | -0,0577 | 40,64 | LDELRDEGK |  |
|  |  |  |  |  |  | 538,1011 | 2 | -0,3041 | 21,44 | NECFLQHK |  |
|  |  |  |  |  |  | 538,1826 | 2 | 0,8153 | 30,4 | LDELRDEGK |  |
|  |  |  |  |  |  | 564,9332 | 2 | 0,1604 | 44,32 | KQTALVELVK |  |
|  |  |  |  |  |  | 569,4356 | 2 | -0,6341 | 27,05 | CCTESLVNR |  |
|  |  |  |  |  |  | 571,0946 | 2 | -0,512 | 54,97 | KLVAASQAALGL |  |
|  |  |  |  |  |  | 574,8837 | 2 | -0,8548 | 22,78 | LVNEVTEFAK |  |
|  |  |  |  |  |  | 383,82 | 3 | -0,1306 | 33,86 | DAHKSEVAHR |  |
|  |  |  |  |  |  | 575,2284 | 2 | -0,1655 | 62,51 | LVNEVTEFAK |  |
|  |  |  |  |  |  | 383,9195 | 3 | 0,168 | 34,67 | DAHKSEVAHR |  |
|  |  |  |  |  |  | 575,4111 | 2 | 0,2 | 37,4 | LVNEVTEFAK |  |
|  |  |  |  |  |  | 384,0132 | 3 | 0,4492 | 31,52 | DAHKSEVAHR |  |
|  |  |  |  |  |  | 575,5282 | 2 | 0,4341 | 54,77 | LVNEVTEFAK |  |
|  |  |  |  |  |  | 384,0708 | 3 | 0,6219 | 36,05 | DAHKSEVAHR |  |
|  |  |  |  |  |  | 575,6445 | 2 | 0,6668 | 48,45 | LVNEVTEFAK |  |
|  |  |  |  |  |  | 384,1084 | 3 | 0,7347 | 30,82 | DAHKSEVAHR |  |
|  |  |  |  |  |  | 575,7491 | 2 | 0,876 | 32,73 | LVNEVTEFAK |  |
|  |  |  |  |  |  | 613,8033 | 2 | -0,0059 | 56,06 | FKDLGEENFK |  |
|  |  |  |  |  |  | 656,7302 | 2 | 0,7112 | 20,17 | HPDYSVVLLLR |  |
|  |  |  |  |  |  | 679,338 | 2 | -0,9609 | 67,45 | AVMDDFAAFVEK | Oxidation (M) |
|  |  |  |  |  |  | 686,1234 | 2 | -0,3272 | 72,18 | AAFTECCQAADK |  |
|  |  |  |  |  |  | 721,8983 | 2 | -0,8526 | 77,58 | YICENQDSISSK |  |
|  |  |  |  |  |  | 726,0365 | 2 | 0,5374 | 51,29 | ETYGEMADCCAK | Oxidation (M) |
|  |  |  |  |  |  | 734,1209 | 2 | -0,6085 | 51,08 | RHPDYSVVLLLR |  |
|  |  |  |  |  |  | 749,3851 | 2 | -0,8155 | 101,68 | TCVADESAENCDK |  |
|  |  |  |  |  |  | 500,39 | 3 | -0,4766 | 46,52 | ADDKETCFAEEGK |  |
|  |  |  |  |  |  | 750,2493 | 2 | 0,9128 | 60,72 | TCVADESAENCDK |  |
|  |  |  |  |  |  | 756,233 | 2 | -0,384 | 66,27 | VPQVSTPTLVEVSR |  |
|  |  |  |  |  |  | 516,4846 | 3 | 0,6426 | 23 | LKECCEKPLLEK |  |
|  |  |  |  |  |  | 776,4042 | 2 | -0,7967 | 23,86 | CCAAADPHECYAK |  |
|  |  |  |  |  |  | 820,1904 | 2 | -0,4089 | 47,19 | DVFLGMFLYEYAR | Oxidation (M) |
|  |  |  |  |  |  | 820,2549 | 2 | -0,4353 | 75,06 | KVPQVSTPTLVEVSR |  |
|  |  |  |  |  |  | 820,3389 | 2 | -0,112 | 34,17 | DVFLGMFLYEYAR | Oxidation (M) |
|  |  |  |  |  |  | 547,2405 | 3 | -0,2309 | 73,35 | KVPQVSTPTLVEVSR |  |
|  |  |  |  |  |  | 820,3988 | 2 | 0,0078 | 50,59 | DVFLGMFLYEYAR | Oxidation (M) |
|  |  |  |  |  |  | 820,4025 | 2 | -0,14 | 74,41 | KVPQVSTPTLVEVSR |  |
|  |  |  |  |  |  | 820,42 | 2 | 0,0503 | 34,7 | DVFLGMFLYEYAR | Oxidation (M) |
|  |  |  |  |  |  | 547,2836 | 3 | -0,1014 | 33,36 | KVPQVSTPTLVEVSR |  |
|  |  |  |  |  |  | 820,4731 | 2 | 0,1565 | 47,99 | DVFLGMFLYEYAR | Oxidation (M) |
|  |  |  |  |  |  | 820,4758 | 2 | 0,0065 | 26,12 | KVPQVSTPTLVEVSR |  |
|  |  |  |  |  |  | 820,5207 | 2 | 0,2516 | 46,98 | DVFLGMFLYEYAR | Oxidation (M) |
|  |  |  |  |  |  | 547,3502 | 3 | 0,0982 | 32,46 | KVPQVSTPTLVEVSR |  |
|  |  |  |  |  |  | 820,7881 | 2 | 0,7865 | 61,94 | DVFLGMFLYEYAR | Oxidation (M) |
|  |  |  |  |  |  | 547,5332 | 3 | 0,6473 | 56,43 | KVPQVSTPTLVEVSR |  |
|  |  |  |  |  |  | 820,8691 | 2 | 0,9484 | 56,41 | DVFLGMFLYEYAR | Oxidation (M) |
|  |  |  |  |  |  | 547,582 | 3 | 0,7938 | 70,24 | KVPQVSTPTLVEVSR |  |
|  |  |  |  |  |  | 828,9081 | 2 | -0,9436 | 37,27 | QNCELFEQLGEYK |  |
|  |  |  |  |  |  | 581,777 | 3 | 0,4225 | 43,97 | HPYFYAPELLFFAK |  |
|  |  |  |  |  |  | 633,5692 | 3 | -0,3023 | 39,03 | RHPYFYAPELLFFAK |  |
|  |  |  |  |  |  | 637,5837 | 3 | -0,195 | 27,56 | RPCFSALEVDETYVPK |  |
|  |  |  |  |  |  | 682,0525 | 3 | -0,9524 | 22,38 | VFDEFKPLVEEPQNLIK |  |
|  |  |  |  |  |  | 696,5445 | 3 | 0,7814 | 29,03 | VHTECCHGDLLECADDR |  |
|  |  |  |  |  |  | 754,0461 | 3 | 0,101 | 23,68 | EFNAETFTFHADICTLSEK |  |
|  |  |  |  |  |  | 830,8218 | 3 | 0,166 | 62,2 | ALVLIAFAQYLQQCPFEDHVK |  |
|  |  |  |  |  |  | 997,1973 | 3 | -0,7621 | 93,32 | SHCIAEVENDEMPADLPSLAADFVESK | Oxidation (M) |
| 2 | P02787 | Serotransferrin OS=Homo sapiens OX=9606 GN=TF PE=1 SV=3 | 1775 | 79294 | 25 | 735,2585 | 1 | -0,145 | 26,47 | GDVAFVK |  |
|  |  |  |  |  |  | 437,6102 | 2 | -0,2286 | 40,08 | DSAHGFLK |  |
|  |  |  |  |  |  | 460,9277 | 2 | -0,5866 | 36,56 | DDTVCLAK |  |
|  |  |  |  |  |  | 482,8449 | 2 | 0,1501 | 54,98 | APNHAVVTR |  |
|  |  |  |  |  |  | 978,2721 | 1 | -0,217 | 34,89 | DGAGDVAFVK |  |
|  |  |  |  |  |  | 499,4927 | 2 | 0,501 | 47,22 | ASYLDCIR |  |
|  |  |  |  |  |  | 500,7014 | 2 | -0,103 | 22,03 | YLGEEYVK |  |
|  |  |  |  |  |  | 563,2208 | 2 | -0,1377 | 35,16 | KASYLDCIR |  |
|  |  |  |  |  |  | 583,9351 | 2 | 0,2716 | 42,29 | HQTVPQNTGGK |  |
|  |  |  |  |  |  | 605,8367 | 2 | -0,8813 | 69,18 | DSGFQMNQLR | Oxidation (M) |
|  |  |  |  |  |  | 637,2433 | 2 | -0,1742 | 60,7 | HSTIFENLANK |  |
|  |  |  |  |  |  | 642,2795 | 2 | -0,0173 | 47,68 | EGYYGYTGAFR |  |
|  |  |  |  |  |  | 677,559 | 2 | -0,52 | 76,77 | DYELLCLDGTR |  |
|  |  |  |  |  |  | 682,4801 | 2 | -0,7713 | 39,16 | CLVEKGDVAFVK |  |
|  |  |  |  |  |  | 708,4113 | 2 | 0,0953 | 67,85 | SVIPSDGPSVACVK |  |
|  |  |  |  |  |  | 748,0689 | 2 | 0,4007 | 67,67 | MYLGYEYVTAIR | Oxidation (M) |
|  |  |  |  |  |  | 766,3018 | 2 | -0,0917 | 32,06 | CSTSSLLEACTFR |  |
|  |  |  |  |  |  | 770,4943 | 2 | 0,2706 | 79,62 | DQYELLCLDNTR |  |
|  |  |  |  |  |  | 529,3942 | 3 | -0,6064 | 24,62 | KPVEEYANCHLAR |  |
|  |  |  |  |  |  | 815,074 | 2 | -0,6752 | 62,2 | EDPQTFYYAVAVVK |  |
|  |  |  |  |  |  | 861,8964 | 2 | 0,0248 | 82,91 | LCMGSGLNLCEPNNK | Oxidation (M) |
|  |  |  |  |  |  | 863,1719 | 2 | -0,4305 | 51,6 | IECVSAETTEDCIAK |  |
|  |  |  |  |  |  | 909,4697 | 2 | 0,1278 | 30,16 | EGTCPEAPTDECKPVK |  |
|  |  |  |  |  |  | 628,0448 | 3 | 0,2439 | 41,67 | ADRDQYELLCLDNTR |  |
|  |  |  |  |  |  | 1095,617 | 2 | -0,778 | 70,87 | IMNGEADAMSLDGGFVYIAGK | 2 Oxidation (M) |
| 3 | P01024 | Complement C3 OS=Homo sapiens OX=9606 GN=C3 PE=1 SV=2 | 1693 | 188569 | 30 | 417,3557 | 2 | 0,2161 | 30,02 | LPYSVVR |  |
|  |  |  |  |  |  | 842,3889 | 1 | -0,1457 | 33,01 | VVLVAVDK |  |
|  |  |  |  |  |  | 430,0206 | 2 | 0,5507 | 33,85 | IFTVNHK |  |
|  |  |  |  |  |  | 444,408 | 2 | 0,3507 | 23,77 | NEQVEIR |  |
|  |  |  |  |  |  | 450,1032 | 2 | 0,7209 | 28,17 | AVLYNYR |  |
|  |  |  |  |  |  | 480,5412 | 2 | 0,5231 | 35,16 | GLEVTITAR |  |
|  |  |  |  |  |  | 502,0083 | 2 | 0,4627 | 52,45 | TGLQEVEVK |  |
|  |  |  |  |  |  | 531,6919 | 2 | -0,1119 | 66,37 | ADIGCTPGSGK |  |
|  |  |  |  |  |  | 546,7294 | 2 | -0,1784 | 48,25 | NTLIIYLDK |  |
|  |  |  |  |  |  | 555,7371 | 2 | -0,1597 | 72,23 | VLLDGVQNPR |  |
|  |  |  |  |  |  | 574,9693 | 2 | 0,2891 | 40,66 | HQQTVTIPPK |  |
|  |  |  |  |  |  | 405,0691 | 3 | 0,5092 | 20,89 | VTIKPAPETEK |  |
|  |  |  |  |  |  | 613,2627 | 2 | 0,9212 | 53,33 | YYTYLIMNK | Oxidation (M) |
|  |  |  |  |  |  | 621,7925 | 2 | -0,0734 | 33,63 | QPVPGQQMTLK | Oxidation (M) |
|  |  |  |  |  |  | 634,0112 | 2 | 0,3971 | 71,16 | NTMILEICTR | Oxidation (M) |
|  |  |  |  |  |  | 645,3862 | 2 | 0,1571 | 86,35 | SGSDEVQVGQQR |  |
|  |  |  |  |  |  | 650,5728 | 2 | -0,4496 | 29,44 | ACEPGVDYVYK |  |
|  |  |  |  |  |  | 685,8008 | 2 | -0,1372 | 49,25 | TIYTPGSTVLYR |  |
|  |  |  |  |  |  | 700,9541 | 2 | -0,9342 | 20,74 | SSLSVPYVIVPLK |  |
|  |  |  |  |  |  | 735,5839 | 2 | -0,6194 | 32,74 | IPIEDGSGEVVLSR |  |
|  |  |  |  |  |  | 754,5386 | 2 | 0,3125 | 35,75 | GQGTLSVVTMYHAK | Oxidation (M) |
|  |  |  |  |  |  | 504,5555 | 3 | -0,1696 | 27,8 | LVAYYTLIGASGQR |  |
|  |  |  |  |  |  | 827,9734 | 2 | -0,9278 | 66,07 | TVMVNIENPEGIPVK | Oxidation (M) |
|  |  |  |  |  |  | 909,0551 | 2 | 0,2106 | 70,29 | SNLDEDIIAEENIVSR |  |
|  |  |  |  |  |  | 614,7235 | 3 | 0,1651 | 31,71 | VHQYFNVELIQPGAVK |  |
|  |  |  |  |  |  | 939,6879 | 2 | -0,605 | 74,94 | EYVLPSFEVIVEPTEK |  |
|  |  |  |  |  |  | 668,2447 | 3 | 0,7539 | 25,71 | SGQSEDRQPVPGQQMTLK | Oxidation (M) |
|  |  |  |  |  |  | 1083,367 | 2 | -0,2757 | 80,4 | AYYENSPQQVFSTEFEVK |  |
|  |  |  |  |  |  | 1099,698 | 2 | 0,2578 | 109,39 | VPVAVQGEDTVQSLTQGDGVAK |  |
|  |  |  |  |  |  | 1247,511 | 2 | -0,1339 | 93,82 | DYAGVFSDAGLTFTSSSGQQTAQR |  |
| 4 | P01834 | Immunoglobulin kappa constant OS=Homo sapiens OX=9606 GN=IGKC PE=1 SV=2 | 1345 | 11929 | 5 | 752,2089 | 2 | 0,652 | 78,26 | DSTYSLSSTLTLSK |  |
|  |  |  |  |  |  | 899,273 | 2 | -0,3566 | 63,99 | SGTASVVCLLNNFYPR |  |
|  |  |  |  |  |  | 938,5518 | 2 | 0,1693 | 33,97 | VYACEVTHQGLSSPVTK |  |
|  |  |  |  |  |  | 973,0864 | 2 | -0,8614 | 53,95 | TVAAPSVFIFPPSDEQLK |  |
|  |  |  |  |  |  | 1068,039 | 2 | -0,8976 | 82,43 | VDNALQSGNSQESVTEQDSK |  |
| 5 | P01009 | Alpha-1-antitrypsin OS=Homo sapiens OX=9606 GN=SERPINA1 PE=1 SV=3 | 1338 | 46878 | 9 | 426,9257 | 2 | 0,3505 | 21,64 | SASLHLPK |  |
|  |  |  |  |  |  | 444,8328 | 2 | 0,1546 | 39,37 | AVLTIDEK |  |
|  |  |  |  |  |  | 504,8314 | 2 | 0,1559 | 21,08 | QINDYVEK |  |
|  |  |  |  |  |  | 508,1875 | 2 | -0,247 | 22,4 | SVLGQLGITK |  |
|  |  |  |  |  |  | 555,3999 | 2 | -0,8116 | 61,89 | LSITGTYDLK |  |
|  |  |  |  |  |  | 821,0342 | 2 | -0,8023 | 21,13 | ITPNLAEFAFSLYR |  |
|  |  |  |  |  |  | 917,3291 | 2 | -0,272 | 115,5 | VFSNGADLSGVTEEAPLK |  |
|  |  |  |  |  |  | 764,063 | 3 | -0,9553 | 34 | GTEAAGAMFLEAIPMSIPPEVK | 2 Oxidation (M) |
|  |  |  |  |  |  | 859,0503 | 3 | 0,7953 | 41,56 | TLNQPDSQLQLTTGNGLFLSEGLK |  |
| 6 | P01023 | Alpha-2-macroglobulin OS=Homo sapiens OX=9606 GN=A2M PE=1 SV=3 | 1061 | 164613 | 22 | 414,9062 | 2 | 0,3477 | 28,8 | SDIAPVAR |  |
|  |  |  |  |  |  | 443,6786 | 2 | -0,0918 | 49,97 | YGAATFTR |  |
|  |  |  |  |  |  | 509,7534 | 2 | -0,0935 | 45,78 | ATVLNYLPK |  |
|  |  |  |  |  |  | 523,5211 | 2 | -0,5532 | 29,08 | FEVQVTVPK |  |
|  |  |  |  |  |  | 542,8824 | 2 | 0,1425 | 33,84 | GHFSISIPVK |  |
|  |  |  |  |  |  | 552,165 | 2 | -0,2827 | 40,1 | SSGSLLNNAIK |  |
|  |  |  |  |  |  | 574,9128 | 2 | 0,1973 | 63,87 | QGIPFFGQVR |  |
|  |  |  |  |  |  | 605,5955 | 2 | -0,4588 | 48,28 | LPPNVVEESAR |  |
|  |  |  |  |  |  | 613,3376 | 2 | 0,1162 | 62,74 | YDVENCLANK |  |
|  |  |  |  |  |  | 628,3539 | 2 | 0,0575 | 35,23 | AIGYLNTGYQR |  |
|  |  |  |  |  |  | 636,7383 | 2 | -0,2036 | 73,68 | VTAAPQSVCALR |  |
|  |  |  |  |  |  | 478,5588 | 3 | 0,8387 | 23,9 | MVSGFIPLKPTVK | Oxidation (M) |
|  |  |  |  |  |  | 756,2675 | 2 | -0,2423 | 108,44 | AAQVTIQSSGTFSSK |  |
|  |  |  |  |  |  | 778,1157 | 2 | 0,4408 | 92,88 | VTGEGCVYLQTSLK |  |
|  |  |  |  |  |  | 783,2813 | 2 | -0,277 | 102,41 | ALLAYAFALAGNQDK |  |
|  |  |  |  |  |  | 857,5693 | 2 | 0,295 | 66,24 | SSSNEEVMFLTVQVK | Oxidation (M) |
|  |  |  |  |  |  | 923,0519 | 2 | 0,0597 | 89,2 | LLIYAVLPTGDVIGDSAK |  |
|  |  |  |  |  |  | 924,9262 | 2 | -0,0353 | 72,33 | QFSFPLSSEPFQGSYK |  |
|  |  |  |  |  |  | 943,0096 | 2 | 0,9643 | 99,54 | VSVQLEASPAFLAVPVEK |  |
|  |  |  |  |  |  | 1031,059 | 2 | 0,0165 | 66,57 | LLLQQVSLPELPGEYSMK | Oxidation (M) |
|  |  |  |  |  |  | 713,2358 | 3 | 0,6368 | 23,19 | HNVYINGITYTPVSSTNEK |  |
|  |  |  |  |  |  | 796,5301 | 3 | 0,3807 | 86,54 | QQNAQGGFSSTQDTVVALHALSK |  |
| 7 | P0DOX5 | Immunoglobulin gamma-1 heavy chain OS=Homo sapiens OX=9606 PE=1 SV=2 | 1052 | 49925 | 7 | 838,3431 | 1 | -0,1601 | 33,42 | ALPAPIEK |  |
|  |  |  |  |  |  | 581,2396 | 2 | -0,1578 | 38,71 | NQVSLTCLVK |  |
|  |  |  |  |  |  | 593,3978 | 2 | -0,8583 | 30,34 | GPSVFPLAPSSK |  |
|  |  |  |  |  |  | 643,7397 | 2 | -0,2017 | 30,12 | EPQVYTLPPSR |  |
|  |  |  |  |  |  | 661,2319 | 2 | -0,2216 | 68,38 | STSGGTAALGCLVK |  |
|  |  |  |  |  |  | 937,1283 | 2 | -0,6725 | 42,09 | TTPPVLDSDGSFFLYSK |  |
|  |  |  |  |  |  | 713,747 | 3 | 0,199 | 41,48 | TPEVTCVVVDVSHEDPEVK |  |
| 8 | P0DOX7 | Immunoglobulin kappa light chain OS=Homo sapiens OX=9606 PE=1 SV=1 | 1012 | 23650 | 7 | 375,3994 | 2 | 0,394 | 27,6 | VTITCR |  |
|  |  |  |  |  |  | 752,2089 | 2 | 0,652 | 78,26 | DSTYSLSSTLTLSK |  |
|  |  |  |  |  |  | 899,273 | 2 | -0,3566 | 63,99 | SGTASVVCLLNNFYPR |  |
|  |  |  |  |  |  | 938,5518 | 2 | 0,1693 | 33,97 | VYACEVTHQGLSSPVTK |  |
|  |  |  |  |  |  | 954,7714 | 2 | -0,3613 | 100,62 | DIQMTQSPSTLSASVGDR | Oxidation (M) |
|  |  |  |  |  |  | 1002,027 | 2 | -0,0022 | 50,63 | GTVAAPSVFIFPPSDEQLK |  |
|  |  |  |  |  |  | 1068,039 | 2 | -0,8976 | 82,43 | VDNALQSGNSQESVTEQDSK |  |
| 9 | P04114 | Apolipoprotein B-100 OS=Homo sapiens OX=9606 GN=APOB PE=1 SV=2 | 937 | 516651 | 32 | 473,0161 | 2 | 0,4838 | 34,71 | LTLDIQNK |  |
|  |  |  |  |  |  | 481,3707 | 2 | 0,2029 | 58,46 | LDVTTSIGR |  |
|  |  |  |  |  |  | 506,9952 | 2 | 0,3431 | 23,17 | TGISPLALIK |  |
|  |  |  |  |  |  | 508,335 | 2 | 0,1169 | 30,95 | VSTAFVYTK |  |
|  |  |  |  |  |  | 509,1479 | 2 | -0,3052 | 37,6 | LATALSLSNK |  |
|  |  |  |  |  |  | 515,2423 | 2 | 0,8675 | 46,1 | LSNVLQQVK |  |
|  |  |  |  |  |  | 518,6531 | 2 | -0,2069 | 20,31 | GAYQNNEIK |  |
|  |  |  |  |  |  | 1039,266 | 1 | -0,3736 | 25,35 | LAPGELTIIL |  |
|  |  |  |  |  |  | 523,5211 | 2 | 0,4308 | 25,26 | IPSVQINFK |  |
|  |  |  |  |  |  | 535,4951 | 2 | -0,6415 | 36,34 | IEIPLPFGGK |  |
|  |  |  |  |  |  | 555,9705 | 2 | 0,4089 | 47,45 | VPQTDMTFR | Oxidation (M) |
|  |  |  |  |  |  | 576,1769 | 2 | -0,2226 | 57,58 | LDFSSQADLR |  |
|  |  |  |  |  |  | 598,9054 | 2 | 0,2208 | 46,34 | NMEVSVATTTK | Oxidation (M) |
|  |  |  |  |  |  | 634,9665 | 2 | 0,205 | 28,46 | DLKVEDIPLAR |  |
|  |  |  |  |  |  | 636,2761 | 2 | -0,1392 | 38,67 | SVSLPSLDPASAK |  |
|  |  |  |  |  |  | 643,937 | 2 | 0,1353 | 78,29 | NTLELSNGVIVK |  |
|  |  |  |  |  |  | 653,5997 | 2 | -0,512 | 38,69 | EFQVPTFTIPK |  |
|  |  |  |  |  |  | 654,6476 | 2 | -0,3955 | 74,65 | GFEPTLEALFGK |  |
|  |  |  |  |  |  | 677,8409 | 2 | -0,0289 | 63,63 | YGMVAQVTQTLK | Oxidation (M) |
|  |  |  |  |  |  | 679,1555 | 2 | 0,4901 | 72,33 | LPYTIITTPPLK |  |
|  |  |  |  |  |  | 700,5026 | 2 | 0,1936 | 83,19 | TLADLTLLDSPIK |  |
|  |  |  |  |  |  | 753,6949 | 2 | 0,602 | 81,72 | IGQDGISTSATTNLK |  |
|  |  |  |  |  |  | 768,1248 | 2 | -0,5496 | 38,61 | CVQSTKPSLMIQK | Oxidation (M) |
|  |  |  |  |  |  | 785,9675 | 2 | 0,0657 | 29,25 | TLQGIPQMIGEVIR | Oxidation (M) |
|  |  |  |  |  |  | 791,5074 | 2 | 0,2135 | 80,61 | AVSMPSFSILGSDVR | Oxidation (M) |
|  |  |  |  |  |  | 796,5306 | 2 | 0,185 | 80,19 | VLLDQLGTTISFER |  |
|  |  |  |  |  |  | 808,8285 | 2 | -0,2397 | 58 | TSSFALNLPTLPEVK |  |
|  |  |  |  |  |  | 873,7443 | 2 | -0,4248 | 49,39 | IEGNLIFDPNNYLPK |  |
|  |  |  |  |  |  | 917,4733 | 2 | -0,0353 | 22,6 | ATFQTPDFIVPLTDLR |  |
|  |  |  |  |  |  | 961,2447 | 2 | 0,5417 | 58,78 | VIGNMGQTMEQLTPELK | 2 Oxidation (M) |
|  |  |  |  |  |  | 982,1138 | 2 | 0,1634 | 88,72 | TILGTMPAFEVSLQALQK | Oxidation (M) |
|  |  |  |  |  |  | 1127,522 | 2 | -0,1273 | 120,73 | AALTELSLGSAYQAMILGVDSK | Oxidation (M) |
| 10 | P02647 | Apolipoprotein A-I OS=Homo sapiens OX=9606 GN=APOA1 PE=1 SV=1 | 810 | 30759 | 15 | 732,1409 | 1 | -0,2365 | 31,06 | DLEEVK |  |
|  |  |  |  |  |  | 391,1426 | 2 | -0,1536 | 30,25 | AHVDALR |  |
|  |  |  |  |  |  | 437,2376 | 2 | 0,0255 | 42,72 | AELQEGAR |  |
|  |  |  |  |  |  | 506,7136 | 2 | -0,1586 | 31,21 | AKPALEDLR |  |
|  |  |  |  |  |  | 524,2706 | 2 | 0,0201 | 48,03 | LSPLGEEMR | Oxidation (M) |
|  |  |  |  |  |  | 608,3939 | 2 | 0,1589 | 61,91 | ATEHLSTLSEK |  |
|  |  |  |  |  |  | 615,696 | 2 | -0,3244 | 47,11 | QGLLPVLESFK |  |
|  |  |  |  |  |  | 618,0851 | 2 | -0,5253 | 59,56 | DLATVYVDVLK |  |
|  |  |  |  |  |  | 626,689 | 2 | -0,2502 | 41,51 | VQPYLDDFQK |  |
|  |  |  |  |  |  | 651,1757 | 2 | -0,3044 | 36,15 | THLAPYSDELR |  |
|  |  |  |  |  |  | 690,9104 | 2 | 0,0977 | 43,62 | VQPYLDDFQKK |  |
|  |  |  |  |  |  | 694,0163 | 2 | 0,3102 | 78,33 | VSFLSALEEYTK |  |
|  |  |  |  |  |  | 1400,347 | 1 | -0,3222 | 40,01 | DYVSQFEGSALGK |  |
|  |  |  |  |  |  | 731,8522 | 2 | -0,1545 | 69,76 | VKDLATVYVDVLK |  |
|  |  |  |  |  |  | 908,1334 | 2 | -0,5912 | 56,48 | DSGRDYVSQFEGSALGK |  |
| 11 | P00738 | Haptoglobin OS=Homo sapiens OX=9606 GN=HP PE=1 SV=1 | 723 | 45861 | 7 | 430,1668 | 2 | 0,8332 | 30,09 | QLVEIEK |  |
|  |  |  |  |  |  | 462,3122 | 2 | 0,0862 | 33,21 | ILGGHLDAK |  |
|  |  |  |  |  |  | 530,7288 | 2 | -0,1027 | 30,14 | VMPICLPSK | Oxidation (M) |
|  |  |  |  |  |  | 1290,257 | 1 | -0,4733 | 30,75 | DIAPTLTLYVGK |  |
|  |  |  |  |  |  | 673,2222 | 2 | -0,2086 | 94,08 | SCAVAEYGVYVK |  |
|  |  |  |  |  |  | 862,4507 | 2 | 0,0799 | 76,03 | YVMLPVADQDQCIR | Oxidation (M) |
|  |  |  |  |  |  | 730,3176 | 3 | 0,8858 | 36,73 | SPVGVQPILNEHTFCAGMSK | Oxidation (M) |
| 12 | P01860 | Immunoglobulin heavy constant gamma 3 OS=Homo sapiens OX=9606 GN=IGHG3 PE=1 SV=2 | 581 | 42287 | 6 | 838,3431 | 1 | -0,1601 | 33,42 | ALPAPIEK |  |
|  |  |  |  |  |  | 581,2396 | 2 | -0,1578 | 38,71 | NQVSLTCLVK |  |
|  |  |  |  |  |  | 593,7118 | 2 | -0,0817 | 26,57 | SCDTPPPCPR |  |
|  |  |  |  |  |  | 643,7397 | 2 | -0,2017 | 30,12 | EPQVYTLPPSR |  |
|  |  |  |  |  |  | 644,2191 | 2 | -0,2205 | 36,13 | GPSVFPLAPCSR |  |
|  |  |  |  |  |  | 661,2319 | 2 | -0,2216 | 68,38 | STSGGTAALGCLVK |  |
| 13 | P01859 | Immunoglobulin heavy constant gamma 2 OS=Homo sapiens OX=9606 GN=IGHG2 PE=1 SV=2 | 524 | 36505 | 5 | 581,2396 | 2 | -0,1578 | 38,71 | NQVSLTCLVK |  |
|  |  |  |  |  |  | 643,7397 | 2 | -0,2017 | 30,12 | EPQVYTLPPSR |  |
|  |  |  |  |  |  | 644,2191 | 2 | -0,2205 | 36,13 | GPSVFPLAPCSR |  |
|  |  |  |  |  |  | 712,1288 | 2 | -0,4594 | 56,55 | STSESTAALGCLVK |  |
|  |  |  |  |  |  | 961,1843 | 2 | -0,5274 | 40,85 | TTPPMLDSDGSFFLYSK | Oxidation (M) |
| 14 | P0DOY2 | Immunoglobulin lambda constant 2 OS=Homo sapiens OX=9606 GN=IGLC2 PE=1 SV=1 | 443 | 11458 | 3 | 864,2033 | 1 | -0,1735 | 24,41 | TVAPTECS |  |
|  |  |  |  |  |  | 495,8189 | 2 | 0,1203 | 60,81 | AGVETTTPSK |  |
|  |  |  |  |  |  | 993,3918 | 2 | -0,2414 | 46,72 | AAPSVTLFPPSSEELQANK |  |
| 15 | P01861 | Immunoglobulin heavy constant gamma 4 OS=Homo sapiens OX=9606 GN=IGHG4 PE=1 SV=1 | 425 | 36431 | 5 | 581,2396 | 2 | -0,1578 | 38,71 | NQVSLTCLVK |  |
|  |  |  |  |  |  | 644,2191 | 2 | -0,2205 | 36,13 | GPSVFPLAPCSR |  |
|  |  |  |  |  |  | 712,1288 | 2 | -0,4594 | 56,55 | STSESTAALGCLVK |  |
|  |  |  |  |  |  | 947,0737 | 2 | 0,2456 | 23,74 | EPQVYTLPPSQEEMTK | Oxidation (M) |
|  |  |  |  |  |  | 951,5059 | 2 | 0,0765 | 42,59 | TTPPVLDSDGSFFLYSR |  |
| 16 | P01876 | Immunoglobulin heavy constant alpha 1 OS=Homo sapiens OX=9606 GN=IGHA1 PE=1 SV=2 | 396 | 38486 | 4 | 470,7708 | 2 | 0,0496 | 23,29 | SAVQGPPER |  |
|  |  |  |  |  |  | 688,1357 | 2 | -0,3558 | 43,56 | TFTCTAAYPESK |  |
|  |  |  |  |  |  | 918,1785 | 2 | -0,6001 | 28,2 | QEPSQGTTTFAVTSILR |  |
|  |  |  |  |  |  | 1585,258 | 2 | 0,9922 | 44,11 | NFPPSQDASGDLYTTSSQLTLPATQCLAGK |  |
| 17 | P05155 | Plasma protease C1 inhibitor OS=Homo sapiens OX=9606 GN=SERPING1 PE=1 SV=2 | 347 | 55347 | 5 | 455,5909 | 2 | -0,2883 | 46,53 | TLYSSSPR |  |
|  |  |  |  |  |  | 558,5795 | 2 | -0,4379 | 35,92 | LLDSLPSDTR |  |
|  |  |  |  |  |  | 593,2719 | 2 | -0,1625 | 39,47 | FQPTLLTLPR |  |
|  |  |  |  |  |  | 632,4605 | 2 | -0,7647 | 53,29 | TNLESILSYPK |  |
|  |  |  |  |  |  | 805,2857 | 2 | -0,2136 | 80,96 | LEDMEQALSPSVFK | Oxidation (M) |
| 18 | P0C0L4 | Complement C4-A OS=Homo sapiens OX=9606 GN=C4A PE=1 SV=2 | 221 | 194261 | 5 | 518,5153 | 2 | 0,4763 | 35,35 | LGQYASPTAK |  |
|  |  |  |  |  |  | 558,1611 | 2 | 0,6934 | 75,71 | VGDTLNLNLR |  |
|  |  |  |  |  |  | 771,7089 | 2 | 0,5936 | 77,35 | VLSLAQEQVGGSPEK |  |
|  |  |  |  |  |  | 1113,399 | 2 | 0,673 | 85,79 | ALEILQEEDLIDEDDIPVR |  |
|  |  |  |  |  |  | 1242,251 | 2 | 0,1964 | 69,24 | VTASDPLDTLGSEGALSPGGVASLLR |  |
| 19 | P02790 | Hemopexin OS=Homo sapiens OX=9606 GN=HPX PE=1 SV=2 | 197 | 52385 | 5 | 571,3995 | 2 | 0,203 | 60,37 | GGYTLVSGYPK |  |
|  |  |  |  |  |  | 580,1707 | 2 | 0,8633 | 24,98 | DYFMPCPGR | Oxidation (M) |
|  |  |  |  |  |  | 610,5521 | 2 | -0,509 | 21,78 | NFPSPVDAAFR |  |
|  |  |  |  |  |  | 635,1028 | 2 | 0,5237 | 24,34 | FDPVRGEVPPR |  |
|  |  |  |  |  |  | 748,5524 | 2 | 0,4188 | 55,34 | YYCFQGNQFLR |  |
| 20 | P00450 | Ceruloplasmin OS=Homo sapiens OX=9606 GN=CP PE=1 SV=1 | 174 | 122983 | 4 | 602,082 | 2 | -0,371 | 33,94 | EYTDASFTNR |  |
|  |  |  |  |  |  | 686,1049 | 2 | -0,5606 | 53,74 | GAYPLSIEPIGVR |  |
|  |  |  |  |  |  | 735,8779 | 2 | 0,0229 | 83 | EVGPTNADPVCLAK |  |
|  |  |  |  |  |  | 952,6543 | 2 | 0,4808 | 62,14 | NNEGTYYSPNYNPQSR |  |
| 21 | P01011 | Alpha-1-antichymotrypsin OS=Homo sapiens OX=9606 GN=SERPINA3 PE=1 SV=2 | 173 | 47792 | 2 | 608,1129 | 2 | -0,5121 | 53,28 | ITLLSALVETR |  |
|  |  |  |  |  |  | 946,8637 | 2 | 0,8492 | 89,24 | LYGSEAFATDFQDSAAAK |  |
| 22 | P01042 | Kininogen-1 OS=Homo sapiens OX=9606 GN=KNG1 PE=1 SV=2 | 114 | 72996 | 6 | 502,126 | 2 | -0,3235 | 38,93 | QVVAGLNFR |  |
|  |  |  |  |  |  | 515,6071 | 2 | -0,3288 | 32,17 | YFIDFVAR |  |
|  |  |  |  |  |  | 626,4208 | 2 | 0,2452 | 23,97 | TVGSDTFYSFK |  |
|  |  |  |  |  |  | 800,3777 | 2 | 0,0009 | 28,28 | IASFSQNCDIYPGK |  |
|  |  |  |  |  |  | 938,2842 | 2 | 0,6831 | 51,4 | YNSQNQSNNQFVLYR |  |
|  |  |  |  |  |  | 1070,015 | 2 | -0,0581 | 30,6 | DIPTNSPELEETLTHTITK |  |
| 23 | P01871 | Immunoglobulin heavy constant mu OS=Homo sapiens OX=9606 GN=IGHM PE=1 SV=4 | 104 | 50093 | 2 | 625,6065 | 2 | 0,5699 | 47,82 | LICQATGFSPR |  |
|  |  |  |  |  |  | 809,6654 | 2 | 0,5157 | 84,92 | QVGSGVTTDQVQAEAK |  |
| 24 | P0DOX2 | Immunoglobulin alpha-2 heavy chain OS=Homo sapiens OX=9606 PE=1 SV=2 | 93 | 49816 | 3 | 470,8153 | 2 | -0,8453 | 27,54 | SAVEGPPER |  |
|  |  |  |  |  |  | 659,525 | 2 | -0,5306 | 60,74 | AEDTAVYYCAR |  |
|  |  |  |  |  |  | 710,5313 | 2 | 0,3226 | 67,95 | GTTVTVSSASPTSPK |  |
| 25 | A0A0B4J1X5 | Immunoglobulin heavy variable 3-74 OS=Homo sapiens OX=9606 GN=IGHV3-74 PE=3 SV=1 | 92 | 13002 | 2 | 659,525 | 2 | -0,5306 | 60,74 | AEDTAVYYCAR |  |
|  |  |  |  |  |  | 685,2455 | 2 | 0,7898 | 64,25 | NTLYLQMNSLR | Oxidation (M) |
| 26 | P08603 | Complement factor H OS=Homo sapiens OX=9606 GN=CFH PE=1 SV=4 | 90 | 143680 | 4 | 487,9526 | 2 | 0,458 | 20,55 | FVCNSGYK |  |
|  |  |  |  |  |  | 741,1879 | 2 | -0,3409 | 29,79 | CFEGFGIDGPAIAK |  |
|  |  |  |  |  |  | 1011,301 | 2 | 0,6191 | 91,92 | DTSCVNPPTVQNAYIVSR |  |
|  |  |  |  |  |  | 1013,209 | 2 | 0,4794 | 20,54 | TDCLSLPSFENAIPMGEK | Oxidation (M) |
| 27 | P0DP03 | Immunoglobulin heavy variable 3-30-5 OS=Homo sapiens OX=9606 GN=IGHV3-30-5 PE=3 SV=1 | 89 | 13110 | 2 | 645,8616 | 2 | 0,1488 | 51,77 | AEDTAVYYCAK |  |
|  |  |  |  |  |  | 685,2455 | 2 | 0,7898 | 64,25 | NTLYLQMNSLR | Oxidation (M) |
| 28 | P04196 | Histidine-rich glycoprotein OS=Homo sapiens OX=9606 GN=HRG PE=1 SV=1 | 88 | 60510 | 2 | 841,6846 | 2 | -0,429 | 51,56 | DSPVLIDFFEDTER |  |
|  |  |  |  |  |  | 913,0073 | 2 | 0,1172 | 62,96 | ADLFYDVEALDLESPK |  |
| 29 | P19827 | Inter-alpha-trypsin inhibitor heavy chain H1 OS=Homo sapiens OX=9606 GN=ITIH1 PE=1 SV=3 | 81 | 101782 | 3 | 581,073 | 2 | 0,519 | 47,58 | EVAFDLEIPK |  |
|  |  |  |  |  |  | 736,1475 | 2 | 0,5653 | 57,62 | QYYEGSEIVVAGR |  |
|  |  |  |  |  |  | 1002,726 | 2 | 0,4309 | 30,85 | GSLVQASEANLQAAQDFVR |  |
| 30 | P0CG04 | Immunoglobulin lambda constant 1 OS=Homo sapiens OX=9606 GN=IGLC1 PE=1 SV=1 | 69 | 11512 | 2 | 864,2033 | 1 | -0,1735 | 24,41 | TVAPTECS |  |
|  |  |  |  |  |  | 1021,861 | 2 | -0,3256 | 51,16 | ANPTVTLFPPSSEELQANK |  |
| 31 | P10909 | Clusterin OS=Homo sapiens OX=9606 GN=CLU PE=1 SV=1 | 58 | 53031 | 3 | 644,9628 | 2 | 0,2804 | 38,08 | ELDESLQVAER |  |
|  |  |  |  |  |  | 937,5903 | 2 | 0,1828 | 51,34 | LFDSDPITVTVPVEVSR |  |
|  |  |  |  |  |  | 772,302 | 3 | 0,7141 | 29,35 | VTTVASHTSDSDVPSGVTEVVVK |  |

**Table S2.3** PEG-MWCNTs Hard Corona R1

| **Prot. Number** | **Accession Number** | **Protein Name** | **Mascot Score** | **Mr** | **N° pep** | **m/z** | **z** | **Pep. error** | **Pep. Score** | **Pep. sequence** | **Pep. Modification** |
| --- | --- | --- | --- | --- | --- | --- | --- | --- | --- | --- | --- |
| 1 | P02768 | Serum albumin OS=Homo sapiens OX=9606 GN=ALB PE=1 SV=2 | 8190 | 71317 | 34 | 673,5054 | 1 | 0,1274 | 35,06 | AWAVAR |  |
|  |  |  |  |  |  | 772,4233 | 1 | -0,0152 | 20,03 | AACLLPK |  |
|  |  |  |  |  |  | 395,1814 | 2 | -0,1162 | 21,95 | LVTDLTK |  |
|  |  |  |  |  |  | 440,6456 | 2 | -0,1571 | 32,39 | AEFAEVSK |  |
|  |  |  |  |  |  | 464,2194 | 2 | -0,0618 | 39,7 | YLYEIAR |  |
|  |  |  |  |  |  | 467,2212 | 2 | -0,0836 | 50,55 | LCTVATLR |  |
|  |  |  |  |  |  | 470,6757 | 2 | -0,1042 | 38,51 | DDNPNLPR |  |
|  |  |  |  |  |  | 476,2887 | 2 | 0,1283 | 33,96 | DLGEENFK |  |
|  |  |  |  |  |  | 480,7385 | 2 | -0,0929 | 49,78 | FQNALLVR |  |
|  |  |  |  |  |  | 984,3772 | 1 | -0,1112 | 20,78 | TYETTLEK |  |
|  |  |  |  |  |  | 500,8244 | 2 | 0,0378 | 51,69 | QTALVELVK |  |
|  |  |  |  |  |  | 1013,253 | 1 | -0,3463 | 22,61 | LVAASQAALGL |  |
|  |  |  |  |  |  | 1017,482 | 1 | -0,0547 | 49,26 | SLHTLFGDK |  |
|  |  |  |  |  |  | 537,8098 | 2 | 0,0697 | 29,31 | LDELRDEGK |  |
|  |  |  |  |  |  | 564,9659 | 2 | 0,2258 | 53,78 | KQTALVELVK |  |
|  |  |  |  |  |  | 569,4252 | 2 | -0,6549 | 31,72 | CCTESLVNR |  |
|  |  |  |  |  |  | 571,2188 | 2 | -0,2636 | 50,12 | KLVAASQAALGL |  |
|  |  |  |  |  |  | 1149,41 | 1 | -0,2055 | 31,83 | LVNEVTEFAK |  |
|  |  |  |  |  |  | 1226,404 | 1 | -0,2009 | 52,13 | FKDLGEENFK |  |
|  |  |  |  |  |  | 656,7837 | 2 | 0,8182 | 56,04 | HPDYSVVLLLR |  |
|  |  |  |  |  |  | 679,4874 | 2 | -0,6621 | 85,95 | AVMDDFAAFVEK | Oxidation (M) |
|  |  |  |  |  |  | 686,1237 | 2 | -0,3267 | 39,66 | AAFTECCQAADK |  |
|  |  |  |  |  |  | 717,9291 | 2 | 0,3175 | 46,48 | ETYGEMADCCAK |  |
|  |  |  |  |  |  | 722,1298 | 2 | -0,3898 | 73,93 | YICENQDSISSK |  |
|  |  |  |  |  |  | 725,955 | 2 | 0,3743 | 43,89 | ETYGEMADCCAK | Oxidation (M) |
|  |  |  |  |  |  | 734,7416 | 2 | 0,6328 | 39,92 | RHPDYSVVLLLR |  |
|  |  |  |  |  |  | 749,7142 | 2 | -0,1573 | 77,69 | TCVADESAENCDK |  |
|  |  |  |  |  |  | 500,7803 | 3 | 0,6945 | 37,75 | ADDKETCFAEEGK |  |
|  |  |  |  |  |  | 756,369 | 2 | -0,112 | 68,45 | VPQVSTPTLVEVSR |  |
|  |  |  |  |  |  | 547,1195 | 3 | -0,594 | 51,19 | KVPQVSTPTLVEVSR |  |
|  |  |  |  |  |  | 820,6151 | 2 | 0,4403 | 33,83 | DVFLGMFLYEYAR | Oxidation (M) |
|  |  |  |  |  |  | 820,6235 | 2 | 0,3019 | 105,58 | KVPQVSTPTLVEVSR |  |
|  |  |  |  |  |  | 820,648 | 2 | 0,5061 | 70,09 | DVFLGMFLYEYAR | Oxidation (M) |
|  |  |  |  |  |  | 820,662 | 2 | 0,3789 | 63,38 | KVPQVSTPTLVEVSR |  |
|  |  |  |  |  |  | 829,4471 | 2 | 0,1344 | 47,62 | QNCELFEQLGEYK |  |
|  |  |  |  |  |  | 637,7223 | 3 | 0,2206 | 42,32 | RPCFSALEVDETYVPK |  |
|  |  |  |  |  |  | 682,2294 | 3 | -0,4218 | 42,55 | VFDEFKPLVEEPQNLIK |  |
|  |  |  |  |  |  | 997,7152 | 3 | 0,7917 | 99,43 | SHCIAEVENDEMPADLPSLAADFVESK | Oxidation (M) |
| 2 | P04114 | Apolipoprotein B-100 OS=Homo sapiens OX=9606 GN=APOB PE=1 SV=2 | 3417 | 516651 | 77 | 353,7564 | 2 | 0,1172 | 38,25 | ANLFNK |  |
|  |  |  |  |  |  | 365,7801 | 2 | 0,1072 | 38,07 | LVTELR |  |
|  |  |  |  |  |  | 371,2408 | 2 | 0,0125 | 35,93 | GVISIPR |  |
|  |  |  |  |  |  | 380,2952 | 2 | 0,1221 | 34,63 | ALVDTLK |  |
|  |  |  |  |  |  | 386,0338 | 2 | 0,5469 | 29,82 | NLLVALK |  |
|  |  |  |  |  |  | 393,3323 | 2 | 0,2745 | 23,58 | FFGEGTK |  |
|  |  |  |  |  |  | 401,4072 | 2 | 0,3202 | 27,16 | FIIPSPK |  |
|  |  |  |  |  |  | 410,562 | 2 | 0,6556 | 24,92 | LSLPDFK |  |
|  |  |  |  |  |  | 415,2184 | 2 | 0,9722 | 41,72 | LGNNPVSK |  |
|  |  |  |  |  |  | 431,4023 | 2 | 0,3145 | 39,44 | ITLPDFR |  |
|  |  |  |  |  |  | 457,1994 | 2 | -0,0862 | 24,84 | FFGEGTKK |  |
|  |  |  |  |  |  | 462,2345 | 2 | -0,0216 | 36,88 | QSFDLSVK |  |
|  |  |  |  |  |  | 467,3485 | 2 | 0,2009 | 42,83 | IEDGTLASK |  |
|  |  |  |  |  |  | 473,1254 | 2 | 0,7024 | 39,28 | LTLDIQNK |  |
|  |  |  |  |  |  | 476,0102 | 2 | 0,5189 | 46,58 | FVTQAEGAK |  |
|  |  |  |  |  |  | 481,4268 | 2 | 0,315 | 57,51 | LDVTTSIGR |  |
|  |  |  |  |  |  | 487,2556 | 2 | 0,0091 | 47,36 | QIDDIDVR |  |
|  |  |  |  |  |  | 500,3621 | 2 | 0,2353 | 52,27 | MGLAFESTK | Oxidation (M) |
|  |  |  |  |  |  | 507,063 | 2 | 0,4786 | 66,12 | TGISPLALIK |  |
|  |  |  |  |  |  | 508,6934 | 2 | 0,8337 | 56,54 | VSTAFVYTK |  |
|  |  |  |  |  |  | 509,6334 | 2 | 0,6658 | 37,68 | LATALSLSNK |  |
|  |  |  |  |  |  | 511,3314 | 2 | 0,1242 | 32,8 | NNALDFVTK |  |
|  |  |  |  |  |  | 514,8282 | 2 | 0,0392 | 47,84 | LSNVLQQVK |  |
|  |  |  |  |  |  | 517,7839 | 2 | 0,0341 | 52,78 | DNVFDGLVR |  |
|  |  |  |  |  |  | 1039,312 | 1 | -0,3277 | 24,29 | LAPGELTIIL |  |
|  |  |  |  |  |  | 523,4115 | 2 | 0,2117 | 37,47 | IPSVQINFK |  |
|  |  |  |  |  |  | 524,4465 | 2 | 0,3137 | 37,49 | FPEVDVLTK |  |
|  |  |  |  |  |  | 535,6122 | 2 | -0,4072 | 20,81 | IEIPLPFGGK |  |
|  |  |  |  |  |  | 536,8048 | 2 | 0,1043 | 21,41 | QTEATMTFK | Oxidation (M) |
|  |  |  |  |  |  | 539,0716 | 2 | 0,5373 | 50,98 | LVGFIDDAVK |  |
|  |  |  |  |  |  | 555,6479 | 2 | -0,2363 | 26,11 | VPQTDMTFR | Oxidation (M) |
|  |  |  |  |  |  | 557,5935 | 2 | 0,6003 | 57,51 | LAAYLMLMR | 2 Oxidation (M) |
|  |  |  |  |  |  | 563,1589 | 2 | -0,2866 | 60,25 | LIDVISMYR | Oxidation (M) |
|  |  |  |  |  |  | 587,1067 | 2 | 0,6228 | 39,8 | YENYELTLK |  |
|  |  |  |  |  |  | 589,1214 | 2 | 0,65 | 79,05 | GNVATEISTER |  |
|  |  |  |  |  |  | 593,7539 | 2 | -0,1057 | 21,42 | SNTVASLHTEK |  |
|  |  |  |  |  |  | 598,8614 | 2 | 0,1328 | 64,13 | NMEVSVATTTK | Oxidation (M) |
|  |  |  |  |  |  | 601,5284 | 2 | 0,396 | 32,04 | LTISEQNIQR |  |
|  |  |  |  |  |  | 615,0657 | 2 | 0,5979 | 71,73 | NSEEFAAAMSR | Oxidation (M) |
|  |  |  |  |  |  | 618,5219 | 2 | 0,4351 | 76,82 | ENFAGEATLQR |  |
|  |  |  |  |  |  | 621,6328 | 2 | -0,3781 | 57,57 | ATGVLYDYVNK |  |
|  |  |  |  |  |  | 635,2034 | 2 | 0,6786 | 21,51 | DLKVEDIPLAR |  |
|  |  |  |  |  |  | 636,6195 | 2 | 0,5476 | 49,71 | SVSLPSLDPASAK |  |
|  |  |  |  |  |  | 643,7904 | 2 | -0,1579 | 85,93 | NTLELSNGVIVK |  |
|  |  |  |  |  |  | 435,7225 | 3 | 0,4725 | 46,54 | KGNVATEISTER |  |
|  |  |  |  |  |  | 654,355 | 2 | 0,9985 | 55,2 | EFQVPTFTIPK |  |
|  |  |  |  |  |  | 654,8641 | 2 | 0,0376 | 65,36 | GFEPTLEALFGK |  |
|  |  |  |  |  |  | 655,4266 | 2 | 0,1462 | 48,7 | EVGTVLSQVYSK |  |
|  |  |  |  |  |  | 444,9699 | 3 | -0,7643 | 32,26 | VRESDEETQIK |  |
|  |  |  |  |  |  | 669,8635 | 2 | 0,084 | 29,96 | ESQLPTVMDFR | Oxidation (M) |
|  |  |  |  |  |  | 672,1825 | 2 | -0,2934 | 24,5 | SKPTVSSSMEFK | Oxidation (M) |
|  |  |  |  |  |  | 677,7505 | 2 | -0,2098 | 66,47 | YGMVAQVTQTLK | Oxidation (M) |
|  |  |  |  |  |  | 678,9148 | 2 | 0,0086 | 38,32 | LPYTIITTPPLK |  |
|  |  |  |  |  |  | 681,1511 | 2 | 0,5883 | 65,35 | INNQLTLDSNTK |  |
|  |  |  |  |  |  | 694,0571 | 2 | 0,3232 | 84,08 | IAELSATAQEIIK |  |
|  |  |  |  |  |  | 700,4442 | 2 | 0,2323 | 75,79 | AASGTTGTYQEWK |  |
|  |  |  |  |  |  | 700,6396 | 2 | 0,4678 | 79,35 | TLADLTLLDSPIK |  |
|  |  |  |  |  |  | 706,5608 | 2 | 0,2877 | 78,16 | QTIIVVLENVQR |  |
|  |  |  |  |  |  | 716,1715 | 2 | 0,5468 | 32,1 | ALVEQGFTVPEIK |  |
|  |  |  |  |  |  | 722,5419 | 2 | -0,6046 | 28,8 | LQSTTVMNPYMK | 2 Oxidation (M) |
|  |  |  |  |  |  | 753,3666 | 2 | -0,0547 | 75,47 | IGQDGISTSATTNLK |  |
|  |  |  |  |  |  | 512,7242 | 3 | 0,366 | 22,31 | CVQSTKPSLMIQK | Oxidation (M) |
|  |  |  |  |  |  | 780,1829 | 2 | 0,5627 | 85,86 | ITENDIQIALDDAK |  |
|  |  |  |  |  |  | 786,1479 | 2 | 0,4264 | 74,34 | TLQGIPQMIGEVIR | Oxidation (M) |
|  |  |  |  |  |  | 791,4653 | 2 | 0,1293 | 94,6 | AVSMPSFSILGSDVR | Oxidation (M) |
|  |  |  |  |  |  | 796,4426 | 2 | 0,0089 | 86,09 | VLLDQLGTTISFER |  |
|  |  |  |  |  |  | 801,8619 | 2 | 0,8633 | 53,76 | VSALLTPAEQTGTWK |  |
|  |  |  |  |  |  | 809,3064 | 2 | 0,7161 | 68,09 | TSSFALNLPTLPEVK |  |
|  |  |  |  |  |  | 815,6672 | 2 | 0,5807 | 59,24 | MTSNFPVDLSDYPK | Oxidation (M) |
|  |  |  |  |  |  | 817,1213 | 2 | 0,3972 | 39,52 | ALYWVNGQVPDGVSK |  |
|  |  |  |  |  |  | 829,4638 | 2 | 0,0447 | 77,04 | SVSDGIAALDLNAVANK |  |
|  |  |  |  |  |  | 834,0158 | 2 | 0,0981 | 56,09 | GIISALLVPPETEEAK |  |
|  |  |  |  |  |  | 862,6556 | 2 | 0,3663 | 64,54 | IVQILPWEQNEQVK |  |
|  |  |  |  |  |  | 961,2659 | 2 | 0,5839 | 75,02 | VIGNMGQTMEQLTPELK | 2 Oxidation (M) |
|  |  |  |  |  |  | 982,2103 | 2 | 0,3565 | 101,28 | TILGTMPAFEVSLQALQK | Oxidation (M) |
|  |  |  |  |  |  | 1077,748 | 2 | 0,5625 | 28,64 | YTYNYEAESSSGVPGTADSR |  |
|  |  |  |  |  |  | 1083,029 | 2 | 0,1081 | 98,48 | TQFNNNEYSQDLDAYNTK |  |
| 3 | P02647 | Apolipoprotein A-I OS=Homo sapiens OX=9606 GN=APOA1 PE=1 SV=1 | 2044 | 30759 | 20 | 573,6193 | 1 | 0,2587 | 23,95 | LEALK |  |
|  |  |  |  |  |  | 732,2402 | 1 | -0,1372 | 36,34 | DLEEVK |  |
|  |  |  |  |  |  | 391,3403 | 2 | 0,2419 | 28,7 | AHVDALR |  |
|  |  |  |  |  |  | 437,1813 | 2 | -0,0871 | 21,69 | AELQEGAR |  |
|  |  |  |  |  |  | 449,0126 | 2 | 0,5343 | 20,8 | LHELQEK |  |
|  |  |  |  |  |  | 506,6532 | 2 | -0,2794 | 25,14 | AKPALEDLR |  |
|  |  |  |  |  |  | 524,2153 | 2 | -0,0905 | 38,15 | LSPLGEEMR | Oxidation (M) |
|  |  |  |  |  |  | 608,4033 | 2 | 0,1778 | 54,56 | ATEHLSTLSEK |  |
|  |  |  |  |  |  | 615,9518 | 2 | 0,187 | 42,34 | QGLLPVLESFK |  |
|  |  |  |  |  |  | 1235,866 | 1 | 0,1779 | 46,56 | DLATVYVDVLK |  |
|  |  |  |  |  |  | 626,4291 | 2 | -0,7699 | 47,53 | VQPYLDDFQK |  |
|  |  |  |  |  |  | 650,3107 | 2 | 0,0467 | 37,08 | WQEEMELYR | Oxidation (M) |
|  |  |  |  |  |  | 651,3519 | 2 | 0,0482 | 38,62 | THLAPYSDELR |  |
|  |  |  |  |  |  | 690,7454 | 2 | -0,2323 | 36,27 | VQPYLDDFQKK |  |
|  |  |  |  |  |  | 693,7026 | 2 | -0,3171 | 80,8 | VSFLSALEEYTK |  |
|  |  |  |  |  |  | 1400,496 | 1 | -0,1733 | 80,05 | DYVSQFEGSALGK |  |
|  |  |  |  |  |  | 732,0529 | 2 | 0,2469 | 59,23 | VKDLATVYVDVLK |  |
|  |  |  |  |  |  | 806,896 | 2 | -0,0006 | 107,7 | LLDNWDSVTSTFSK |  |
|  |  |  |  |  |  | 908,6892 | 2 | 0,5204 | 73,03 | DSGRDYVSQFEGSALGK |  |
|  |  |  |  |  |  | 967,202 | 2 | 0,463 | 108,02 | EQLGPVTQEFWDNLEK |  |
| 4 | P01024 | Complement C3 OS=Homo sapiens OX=9606 GN=C3 PE=1 SV=2 | 1340 | 188569 | 24 | 805,5962 | 1 | 0,1144 | 37,89 | TFISPIK |  |
|  |  |  |  |  |  | 417,4905 | 2 | 0,4858 | 41,57 | LPYSVVR |  |
|  |  |  |  |  |  | 422,0299 | 2 | 0,5178 | 27,25 | VVLVAVDK |  |
|  |  |  |  |  |  | 436,4108 | 2 | 0,2896 | 29,28 | QGALELIK |  |
|  |  |  |  |  |  | 453,0523 | 2 | 0,5358 | 26,79 | GVFVLNKK |  |
|  |  |  |  |  |  | 480,4299 | 2 | 0,3118 | 41,12 | EALKLEEK |  |
|  |  |  |  |  |  | 532,4134 | 2 | 0,2743 | 26,46 | DSCVGSLVVK |  |
|  |  |  |  |  |  | 542,2274 | 2 | -0,1106 | 56,87 | GYTQQLAFR |  |
|  |  |  |  |  |  | 570,6582 | 2 | 0,7684 | 26,25 | FYYIYNEK |  |
|  |  |  |  |  |  | 576,8929 | 2 | 0,1738 | 35,21 | QPSSAFAAFVK |  |
|  |  |  |  |  |  | 398,1436 | 3 | 0,8309 | 59,27 | SDDKVTLEER |  |
|  |  |  |  |  |  | 606,2274 | 2 | -0,2057 | 39,29 | KGYTQQLAFR |  |
|  |  |  |  |  |  | 641,3201 | 2 | 0,0371 | 75,24 | ENEGFTVTAEGK |  |
|  |  |  |  |  |  | 645,5319 | 2 | 0,4484 | 57,94 | SGSDEVQVGQQR |  |
|  |  |  |  |  |  | 668,7059 | 2 | 0,6739 | 60,39 | APSTWLTAYVVK |  |
|  |  |  |  |  |  | 685,8627 | 2 | -0,0134 | 53,58 | TIYTPGSTVLYR |  |
|  |  |  |  |  |  | 701,6194 | 2 | 0,3963 | 39,87 | SSLSVPYVIVPLK |  |
|  |  |  |  |  |  | 828,4726 | 2 | 0,0706 | 80 | TVMVNIENPEGIPVK | Oxidation (M) |
|  |  |  |  |  |  | 829,6597 | 2 | 0,5482 | 31,65 | AGDFLEANYMNLQR | Oxidation (M) |
|  |  |  |  |  |  | 895,4548 | 2 | 0,0387 | 106,52 | DICEEQVNSLPGSITK |  |
|  |  |  |  |  |  | 619,5592 | 3 | 0,8075 | 68,56 | SEETKENEGFTVTAEGK |  |
|  |  |  |  |  |  | 1083,817 | 2 | 0,6232 | 62,1 | AYYENSPQQVFSTEFEVK |  |
|  |  |  |  |  |  | 1086,852 | 2 | -0,3847 | 151,21 | ILLQGTPVAQMTEDAVDAER | Oxidation (M) |
|  |  |  |  |  |  | 919,3348 | 3 | 0,6995 | 36,11 | EGVQKEDIPPADLSDQVPDTESETR |  |
| 5 | P01834 | Immunoglobulin kappa constant OS=Homo sapiens OX=9606 GN=IGKC PE=1 SV=2 | 1073 | 11929 | 5 | 751,9858 | 2 | 0,206 | 80,28 | DSTYSLSSTLTLSK |  |
|  |  |  |  |  |  | 899,3704 | 2 | -0,1618 | 69,86 | SGTASVVCLLNNFYPR |  |
|  |  |  |  |  |  | 938,6497 | 2 | 0,3651 | 75,65 | VYACEVTHQGLSSPVTK |  |
|  |  |  |  |  |  | 973,5646 | 2 | 0,0949 | 60,77 | TVAAPSVFIFPPSDEQLK |  |
|  |  |  |  |  |  | 712,7479 | 3 | 0,2603 | 66,98 | VDNALQSGNSQESVTEQDSK |  |
| 6 | P01009 | Alpha-1-antitrypsin OS=Homo sapiens OX=9606 GN=SERPINA1 PE=1 SV=3 | 952 | 46878 | 11 | 686,5273 | 1 | 0,0826 | 22,85 | IVDLVK |  |
|  |  |  |  |  |  | 390,2949 | 2 | 0,1706 | 21,78 | SPLFMGK |  |
|  |  |  |  |  |  | 444,9235 | 2 | 0,336 | 25,18 | AVLTIDEK |  |
|  |  |  |  |  |  | 461,9119 | 2 | 0,3901 | 26,1 | FLENEDR |  |
|  |  |  |  |  |  | 505,1454 | 2 | 0,7838 | 29,33 | QINDYVEK |  |
|  |  |  |  |  |  | 508,2525 | 2 | -0,1169 | 60,81 | SVLGQLGITK |  |
|  |  |  |  |  |  | 539,0725 | 2 | 0,5204 | 55,16 | LSSWVLLMK |  |
|  |  |  |  |  |  | 360,3827 | 3 | 0,606 | 25,42 | FLENEDRR |  |
|  |  |  |  |  |  | 547,1626 | 2 | 0,7058 | 49,23 | LSSWVLLMK | Oxidation (M) |
|  |  |  |  |  |  | 555,6366 | 2 | -0,3382 | 58,44 | LSITGTYDLK |  |
|  |  |  |  |  |  | 917,4222 | 2 | -0,0857 | 92,74 | VFSNGADLSGVTEEAPLK |  |
|  |  |  |  |  |  | 1146,256 | 2 | 0,3752 | 85,6 | GTEAAGAMFLEAIPMSIPPEVK | 2 Oxidation (M) |
| 7 | P0DOX5 | Immunoglobulin gamma-1 heavy chain OS=Homo sapiens OX=9606 PE=1 SV=2 | 906 | 49925 | 9 | 420,1064 | 2 | 0,7022 | 35,58 | ALPAPIEK |  |
|  |  |  |  |  |  | 851,4212 | 1 | -0,0079 | 30,6 | DTLMISR | Oxidation (M) |
|  |  |  |  |  |  | 581,3003 | 2 | -0,0362 | 31,87 | NQVSLTCLVK |  |
|  |  |  |  |  |  | 593,5921 | 2 | -0,4697 | 43,3 | GPSVFPLAPSSK |  |
|  |  |  |  |  |  | 643,9451 | 2 | 0,2089 | 28,61 | EPQVYTLPPSR |  |
|  |  |  |  |  |  | 652,7553 | 2 | 0,8933 | 58,98 | LSCAASGFTFSR |  |
|  |  |  |  |  |  | 661,317 | 2 | -0,0514 | 84,07 | STSGGTAALGCLVK |  |
|  |  |  |  |  |  | 839,7802 | 2 | 0,751 | 57,8 | FNWYVDGVEVHNAK |  |
|  |  |  |  |  |  | 937,4734 | 2 | 0,0177 | 58,76 | TTPPVLDSDGSFFLYSK |  |
| 8 | P0DOX7 | Immunoglobulin kappa light chain OS=Homo sapiens OX=9606 PE=1 SV=1 | 824 | 23650 | 6 | 751,9858 | 2 | 0,206 | 80,28 | DSTYSLSSTLTLSK |  |
|  |  |  |  |  |  | 899,3704 | 2 | -0,1618 | 69,86 | SGTASVVCLLNNFYPR |  |
|  |  |  |  |  |  | 938,6497 | 2 | 0,3651 | 75,65 | VYACEVTHQGLSSPVTK |  |
|  |  |  |  |  |  | 955,0535 | 2 | 0,2029 | 138,12 | DIQMTQSPSTLSASVGDR | Oxidation (M) |
|  |  |  |  |  |  | 1002,323 | 2 | 0,5893 | 28,75 | GTVAAPSVFIFPPSDEQLK |  |
|  |  |  |  |  |  | 712,7479 | 3 | 0,2603 | 66,98 | VDNALQSGNSQESVTEQDSK |  |
| 9 | P00738 | Haptoglobin OS=Homo sapiens OX=9606 GN=HP PE=1 SV=1 | 754 | 45861 | 13 | 380,9049 | 2 | 0,4036 | 32,88 | FTDHLK |  |
|  |  |  |  |  |  | 809,4638 | 1 | 0,085 | 31,14 | DYAEVGR |  |
|  |  |  |  |  |  | 429,7944 | 2 | 0,0884 | 48,62 | QLVEIEK |  |
|  |  |  |  |  |  | 460,8407 | 2 | 0,2117 | 48,77 | GSFPWQAK |  |
|  |  |  |  |  |  | 490,8795 | 2 | 0,2569 | 64,76 | VGYVSGWGR |  |
|  |  |  |  |  |  | 530,8049 | 2 | 0,0496 | 27,37 | VMPICLPSK | Oxidation (M) |
|  |  |  |  |  |  | 573,6469 | 2 | -0,256 | 24,07 | HYEGSTVPEK |  |
|  |  |  |  |  |  | 602,3936 | 2 | 0,143 | 61,14 | VTSIQDWVQK |  |
|  |  |  |  |  |  | 646,1267 | 2 | 0,5158 | 54,2 | DIAPTLTLYVGK |  |
|  |  |  |  |  |  | 656,7655 | 2 | 0,9174 | 64,79 | TEGDGVYTLNDK |  |
|  |  |  |  |  |  | 672,8491 | 2 | -0,9547 | 56,31 | SCAVAEYGVYVK |  |
|  |  |  |  |  |  | 720,4502 | 2 | 0,2283 | 66,66 | TEGDGVYTLNNEK |  |
|  |  |  |  |  |  | 862,7205 | 2 | 0,6194 | 68,01 | YVMLPVADQDQCIR | Oxidation (M) |
| 10 | P01859 | Immunoglobulin heavy constant gamma 2 OS=Homo sapiens OX=9606 GN=IGHG2 PE=1 SV=2 | 426 | 36505 | 8 | 412,7406 | 2 | -0,0137 | 20,09 | GLPAPIEK |  |
|  |  |  |  |  |  | 851,4212 | 1 | -0,0079 | 30,6 | DTLMISR | Oxidation (M) |
|  |  |  |  |  |  | 581,3003 | 2 | -0,0362 | 31,87 | NQVSLTCLVK |  |
|  |  |  |  |  |  | 643,9451 | 2 | 0,2089 | 28,61 | EPQVYTLPPSR |  |
|  |  |  |  |  |  | 644,3921 | 2 | 0,1255 | 44,89 | GPSVFPLAPCSR |  |
|  |  |  |  |  |  | 712,2378 | 2 | -0,2413 | 74,08 | STSESTAALGCLVK |  |
|  |  |  |  |  |  | 961,0446 | 2 | -0,807 | 73,86 | TTPPMLDSDGSFFLYSK | Oxidation (M) |
|  |  |  |  |  |  | 961,2441 | 2 | 0,5439 | 20,54 | EPQVYTLPPSREEMTK | Oxidation (M) |
|  |  |  |  |  |  | 961,6111 | 2 | 0,3261 | 65,73 | TTPPMLDSDGSFFLYSK | Oxidation (M) |
| 11 | P01876 | Immunoglobulin heavy constant alpha 1 OS=Homo sapiens OX=9606 GN=IGHA1 PE=1 SV=2 | 413 | 38486 | 6 | 409,8125 | 2 | 0,2134 | 26,18 | VAAEDWK |  |
|  |  |  |  |  |  | 466,6309 | 2 | 0,7086 | 27,49 | TPLTATLSK |  |
|  |  |  |  |  |  | 470,9631 | 2 | 0,4342 | 47,57 | SAVQGPPER |  |
|  |  |  |  |  |  | 607,2776 | 2 | -0,0845 | 54,72 | WLQGSQELPR |  |
|  |  |  |  |  |  | 771,1317 | 2 | 0,5283 | 91,15 | DASGVTFTWTPSSGK |  |
|  |  |  |  |  |  | 918,4977 | 2 | 0,0383 | 36,34 | QEPSQGTTTFAVTSILR |  |
| 12 | P01023 | Alpha-2-macroglobulin OS=Homo sapiens OX=9606 GN=A2M PE=1 SV=3 | 406 | 164613 | 13 | 404,0056 | 2 | 0,5995 | 34,47 | GPTQEFK |  |
|  |  |  |  |  |  | 414,6756 | 2 | -0,1134 | 43,24 | SDIAPVAR |  |
|  |  |  |  |  |  | 509,6854 | 2 | -0,2297 | 50,57 | ATVLNYLPK |  |
|  |  |  |  |  |  | 524,2235 | 2 | 0,8517 | 54,43 | FEVQVTVPK |  |
|  |  |  |  |  |  | 552,3246 | 2 | 0,0364 | 35,34 | SSGSLLNNAIK |  |
|  |  |  |  |  |  | 558,8536 | 2 | 0,0951 | 58,9 | QTVSWAVTPK |  |
|  |  |  |  |  |  | 576,0839 | 2 | 0,5832 | 22,26 | SASNMAIVDVK | Oxidation (M) |
|  |  |  |  |  |  | 606,1802 | 2 | 0,7105 | 63,78 | LPPNVVEESAR |  |
|  |  |  |  |  |  | 628,3235 | 2 | -0,0032 | 49,24 | AIGYLNTGYQR |  |
|  |  |  |  |  |  | 637,0696 | 2 | 0,459 | 53,29 | VTAAPQSVCALR |  |
|  |  |  |  |  |  | 698,213 | 2 | 0,7389 | 83,43 | NEDSLVFVQTDK |  |
|  |  |  |  |  |  | 717,0905 | 2 | 0,3504 | 53,21 | MVSGFIPLKPTVK | Oxidation (M) |
|  |  |  |  |  |  | 942,7462 | 2 | 0,4375 | 64,84 | VSVQLEASPAFLAVPVEK |  |
| 13 | P01860 | Immunoglobulin heavy constant gamma 3 OS=Homo sapiens OX=9606 GN=IGHG3 PE=1 SV=2 | 383 | 42287 | 8 | 420,1064 | 2 | 0,7022 | 35,58 | ALPAPIEK |  |
|  |  |  |  |  |  | 851,4212 | 1 | -0,0079 | 30,6 | DTLMISR | Oxidation (M) |
|  |  |  |  |  |  | 581,3003 | 2 | -0,0362 | 31,87 | NQVSLTCLVK |  |
|  |  |  |  |  |  | 643,9451 | 2 | 0,2089 | 28,61 | EPQVYTLPPSR |  |
|  |  |  |  |  |  | 644,3921 | 2 | 0,1255 | 44,89 | GPSVFPLAPCSR |  |
|  |  |  |  |  |  | 661,317 | 2 | -0,0514 | 84,07 | STSGGTAALGCLVK |  |
|  |  |  |  |  |  | 473,1581 | 3 | 0,769 | 24,41 | WYVDGVEVHNAK |  |
|  |  |  |  |  |  | 961,2441 | 2 | 0,5439 | 20,54 | EPQVYTLPPSREEMTK | Oxidation (M) |
| 14 | P01861 | Immunoglobulin heavy constant gamma 4 OS=Homo sapiens OX=9606 GN=IGHG4 PE=1 SV=1 | 379 | 36431 | 5 | 851,4212 | 1 | -0,0079 | 30,6 | DTLMISR | Oxidation (M) |
|  |  |  |  |  |  | 581,3003 | 2 | -0,0362 | 31,87 | NQVSLTCLVK |  |
|  |  |  |  |  |  | 644,3921 | 2 | 0,1255 | 44,89 | GPSVFPLAPCSR |  |
|  |  |  |  |  |  | 712,2378 | 2 | -0,2413 | 74,08 | STSESTAALGCLVK |  |
|  |  |  |  |  |  | 951,2992 | 2 | -0,3369 | 74,68 | TTPPVLDSDGSFFLYSR |  |
| 15 | P02787 | Serotransferrin OS=Homo sapiens OX=9606 GN=TF PE=1 SV=3 | 378 | 79294 | 7 | 489,7521 | 2 | 0,0079 | 66,56 | DGAGDVAFVK |  |
|  |  |  |  |  |  | 500,6549 | 2 | -0,196 | 23,99 | YLGEEYVK |  |
|  |  |  |  |  |  | 606,326 | 2 | 0,0974 | 52,29 | DSGFQMNQLR | Oxidation (M) |
|  |  |  |  |  |  | 642,5472 | 2 | 0,5181 | 43,97 | EGYYGYTGAFR |  |
|  |  |  |  |  |  | 708,2518 | 2 | -0,2235 | 58,73 | SVIPSDGPSVACVK |  |
|  |  |  |  |  |  | 748,2284 | 2 | 0,7198 | 69,85 | MYLGYEYVTAIR | Oxidation (M) |
|  |  |  |  |  |  | 815,5613 | 2 | 0,2995 | 78,6 | EDPQTFYYAVAVVK |  |
| 16 | P0DOY2 | Immunoglobulin lambda constant 2 OS=Homo sapiens OX=9606 GN=IGLC2 PE=1 SV=1 | 332 | 11458 | 4 | 495,6534 | 2 | -0,2107 | 53,79 | AGVETTTPSK |  |
|  |  |  |  |  |  | 571,5222 | 3 | 0,7929 | 30,65 | SYSCQVTHEGSTVEK |  |
|  |  |  |  |  |  | 872,681 | 2 | 0,496 | 75,35 | YAASSYLSLTPEQWK |  |
|  |  |  |  |  |  | 993,7383 | 2 | 0,4515 | 62,86 | AAPSVTLFPPSSEELQANK |  |
| 17 | Q03591 | Complement factor H-related protein 1 OS=Homo sapiens OX=9606 GN=CFHR1 PE=1 SV=2 | 219 | 38766 | 4 | 564,3469 | 2 | 0,1827 | 44,69 | TGESAEFVCK |  |
|  |  |  |  |  |  | 691,3195 | 2 | -0,0463 | 50,95 | EIMENYNIALR | Oxidation (M) |
|  |  |  |  |  |  | 753,266 | 2 | -0,1694 | 48,2 | ITCTEEGWSPTPK |  |
|  |  |  |  |  |  | 795,5174 | 2 | 0,2939 | 84,36 | LQNNENNISCVER |  |
| 18 | P01042 | Kininogen-1 OS=Homo sapiens OX=9606 GN=KNG1 PE=1 SV=2 | 205 | 72996 | 7 | 502,7208 | 2 | 0,8659 | 58,45 | QVVAGLNFR |  |
|  |  |  |  |  |  | 516,1246 | 2 | 0,7063 | 56,37 | YFIDFVAR |  |
|  |  |  |  |  |  | 579,7086 | 2 | -0,2206 | 30,76 | KYFIDFVAR |  |
|  |  |  |  |  |  | 626,3152 | 2 | 0,034 | 43,78 | TVGSDTFYSFK |  |
|  |  |  |  |  |  | 692,6909 | 2 | 0,7133 | 57,12 | ENFLFLTPDCK |  |
|  |  |  |  |  |  | 938,3026 | 2 | 0,72 | 59,45 | YNSQNQSNNQFVLYR |  |
|  |  |  |  |  |  | 1070,403 | 2 | 0,7166 | 48,26 | DIPTNSPELEETLTHTITK |  |
| 19 | P0DOX8 | Immunoglobulin lambda-1 light chain OS=Homo sapiens OX=9606 PE=1 SV=1 | 201 | 23101 | 4 | 421,3279 | 2 | 0,1344 | 37,21 | VTVLGQPK |  |
|  |  |  |  |  |  | 571,5222 | 3 | 0,7929 | 30,65 | SYSCQVTHEGSTVEK |  |
|  |  |  |  |  |  | 872,681 | 2 | 0,496 | 75,35 | YAASSYLSLTPEQWK |  |
|  |  |  |  |  |  | 1022,004 | 2 | -0,0378 | 22,15 | ANPTVTLFPPSSEELQANK |  |
| 20 | P0C0L4 | Complement C4-A OS=Homo sapiens OX=9606 GN=C4A PE=1 SV=2 | 195 | 194261 | 6 | 485,5738 | 2 | 0,6364 | 39,07 | VEYGFQVK |  |
|  |  |  |  |  |  | 492,4733 | 2 | 0,4699 | 40,05 | QGSFQGGFR |  |
|  |  |  |  |  |  | 518,7096 | 2 | 0,865 | 24,04 | LGQYASPTAK |  |
|  |  |  |  |  |  | 599,2543 | 2 | 0,9318 | 24,33 | ADGSYAAWLSR |  |
|  |  |  |  |  |  | 670,7148 | 2 | 0,7695 | 59,84 | GSFEFPVGDAVSK |  |
|  |  |  |  |  |  | 771,3195 | 2 | -0,1853 | 108,57 | VLSLAQEQVGGSPEK |  |
| 21 | P01011 | Alpha-1-antichymotrypsin OS=Homo sapiens OX=9606 GN=SERPINA3 PE=1 SV=2 | 180 | 47792 | 5 | 487,5779 | 2 | 0,6172 | 40,19 | EQLSLLDR |  |
|  |  |  |  |  |  | 531,3648 | 2 | 0,1346 | 32,69 | EIGELYLPK |  |
|  |  |  |  |  |  | 548,1906 | 2 | 0,7423 | 46,34 | NLAVSQVVHK |  |
|  |  |  |  |  |  | 608,7216 | 2 | 0,7051 | 75,3 | ITLLSALVETR |  |
|  |  |  |  |  |  | 954,7748 | 2 | 0,5826 | 115,65 | AVLDVFEEGTEASAATAVK |  |
| 22 | P0DOX2 | Immunoglobulin alpha-2 heavy chain OS=Homo sapiens OX=9606 PE=1 SV=2 | 168 | 49816 | 5 | 409,8125 | 2 | 0,2134 | 26,18 | VAAEDWK |  |
|  |  |  |  |  |  | 470,9631 | 2 | -0,5498 | 41,48 | SAVEGPPER |  |
|  |  |  |  |  |  | 607,2776 | 2 | -0,0845 | 54,72 | WLQGSQELPR |  |
|  |  |  |  |  |  | 678,0809 | 2 | 0,4762 | 54,77 | NTVYLQMNSLR | Oxidation (M) |
|  |  |  |  |  |  | 926,6844 | 2 | 0,4169 | 75,3 | QEPSQGTTTYAVTSILR |  |
| 23 | P01871 | Immunoglobulin heavy constant mu OS=Homo sapiens OX=9606 GN=IGHM PE=1 SV=4 | 155 | 50093 | 6 | 431,7391 | 2 | -0,0535 | 28,99 | VTSTLTIK |  |
|  |  |  |  |  |  | 515,2426 | 2 | -0,1062 | 44,75 | QIQVSWLR |  |
|  |  |  |  |  |  | 625,4408 | 2 | 0,2386 | 57,95 | LICQATGFSPR |  |
|  |  |  |  |  |  | 639,6987 | 2 | 0,6802 | 76,26 | YAATSQVLLPSK |  |
|  |  |  |  |  |  | 809,1454 | 2 | 0,5099 | 41,34 | YVTSAPMPEPQAPGR | Oxidation (M) |
|  |  |  |  |  |  | 809,8494 | 2 | 0,8836 | 68,26 | QVGSGVTTDQVQAEAK |  |
| 24 | P08603 | Complement factor H OS=Homo sapiens OX=9606 GN=CFH PE=1 SV=4 | 152 | 143680 | 4 | 487,7807 | 2 | 0,1142 | 27,22 | FVCNSGYK |  |
|  |  |  |  |  |  | 578,2854 | 2 | 0,0285 | 56,95 | TGESVEFVCK |  |
|  |  |  |  |  |  | 663,5806 | 2 | 0,5562 | 55,52 | SCDIPVFMNAR | Oxidation (M) |
|  |  |  |  |  |  | 691,3195 | 2 | -0,0463 | 50,95 | EIMENYNIALR | Oxidation (M) |
| 25 | P02747 | Complement C1q subcomponent subunit C OS=Homo sapiens OX=9606 GN=C1QC PE=1 SV=3 | 142 | 25985 | 2 | 629,778 | 2 | 0,8578 | 52,48 | TNQVNSGGVLLR |  |
|  |  |  |  |  |  | 964,3247 | 2 | -0,2611 | 124,17 | FNAVLTNPQGDYDTSTGK |  |
| 26 | P01619 | Immunoglobulin kappa variable 3-20 OS=Homo sapiens OX=9606 GN=IGKV3-20 PE=1 SV=2 | 141 | 12663 | 2 | 490,2242 | 2 | -0,116 | 40,86 | LLIYGASSR |  |
|  |  |  |  |  |  | 817,1569 | 2 | 0,5201 | 95,05 | FSGSGSGTDFTLTISR |  |
| 27 | P02749 | Beta-2-glycoprotein 1 OS=Homo sapiens OX=9606 GN=APOH PE=1 SV=3 | 135 | 39584 | 2 | 511,9864 | 2 | 0,4389 | 71,71 | ATVVYQGER |  |
|  |  |  |  |  |  | 751,8283 | 2 | -0,1292 | 67,06 | VCPFAGILENGAVR |  |
| 28 | P02751 | Fibronectin OS=Homo sapiens OX=9606 GN=FN1 PE=1 SV=5 | 124 | 275742 | 2 | 536,0955 | 2 | -0,3884 | 21,71 | YEVSVYALK |  |
|  |  |  |  |  |  | 731,593 | 2 | 0,388 | 77,76 | VPGTSTSATLTGLTR |  |
| 29 | P02649 | Apolipoprotein E OS=Homo sapiens OX=9606 GN=APOE PE=1 SV=1 | 123 | 36246 | 4 | 474,7222 | 2 | -0,0889 | 59,06 | LAVYQAGAR |  |
|  |  |  |  |  |  | 517,199 | 2 | -0,1518 | 25,41 | LQAEAFQAR |  |
|  |  |  |  |  |  | 624,4138 | 2 | 0,244 | 36,34 | QQTEWQSGQR |  |
|  |  |  |  |  |  | 811,1445 | 2 | 0,4841 | 66,76 | VQAAVGTSAAPVPSDNH |  |
| 30 | P05155 | Plasma protease C1 inhibitor OS=Homo sapiens OX=9606 GN=SERPING1 PE=1 SV=2 | 121 | 55347 | 6 | 455,8244 | 2 | 0,1787 | 24,24 | TLYSSSPR |  |
|  |  |  |  |  |  | 558,6011 | 2 | -0,3947 | 28,1 | LLDSLPSDTR |  |
|  |  |  |  |  |  | 593,6785 | 2 | 0,6508 | 35,36 | FQPTLLTLPR |  |
|  |  |  |  |  |  | 610,1981 | 2 | 0,7954 | 28,47 | DFTCVHQALK |  |
|  |  |  |  |  |  | 632,8314 | 2 | -0,0227 | 44,82 | TNLESILSYPK |  |
|  |  |  |  |  |  | 805,0479 | 2 | -0,6894 | 37,62 | LEDMEQALSPSVFK | Oxidation (M) |
| 31 | P05090 | Apolipoprotein D OS=Homo sapiens OX=9606 GN=APOD PE=1 SV=1 | 108 | 21547 | 4 | 436,4941 | 2 | 0,4813 | 47,82 | VLNQELR |  |
|  |  |  |  |  |  | 616,0652 | 2 | 0,4543 | 44,88 | NILTSNNIDVK |  |
|  |  |  |  |  |  | 712,6334 | 2 | 0,5167 | 49,04 | NPNLPPETVDSLK |  |
|  |  |  |  |  |  | 829,8049 | 2 | 0,8387 | 58,28 | CPNPPVQENFDVNK |  |
| 32 | P02743 | Serum amyloid P-component OS=Homo sapiens OX=9606 GN=APCS PE=1 SV=2 | 105 | 25485 | 2 | 579,0452 | 2 | 0,4834 | 73,66 | VGEYSLYIGR |  |
|  |  |  |  |  |  | 697,7454 | 2 | 0,7877 | 61,63 | IVLGQEQDSYGGK |  |
| 33 | A0A0C4DH25 | Immunoglobulin kappa variable 3D-20 OS=Homo sapiens OX=9606 GN=IGKV3D-20 PE=3 SV=1 | 95 | 12621 | 2 | 518,8142 | 2 | -0,9414 | 20,87 | LLIYDASSR |  |
|  |  |  |  |  |  | 817,1569 | 2 | 0,5201 | 95,05 | FSGSGSGTDFTLTISR |  |
| 34 | P35542 | Serum amyloid A-4 protein OS=Homo sapiens OX=9606 GN=SAA4 PE=1 SV=2 | 91 | 14851 | 3 | 685,392 | 1 | 0,0252 | 20,1 | YLYAR |  |
|  |  |  |  |  |  | 422,0624 | 2 | 0,6656 | 49,53 | GPGGVWAAK |  |
|  |  |  |  |  |  | 574,6688 | 2 | -0,2062 | 56,48 | EALQGVGDMGR | Oxidation (M) |
| 35 | P01019 | Angiotensinogen OS=Homo sapiens OX=9606 GN=AGT PE=1 SV=1 | 84 | 53406 | 3 | 542,5533 | 2 | 0,5701 | 60,36 | FMQAVTGWK | Oxidation (M) |
|  |  |  |  |  |  | 563,1465 | 2 | 0,6031 | 57,31 | LQAILGVPWK |  |
|  |  |  |  |  |  | 635,3822 | 2 | 0,9999 | 22,4 | ALQDQLVLVAAK |  |
| 36 | P03952 | Plasma kallikrein OS=Homo sapiens OX=9606 GN=KLKB1 PE=1 SV=1 | 83 | 73433 | 2 | 546,6816 | 2 | 0,7827 | 25,3 | YSPGGTPTAIK |  |
|  |  |  |  |  |  | 730,7327 | 2 | 0,7406 | 83,39 | IAYGTQGSSGYSLR |  |
| 37 | P19823 | Inter-alpha-trypsin inhibitor heavy chain H2 OS=Homo sapiens OX=9606 GN=ITIH2 PE=1 SV=2 | 76 | 106853 | 3 | 592,7771 | 2 | -0,9735 | 25,7 | SSALDMENFR | Oxidation (M) |
|  |  |  |  |  |  | 792,0778 | 2 | 0,2935 | 31,35 | IQPSGGTNINEALLR |  |
|  |  |  |  |  |  | 1043,991 | 3 | 0,3498 | 25,65 | MATTMIQSKVVNNSPQPQNVVFDVQIPK | Oxidation (M) |
| 38 | P00747 | Plasminogen OS=Homo sapiens OX=9606 GN=PLG PE=1 SV=2 | 67 | 93247 | 2 | 515,9742 | 2 | 0,3632 | 62,63 | LSSPAVITDK |  |
|  |  |  |  |  |  | 570,6111 | 2 | -0,411 | 38,22 | EAQLPVIENK |  |
| 39 | P04004 | Vitronectin OS=Homo sapiens OX=9606 GN=VTN PE=1 SV=1 | 64 | 55069 | 2 | 438,7109 | 2 | 0,9411 | 22,09 | QPQFISR |  |
|  |  |  |  |  |  | 711,9153 | 2 | 0,1697 | 63,82 | FEDGVLDPDYPR |  |
| 40 | P00450 | Ceruloplasmin OS=Homo sapiens OX=9606 GN=CP PE=1 SV=1 | 59 | 122983 | 4 | 604,2684 | 2 | -0,1097 | 43,4 | DIFTGLIGPMK | Oxidation (M) |
|  |  |  |  |  |  | 686,6725 | 2 | 0,5746 | 24,85 | GAYPLSIEPIGVR |  |
|  |  |  |  |  |  | 523,0305 | 3 | 0,3488 | 23,31 | VNKDDEEFIESNK |  |
|  |  |  |  |  |  | 831,0151 | 3 | 0,9696 | 39,34 | MFTTAPDQVDKEDEDFQESNK | Oxidation (M) |
| 41 | P27169 | Serum paraoxonase/arylesterase 1 OS=Homo sapiens OX=9606 GN=PON1 PE=1 SV=3 | 54 | 39877 | 3 | 412,3701 | 2 | 0,277 | 24,6 | STVELFK |  |
|  |  |  |  |  |  | 474,5216 | 2 | 0,5778 | 27,77 | SFNPNSPGK |  |
|  |  |  |  |  |  | 592,6834 | 2 | -0,2927 | 54,19 | IQNILTEEPK |  |
| 42 | P06727 | Apolipoprotein A-IV OS=Homo sapiens OX=9606 GN=APOA4 PE=1 SV=3 | 51 | 45371 | 2 | 429,7944 | 3 | 0,6736 | 29,32 | AKIDQNVEELK |  |
|  |  |  |  |  |  | 704,7438 | 2 | 0,7688 | 51,27 | LGEVNTYAGDLQK |  |

**Table S2.4** PEG-MWCNTs Hard Corona R2

| **Prot. Number** | **Accession Number** | **Protein Name** | **Mascot Score** | **Mr** | **N° pep** | **m/z** | **z** | **Pep. error** | **Pep. Score** | **Pep. sequence** | **Pep. Modification** |
| --- | --- | --- | --- | --- | --- | --- | --- | --- | --- | --- | --- |
| 1 | P02768 | Serum albumin OS=Homo sapiens OX=9606 GN=ALB PE=1 SV=2 | 5458 | 71317 | 33 | 772,3386 | 1 | -0,1 | 20,11 | AACLLPK |  |
|  |  |  |  |  |  | 789,3229 | 1 | -0,1487 | 21,22 | LVTDLTK |  |
|  |  |  |  |  |  | 880,2865 | 1 | -0,1546 | 44,68 | AEFAEVSK |  |
|  |  |  |  |  |  | 464,1144 | 2 | -0,2718 | 34,51 | YLYEIAR |  |
|  |  |  |  |  |  | 467,2481 | 2 | -0,0297 | 39,07 | LCTVATLR |  |
|  |  |  |  |  |  | 470,6563 | 2 | -0,1429 | 20,24 | DDNPNLPR |  |
|  |  |  |  |  |  | 951,2798 | 1 | -0,162 | 39,97 | DLGEENFK |  |
|  |  |  |  |  |  | 480,3486 | 2 | -0,8726 | 26,34 | FQNALLVR |  |
|  |  |  |  |  |  | 984,2394 | 1 | -0,249 | 22,12 | TYETTLEK |  |
|  |  |  |  |  |  | 1000,368 | 1 | -0,2354 | 33,32 | QTALVELVK |  |
|  |  |  |  |  |  | 506,8278 | 2 | -0,9506 | 69,62 | LVAASQAALGL |  |
|  |  |  |  |  |  | 509,2901 | 2 | 0,0365 | 38,52 | SLHTLFGDK |  |
|  |  |  |  |  |  | 528,3942 | 2 | 0,1927 | 38,76 | KYLYEIAR |  |
|  |  |  |  |  |  | 1074,51 | 1 | -0,0328 | 30,88 | LDELRDEGK |  |
|  |  |  |  |  |  | 564,7093 | 2 | -0,2872 | 51,14 | KQTALVELVK |  |
|  |  |  |  |  |  | 569,9869 | 2 | 0,4687 | 44,03 | CCTESLVNR |  |
|  |  |  |  |  |  | 571,5006 | 2 | 0,3 | 71,02 | KLVAASQAALGL |  |
|  |  |  |  |  |  | 575,119 | 2 | -0,3842 | 56,3 | LVNEVTEFAK |  |
|  |  |  |  |  |  | 384,0177 | 3 | 0,4627 | 26,1 | DAHKSEVAHR |  |
|  |  |  |  |  |  | 575,5276 | 2 | 0,4329 | 59,42 | LVNEVTEFAK |  |
|  |  |  |  |  |  | 384,1751 | 3 | 0,9349 | 40,43 | DAHKSEVAHR |  |
|  |  |  |  |  |  | 613,4629 | 2 | -0,6865 | 27,08 | FKDLGEENFK |  |
|  |  |  |  |  |  | 679,3903 | 2 | -0,8563 | 42,82 | AVMDDFAAFVEK | Oxidation (M) |
|  |  |  |  |  |  | 686,1926 | 2 | -0,1888 | 44,73 | AAFTECCQAADK |  |
|  |  |  |  |  |  | 722,1861 | 2 | -0,2771 | 51,87 | YICENQDSISSK |  |
|  |  |  |  |  |  | 726,0501 | 2 | 0,5645 | 47,01 | ETYGEMADCCAK | Oxidation (M) |
|  |  |  |  |  |  | 490,2274 | 3 | 0,8245 | 41,53 | RHPDYSVVLLLR |  |
|  |  |  |  |  |  | 749,9475 | 2 | 0,3093 | 67,6 | TCVADESAENCDK |  |
|  |  |  |  |  |  | 500,7654 | 3 | 0,6498 | 46,86 | ADDKETCFAEEGK |  |
|  |  |  |  |  |  | 756,2843 | 2 | -0,2815 | 52,76 | VPQVSTPTLVEVSR |  |
|  |  |  |  |  |  | 547,1768 | 3 | -0,422 | 84,99 | KVPQVSTPTLVEVSR |  |
|  |  |  |  |  |  | 820,55 | 2 | 0,3102 | 65,57 | DVFLGMFLYEYAR | Oxidation (M) |
|  |  |  |  |  |  | 820,5557 | 2 | 0,1664 | 44,99 | KVPQVSTPTLVEVSR |  |
|  |  |  |  |  |  | 820,5969 | 2 | 0,4041 | 70,79 | DVFLGMFLYEYAR | Oxidation (M) |
|  |  |  |  |  |  | 820,6086 | 2 | 0,2721 | 80,6 | KVPQVSTPTLVEVSR |  |
|  |  |  |  |  |  | 828,9356 | 2 | -0,8888 | 29,76 | QNCELFEQLGEYK |  |
|  |  |  |  |  |  | 633,4451 | 3 | -0,6745 | 22,13 | RHPYFYAPELLFFAK |  |
|  |  |  |  |  |  | 682,3462 | 3 | -0,0713 | 21,26 | VFDEFKPLVEEPQNLIK |  |
| 2 | P01024 | Complement C3 OS=Homo sapiens OX=9606 GN=C3 PE=1 SV=2 | 1005 | 188569 | 18 | 403,5405 | 2 | 0,5919 | 27,03 | TFISPIK |  |
|  |  |  |  |  |  | 436,2935 | 2 | 0,0549 | 39,51 | QGALELIK |  |
|  |  |  |  |  |  | 461,8055 | 2 | -0,9158 | 21,36 | FLTTAKDK |  |
|  |  |  |  |  |  | 542,2058 | 2 | -0,1538 | 53,32 | GYTQQLAFR |  |
|  |  |  |  |  |  | 546,7092 | 2 | -0,2188 | 22,65 | NTLIIYLDK |  |
|  |  |  |  |  |  | 577,0147 | 2 | 0,4174 | 24,11 | QPSSAFAAFVK |  |
|  |  |  |  |  |  | 596,2065 | 2 | 0,7853 | 30,51 | DFDFVPPVVR |  |
|  |  |  |  |  |  | 596,2632 | 2 | -0,0661 | 34,22 | SDDKVTLEER |  |
|  |  |  |  |  |  | 641,2888 | 2 | -0,0254 | 77,3 | ENEGFTVTAEGK |  |
|  |  |  |  |  |  | 645,488 | 2 | 0,3608 | 62,28 | SGSDEVQVGQQR |  |
|  |  |  |  |  |  | 685,6914 | 2 | -0,3559 | 31,17 | TIYTPGSTVLYR |  |
|  |  |  |  |  |  | 701,6484 | 2 | 0,4544 | 23,11 | SSLSVPYVIVPLK |  |
|  |  |  |  |  |  | 756,3619 | 2 | -0,1052 | 85,21 | LVAYYTLIGASGQR |  |
|  |  |  |  |  |  | 829,0449 | 2 | -0,6814 | 61,55 | AGDFLEANYMNLQR | Oxidation (M) |
|  |  |  |  |  |  | 619,3999 | 3 | 0,3296 | 34,76 | SEETKENEGFTVTAEGK |  |
|  |  |  |  |  |  | 1087,173 | 2 | 0,2579 | 109,18 | ILLQGTPVAQMTEDAVDAER | Oxidation (M) |
|  |  |  |  |  |  | 1107,323 | 2 | -0,3657 | 58,23 | EDIPPADLSDQVPDTESETR |  |
|  |  |  |  |  |  | 918,9044 | 3 | -0,5917 | 31,07 | EGVQKEDIPPADLSDQVPDTESETR |  |
| 3 | P01834 | Immunoglobulin kappa constant OS=Homo sapiens OX=9606 GN=IGKC PE=1 SV=2 | 980 | 11929 | 5 | 751,8933 | 2 | 0,0209 | 88,53 | DSTYSLSSTLTLSK |  |
|  |  |  |  |  |  | 938,4102 | 2 | -0,1139 | 67,09 | VYACEVTHQGLSSPVTK |  |
|  |  |  |  |  |  | 973,3578 | 2 | -0,3187 | 91,64 | TVAAPSVFIFPPSDEQLK |  |
|  |  |  |  |  |  | 701,638 | 3 | 0,7714 | 27,67 | RTVAAPSVFIFPPSDEQLK |  |
|  |  |  |  |  |  | 1068,493 | 2 | 0,0108 | 111,06 | VDNALQSGNSQESVTEQDSK |  |
| 4 | P04114 | Apolipoprotein B-100 OS=Homo sapiens OX=9606 GN=APOB PE=1 SV=2 | 789 | 516651 | 31 | 364,2434 | 2 | 0,0447 | 28,45 | LAIPEGK |  |
|  |  |  |  |  |  | 380,4271 | 2 | 0,3858 | 25,51 | ALVDTLK |  |
|  |  |  |  |  |  | 448,6072 | 2 | -0,2224 | 25,18 | EIFNMAR | Oxidation (M) |
|  |  |  |  |  |  | 472,6568 | 2 | -0,2348 | 50,88 | LTLDIQNK |  |
|  |  |  |  |  |  | 480,2808 | 2 | 0,075 | 60,92 | SPSQADINK |  |
|  |  |  |  |  |  | 481,4553 | 2 | 0,3721 | 53,97 | LDVTTSIGR |  |
|  |  |  |  |  |  | 500,2571 | 2 | 0,0253 | 45,87 | MGLAFESTK | Oxidation (M) |
|  |  |  |  |  |  | 506,7164 | 2 | -0,2146 | 68,73 | TGISPLALIK |  |
|  |  |  |  |  |  | 509,1239 | 2 | -0,3533 | 64,54 | LATALSLSNK |  |
|  |  |  |  |  |  | 1039,375 | 1 | -0,2652 | 21,52 | LAPGELTIIL |  |
|  |  |  |  |  |  | 523,5414 | 2 | 0,4716 | 41,64 | IPSVQINFK |  |
|  |  |  |  |  |  | 524,5399 | 2 | 0,5005 | 54,21 | FPEVDVLTK |  |
|  |  |  |  |  |  | 555,725 | 2 | -0,082 | 53,13 | VPQTDMTFR | Oxidation (M) |
|  |  |  |  |  |  | 569,9119 | 2 | 0,2637 | 37,77 | QGFFPDSVNK |  |
|  |  |  |  |  |  | 601,2769 | 2 | -0,1069 | 91,88 | LTISEQNIQR |  |
|  |  |  |  |  |  | 614,5813 | 2 | -0,3709 | 68,32 | NSEEFAAAMSR | Oxidation (M) |
|  |  |  |  |  |  | 618,333 | 2 | 0,0573 | 62,39 | ENFAGEATLQR |  |
|  |  |  |  |  |  | 636,4418 | 2 | 0,1921 | 74 | SVSLPSLDPASAK |  |
|  |  |  |  |  |  | 640,6318 | 2 | -0,4644 | 47,27 | TEVIPPLIENR |  |
|  |  |  |  |  |  | 654,0569 | 2 | 0,4023 | 42,37 | EFQVPTFTIPK |  |
|  |  |  |  |  |  | 655,0438 | 2 | 0,3968 | 48,2 | GFEPTLEALFGK |  |
|  |  |  |  |  |  | 667,7001 | 2 | 0,7335 | 28,51 | VRESDEETQIK |  |
|  |  |  |  |  |  | 669,9564 | 2 | 0,2697 | 48,8 | ESQLPTVMDFR | Oxidation (M) |
|  |  |  |  |  |  | 693,7749 | 2 | -0,2413 | 76,82 | IAELSATAQEIIK |  |
|  |  |  |  |  |  | 716,0582 | 2 | 0,3202 | 72,88 | ALVEQGFTVPEIK |  |
|  |  |  |  |  |  | 753,3796 | 2 | -0,0286 | 55,13 | IGQDGISTSATTNLK |  |
|  |  |  |  |  |  | 808,6453 | 2 | -0,6061 | 63,32 | TSSFALNLPTLPEVK |  |
|  |  |  |  |  |  | 815,8398 | 2 | 0,9259 | 40,36 | MTSNFPVDLSDYPK | Oxidation (M) |
|  |  |  |  |  |  | 829,4597 | 2 | 0,0365 | 99,32 | SVSDGIAALDLNAVANK |  |
|  |  |  |  |  |  | 961,0438 | 2 | 0,1398 | 70,04 | VIGNMGQTMEQLTPELK | 2 Oxidation (M) |
|  |  |  |  |  |  | 981,7396 | 2 | -0,5849 | 40,98 | TILGTMPAFEVSLQALQK | Oxidation (M) |
| 5 | P0DOX7 | Immunoglobulin kappa light chain OS=Homo sapiens OX=9606 PE=1 SV=1 | 733 | 23650 | 5 | 751,8933 | 2 | 0,0209 | 88,53 | DSTYSLSSTLTLSK |  |
|  |  |  |  |  |  | 938,4102 | 2 | -0,1139 | 67,09 | VYACEVTHQGLSSPVTK |  |
|  |  |  |  |  |  | 954,7505 | 2 | -0,4031 | 103,45 | DIQMTQSPSTLSASVGDR | Oxidation (M) |
|  |  |  |  |  |  | 1001,954 | 2 | -0,1487 | 44,16 | GTVAAPSVFIFPPSDEQLK |  |
|  |  |  |  |  |  | 1068,493 | 2 | 0,0108 | 111,06 | VDNALQSGNSQESVTEQDSK |  |
| 6 | P0DOX5 | Immunoglobulin gamma-1 heavy chain OS=Homo sapiens OX=9606 PE=1 SV=2 | 668 | 49925 | 6 | 419,5972 | 2 | -0,3162 | 20,94 | ALPAPIEK |  |
|  |  |  |  |  |  | 581,4827 | 2 | 0,3284 | 44,17 | NQVSLTCLVK |  |
|  |  |  |  |  |  | 593,5472 | 2 | -0,5594 | 34,59 | GPSVFPLAPSSK |  |
|  |  |  |  |  |  | 643,7199 | 2 | -0,2414 | 40,39 | EPQVYTLPPSR |  |
|  |  |  |  |  |  | 661,0167 | 2 | -0,6519 | 72,91 | STSGGTAALGCLVK |  |
|  |  |  |  |  |  | 937,5041 | 2 | 0,0792 | 62,36 | TTPPVLDSDGSFFLYSK |  |
| 7 | P04196 | Histidine-rich glycoprotein OS=Homo sapiens OX=9606 GN=HRG PE=1 SV=1 | 647 | 60510 | 6 | 455,9735 | 2 | 0,4657 | 60,85 | IADAHLDR |  |
|  |  |  |  |  |  | 472,095 | 2 | 0,6097 | 35,19 | ALDLINKR |  |
|  |  |  |  |  |  | 745,6109 | 2 | -0,4765 | 70,29 | GGEGTGYFVDFSVR |  |
|  |  |  |  |  |  | 760,6588 | 2 | -0,3548 | 51,7 | YKEENDDFASFR |  |
|  |  |  |  |  |  | 841,5764 | 2 | -0,6453 | 93,69 | DSPVLIDFFEDTER |  |
|  |  |  |  |  |  | 912,9764 | 2 | 0,0554 | 100,92 | ADLFYDVEALDLESPK |  |
| 8 | P02647 | Apolipoprotein A-I OS=Homo sapiens OX=9606 GN=APOA1 PE=1 SV=1 | 556 | 30759 | 11 | 732,0895 | 1 | -0,2879 | 27,06 | DLEEVK |  |
|  |  |  |  |  |  | 506,8128 | 2 | 0,0398 | 22,31 | AKPALEDLR |  |
|  |  |  |  |  |  | 524,3755 | 2 | 0,2298 | 61,92 | LSPLGEEMR | Oxidation (M) |
|  |  |  |  |  |  | 607,9487 | 2 | -0,7315 | 57,51 | ATEHLSTLSEK |  |
|  |  |  |  |  |  | 615,6576 | 2 | -0,4013 | 52,56 | QGLLPVLESFK |  |
|  |  |  |  |  |  | 1235,417 | 1 | -0,2717 | 41,36 | DLATVYVDVLK |  |
|  |  |  |  |  |  | 626,8304 | 2 | 0,0328 | 33,83 | VQPYLDDFQK |  |
|  |  |  |  |  |  | 651,5575 | 2 | 0,4593 | 36 | THLAPYSDELR |  |
|  |  |  |  |  |  | 693,9042 | 2 | 0,086 | 81,63 | VSFLSALEEYTK |  |
|  |  |  |  |  |  | 700,6804 | 2 | -0,3157 | 109,63 | DYVSQFEGSALGK |  |
|  |  |  |  |  |  | 606,2041 | 3 | 0,747 | 59,69 | DSGRDYVSQFEGSALGK |  |
| 9 | P0C0L4 | Complement C4-A OS=Homo sapiens OX=9606 GN=C4A PE=1 SV=2 | 383 | 194261 | 4 | 518,7404 | 2 | 0,9266 | 24,72 | LGQYASPTAK |  |
|  |  |  |  |  |  | 771,3542 | 2 | -0,1157 | 97,93 | VLSLAQEQVGGSPEK |  |
|  |  |  |  |  |  | 810,4896 | 3 | -0,6712 | 21,58 | AEMADQASAWLTRQGSFQGGFR | Oxidation (M) |
|  |  |  |  |  |  | 1242,321 | 2 | 0,3368 | 111,66 | VTASDPLDTLGSEGALSPGGVASLLR |  |
| 10 | P00738 | Haptoglobin OS=Homo sapiens OX=9606 GN=HP PE=1 SV=1 | 311 | 45861 | 8 | 381,0168 | 2 | 0,6274 | 30,34 | FTDHLK |  |
|  |  |  |  |  |  | 405,1948 | 2 | 0,0036 | 39,42 | DYAEVGR |  |
|  |  |  |  |  |  | 573,8807 | 2 | 0,2116 | 31,33 | HYEGSTVPEK |  |
|  |  |  |  |  |  | 645,6359 | 2 | -0,4659 | 23,12 | DIAPTLTLYVGK |  |
|  |  |  |  |  |  | 673,3481 | 2 | 0,0433 | 80,42 | SCAVAEYGVYVK |  |
|  |  |  |  |  |  | 720,5649 | 2 | 0,4213 | 37,16 | TEGDGVYTLNDKK |  |
|  |  |  |  |  |  | 720,5994 | 2 | 0,5266 | 64,3 | TEGDGVYTLNNEK |  |
|  |  |  |  |  |  | 862,6779 | 2 | 0,5342 | 52,14 | YVMLPVADQDQCIR | Oxidation (M) |
| 11 | P01009 | Alpha-1-antitrypsin OS=Homo sapiens OX=9606 GN=SERPINA1 PE=1 SV=3 | 309 | 46878 | 8 | 398,3869 | 2 | 0,3596 | 27,5 | SPLFMGK | Oxidation (M) |
|  |  |  |  |  |  | 445,0695 | 2 | 0,6281 | 27,65 | AVLTIDEK |  |
|  |  |  |  |  |  | 461,8055 | 2 | 0,1773 | 26,8 | FLENEDR |  |
|  |  |  |  |  |  | 505,0001 | 2 | 0,4933 | 39,43 | QINDYVEK |  |
|  |  |  |  |  |  | 508,2387 | 2 | -0,1444 | 40,56 | SVLGQLGITK |  |
|  |  |  |  |  |  | 555,7912 | 2 | -0,029 | 64,51 | LSITGTYDLK |  |
|  |  |  |  |  |  | 1146,04 | 2 | -0,0579 | 70,75 | GTEAAGAMFLEAIPMSIPPEVK | 2 Oxidation (M) |
|  |  |  |  |  |  | 858,821 | 3 | 0,1076 | 62,09 | TLNQPDSQLQLTTGNGLFLSEGLK |  |
| 12 | P01023 | Alpha-2-macroglobulin OS=Homo sapiens OX=9606 GN=A2M PE=1 SV=3 | 254 | 164613 | 7 | 552,4662 | 2 | 0,3196 | 23,99 | SSGSLLNNAIK |  |
|  |  |  |  |  |  | 575,9602 | 2 | 0,3359 | 58,59 | SASNMAIVDVK | Oxidation (M) |
|  |  |  |  |  |  | 605,8537 | 2 | 0,0575 | 52,76 | LPPNVVEESAR |  |
|  |  |  |  |  |  | 628,5205 | 2 | 0,3908 | 46,72 | AIGYLNTGYQR |  |
|  |  |  |  |  |  | 698,0435 | 2 | 0,3998 | 83,65 | NEDSLVFVQTDK |  |
|  |  |  |  |  |  | 857,5516 | 2 | 0,2595 | 68,35 | SSSNEEVMFLTVQVK | Oxidation (M) |
|  |  |  |  |  |  | 942,4318 | 2 | -0,1914 | 74,89 | VSVQLEASPAFLAVPVEK |  |
| 13 | P27169 | Serum paraoxonase/arylesterase 1 OS=Homo sapiens OX=9606 GN=PON1 PE=1 SV=3 | 247 | 39877 | 4 | 474,3954 | 2 | 0,3255 | 30,81 | SFNPNSPGK |  |
|  |  |  |  |  |  | 592,5513 | 2 | -0,5567 | 40,45 | IQNILTEEPK |  |
|  |  |  |  |  |  | 942,385 | 2 | -0,1546 | 114,46 | IFFYDSENPPASEVLR |  |
|  |  |  |  |  |  | 975,6095 | 2 | 0,2514 | 106,94 | VVAEGFDFANGINISPDGK |  |
| 14 | P0DOY2 | Immunoglobulin lambda constant 2 OS=Homo sapiens OX=9606 GN=IGLC2 PE=1 SV=1 | 201 | 11458 | 2 | 495,7483 | 2 | -0,0209 | 58,96 | AGVETTTPSK |  |
|  |  |  |  |  |  | 993,3245 | 2 | -0,3762 | 26,59 | AAPSVTLFPPSSEELQANK |  |
| 15 | P01011 | Alpha-1-antichymotrypsin OS=Homo sapiens OX=9606 GN=SERPINA3 PE=1 SV=2 | 191 | 47792 | 4 | 487,2956 | 2 | 0,0527 | 32,22 | EQLSLLDR |  |
|  |  |  |  |  |  | 531,4916 | 2 | 0,3882 | 27,04 | EIGELYLPK |  |
|  |  |  |  |  |  | 608,3283 | 2 | -0,0815 | 80,05 | ITLLSALVETR |  |
|  |  |  |  |  |  | 954,5331 | 2 | 0,0992 | 104,97 | AVLDVFEEGTEASAATAVK |  |
| 16 | P01876 | Immunoglobulin heavy constant alpha 1 OS=Homo sapiens OX=9606 GN=IGHA1 PE=1 SV=2 | 179 | 38486 | 4 | 601,4797 | 1 | 0,1129 | 30,74 | DVLVR |  |
|  |  |  |  |  |  | 931,3586 | 1 | -0,1872 | 33,95 | TPLTATLSK |  |
|  |  |  |  |  |  | 470,5313 | 2 | -0,4294 | 44,71 | SAVQGPPER |  |
|  |  |  |  |  |  | 918,3447 | 2 | -0,2676 | 49,56 | QEPSQGTTTFAVTSILR |  |
| 17 | P02747 | Complement C1q subcomponent subunit C OS=Homo sapiens OX=9606 GN=C1QC PE=1 SV=3 | 152 | 25985 | 2 | 629,307 | 2 | -0,0844 | 57,31 | TNQVNSGGVLLR |  |
|  |  |  |  |  |  | 964,6904 | 2 | 0,4704 | 96,12 | FNAVLTNPQGDYDTSTGK |  |
| 18 | P01859 | Immunoglobulin heavy constant gamma 2 OS=Homo sapiens OX=9606 GN=IGHG2 PE=1 SV=2 | 150 | 36505 | 4 | 581,4827 | 2 | 0,3284 | 44,17 | NQVSLTCLVK |  |
|  |  |  |  |  |  | 643,7199 | 2 | -0,2414 | 40,39 | EPQVYTLPPSR |  |
|  |  |  |  |  |  | 712,6018 | 2 | 0,4866 | 72,18 | STSESTAALGCLVK |  |
|  |  |  |  |  |  | 960,977 | 2 | -0,942 | 54,73 | TTPPMLDSDGSFFLYSK | Oxidation (M) |
| 19 | P02649 | Apolipoprotein E OS=Homo sapiens OX=9606 GN=APOE PE=1 SV=1 | 134 | 36246 | 3 | 474,6153 | 2 | -0,3028 | 38,36 | LAVYQAGAR |  |
|  |  |  |  |  |  | 485,0779 | 2 | 0,5961 | 39,18 | LGPLVEQGR |  |
|  |  |  |  |  |  | 811,2017 | 2 | 0,5984 | 88,35 | VQAAVGTSAAPVPSDNH |  |
| 20 | P0DOX8 | Immunoglobulin lambda-1 light chain OS=Homo sapiens OX=9606 PE=1 SV=1 | 131 | 23101 | 2 | 421,404 | 2 | 0,2866 | 46,65 | VTVLGQPK |  |
|  |  |  |  |  |  | 1022,057 | 2 | 0,0677 | 53,44 | ANPTVTLFPPSSEELQANK |  |
| 21 | P01861 | Immunoglobulin heavy constant gamma 4 OS=Homo sapiens OX=9606 GN=IGHG4 PE=1 SV=1 | 118 | 36431 | 3 | 581,4827 | 2 | 0,3284 | 44,17 | NQVSLTCLVK |  |
|  |  |  |  |  |  | 712,6018 | 2 | 0,4866 | 72,18 | STSESTAALGCLVK |  |
|  |  |  |  |  |  | 951,1725 | 2 | -0,5903 | 65,62 | TTPPVLDSDGSFFLYSR |  |
| 22 | P02787 | Serotransferrin OS=Homo sapiens OX=9606 GN=TF PE=1 SV=3 | 108 | 79294 | 3 | 584,1049 | 2 | 0,6112 | 37,18 | HQTVPQNTGGK |  |
|  |  |  |  |  |  | 606,3215 | 2 | 0,0884 | 34,2 | DSGFQMNQLR | Oxidation (M) |
|  |  |  |  |  |  | 815,5921 | 2 | 0,361 | 67,58 | EDPQTFYYAVAVVK |  |
| 23 | Q03591 | Complement factor H-related protein 1 OS=Homo sapiens OX=9606 GN=CFHR1 PE=1 SV=2 | 90 | 38766 | 2 | 564,6262 | 2 | 0,7414 | 56,49 | TGESAEFVCK |  |
|  |  |  |  |  |  | 665,9187 | 2 | 0,1664 | 40,52 | INHGILYDEEK |  |
| 24 | P0DOX2 | Immunoglobulin alpha-2 heavy chain OS=Homo sapiens OX=9606 PE=1 SV=2 | 55 | 49816 | 3 | 601,4797 | 1 | 0,1129 | 30,74 | DVLVR |  |
|  |  |  |  |  |  | 471,1413 | 2 | -0,1933 | 34,05 | SAVEGPPER |  |
|  |  |  |  |  |  | 678,134 | 2 | 0,5824 | 55,25 | NTVYLQMNSLR | Oxidation (M) |
| 25 | P01019 | Angiotensinogen OS=Homo sapiens OX=9606 GN=AGT PE=1 SV=1 | 52 | 53406 | 2 | 635,1459 | 2 | 0,5272 | 55,69 | ALQDQLVLVAAK |  |
|  |  |  |  |  |  | 765,0559 | 2 | -0,6558 | 25,13 | AAMVGMLANFLGFR | 2 Oxidation (M) |

**Table S2.5** PEG-MWCNTs-wtDAAO Soft Corona R1

| **Prot. Number** | **Accession Number** | **Protein Name** | **Mascot Score** | **Mr** | **N° pep** | **m/z** | **z** | **Pep. error** | **Pep. Score** | **Pep. sequence** | **Pep. Modification** |
| --- | --- | --- | --- | --- | --- | --- | --- | --- | --- | --- | --- |
| 1 | P02768 | Serum albumin OS=Homo sapiens OX=9606 GN=ALB PE=1 SV=2 | 11888 | 71317 | 43 | 517,4586 | 1 | 0,1605 | 27,35 | ADLAK |  |
|  |  |  |  |  |  | 673,4231 | 1 | 0,0451 | 26,86 | AWAVAR |  |
|  |  |  |  |  |  | 694,4888 | 1 | -0,8471 | 26,78 | NYAEAK |  |
|  |  |  |  |  |  | 386,6339 | 2 | -0,1781 | 32,46 | AACLLPK |  |
|  |  |  |  |  |  | 395,2823 | 2 | 0,0857 | 21,64 | LVTDLTK |  |
|  |  |  |  |  |  | 880,3147 | 1 | -0,1264 | 48,52 | AEFAEVSK |  |
|  |  |  |  |  |  | 464,1943 | 2 | -0,112 | 43,4 | YLYEIAR |  |
|  |  |  |  |  |  | 933,5263 | 1 | 0,0076 | 22,2 | LCTVATLR |  |
|  |  |  |  |  |  | 470,4431 | 2 | -0,5693 | 38,95 | DDNPNLPR |  |
|  |  |  |  |  |  | 951,3647 | 1 | -0,077 | 44,48 | DLGEENFK |  |
|  |  |  |  |  |  | 480,7407 | 2 | -0,0883 | 44,41 | FQNALLVR |  |
|  |  |  |  |  |  | 984,3562 | 1 | -0,1322 | 24,07 | TYETTLEK |  |
|  |  |  |  |  |  | 1000,486 | 1 | -0,1179 | 26,23 | QTALVELVK |  |
|  |  |  |  |  |  | 506,9946 | 2 | -0,6171 | 39,54 | LVAASQAALGL |  |
|  |  |  |  |  |  | 509,4058 | 2 | 0,2679 | 23,08 | SLHTLFGDK |  |
|  |  |  |  |  |  | 528,4778 | 2 | 0,3599 | 31,82 | KYLYEIAR |  |
|  |  |  |  |  |  | 535,9104 | 2 | 0,3676 | 23,55 | ETCFAEEGK |  |
|  |  |  |  |  |  | 537,8923 | 2 | 0,2348 | 35,64 | LDELRDEGK |  |
|  |  |  |  |  |  | 538,2899 | 2 | 0,0736 | 32,19 | NECFLQHK |  |
|  |  |  |  |  |  | 565,0991 | 2 | 0,4923 | 46,4 | KQTALVELVK |  |
|  |  |  |  |  |  | 569,4104 | 2 | -0,6844 | 25,39 | CCTESLVNR |  |
|  |  |  |  |  |  | 571,2836 | 2 | -0,134 | 76,88 | KLVAASQAALGL |  |
|  |  |  |  |  |  | 574,847 | 2 | -0,9282 | 62,49 | LVNEVTEFAK |  |
|  |  |  |  |  |  | 613,652 | 2 | -0,3085 | 30,64 | FKDLGEENFK |  |
|  |  |  |  |  |  | 653,6427 | 2 | 0,6604 | 35,81 | ECCEKPLLEK |  |
|  |  |  |  |  |  | 656,4036 | 2 | 0,0579 | 43,01 | HPDYSVVLLLR |  |
|  |  |  |  |  |  | 679,4238 | 2 | -0,7893 | 44,56 | AVMDDFAAFVEK | Oxidation (M) |
|  |  |  |  |  |  | 686,004 | 2 | -0,566 | 32,58 | AAFTECCQAADK |  |
|  |  |  |  |  |  | 717,8057 | 2 | 0,0708 | 25,67 | ETYGEMADCCAK |  |
|  |  |  |  |  |  | 722,1967 | 2 | -0,256 | 69,34 | YICENQDSISSK |  |
|  |  |  |  |  |  | 725,7767 | 2 | 0,0177 | 48,73 | ETYGEMADCCAK | Oxidation (M) |
|  |  |  |  |  |  | 489,8335 | 3 | 0,7769 | 23,85 | VTKCCTESLVNR |  |
|  |  |  |  |  |  | 1498,426 | 1 | -0,1527 | 53,14 | TCVADESAENCDK |  |
|  |  |  |  |  |  | 750,3675 | 2 | 0,0959 | 62,3 | ADDKETCFAEEGK |  |
|  |  |  |  |  |  | 756,2905 | 2 | -0,269 | 59,46 | VPQVSTPTLVEVSR |  |
|  |  |  |  |  |  | 518,3414 | 3 | 0,412 | 26,57 | CCAAADPHECYAK |  |
|  |  |  |  |  |  | 820,1451 | 2 | -0,4995 | 41,97 | DVFLGMFLYEYAR | Oxidation (M) |
|  |  |  |  |  |  | 547,2602 | 3 | -0,1717 | 43,78 | KVPQVSTPTLVEVSR |  |
|  |  |  |  |  |  | 820,5505 | 2 | 0,3113 | 73,38 | DVFLGMFLYEYAR | Oxidation (M) |
|  |  |  |  |  |  | 820,5756 | 2 | 0,2061 | 68,24 | KVPQVSTPTLVEVSR |  |
|  |  |  |  |  |  | 820,5992 | 2 | 0,4086 | 53,8 | DVFLGMFLYEYAR | Oxidation (M) |
|  |  |  |  |  |  | 820,6111 | 2 | 0,2771 | 62,87 | KVPQVSTPTLVEVSR |  |
|  |  |  |  |  |  | 820,6414 | 2 | 0,493 | 40,75 | DVFLGMFLYEYAR | Oxidation (M) |
|  |  |  |  |  |  | 820,6461 | 2 | 0,3472 | 47,1 | KVPQVSTPTLVEVSR |  |
|  |  |  |  |  |  | 820,6621 | 2 | 0,5345 | 56,83 | DVFLGMFLYEYAR | Oxidation (M) |
|  |  |  |  |  |  | 547,4522 | 3 | 0,4043 | 33,85 | KVPQVSTPTLVEVSR |  |
|  |  |  |  |  |  | 820,687 | 2 | 0,5843 | 64,31 | DVFLGMFLYEYAR | Oxidation (M) |
|  |  |  |  |  |  | 820,6907 | 2 | 0,4364 | 77,84 | KVPQVSTPTLVEVSR |  |
|  |  |  |  |  |  | 820,798 | 2 | 0,8062 | 44,78 | DVFLGMFLYEYAR | Oxidation (M) |
|  |  |  |  |  |  | 820,8099 | 2 | 0,6748 | 83,6 | KVPQVSTPTLVEVSR |  |
|  |  |  |  |  |  | 828,9712 | 2 | -0,8175 | 37,72 | QNCELFEQLGEYK |  |
|  |  |  |  |  |  | 872,2277 | 2 | 0,554 | 31,43 | HPYFYAPELLFFAK |  |
|  |  |  |  |  |  | 956,014 | 2 | 0,0891 | 40,45 | RPCFSALEVDETYVPK |  |
|  |  |  |  |  |  | 1022,945 | 2 | -0,213 | 75,48 | VFDEFKPLVEEPQNLIK |  |
|  |  |  |  |  |  | 696,1009 | 3 | -0,5494 | 31,83 | VHTECCHGDLLECADDR |  |
|  |  |  |  |  |  | 1496,003 | 2 | 0,6597 | 146,13 | SHCIAEVENDEMPADLPSLAADFVESK | Oxidation (M) |
| 2 | P04114 | Apolipoprotein B-100 OS=Homo sapiens OX=9606 GN=APOB PE=1 SV=2 | 3531 | 516651 | 82 | 384,8705 | 2 | 0,2359 | 27,1 | INPLALK |  |
|  |  |  |  |  |  | 386,0168 | 2 | 0,513 | 30,06 | NLLVALK |  |
|  |  |  |  |  |  | 397,5015 | 2 | 0,5502 | 29,22 | EIQIYK |  |
|  |  |  |  |  |  | 401,5669 | 2 | 0,6397 | 23,13 | FIIPSPK |  |
|  |  |  |  |  |  | 406,0873 | 2 | 0,6477 | 25,24 | AQIPILR |  |
|  |  |  |  |  |  | 419,0411 | 2 | 0,6237 | 32,66 | FLDSNIK |  |
|  |  |  |  |  |  | 423,1608 | 2 | 0,8256 | 69,45 | IGVELTGR |  |
|  |  |  |  |  |  | 431,3412 | 2 | 0,1922 | 29,16 | ITLPDFR |  |
|  |  |  |  |  |  | 451,8892 | 2 | 0,3021 | 20,87 | LNGESNLR |  |
|  |  |  |  |  |  | 462,4193 | 2 | 0,348 | 37,34 | QSFDLSVK |  |
|  |  |  |  |  |  | 463,3268 | 2 | 0,2014 | 60,35 | GMALFGEGK | Oxidation (M) |
|  |  |  |  |  |  | 467,2955 | 2 | 0,095 | 52,75 | IEDGTLASK |  |
|  |  |  |  |  |  | 472,7806 | 2 | 0,0128 | 36,48 | LTLDIQNK |  |
|  |  |  |  |  |  | 475,9796 | 2 | 0,4578 | 31,73 | FVTQAEGAK |  |
|  |  |  |  |  |  | 481,3619 | 2 | 0,1853 | 53,52 | LDVTTSIGR |  |
|  |  |  |  |  |  | 487,5108 | 2 | 0,5194 | 42,06 | QIDDIDVR |  |
|  |  |  |  |  |  | 500,474 | 2 | 0,4592 | 41,58 | MGLAFESTK | Oxidation (M) |
|  |  |  |  |  |  | 506,8641 | 2 | 0,0809 | 58,4 | TGISPLALIK |  |
|  |  |  |  |  |  | 508,3344 | 2 | 0,1157 | 50,61 | VSTAFVYTK |  |
|  |  |  |  |  |  | 511,5615 | 2 | 0,5844 | 43,51 | NNALDFVTK |  |
|  |  |  |  |  |  | 515,1116 | 2 | 0,606 | 58,88 | LSNVLQQVK |  |
|  |  |  |  |  |  | 518,7488 | 2 | -0,0155 | 25,33 | GAYQNNEIK |  |
|  |  |  |  |  |  | 1039,489 | 1 | -0,1505 | 29,2 | LAPGELTIIL |  |
|  |  |  |  |  |  | 523,6329 | 2 | 0,6545 | 28,27 | IPSVQINFK |  |
|  |  |  |  |  |  | 524,6844 | 2 | 0,7894 | 25,77 | FPEVDVLTK |  |
|  |  |  |  |  |  | 528,068 | 2 | 0,6237 | 51,1 | LDNIYSSDK |  |
|  |  |  |  |  |  | 353,7261 | 3 | 0,6261 | 23,51 | VQGVEFSHR |  |
|  |  |  |  |  |  | 536,156 | 2 | 0,6803 | 29,07 | IEIPLPFGGK |  |
|  |  |  |  |  |  | 538,1698 | 2 | 0,713 | 34,15 | NIILPVYDK |  |
|  |  |  |  |  |  | 553,6044 | 2 | 0,6854 | 30,86 | DSYDLHDLK |  |
|  |  |  |  |  |  | 556,1295 | 2 | 0,7268 | 54,12 | VPQTDMTFR | Oxidation (M) |
|  |  |  |  |  |  | 563,3772 | 2 | 0,1499 | 51,01 | LIDVISMYR | Oxidation (M) |
|  |  |  |  |  |  | 570,4019 | 2 | 0,2598 | 41,04 | EVYGFNPEGK |  |
|  |  |  |  |  |  | 576,5589 | 2 | 0,5414 | 51,39 | LDFSSQADLR |  |
|  |  |  |  |  |  | 587,0906 | 2 | 0,5905 | 54,32 | YENYELTLK |  |
|  |  |  |  |  |  | 588,8052 | 2 | 0,0176 | 64,43 | GNVATEISTER |  |
|  |  |  |  |  |  | 396,4569 | 3 | 0,7499 | 21,01 | SNTVASLHTEK |  |
|  |  |  |  |  |  | 598,8472 | 2 | 0,1043 | 51,9 | NMEVSVATTTK | Oxidation (M) |
|  |  |  |  |  |  | 601,3279 | 2 | -0,0049 | 69,33 | LTISEQNIQR |  |
|  |  |  |  |  |  | 608,1786 | 2 | 0,7955 | 41,01 | EELCTMFIR | Oxidation (M) |
|  |  |  |  |  |  | 612,0928 | 2 | 0,572 | 53,55 | YNALDLTNNGK |  |
|  |  |  |  |  |  | 614,971 | 2 | 0,4084 | 73,52 | NSEEFAAAMSR | Oxidation (M) |
|  |  |  |  |  |  | 618,6247 | 2 | 0,6406 | 77,7 | ENFAGEATLQR |  |
|  |  |  |  |  |  | 621,8264 | 2 | 0,0091 | 50,06 | ATGVLYDYVNK |  |
|  |  |  |  |  |  | 635,3253 | 2 | 0,9225 | 30,87 | DLKVEDIPLAR |  |
|  |  |  |  |  |  | 636,3611 | 2 | 0,0309 | 54,96 | SVSLPSLDPASAK |  |
|  |  |  |  |  |  | 640,6801 | 2 | -0,368 | 57,06 | TEVIPPLIENR |  |
|  |  |  |  |  |  | 644,2015 | 2 | 0,6644 | 77,2 | NTLELSNGVIVK |  |
|  |  |  |  |  |  | 654,3158 | 2 | 0,9201 | 52,18 | EFQVPTFTIPK |  |
|  |  |  |  |  |  | 655,7315 | 2 | 0,7558 | 60,95 | EVGTVLSQVYSK |  |
|  |  |  |  |  |  | 661,8524 | 2 | 0,0235 | 50,5 | NPNGYSFSIPVK |  |
|  |  |  |  |  |  | 672,3475 | 2 | 0,0366 | 29,64 | SKPTVSSSMEFK | Oxidation (M) |
|  |  |  |  |  |  | 677,9089 | 2 | 0,1071 | 73,12 | YGMVAQVTQTLK | Oxidation (M) |
|  |  |  |  |  |  | 678,9642 | 2 | 0,1074 | 71,61 | LPYTIITTPPLK |  |
|  |  |  |  |  |  | 680,842 | 2 | 0,0753 | 53,54 | SEYQADYESLR |  |
|  |  |  |  |  |  | 681,0668 | 2 | 0,4196 | 66,6 | INNQLTLDSNTK |  |
|  |  |  |  |  |  | 694,2502 | 2 | 0,7094 | 42,75 | IAELSATAQEIIK |  |
|  |  |  |  |  |  | 700,436 | 2 | 0,0605 | 89,33 | TLADLTLLDSPIK |  |
|  |  |  |  |  |  | 700,4764 | 2 | 0,2968 | 81,31 | AASGTTGTYQEWK |  |
|  |  |  |  |  |  | 700,5895 | 2 | 0,3674 | 20,42 | TLADLTLLDSPIK |  |
|  |  |  |  |  |  | 715,8223 | 2 | -0,1517 | 61,19 | ALVEQGFTVPEIK |  |
|  |  |  |  |  |  | 753,6316 | 2 | 0,4753 | 73,3 | IGQDGISTSATTNLK |  |
|  |  |  |  |  |  | 762,9542 | 2 | 0,2997 | 68,59 | LSNDMMGSYAEMK | 3 Oxidation (M) |
|  |  |  |  |  |  | 779,8839 | 2 | -0,0353 | 108,58 | ITENDIQIALDDAK |  |
|  |  |  |  |  |  | 786,3471 | 2 | 0,8248 | 59,97 | TLQGIPQMIGEVIR | Oxidation (M) |
|  |  |  |  |  |  | 791,4143 | 2 | 0,0271 | 81,61 | AVSMPSFSILGSDVR | Oxidation (M) |
|  |  |  |  |  |  | 796,751 | 2 | 0,6258 | 83,19 | VLLDQLGTTISFER |  |
|  |  |  |  |  |  | 808,1861 | 2 | 0,6399 | 66,01 | MYQMDIQQELQR | 2 Oxidation (M) |
|  |  |  |  |  |  | 809,2477 | 2 | 0,5987 | 65,09 | TSSFALNLPTLPEVK |  |
|  |  |  |  |  |  | 813,1946 | 2 | 0,5933 | 94,42 | YEVDQQIQVLMDK | Oxidation (M) |
|  |  |  |  |  |  | 815,6298 | 2 | 0,5059 | 81,67 | MTSNFPVDLSDYPK | Oxidation (M) |
|  |  |  |  |  |  | 834,2603 | 2 | 0,5871 | 39,57 | GIISALLVPPETEEAK |  |
|  |  |  |  |  |  | 862,8995 | 2 | 0,8541 | 48,6 | IVQILPWEQNEQVK |  |
|  |  |  |  |  |  | 874,1834 | 2 | 0,4533 | 60,61 | IEGNLIFDPNNYLPK |  |
|  |  |  |  |  |  | 874,3896 | 2 | 0,8812 | 59,17 | VNWEEEAASGLLTSLK |  |
|  |  |  |  |  |  | 876,1436 | 2 | 0,4992 | 91,05 | LQDFSDQLSDYYEK |  |
|  |  |  |  |  |  | 944,798 | 2 | 0,6519 | 104,41 | LLLQMDSSATAYGSTVSK | Oxidation (M) |
|  |  |  |  |  |  | 960,7103 | 2 | -0,5271 | 71,56 | VIGNMGQTMEQLTPELK | 2 Oxidation (M) |
|  |  |  |  |  |  | 982,352 | 2 | 0,64 | 95,1 | TILGTMPAFEVSLQALQK | Oxidation (M) |
|  |  |  |  |  |  | 678,6185 | 3 | 0,884 | 57,78 | IHSGSFQSQVELSNDQEK |  |
|  |  |  |  |  |  | 1077,667 | 2 | 0,4002 | 110,59 | YTYNYEAESSSGVPGTADSR |  |
|  |  |  |  |  |  | 761,6534 | 3 | 0,925 | 30,59 | KYTYNYEAESSSGVPGTADSR |  |
|  |  |  |  |  |  | 1318,249 | 2 | 0,389 | 80,69 | GISTSAASPAVGTVGMDMDEDDDFSK | 2 Oxidation (M) |
| 3 | P02787 | Serotransferrin OS=Homo sapiens OX=9606 GN=TF PE=1 SV=3 | 1731 | 79294 | 21 | 368,3739 | 2 | 0,3369 | 45,95 | GDVAFVK |  |
|  |  |  |  |  |  | 414,4322 | 2 | 0,4524 | 23,14 | NPDPWAK |  |
|  |  |  |  |  |  | 461,4705 | 2 | 0,4991 | 46,88 | DDTVCLAK |  |
|  |  |  |  |  |  | 978,5731 | 1 | 0,084 | 35,19 | DGAGDVAFVK |  |
|  |  |  |  |  |  | 499,3204 | 2 | 0,1563 | 59,07 | ASYLDCIR |  |
|  |  |  |  |  |  | 1000,547 | 1 | 0,0482 | 25,18 | YLGEEYVK |  |
|  |  |  |  |  |  | 584,202 | 2 | 0,8055 | 33,96 | HQTVPQNTGGK |  |
|  |  |  |  |  |  | 606,2958 | 2 | 0,0371 | 56,62 | DSGFQMNQLR | Oxidation (M) |
|  |  |  |  |  |  | 625,4034 | 2 | 0,1936 | 62,02 | SASDLTWDNLK |  |
|  |  |  |  |  |  | 642,3765 | 2 | 0,1767 | 46,35 | EGYYGYTGAFR |  |
|  |  |  |  |  |  | 678,0608 | 2 | 0,4837 | 72,37 | DYELLCLDGTR |  |
|  |  |  |  |  |  | 690,0964 | 2 | -0,5132 | 35,27 | CLKDGAGDVAFVK |  |
|  |  |  |  |  |  | 708,3577 | 2 | -0,0118 | 59,55 | SVIPSDGPSVACVK |  |
|  |  |  |  |  |  | 748,1492 | 2 | 0,5614 | 69,44 | MYLGYEYVTAIR | Oxidation (M) |
|  |  |  |  |  |  | 766,0554 | 2 | -0,5844 | 42,56 | CSTSSLLEACTFR |  |
|  |  |  |  |  |  | 789,6287 | 2 | 0,5923 | 80,92 | FDEFFSEGCAPGSK |  |
|  |  |  |  |  |  | 815,0531 | 2 | -0,717 | 89,32 | EDPQTFYYAVAVVK |  |
|  |  |  |  |  |  | 862,3292 | 2 | 0,8904 | 90,61 | LCMGSGLNLCEPNNK | Oxidation (M) |
|  |  |  |  |  |  | 863,3447 | 2 | -0,0848 | 99,34 | IECVSAETTEDCIAK |  |
|  |  |  |  |  |  | 606,9013 | 3 | 0,8848 | 24,08 | EGTCPEAPTDECKPVK |  |
|  |  |  |  |  |  | 1096,166 | 2 | 0,3207 | 92,89 | IMNGEADAMSLDGGFVYIAGK | 2 Oxidation (M) |
| 4 | P01834 | Immunoglobulin kappa constant OS=Homo sapiens OX=9606 GN=IGKC PE=1 SV=2 | 1375 | 11929 | 5 | 751,6001 | 2 | -0,5655 | 67,45 | DSTYSLSSTLTLSK |  |
|  |  |  |  |  |  | 899,828 | 2 | 0,7535 | 73,4 | SGTASVVCLLNNFYPR |  |
|  |  |  |  |  |  | 626,0721 | 3 | 0,2749 | 20,09 | VYACEVTHQGLSSPVTK |  |
|  |  |  |  |  |  | 973,4836 | 2 | -0,067 | 66,42 | TVAAPSVFIFPPSDEQLK |  |
|  |  |  |  |  |  | 1068,341 | 2 | -0,2934 | 132,66 | VDNALQSGNSQESVTEQDSK |  |
| 5 | P0DOX5 | Immunoglobulin gamma-1 heavy chain OS=Homo sapiens OX=9606 PE=1 SV=2 | 1350 | 49925 | 9 | 420,0988 | 2 | 0,687 | 28,81 | ALPAPIEK |  |
|  |  |  |  |  |  | 851,5507 | 1 | 0,1215 | 27,7 | DTLMISR | Oxidation (M) |
|  |  |  |  |  |  | 581,2506 | 2 | -0,1357 | 33,13 | NQVSLTCLVK |  |
|  |  |  |  |  |  | 593,3329 | 2 | -0,9881 | 46,25 | GPSVFPLAPSSK |  |
|  |  |  |  |  |  | 643,7083 | 2 | -0,2647 | 24,32 | EPQVYTLPPSR |  |
|  |  |  |  |  |  | 661,3072 | 2 | -0,0708 | 83,99 | STSGGTAALGCLVK |  |
|  |  |  |  |  |  | 937,1785 | 2 | -0,5722 | 38,99 | TTPPVLDSDGSFFLYSK |  |
|  |  |  |  |  |  | 713,7302 | 3 | 0,1486 | 31,75 | TPEVTCVVVDVSHEDPEVK |  |
|  |  |  |  |  |  | 1272,833 | 2 | 0,5264 | 70,28 | GFYPSDIAVEWESNGQPENNYK |  |
| 6 | P0DOX7 | Immunoglobulin kappa light chain OS=Homo sapiens OX=9606 PE=1 SV=1 | 1322 | 23650 | 7 | 375,1305 | 2 | -0,1437 | 21,8 | VTITCR |  |
|  |  |  |  |  |  | 751,6001 | 2 | -0,5655 | 67,45 | DSTYSLSSTLTLSK |  |
|  |  |  |  |  |  | 899,828 | 2 | 0,7535 | 73,4 | SGTASVVCLLNNFYPR |  |
|  |  |  |  |  |  | 626,0721 | 3 | 0,2749 | 20,09 | VYACEVTHQGLSSPVTK |  |
|  |  |  |  |  |  | 955,1303 | 2 | 0,3565 | 96,68 | DIQMTQSPSTLSASVGDR | Oxidation (M) |
|  |  |  |  |  |  | 1002,22 | 2 | 0,384 | 45,72 | GTVAAPSVFIFPPSDEQLK |  |
|  |  |  |  |  |  | 1068,341 | 2 | -0,2934 | 132,66 | VDNALQSGNSQESVTEQDSK |  |
| 7 | P01009 | Alpha-1-antitrypsin OS=Homo sapiens OX=9606 GN=SERPINA1 PE=1 SV=3 | 1244 | 46878 | 9 | 686,4254 | 1 | -0,0193 | 21,32 | IVDLVK |  |
|  |  |  |  |  |  | 445,046 | 2 | 0,581 | 21,1 | AVLTIDEK |  |
|  |  |  |  |  |  | 461,6838 | 2 | -0,0662 | 34,89 | FLENEDR |  |
|  |  |  |  |  |  | 504,8053 | 2 | 0,1038 | 22,85 | QINDYVEK |  |
|  |  |  |  |  |  | 508,3256 | 2 | 0,0293 | 26,08 | SVLGQLGITK |  |
|  |  |  |  |  |  | 555,415 | 2 | -0,7813 | 53,02 | LSITGTYDLK |  |
|  |  |  |  |  |  | 917,3108 | 2 | -0,3086 | 120,2 | VFSNGADLSGVTEEAPLK |  |
|  |  |  |  |  |  | 631,3774 | 3 | 0,2621 | 21,78 | DTEEEDFHVDQVTTVK |  |
|  |  |  |  |  |  | 1146,166 | 2 | 0,1947 | 72,86 | GTEAAGAMFLEAIPMSIPPEVK | 2 Oxidation (M) |
| 8 | P01024 | Complement C3 OS=Homo sapiens OX=9606 GN=C3 PE=1 SV=2 | 1095 | 188569 | 23 | 403,437 | 2 | 0,385 | 24,7 | TFISPIK |  |
|  |  |  |  |  |  | 417,4172 | 2 | 0,3391 | 38,51 | LPYSVVR |  |
|  |  |  |  |  |  | 531,9404 | 2 | 0,385 | 46,85 | ADIGCTPGSGK |  |
|  |  |  |  |  |  | 542,6179 | 2 | 0,6704 | 62,68 | GYTQQLAFR |  |
|  |  |  |  |  |  | 547,2185 | 2 | 0,7998 | 37,43 | NTLIIYLDK |  |
|  |  |  |  |  |  | 555,8292 | 2 | 0,0245 | 22,04 | VLLDGVQNPR |  |
|  |  |  |  |  |  | 570,6832 | 2 | 0,8183 | 40,74 | FYYIYNEK |  |
|  |  |  |  |  |  | 577,1941 | 2 | 0,7761 | 46,51 | QPSSAFAAFVK |  |
|  |  |  |  |  |  | 613,0017 | 2 | 0,3993 | 41,45 | YYTYLIMNK | Oxidation (M) |
|  |  |  |  |  |  | 634,1649 | 2 | 0,7045 | 54,92 | NTMILEICTR | Oxidation (M) |
|  |  |  |  |  |  | 645,4147 | 2 | 0,2141 | 45,26 | SGSDEVQVGQQR |  |
|  |  |  |  |  |  | 668,7539 | 2 | 0,7698 | 54,39 | APSTWLTAYVVK |  |
|  |  |  |  |  |  | 686,2624 | 2 | 0,7862 | 38,55 | TIYTPGSTVLYR |  |
|  |  |  |  |  |  | 701,5426 | 2 | 0,2428 | 36,53 | SSLSVPYVIVPLK |  |
|  |  |  |  |  |  | 735,605 | 2 | -0,5772 | 36,21 | IPIEDGSGEVVLSR |  |
|  |  |  |  |  |  | 756,4413 | 2 | 0,0536 | 106,37 | LVAYYTLIGASGQR |  |
|  |  |  |  |  |  | 828,5557 | 2 | 0,2368 | 48,82 | TVMVNIENPEGIPVK | Oxidation (M) |
|  |  |  |  |  |  | 829,4309 | 2 | 0,0907 | 69,57 | AGDFLEANYMNLQR | Oxidation (M) |
|  |  |  |  |  |  | 940,2522 | 2 | 0,5236 | 71,28 | EYVLPSFEVIVEPTEK |  |
|  |  |  |  |  |  | 1083,682 | 2 | 0,3544 | 71,65 | AYYENSPQQVFSTEFEVK |  |
|  |  |  |  |  |  | 1087,188 | 2 | 0,2872 | 108,18 | ILLQGTPVAQMTEDAVDAER | Oxidation (M) |
|  |  |  |  |  |  | 1099,784 | 2 | 0,4311 | 103,66 | VPVAVQGEDTVQSLTQGDGVAK |  |
|  |  |  |  |  |  | 919,4269 | 3 | 0,9756 | 51,07 | EGVQKEDIPPADLSDQVPDTESETR |  |
| 9 | P00738 | Haptoglobin OS=Homo sapiens OX=9606 GN=HP PE=1 SV=1 | 1077 | 45861 | 17 | 405,2912 | 2 | 0,1963 | 35,57 | DYAEVGR |  |
|  |  |  |  |  |  | 430,0601 | 2 | 0,6198 | 21,71 | QLVEIEK |  |
|  |  |  |  |  |  | 461,0983 | 2 | 0,7268 | 39,38 | GSFPWQAK |  |
|  |  |  |  |  |  | 462,0166 | 2 | -0,505 | 23,01 | ILGGHLDAK |  |
|  |  |  |  |  |  | 491,0307 | 2 | 0,5592 | 64,89 | VGYVSGWGR |  |
|  |  |  |  |  |  | 531,087 | 2 | 0,6137 | 36,39 | VMPICLPSK | Oxidation (M) |
|  |  |  |  |  |  | 573,8298 | 2 | 0,1098 | 22,39 | HYEGSTVPEK |  |
|  |  |  |  |  |  | 602,3426 | 2 | 0,0411 | 36,18 | VTSIQDWVQK |  |
|  |  |  |  |  |  | 425,7708 | 3 | 0,6603 | 26,41 | HYEGSTVPEKK |  |
|  |  |  |  |  |  | 645,687 | 2 | -0,3636 | 52,73 | DIAPTLTLYVGK |  |
|  |  |  |  |  |  | 656,1968 | 2 | -0,22 | 74,03 | TEGDGVYTLNDK |  |
|  |  |  |  |  |  | 673,3743 | 2 | 0,0956 | 81,86 | SCAVAEYGVYVK |  |
|  |  |  |  |  |  | 463,474 | 3 | 0,7367 | 23,01 | LPECEAVCGKPK |  |
|  |  |  |  |  |  | 720,6077 | 2 | 0,5432 | 57,67 | TEGDGVYTLNNEK |  |
|  |  |  |  |  |  | 720,6157 | 2 | 0,5228 | 66,2 | TEGDGVYTLNDKK |  |
|  |  |  |  |  |  | 720,6298 | 2 | 0,5874 | 52,11 | TEGDGVYTLNNEK |  |
|  |  |  |  |  |  | 570,4772 | 3 | 0,5669 | 42,18 | LRTEGDGVYTLNNEK |  |
|  |  |  |  |  |  | 862,3025 | 2 | -0,2165 | 51,77 | YVMLPVADQDQCIR | Oxidation (M) |
| 10 | P01860 | Immunoglobulin heavy constant gamma 3 OS=Homo sapiens OX=9606 GN=IGHG3 PE=1 SV=2 | 749 | 42287 | 7 | 420,0988 | 2 | 0,687 | 28,81 | ALPAPIEK |  |
|  |  |  |  |  |  | 851,5507 | 1 | 0,1215 | 27,7 | DTLMISR | Oxidation (M) |
|  |  |  |  |  |  | 581,2506 | 2 | -0,1357 | 33,13 | NQVSLTCLVK |  |
|  |  |  |  |  |  | 643,7083 | 2 | -0,2647 | 24,32 | EPQVYTLPPSR |  |
|  |  |  |  |  |  | 644,2031 | 2 | -0,2526 | 27,29 | GPSVFPLAPCSR |  |
|  |  |  |  |  |  | 644,218 | 2 | 0,7548 | 22,87 | EPQVYTLPPSR |  |
|  |  |  |  |  |  | 644,5614 | 2 | 0,4641 | 33,34 | GPSVFPLAPCSR |  |
|  |  |  |  |  |  | 661,3072 | 2 | -0,0708 | 83,99 | STSGGTAALGCLVK |  |
|  |  |  |  |  |  | 641,243 | 3 | 0,7774 | 26,74 | EPQVYTLPPSREEMTK | Oxidation (M) |
| 11 | P01023 | Alpha-2-macroglobulin OS=Homo sapiens OX=9606 GN=A2M PE=1 SV=3 | 699 | 164613 | 30 | 403,9007 | 2 | 0,3898 | 37,55 | GPTQEFK |  |
|  |  |  |  |  |  | 414,7744 | 2 | 0,0842 | 43,06 | SDIAPVAR |  |
|  |  |  |  |  |  | 443,9555 | 2 | 0,4619 | 38,93 | YGAATFTR |  |
|  |  |  |  |  |  | 444,9216 | 2 | -0,6265 | 32,2 | SLNEEAVK |  |
|  |  |  |  |  |  | 463,474 | 2 | 0,3942 | 28,45 | TGTHGLLVK |  |
|  |  |  |  |  |  | 504,0302 | 2 | 0,5831 | 29,72 | FQVDNNNR |  |
|  |  |  |  |  |  | 509,502 | 2 | 0,4392 | 40,18 | SLNEEAVKK |  |
|  |  |  |  |  |  | 509,7713 | 2 | -0,0579 | 45,68 | ATVLNYLPK |  |
|  |  |  |  |  |  | 523,8437 | 2 | 0,092 | 70,91 | FEVQVTVPK |  |
|  |  |  |  |  |  | 552,5649 | 2 | 0,5171 | 34,15 | SSGSLLNNAIK |  |
|  |  |  |  |  |  | 559,1301 | 2 | 0,6482 | 57,14 | QTVSWAVTPK |  |
|  |  |  |  |  |  | 561,1306 | 2 | 0,6179 | 26,65 | SIYKPGQTVK |  |
|  |  |  |  |  |  | 575,9634 | 2 | 0,3422 | 25,59 | SASNMAIVDVK | Oxidation (M) |
|  |  |  |  |  |  | 606,098 | 2 | 0,546 | 52,93 | LPPNVVEESAR |  |
|  |  |  |  |  |  | 613,1604 | 2 | -0,2382 | 64,28 | YDVENCLANK |  |
|  |  |  |  |  |  | 628,5508 | 2 | 0,4513 | 47,42 | AIGYLNTGYQR |  |
|  |  |  |  |  |  | 637,0725 | 2 | 0,4649 | 65,33 | VTAAPQSVCALR |  |
|  |  |  |  |  |  | 638,6072 | 2 | 0,6397 | 58,55 | VGFYESDVMGR | Oxidation (M) |
|  |  |  |  |  |  | 697,8225 | 2 | -0,0422 | 39,8 | NEDSLVFVQTDK |  |
|  |  |  |  |  |  | 710,1583 | 2 | 0,7122 | 58,09 | HYDGSYSTFGER |  |
|  |  |  |  |  |  | 740,8287 | 2 | 0,0168 | 56,87 | DMYSFLEDMGLK | 2 Oxidation (M) |
|  |  |  |  |  |  | 746,6945 | 2 | 0,5862 | 81,68 | NQGNTWLTAFVLK |  |
|  |  |  |  |  |  | 510,8357 | 3 | 0,7945 | 29,25 | TAQEGDHGSHVYTK |  |
|  |  |  |  |  |  | 540,2047 | 3 | 0,7513 | 21,89 | TEVSSNHVLIYLDK |  |
|  |  |  |  |  |  | 922,9658 | 2 | -0,1124 | 66,7 | LLIYAVLPTGDVIGDSAK |  |
|  |  |  |  |  |  | 942,8635 | 2 | 0,6721 | 80,44 | VSVQLEASPAFLAVPVEK |  |
|  |  |  |  |  |  | 1031,324 | 2 | 0,5468 | 81,31 | LLLQQVSLPELPGEYSMK | Oxidation (M) |
|  |  |  |  |  |  | 702,7495 | 3 | 0,3137 | 20,31 | MCPQLQQYEMHGPEGLR | 2 Oxidation (M) |
|  |  |  |  |  |  | 745,6097 | 3 | 0,9386 | 48,11 | KYSDASDCHGEDSQAFCEK |  |
|  |  |  |  |  |  | 796,6514 | 3 | 0,7446 | 40,72 | QQNAQGGFSSTQDTVVALHALSK |  |
| 12 | P01859 | Immunoglobulin heavy constant gamma 2 OS=Homo sapiens OX=9606 GN=IGHG2 PE=1 SV=2 | 553 | 36505 | 7 | 851,5507 | 1 | 0,1215 | 27,7 | DTLMISR | Oxidation (M) |
|  |  |  |  |  |  | 581,2506 | 2 | -0,1357 | 33,13 | NQVSLTCLVK |  |
|  |  |  |  |  |  | 643,7083 | 2 | -0,2647 | 24,32 | EPQVYTLPPSR |  |
|  |  |  |  |  |  | 644,2031 | 2 | -0,2526 | 27,29 | GPSVFPLAPCSR |  |
|  |  |  |  |  |  | 644,218 | 2 | 0,7548 | 22,87 | EPQVYTLPPSR |  |
|  |  |  |  |  |  | 644,5614 | 2 | 0,4641 | 33,34 | GPSVFPLAPCSR |  |
|  |  |  |  |  |  | 712,3235 | 2 | -0,0699 | 100,8 | STSESTAALGCLVK |  |
|  |  |  |  |  |  | 641,243 | 3 | 0,7774 | 26,74 | EPQVYTLPPSREEMTK | Oxidation (M) |
|  |  |  |  |  |  | 961,7534 | 2 | 0,6107 | 57,23 | TTPPMLDSDGSFFLYSK | Oxidation (M) |
| 13 | P01861 | Immunoglobulin heavy constant gamma 4 OS=Homo sapiens OX=9606 GN=IGHG4 PE=1 SV=1 | 514 | 36431 | 6 | 851,5507 | 1 | 0,1215 | 27,7 | DTLMISR | Oxidation (M) |
|  |  |  |  |  |  | 581,2506 | 2 | -0,1357 | 33,13 | NQVSLTCLVK |  |
|  |  |  |  |  |  | 644,2031 | 2 | -0,2526 | 27,29 | GPSVFPLAPCSR |  |
|  |  |  |  |  |  | 712,3235 | 2 | -0,0699 | 100,8 | STSESTAALGCLVK |  |
|  |  |  |  |  |  | 951,3949 | 2 | -0,1455 | 53,26 | TTPPVLDSDGSFFLYSR |  |
|  |  |  |  |  |  | 1272,833 | 2 | 0,5264 | 70,28 | GFYPSDIAVEWESNGQPENNYK |  |
| 14 | P0DOY2 | Immunoglobulin lambda constant 2 OS=Homo sapiens OX=9606 GN=IGLC2 PE=1 SV=1 | 469 | 11458 | 5 | 864,2543 | 1 | -0,1225 | 22,18 | TVAPTECS |  |
|  |  |  |  |  |  | 495,6716 | 2 | -0,1743 | 42,91 | AGVETTTPSK |  |
|  |  |  |  |  |  | 856,4692 | 2 | 0,1719 | 57,96 | SYSCQVTHEGSTVEK |  |
|  |  |  |  |  |  | 872,1476 | 2 | -0,5709 | 65,64 | YAASSYLSLTPEQWK |  |
|  |  |  |  |  |  | 993,155 | 2 | -0,715 | 34,39 | AAPSVTLFPPSSEELQANK |  |
| 15 | P02647 | Apolipoprotein A-I OS=Homo sapiens OX=9606 GN=APOA1 PE=1 SV=1 | 349 | 30759 | 9 | 732,4627 | 1 | 0,0853 | 33,21 | DLEEVK |  |
|  |  |  |  |  |  | 436,9973 | 2 | -0,4552 | 63,66 | AELQEGAR |  |
|  |  |  |  |  |  | 524,149 | 2 | -0,2232 | 25,3 | LSPLGEEMR | Oxidation (M) |
|  |  |  |  |  |  | 608,2915 | 2 | -0,0458 | 34,46 | ATEHLSTLSEK |  |
|  |  |  |  |  |  | 626,6575 | 2 | -0,3132 | 25,89 | VQPYLDDFQK |  |
|  |  |  |  |  |  | 650,6782 | 2 | 0,7818 | 22,34 | WQEEMELYR | Oxidation (M) |
|  |  |  |  |  |  | 693,7838 | 2 | -0,1548 | 56,17 | VSFLSALEEYTK |  |
|  |  |  |  |  |  | 700,9475 | 2 | 0,2185 | 90,18 | DYVSQFEGSALGK |  |
|  |  |  |  |  |  | 807,212 | 2 | 0,6315 | 107,59 | LLDNWDSVTSTFSK |  |
| 16 | P01876 | Immunoglobulin heavy constant alpha 1 OS=Homo sapiens OX=9606 GN=IGHA1 PE=1 SV=2 | 314 | 38486 | 7 | 409,7907 | 2 | 0,1699 | 36,84 | VAAEDWK |  |
|  |  |  |  |  |  | 466,523 | 2 | 0,4929 | 41,95 | TPLTATLSK |  |
|  |  |  |  |  |  | 470,931 | 2 | 0,37 | 37,66 | SAVQGPPER |  |
|  |  |  |  |  |  | 607,5007 | 2 | 0,3618 | 65,74 | WLQGSQELPR |  |
|  |  |  |  |  |  | 688,6388 | 2 | 0,6505 | 54,68 | TFTCTAAYPESK |  |
|  |  |  |  |  |  | 770,9597 | 2 | 0,1843 | 28,89 | DASGVTFTWTPSSGK |  |
|  |  |  |  |  |  | 918,6771 | 2 | 0,3972 | 99,34 | QEPSQGTTTFAVTSILR |  |
| 17 | P00734 | Prothrombin OS=Homo sapiens OX=9606 GN=F2 PE=1 SV=2 | 162 | 71475 | 3 | 503,4299 | 2 | 0,3525 | 40,55 | ETWTANVGK |  |
|  |  |  |  |  |  | 626,3136 | 2 | -0,038 | 79,66 | ETAASLLQAGYK |  |
|  |  |  |  |  |  | 1036,168 | 2 | 0,3531 | 101,38 | SEGSSVNLSPPLEQCVPDR |  |
| 18 | P02743 | Serum amyloid P-component OS=Homo sapiens OX=9606 GN=APCS PE=1 SV=2 | 159 | 25485 | 3 | 497,605 | 2 | 0,6776 | 43,63 | DNELLVYK |  |
|  |  |  |  |  |  | 579,1691 | 2 | 0,7312 | 76,9 | VGEYSLYIGR |  |
|  |  |  |  |  |  | 697,3781 | 2 | 0,053 | 56,27 | IVLGQEQDSYGGK |  |
| 19 | P05155 | Plasma protease C1 inhibitor OS=Homo sapiens OX=9606 GN=SERPING1 PE=1 SV=2 | 153 | 55347 | 5 | 456,0706 | 2 | 0,671 | 42,33 | TLYSSSPR |  |
|  |  |  |  |  |  | 559,0724 | 2 | 0,548 | 35,05 | LLDSLPSDTR |  |
|  |  |  |  |  |  | 593,5208 | 2 | 0,3352 | 26,72 | FQPTLLTLPR |  |
|  |  |  |  |  |  | 633,0338 | 2 | 0,382 | 22,09 | TNLESILSYPK |  |
|  |  |  |  |  |  | 805,6228 | 2 | 0,4605 | 68,52 | LEDMEQALSPSVFK | Oxidation (M) |
| 20 | P01011 | Alpha-1-antichymotrypsin OS=Homo sapiens OX=9606 GN=SERPINA3 PE=1 SV=2 | 153 | 47792 | 3 | 547,7632 | 2 | -0,1126 | 34,19 | NLAVSQVVHK |  |
|  |  |  |  |  |  | 711,6894 | 2 | 0,6888 | 26,23 | DEELSCTVVELK |  |
|  |  |  |  |  |  | 954,7181 | 2 | 0,4694 | 135,18 | AVLDVFEEGTEASAATAVK |  |
| 21 | P0DOX2 | Immunoglobulin alpha-2 heavy chain OS=Homo sapiens OX=9606 PE=1 SV=2 | 140 | 49816 | 5 | 409,7907 | 2 | 0,1699 | 36,84 | VAAEDWK |  |
|  |  |  |  |  |  | 470,931 | 2 | -0,614 | 35,71 | SAVEGPPER |  |
|  |  |  |  |  |  | 607,5007 | 2 | 0,3618 | 65,74 | WLQGSQELPR |  |
|  |  |  |  |  |  | 660,2322 | 2 | 0,8839 | 22,02 | AEDTAVYYCAR |  |
|  |  |  |  |  |  | 678,1786 | 2 | 0,6716 | 50,93 | NTVYLQMNSLR | Oxidation (M) |
| 22 | P02790 | Hemopexin OS=Homo sapiens OX=9606 GN=HPX PE=1 SV=2 | 132 | 52385 | 3 | 571,469 | 2 | 0,3419 | 69,59 | GGYTLVSGYPK |  |
|  |  |  |  |  |  | 611,1536 | 2 | 0,694 | 31,75 | NFPSPVDAAFR |  |
|  |  |  |  |  |  | 748,2476 | 2 | -0,1907 | 23,38 | YYCFQGNQFLR |  |
| 23 | A0A0B4J1X5 | Immunoglobulin heavy variable 3-74 OS=Homo sapiens OX=9606 GN=IGHV3-74 PE=3 SV=1 | 119 | 13002 | 2 | 660,2322 | 2 | 0,8839 | 22,02 | AEDTAVYYCAR |  |
|  |  |  |  |  |  | 684,8964 | 2 | 0,0915 | 53,71 | NTLYLQMNSLR | Oxidation (M) |
| 24 | P00751 | Complement factor B OS=Homo sapiens OX=9606 GN=CFB PE=1 SV=2 | 119 | 86847 | 2 | 638,5771 | 2 | 0,4851 | 63,82 | YGLVTYATYPK |  |
|  |  |  |  |  |  | 958,136 | 2 | 0,4174 | 84,17 | FLCTGGVSPYADPNTCR |  |
| 25 | P01871 | Immunoglobulin heavy constant mu OS=Homo sapiens OX=9606 GN=IGHM PE=1 SV=4 | 112 | 50093 | 5 | 431,803 | 2 | 0,0743 | 32,12 | VTSTLTIK |  |
|  |  |  |  |  |  | 450,7505 | 2 | -0,0364 | 42,85 | VSVFVPPR |  |
|  |  |  |  |  |  | 515,6846 | 2 | 0,7779 | 38,16 | QIQVSWLR |  |
|  |  |  |  |  |  | 694,0764 | 2 | 0,3101 | 22,74 | NVPLPVIAELPPK |  |
|  |  |  |  |  |  | 809,6359 | 2 | 0,4567 | 93,39 | QVGSGVTTDQVQAEAK |  |
| 26 | P10909 | Clusterin OS=Homo sapiens OX=9606 GN=CLU PE=1 SV=1 | 100 | 53031 | 3 | 644,4988 | 2 | -0,6476 | 28,61 | ELDESLQVAER |  |
|  |  |  |  |  |  | 882,0515 | 2 | 0,2682 | 56,03 | EILSVDCSTNNPSQAK |  |
|  |  |  |  |  |  | 772,2594 | 3 | 0,5863 | 26,25 | VTTVASHTSDSDVPSGVTEVVVK |  |
| 27 | P0DP03 | Immunoglobulin heavy variable 3-30-5 OS=Homo sapiens OX=9606 GN=IGHV3-30-5 PE=3 SV=1 | 90 | 13110 | 2 | 646,2557 | 2 | 0,9371 | 31,02 | AEDTAVYYCAK |  |
|  |  |  |  |  |  | 684,8964 | 2 | 0,0915 | 53,71 | NTLYLQMNSLR | Oxidation (M) |
| 28 | P04003 | C4b-binding protein alpha chain OS=Homo sapiens OX=9606 GN=C4BPA PE=1 SV=2 | 85 | 69042 | 3 | 565,2317 | 2 | 0,9055 | 28,53 | YTCLPGYVR |  |
|  |  |  |  |  |  | 625,7293 | 2 | 0,7727 | 61,15 | EDVYVVGTVLR |  |
|  |  |  |  |  |  | 736,4091 | 2 | 0,9947 | 58,84 | LSLEIEQLELQR |  |
| 29 | P02774-2 | Isoform 2 of Vitamin D-binding protein OS=Homo sapiens OX=9606 GN=GC | 84 | 40611 | 2 | 476,1051 | 2 | 0,7418 | 33,66 | LCDNLSTK |  |
|  |  |  |  |  |  | 1047,297 | 2 | 0,7603 | 82,27 | SLGECCDVEDSTTCFNAK |  |
| 30 | P01042 | Kininogen-1 OS=Homo sapiens OX=9606 GN=KNG1 PE=1 SV=2 | 79 | 72996 | 2 | 626,3701 | 2 | 0,1437 | 51,11 | TVGSDTFYSFK |  |
|  |  |  |  |  |  | 938,4379 | 2 | 0,9905 | 55,13 | YNSQNQSNNQFVLYR |  |
| 31 | A0A0C4DH25 | Immunoglobulin kappa variable 3D-20 OS=Homo sapiens OX=9606 GN=IGKV3D-20 PE=3 SV=1 | 70 | 12621 | 2 | 365,3057 | 2 | 0,2151 | 22,6 | ATGIPDR |  |
|  |  |  |  |  |  | 817,2213 | 2 | 0,6488 | 70,07 | FSGSGSGTDFTLTISR |  |
| 32 | P04004 | Vitronectin OS=Homo sapiens OX=9606 GN=VTN PE=1 SV=1 | 65 | 55069 | 2 | 711,6894 | 2 | -0,282 | 58,97 | FEDGVLDPDYPR |  |
|  |  |  |  |  |  | 561,5897 | 3 | -0,0158 | 29,09 | DWHGVPGQVDAAMAGR | Oxidation (M) |
| 33 | P00450 | Ceruloplasmin OS=Homo sapiens OX=9606 GN=CP PE=1 SV=1 | 63 | 122983 | 3 | 602,6311 | 2 | 0,7273 | 39,63 | EYTDASFTNR |  |
|  |  |  |  |  |  | 686,3298 | 2 | -0,1107 | 28,91 | GAYPLSIEPIGVR |  |
|  |  |  |  |  |  | 736,2385 | 2 | 0,7441 | 50,08 | EVGPTNADPVCLAK |  |

**Table S2.6** PEG-MWCNTs-wtDAAO Soft Corona R2

| **Prot. Number** | **Accession Number** | **Protein Name** | **Mascot Score** | **Mr** | **N° pep** | **m/z** | **z** | **Pep. error** | **Pep. Score** | **Pep. sequence** | **Pep. Modification** |
| --- | --- | --- | --- | --- | --- | --- | --- | --- | --- | --- | --- |
| 1 | P02768 | Serum albumin OS=Homo sapiens OX=9606 GN=ALB PE=1 SV=2 | 12080 | 71317 | 46 | 462,8868 | 1 | -0,3643 | 20,51 | ASSAK |  |
|  |  |  |  |  |  | 517,3862 | 1 | 0,0882 | 23,02 | ADLAK |  |
|  |  |  |  |  |  | 673,5184 | 1 | 0,1404 | 21,6 | AWAVAR |  |
|  |  |  |  |  |  | 694,6228 | 1 | -0,713 | 34,3 | NYAEAK |  |
|  |  |  |  |  |  | 354,1356 | 2 | 0,9087 | 24,02 | CASLQK |  |
|  |  |  |  |  |  | 386,655 | 2 | -0,1359 | 34,78 | AACLLPK |  |
|  |  |  |  |  |  | 395,2773 | 2 | 0,0757 | 27,39 | LVTDLTK |  |
|  |  |  |  |  |  | 880,2864 | 1 | -0,1547 | 44,68 | AEFAEVSK |  |
|  |  |  |  |  |  | 464,0628 | 2 | -0,3751 | 40,15 | YLYEIAR |  |
|  |  |  |  |  |  | 467,1965 | 2 | -0,1329 | 25,76 | LCTVATLR |  |
|  |  |  |  |  |  | 470,6732 | 2 | -0,1092 | 39,52 | DDNPNLPR |  |
|  |  |  |  |  |  | 951,3778 | 1 | -0,064 | 21,85 | DLGEENFK |  |
|  |  |  |  |  |  | 960,5249 | 1 | -0,0376 | 24,79 | FQNALLVR |  |
|  |  |  |  |  |  | 984,3661 | 1 | -0,1223 | 20,36 | TYETTLEK |  |
|  |  |  |  |  |  | 1000,53 | 1 | -0,0737 | 29,63 | QTALVELVK |  |
|  |  |  |  |  |  | 507,1015 | 2 | -0,4032 | 42,05 | LVAASQAALGL |  |
|  |  |  |  |  |  | 509,3563 | 2 | 0,1689 | 33,74 | SLHTLFGDK |  |
|  |  |  |  |  |  | 528,6042 | 2 | 0,6129 | 43,65 | KYLYEIAR |  |
|  |  |  |  |  |  | 536,0132 | 2 | 0,5732 | 30,69 | ETCFAEEGK |  |
|  |  |  |  |  |  | 537,795 | 2 | 0,0403 | 33,35 | LDELRDEGK |  |
|  |  |  |  |  |  | 538,4929 | 2 | 0,4796 | 23,33 | NECFLQHK |  |
|  |  |  |  |  |  | 564,8652 | 2 | 0,0245 | 51,67 | KQTALVELVK |  |
|  |  |  |  |  |  | 569,6243 | 2 | -0,2566 | 27,96 | CCTESLVNR |  |
|  |  |  |  |  |  | 571,5107 | 2 | 0,3203 | 39,39 | KLVAASQAALGL |  |
|  |  |  |  |  |  | 574,9448 | 2 | -0,7326 | 74,57 | LVNEVTEFAK |  |
|  |  |  |  |  |  | 400,3024 | 3 | 0,3519 | 24,03 | ETCFAEEGKK |  |
|  |  |  |  |  |  | 613,741 | 2 | -0,1305 | 47,96 | FKDLGEENFK |  |
|  |  |  |  |  |  | 653,4764 | 2 | 0,3279 | 43,14 | ECCEKPLLEK |  |
|  |  |  |  |  |  | 656,6251 | 2 | 0,501 | 27,17 | HPDYSVVLLLR |  |
|  |  |  |  |  |  | 679,3992 | 2 | -0,8385 | 72,25 | AVMDDFAAFVEK | Oxidation (M) |
|  |  |  |  |  |  | 686,1343 | 2 | -0,3055 | 42,04 | AAFTECCQAADK |  |
|  |  |  |  |  |  | 717,3296 | 2 | -0,8815 | 63,91 | ETYGEMADCCAK |  |
|  |  |  |  |  |  | 722,1382 | 2 | -0,3728 | 77,46 | YICENQDSISSK |  |
|  |  |  |  |  |  | 725,7866 | 2 | 0,0375 | 58,07 | ETYGEMADCCAK | Oxidation (M) |
|  |  |  |  |  |  | 1498,349 | 1 | -0,2298 | 67,26 | TCVADESAENCDK |  |
|  |  |  |  |  |  | 750,747 | 2 | 0,8549 | 54,62 | ADDKETCFAEEGK |  |
|  |  |  |  |  |  | 755,9941 | 2 | -0,8618 | 21,56 | VPQVSTPTLVEVSR |  |
|  |  |  |  |  |  | 518,5194 | 3 | 0,9458 | 30,82 | CCAAADPHECYAK |  |
|  |  |  |  |  |  | 820,3085 | 2 | -0,1728 | 54,69 | DVFLGMFLYEYAR | Oxidation (M) |
|  |  |  |  |  |  | 547,2329 | 3 | -0,2536 | 39,59 | KVPQVSTPTLVEVSR |  |
|  |  |  |  |  |  | 820,6371 | 2 | 0,4844 | 75,3 | DVFLGMFLYEYAR | Oxidation (M) |
|  |  |  |  |  |  | 547,4334 | 3 | 0,3477 | 59,97 | KVPQVSTPTLVEVSR |  |
|  |  |  |  |  |  | 820,7227 | 2 | 0,6557 | 70,77 | DVFLGMFLYEYAR | Oxidation (M) |
|  |  |  |  |  |  | 820,738 | 2 | 0,5309 | 83,94 | KVPQVSTPTLVEVSR |  |
|  |  |  |  |  |  | 828,9024 | 2 | -0,9551 | 41,42 | QNCELFEQLGEYK |  |
|  |  |  |  |  |  | 581,8439 | 3 | 0,6232 | 29,48 | HPYFYAPELLFFAK |  |
|  |  |  |  |  |  | 637,4058 | 3 | -0,7289 | 22,28 | RPCFSALEVDETYVPK |  |
|  |  |  |  |  |  | 666,5454 | 3 | 0,6923 | 23,12 | NECFLQHKDDNPNLPR |  |
|  |  |  |  |  |  | 682,3284 | 3 | -0,1248 | 50,27 | VFDEFKPLVEEPQNLIK |  |
|  |  |  |  |  |  | 696,4658 | 3 | 0,5454 | 33,19 | VHTECCHGDLLECADDR |  |
|  |  |  |  |  |  | 1495,873 | 2 | 0,399 | 141,61 | SHCIAEVENDEMPADLPSLAADFVESK | Oxidation (M) |
| 2 | P04114 | Apolipoprotein B-100 OS=Homo sapiens OX=9606 GN=APOB PE=1 SV=2 | 3382 | 516651 | 91 | 630,5476 | 1 | 0,1291 | 22,64 | VIGTLK |  |
|  |  |  |  |  |  | 364,3974 | 2 | 0,3527 | 32,7 | LAIPEGK |  |
|  |  |  |  |  |  | 369,1776 | 2 | 0,8876 | 22,38 | LYSILK |  |
|  |  |  |  |  |  | 371,329 | 2 | 0,1889 | 24,12 | GVISIPR |  |
|  |  |  |  |  |  | 380,1111 | 2 | -0,2462 | 32,4 | ALVDTLK |  |
|  |  |  |  |  |  | 382,9052 | 2 | 0,4094 | 25,6 | INFNEK |  |
|  |  |  |  |  |  | 401,4614 | 2 | 0,5014 | 28,8 | VSSFYAK |  |
|  |  |  |  |  |  | 414,8059 | 2 | 0,1472 | 46,61 | LGNNPVSK |  |
|  |  |  |  |  |  | 414,9581 | 2 | 0,4839 | 25,56 | NYQLYK |  |
|  |  |  |  |  |  | 419,087 | 2 | 0,7156 | 40,01 | FLDSNIK |  |
|  |  |  |  |  |  | 422,7709 | 2 | 0,0458 | 60,31 | IGVELTGR |  |
|  |  |  |  |  |  | 426,0832 | 2 | -0,3506 | 27,3 | LHVAGNLK |  |
|  |  |  |  |  |  | 431,3589 | 2 | 0,2277 | 32,98 | ITLPDFR |  |
|  |  |  |  |  |  | 440,5532 | 2 | 0,617 | 23,07 | LFLEETK |  |
|  |  |  |  |  |  | 451,6872 | 2 | -0,1019 | 42,7 | LNGESNLR |  |
|  |  |  |  |  |  | 457,3341 | 2 | 0,1832 | 21,4 | FFGEGTKK |  |
|  |  |  |  |  |  | 462,1131 | 2 | -0,2643 | 28,05 | QSFDLSVK |  |
|  |  |  |  |  |  | 464,0302 | 2 | -0,4186 | 29,37 | HVAEAICK |  |
|  |  |  |  |  |  | 467,1967 | 2 | -0,1027 | 40,72 | IEDGTLASK |  |
|  |  |  |  |  |  | 473,1332 | 2 | 0,718 | 35,79 | LTLDIQNK |  |
|  |  |  |  |  |  | 481,3338 | 2 | 0,1291 | 62,56 | LDVTTSIGR |  |
|  |  |  |  |  |  | 487,6346 | 2 | 0,8106 | 25,83 | YYELEEK |  |
|  |  |  |  |  |  | 487,7348 | 2 | 0,9674 | 31,43 | QIDDIDVR |  |
|  |  |  |  |  |  | 500,3689 | 2 | 0,2489 | 42,98 | MGLAFESTK | Oxidation (M) |
|  |  |  |  |  |  | 506,9862 | 2 | 0,3249 | 65,35 | TGISPLALIK |  |
|  |  |  |  |  |  | 508,3399 | 2 | 0,1266 | 47,62 | VSTAFVYTK |  |
|  |  |  |  |  |  | 511,3553 | 2 | 0,172 | 46,64 | NNALDFVTK |  |
|  |  |  |  |  |  | 514,8925 | 2 | 0,1678 | 58,93 | LSNVLQQVK |  |
|  |  |  |  |  |  | 518,8172 | 2 | 0,1214 | 33,84 | GAYQNNEIK |  |
|  |  |  |  |  |  | 1039,478 | 1 | -0,1618 | 29,07 | LAPGELTIIL |  |
|  |  |  |  |  |  | 523,3731 | 2 | 0,1349 | 39,55 | IPSVQINFK |  |
|  |  |  |  |  |  | 524,4249 | 2 | 0,2704 | 50,09 | FPEVDVLTK |  |
|  |  |  |  |  |  | 527,8806 | 2 | 0,2489 | 50,23 | LDNIYSSDK |  |
|  |  |  |  |  |  | 353,8072 | 3 | 0,8692 | 31,53 | VQGVEFSHR |  |
|  |  |  |  |  |  | 536,1335 | 2 | 0,6352 | 24,41 | IEIPLPFGGK |  |
|  |  |  |  |  |  | 538,0676 | 2 | 0,5085 | 22,94 | NIILPVYDK |  |
|  |  |  |  |  |  | 553,5823 | 2 | 0,6413 | 31,2 | DSYDLHDLK |  |
|  |  |  |  |  |  | 555,7686 | 2 | 0,0051 | 30,48 | VPQTDMTFR | Oxidation (M) |
|  |  |  |  |  |  | 563,2866 | 2 | -0,0313 | 51,05 | LIDVISMYR | Oxidation (M) |
|  |  |  |  |  |  | 570,1786 | 2 | 0,7973 | 31,32 | QGFFPDSVNK |  |
|  |  |  |  |  |  | 570,4646 | 2 | 0,3852 | 55 | EVYGFNPEGK |  |
|  |  |  |  |  |  | 576,5894 | 2 | 0,6023 | 60,57 | LDFSSQADLR |  |
|  |  |  |  |  |  | 586,9048 | 2 | 0,219 | 41,4 | YENYELTLK |  |
|  |  |  |  |  |  | 588,9061 | 2 | 0,2195 | 61,66 | GNVATEISTER |  |
|  |  |  |  |  |  | 593,9094 | 2 | 0,2053 | 45,29 | SNTVASLHTEK |  |
|  |  |  |  |  |  | 598,8961 | 2 | 0,2021 | 62,12 | NMEVSVATTTK | Oxidation (M) |
|  |  |  |  |  |  | 608,2378 | 2 | 0,9139 | 58,55 | EELCTMFIR | Oxidation (M) |
|  |  |  |  |  |  | 611,8594 | 2 | 0,1053 | 57,01 | YNALDLTNNGK |  |
|  |  |  |  |  |  | 614,9724 | 2 | 0,4113 | 67,7 | NSEEFAAAMSR | Oxidation (M) |
|  |  |  |  |  |  | 621,719 | 2 | -0,2058 | 68,12 | ATGVLYDYVNK |  |
|  |  |  |  |  |  | 622,8886 | 2 | 0,1177 | 59,39 | IDDIWNLEVK |  |
|  |  |  |  |  |  | 635,2306 | 2 | 0,7331 | 51,73 | DLKVEDIPLAR |  |
|  |  |  |  |  |  | 640,9379 | 2 | 0,1476 | 43,33 | TEVIPPLIENR |  |
|  |  |  |  |  |  | 653,7911 | 2 | -0,1292 | 47,46 | EFQVPTFTIPK |  |
|  |  |  |  |  |  | 655,6804 | 2 | 0,6538 | 70,5 | EVGTVLSQVYSK |  |
|  |  |  |  |  |  | 662,1832 | 2 | 0,6853 | 44,67 | NPNGYSFSIPVK |  |
|  |  |  |  |  |  | 666,9304 | 2 | -0,8059 | 51,14 | VRESDEETQIK |  |
|  |  |  |  |  |  | 670,1994 | 2 | 0,7557 | 30,72 | ESQLPTVMDFR | Oxidation (M) |
|  |  |  |  |  |  | 672,1877 | 2 | -0,2829 | 34,54 | SKPTVSSSMEFK | Oxidation (M) |
|  |  |  |  |  |  | 677,8093 | 2 | -0,0923 | 78,84 | YGMVAQVTQTLK | Oxidation (M) |
|  |  |  |  |  |  | 678,9615 | 2 | 0,1021 | 63,6 | LPYTIITTPPLK |  |
|  |  |  |  |  |  | 681,2025 | 2 | 0,6911 | 66,87 | INNQLTLDSNTK |  |
|  |  |  |  |  |  | 694,2172 | 2 | 0,6433 | 65,77 | IAELSATAQEIIK |  |
|  |  |  |  |  |  | 700,3289 | 2 | -0,1537 | 97,3 | TLADLTLLDSPIK |  |
|  |  |  |  |  |  | 700,4503 | 2 | 0,2445 | 67,04 | AASGTTGTYQEWK |  |
|  |  |  |  |  |  | 700,692 | 2 | 0,5724 | 70,35 | TLADLTLLDSPIK |  |
|  |  |  |  |  |  | 727,4022 | 2 | 0,9914 | 34,9 | LNGEIQALELPQK |  |
|  |  |  |  |  |  | 753,4535 | 2 | 0,1191 | 89,73 | IGQDGISTSATTNLK |  |
|  |  |  |  |  |  | 763,1202 | 2 | 0,6317 | 57,6 | LSNDMMGSYAEMK | 3 Oxidation (M) |
|  |  |  |  |  |  | 780,219 | 2 | 0,6348 | 87,3 | ITENDIQIALDDAK |  |
|  |  |  |  |  |  | 791,0888 | 2 | 0,4151 | 72,22 | SGSSTASWIQNVDTK |  |
|  |  |  |  |  |  | 791,6398 | 2 | 0,4783 | 92,68 | AVSMPSFSILGSDVR | Oxidation (M) |
|  |  |  |  |  |  | 808,2413 | 2 | 0,7503 | 58,89 | MYQMDIQQELQR | 2 Oxidation (M) |
|  |  |  |  |  |  | 808,7051 | 2 | -0,4865 | 97,04 | TSSFALNLPTLPEVK |  |
|  |  |  |  |  |  | 813,1753 | 2 | 0,5546 | 96,97 | YEVDQQIQVLMDK | Oxidation (M) |
|  |  |  |  |  |  | 815,5673 | 2 | 0,3809 | 92,52 | MTSNFPVDLSDYPK | Oxidation (M) |
|  |  |  |  |  |  | 829,6729 | 2 | 0,4629 | 86,44 | SVSDGIAALDLNAVANK |  |
|  |  |  |  |  |  | 834,3148 | 2 | 0,6962 | 32,42 | GIISALLVPPETEEAK |  |
|  |  |  |  |  |  | 862,5144 | 2 | 0,0838 | 53,4 | IVQILPWEQNEQVK |  |
|  |  |  |  |  |  | 874,2034 | 2 | 0,4934 | 54,62 | IEGNLIFDPNNYLPK |  |
|  |  |  |  |  |  | 876,132 | 2 | 0,476 | 92,41 | LQDFSDQLSDYYEK |  |
|  |  |  |  |  |  | 888,4009 | 2 | -0,0878 | 86,84 | NLQNNAEWVYQGAIR |  |
|  |  |  |  |  |  | 917,5933 | 2 | 0,2047 | 55,5 | ATFQTPDFIVPLTDLR |  |
|  |  |  |  |  |  | 924,7065 | 2 | 0,5401 | 75 | EYSGTIASEANTYLNSK |  |
|  |  |  |  |  |  | 944,3424 | 2 | -0,2593 | 78,85 | LLLQMDSSATAYGSTVSK | Oxidation (M) |
|  |  |  |  |  |  | 960,9319 | 2 | -0,084 | 56,74 | VIGNMGQTMEQLTPELK | 2 Oxidation (M) |
|  |  |  |  |  |  | 982,2654 | 2 | 0,4666 | 62,14 | TILGTMPAFEVSLQALQK | Oxidation (M) |
|  |  |  |  |  |  | 1077,725 | 2 | 0,5169 | 104,18 | YTYNYEAESSSGVPGTADSR |  |
|  |  |  |  |  |  | 1083,143 | 2 | 0,3371 | 112,59 | TQFNNNEYSQDLDAYNTK |  |
|  |  |  |  |  |  | 761,4603 | 3 | 0,3455 | 52,14 | KYTYNYEAESSSGVPGTADSR |  |
|  |  |  |  |  |  | 1234,073 | 2 | 0,7636 | 54,45 | IADFELPTIIVPEQTIEIPSIK |  |
|  |  |  |  |  |  | 1318,215 | 2 | 0,3202 | 85,49 | GISTSAASPAVGTVGMDMDEDDDFSK | 2 Oxidation (M) |
| 3 | P02787 | Serotransferrin OS=Homo sapiens OX=9606 GN=TF PE=1 SV=3 | 1864 | 79294 | 25 | 368,4208 | 2 | 0,4308 | 57,32 | GDVAFVK |  |
|  |  |  |  |  |  | 414,3268 | 2 | 0,2417 | 25,95 | NPDPWAK |  |
|  |  |  |  |  |  | 438,0656 | 2 | 0,6821 | 38,31 | DSAHGFLK |  |
|  |  |  |  |  |  | 461,5936 | 2 | 0,7454 | 39,43 | DDTVCLAK |  |
|  |  |  |  |  |  | 978,4314 | 1 | -0,0577 | 46,4 | DGAGDVAFVK |  |
|  |  |  |  |  |  | 500,7039 | 2 | -0,0981 | 21,56 | YLGEEYVK |  |
|  |  |  |  |  |  | 584,1707 | 2 | 0,7429 | 31,93 | HQTVPQNTGGK |  |
|  |  |  |  |  |  | 606,3867 | 2 | 0,2187 | 32,33 | DSGFQMNQLR | Oxidation (M) |
|  |  |  |  |  |  | 625,4141 | 2 | 0,215 | 27,79 | SASDLTWDNLK |  |
|  |  |  |  |  |  | 641,1417 | 2 | 0,7186 | 49,83 | CDEWSVNSVGK |  |
|  |  |  |  |  |  | 642,2062 | 2 | -0,164 | 33,43 | EGYYGYTGAFR |  |
|  |  |  |  |  |  | 440,1607 | 3 | 0,8783 | 24,83 | WCAVSEHEATK |  |
|  |  |  |  |  |  | 677,8231 | 2 | 0,0082 | 85,95 | DYELLCLDGTR |  |
|  |  |  |  |  |  | 690,0566 | 2 | -0,5928 | 41,62 | CLKDGAGDVAFVK |  |
|  |  |  |  |  |  | 708,5571 | 2 | 0,3871 | 44,99 | SVIPSDGPSVACVK |  |
|  |  |  |  |  |  | 747,9813 | 2 | 0,2255 | 43,07 | MYLGYEYVTAIR | Oxidation (M) |
|  |  |  |  |  |  | 508,239 | 3 | 0,9659 | 23,24 | LKCDEWSVNSVGK |  |
|  |  |  |  |  |  | 766,8127 | 2 | 0,9301 | 88,89 | CSTSSLLEACTFR |  |
|  |  |  |  |  |  | 770,7434 | 2 | 0,7688 | 66,45 | DQYELLCLDNTR |  |
|  |  |  |  |  |  | 789,3345 | 2 | 0,004 | 98,42 | FDEFFSEGCAPGSK |  |
|  |  |  |  |  |  | 815,4874 | 2 | 0,1516 | 71,78 | EDPQTFYYAVAVVK |  |
|  |  |  |  |  |  | 862,271 | 2 | 0,774 | 80,89 | LCMGSGLNLCEPNNK | Oxidation (M) |
|  |  |  |  |  |  | 863,2859 | 2 | -0,2025 | 55,22 | IECVSAETTEDCIAK |  |
|  |  |  |  |  |  | 606,8513 | 3 | 0,7348 | 36,98 | EGTCPEAPTDECKPVK |  |
|  |  |  |  |  |  | 1096,285 | 2 | 0,5575 | 112,44 | IMNGEADAMSLDGGFVYIAGK | 2 Oxidation (M) |
| 4 | P0DOX5 | Immunoglobulin gamma-1 heavy chain OS=Homo sapiens OX=9606 PE=1 SV=2 | 1253 | 49925 | 8 | 623,2529 | 1 | -0,0982 | 22,12 | FTISR |  |
|  |  |  |  |  |  | 419,811 | 2 | 0,1115 | 21,05 | ALPAPIEK |  |
|  |  |  |  |  |  | 581,3208 | 2 | 0,0047 | 47,46 | NQVSLTCLVK |  |
|  |  |  |  |  |  | 593,7205 | 2 | -0,2129 | 42,49 | GPSVFPLAPSSK |  |
|  |  |  |  |  |  | 643,8583 | 2 | 0,0354 | 40,95 | EPQVYTLPPSR |  |
|  |  |  |  |  |  | 661,3684 | 2 | 0,0515 | 70,28 | STSGGTAALGCLVK |  |
|  |  |  |  |  |  | 937,4716 | 2 | 0,014 | 73,78 | TTPPVLDSDGSFFLYSK |  |
|  |  |  |  |  |  | 713,9197 | 3 | 0,7172 | 33,9 | TPEVTCVVVDVSHEDPEVK |  |
| 5 | P01009 | Alpha-1-antitrypsin OS=Homo sapiens OX=9606 GN=SERPINA1 PE=1 SV=3 | 1199 | 46878 | 8 | 445,0057 | 2 | 0,5005 | 24,92 | AVLTIDEK |  |
|  |  |  |  |  |  | 462,1025 | 2 | 0,7713 | 26,56 | FLENEDR |  |
|  |  |  |  |  |  | 504,7934 | 2 | 0,0799 | 32,33 | QINDYVEK |  |
|  |  |  |  |  |  | 508,2971 | 2 | -0,0277 | 30,51 | SVLGQLGITK |  |
|  |  |  |  |  |  | 555,3559 | 2 | -0,8996 | 28,39 | LSITGTYDLK |  |
|  |  |  |  |  |  | 821,4125 | 2 | -0,0458 | 22,4 | ITPNLAEFAFSLYR |  |
|  |  |  |  |  |  | 917,3892 | 2 | -0,1518 | 118,1 | VFSNGADLSGVTEEAPLK |  |
|  |  |  |  |  |  | 1145,902 | 2 | -0,3336 | 77,2 | GTEAAGAMFLEAIPMSIPPEVK | 2 Oxidation (M) |
| 6 | P01834 | Immunoglobulin kappa constant OS=Homo sapiens OX=9606 GN=IGKC PE=1 SV=2 | 1097 | 11929 | 5 | 752,3405 | 2 | 0,9152 | 43,85 | DSTYSLSSTLTLSK |  |
|  |  |  |  |  |  | 899,6333 | 2 | 0,3641 | 60,52 | SGTASVVCLLNNFYPR |  |
|  |  |  |  |  |  | 626,2667 | 3 | 0,8585 | 39 | VYACEVTHQGLSSPVTK |  |
|  |  |  |  |  |  | 973,5614 | 2 | 0,0886 | 57,01 | TVAAPSVFIFPPSDEQLK |  |
|  |  |  |  |  |  | 1068,279 | 2 | -0,4184 | 129,62 | VDNALQSGNSQESVTEQDSK |  |
| 7 | P00738 | Haptoglobin OS=Homo sapiens OX=9606 GN=HP PE=1 SV=1 | 1000 | 45861 | 17 | 380,9006 | 2 | 0,3952 | 33,22 | FTDHLK |  |
|  |  |  |  |  |  | 404,9047 | 2 | -0,5768 | 37,79 | DYAEVGR |  |
|  |  |  |  |  |  | 429,9488 | 2 | 0,3973 | 21,82 | QLVEIEK |  |
|  |  |  |  |  |  | 460,9913 | 2 | 0,5128 | 46,53 | GSFPWQAK |  |
|  |  |  |  |  |  | 462,1615 | 2 | -0,2152 | 36,98 | ILGGHLDAK |  |
|  |  |  |  |  |  | 491,1639 | 2 | 0,8256 | 64,76 | VGYVSGWGR |  |
|  |  |  |  |  |  | 531,1179 | 2 | 0,6756 | 20,68 | VMPICLPSK | Oxidation (M) |
|  |  |  |  |  |  | 573,5963 | 2 | -0,3573 | 27,76 | HYEGSTVPEK |  |
|  |  |  |  |  |  | 602,5518 | 2 | 0,4594 | 41,78 | VTSIQDWVQK |  |
|  |  |  |  |  |  | 425,6791 | 3 | 0,3853 | 35,67 | HYEGSTVPEKK |  |
|  |  |  |  |  |  | 645,6462 | 2 | -0,4453 | 36,87 | DIAPTLTLYVGK |  |
|  |  |  |  |  |  | 656,583 | 2 | 0,5524 | 68,05 | TEGDGVYTLNDK |  |
|  |  |  |  |  |  | 673,0731 | 2 | -0,5067 | 53,49 | SCAVAEYGVYVK |  |
|  |  |  |  |  |  | 720,6262 | 2 | 0,5802 | 64,57 | TEGDGVYTLNNEK |  |
|  |  |  |  |  |  | 720,7555 | 2 | 0,8024 | 61,64 | TEGDGVYTLNDKK |  |
|  |  |  |  |  |  | 862,355 | 2 | -0,1115 | 82,26 | YVMLPVADQDQCIR | Oxidation (M) |
|  |  |  |  |  |  | 730,1342 | 3 | 0,3354 | 28,18 | SPVGVQPILNEHTFCAGMSK | Oxidation (M) |
| 8 | P0DOX7 | Immunoglobulin kappa light chain OS=Homo sapiens OX=9606 PE=1 SV=1 | 872 | 23650 | 7 | 375,0228 | 2 | -0,3592 | 21,6 | VTITCR |  |
|  |  |  |  |  |  | 752,3405 | 2 | 0,9152 | 43,85 | DSTYSLSSTLTLSK |  |
|  |  |  |  |  |  | 899,6333 | 2 | 0,3641 | 60,52 | SGTASVVCLLNNFYPR |  |
|  |  |  |  |  |  | 626,2667 | 3 | 0,8585 | 39 | VYACEVTHQGLSSPVTK |  |
|  |  |  |  |  |  | 954,8413 | 2 | -0,2214 | 136,79 | DIQMTQSPSTLSASVGDR | Oxidation (M) |
|  |  |  |  |  |  | 1002,253 | 2 | 0,4499 | 40,26 | GTVAAPSVFIFPPSDEQLK |  |
|  |  |  |  |  |  | 1068,279 | 2 | -0,4184 | 129,62 | VDNALQSGNSQESVTEQDSK |  |
| 9 | P01023 | Alpha-2-macroglobulin OS=Homo sapiens OX=9606 GN=A2M PE=1 SV=3 | 764 | 164613 | 25 | 403,9489 | 2 | 0,4863 | 32,24 | GPTQEFK |  |
|  |  |  |  |  |  | 414,979 | 2 | 0,4933 | 56,41 | SDIAPVAR |  |
|  |  |  |  |  |  | 442,5221 | 2 | 0,4758 | 22,66 | DLKPAIVK |  |
|  |  |  |  |  |  | 445,0448 | 2 | -0,3802 | 30,06 | SLNEEAVK |  |
|  |  |  |  |  |  | 509,4352 | 2 | 0,3057 | 49,19 | SLNEEAVKK |  |
|  |  |  |  |  |  | 509,5156 | 2 | -0,5692 | 21,51 | ATVLNYLPK |  |
|  |  |  |  |  |  | 523,8276 | 2 | 0,0599 | 61,75 | FEVQVTVPK |  |
|  |  |  |  |  |  | 552,597 | 2 | 0,5812 | 43,67 | SSGSLLNNAIK |  |
|  |  |  |  |  |  | 559,2334 | 2 | 0,8547 | 65,36 | QTVSWAVTPK |  |
|  |  |  |  |  |  | 575,183 | 2 | 0,7377 | 45,59 | QGIPFFGQVR |  |
|  |  |  |  |  |  | 575,7078 | 2 | -0,1689 | 43,88 | SASNMAIVDVK | Oxidation (M) |
|  |  |  |  |  |  | 605,7876 | 2 | -0,0747 | 57,47 | LPPNVVEESAR |  |
|  |  |  |  |  |  | 613,5575 | 2 | 0,556 | 50,54 | YDVENCLANK |  |
|  |  |  |  |  |  | 628,346 | 2 | 0,0417 | 56,57 | AIGYLNTGYQR |  |
|  |  |  |  |  |  | 638,3711 | 2 | 0,1675 | 66,78 | VGFYESDVMGR | Oxidation (M) |
|  |  |  |  |  |  | 697,7984 | 2 | -0,0903 | 26,23 | NEDSLVFVQTDK |  |
|  |  |  |  |  |  | 473,5463 | 3 | 0,0271 | 23,67 | HYDGSYSTFGER |  |
|  |  |  |  |  |  | 717,2723 | 2 | 0,7142 | 79,1 | MVSGFIPLKPTVK | Oxidation (M) |
|  |  |  |  |  |  | 740,5218 | 2 | -0,5971 | 48,96 | DMYSFLEDMGLK | 2 Oxidation (M) |
|  |  |  |  |  |  | 746,6428 | 2 | 0,4829 | 83,84 | NQGNTWLTAFVLK |  |
|  |  |  |  |  |  | 594,1585 | 3 | 0,4508 | 56,52 | DTVIKPLLVEPEGLEK |  |
|  |  |  |  |  |  | 923,5178 | 2 | 0,9916 | 51,26 | LLIYAVLPTGDVIGDSAK |  |
|  |  |  |  |  |  | 925,3149 | 2 | 0,7423 | 85,33 | QFSFPLSSEPFQGSYK |  |
|  |  |  |  |  |  | 942,4852 | 2 | -0,0845 | 33,01 | VSVQLEASPAFLAVPVEK |  |
|  |  |  |  |  |  | 1030,945 | 2 | -0,2108 | 64,24 | LLLQQVSLPELPGEYSMK | Oxidation (M) |
| 10 | P0DOY2 | Immunoglobulin lambda constant 2 OS=Homo sapiens OX=9606 GN=IGLC2 PE=1 SV=1 | 753 | 11458 | 5 | 864,1694 | 1 | -0,2073 | 28,89 | TVAPTECS |  |
|  |  |  |  |  |  | 495,89 | 2 | 0,2625 | 67,19 | AGVETTTPSK |  |
|  |  |  |  |  |  | 856,8155 | 2 | 0,8645 | 46,24 | SYSCQVTHEGSTVEK |  |
|  |  |  |  |  |  | 872,6925 | 2 | 0,5189 | 82,12 | YAASSYLSLTPEQWK |  |
|  |  |  |  |  |  | 993,2495 | 2 | -0,5261 | 51,2 | AAPSVTLFPPSSEELQANK |  |
| 11 | P01860 | Immunoglobulin heavy constant gamma 3 OS=Homo sapiens OX=9606 GN=IGHG3 PE=1 SV=2 | 746 | 42287 | 6 | 419,811 | 2 | 0,1115 | 21,05 | ALPAPIEK |  |
|  |  |  |  |  |  | 581,3208 | 2 | 0,0047 | 47,46 | NQVSLTCLVK |  |
|  |  |  |  |  |  | 593,8702 | 2 | 0,2352 | 37,89 | SCDTPPPCPR |  |
|  |  |  |  |  |  | 643,8583 | 2 | 0,0354 | 40,95 | EPQVYTLPPSR |  |
|  |  |  |  |  |  | 644,3432 | 2 | 0,0277 | 39,76 | GPSVFPLAPCSR |  |
|  |  |  |  |  |  | 661,3684 | 2 | 0,0515 | 70,28 | STSGGTAALGCLVK |  |
| 12 | P02647 | Apolipoprotein A-I OS=Homo sapiens OX=9606 GN=APOA1 PE=1 SV=1 | 609 | 30759 | 14 | 366,9008 | 2 | 0,417 | 34,31 | DLEEVK |  |
|  |  |  |  |  |  | 391,2753 | 2 | 0,1118 | 29,11 | AHVDALR |  |
|  |  |  |  |  |  | 416,5837 | 2 | 0,7243 | 25,47 | LAEYHAK |  |
|  |  |  |  |  |  | 524,1353 | 2 | -0,2506 | 27,81 | LSPLGEEMR | Oxidation (M) |
|  |  |  |  |  |  | 608,3713 | 2 | 0,1139 | 41,26 | ATEHLSTLSEK |  |
|  |  |  |  |  |  | 616,2098 | 2 | 0,7032 | 40,34 | QGLLPVLESFK |  |
|  |  |  |  |  |  | 618,1609 | 2 | -0,3737 | 44,28 | DLATVYVDVLK |  |
|  |  |  |  |  |  | 627,1682 | 2 | 0,7083 | 33,01 | VQPYLDDFQK |  |
|  |  |  |  |  |  | 650,6759 | 2 | 0,7771 | 40,01 | WQEEMELYR | Oxidation (M) |
|  |  |  |  |  |  | 668,7178 | 2 | 0,7822 | 26,84 | QEMSKDLEEVK |  |
|  |  |  |  |  |  | 691,2369 | 2 | 0,7508 | 33,58 | VQPYLDDFQKK |  |
|  |  |  |  |  |  | 694,0014 | 2 | 0,2804 | 56,24 | VSFLSALEEYTK |  |
|  |  |  |  |  |  | 700,6268 | 2 | -0,423 | 65,15 | DYVSQFEGSALGK |  |
|  |  |  |  |  |  | 807,1975 | 2 | 0,6024 | 89,92 | LLDNWDSVTSTFSK |  |
| 13 | P01024 | Complement C3 OS=Homo sapiens OX=9606 GN=C3 PE=1 SV=2 | 571 | 188569 | 17 | 417,4719 | 2 | 0,4487 | 30,04 | LPYSVVR |  |
|  |  |  |  |  |  | 422,0903 | 2 | 0,6388 | 40,06 | VVLVAVDK |  |
|  |  |  |  |  |  | 542,4573 | 2 | 0,3491 | 52,74 | GYTQQLAFR |  |
|  |  |  |  |  |  | 576,7792 | 2 | -0,0537 | 37,11 | QPSSAFAAFVK |  |
|  |  |  |  |  |  | 595,9797 | 2 | 0,3317 | 46,41 | DFDFVPPVVR |  |
|  |  |  |  |  |  | 397,8707 | 3 | 0,0123 | 39,06 | SDDKVTLEER |  |
|  |  |  |  |  |  | 613,0505 | 2 | 0,4969 | 40,03 | YYTYLIMNK | Oxidation (M) |
|  |  |  |  |  |  | 645,5225 | 2 | 0,4296 | 36,09 | SGSDEVQVGQQR |  |
|  |  |  |  |  |  | 668,2649 | 2 | -0,2082 | 55,86 | APSTWLTAYVVK |  |
|  |  |  |  |  |  | 686,2261 | 2 | 0,7134 | 47,65 | TIYTPGSTVLYR |  |
|  |  |  |  |  |  | 701,5463 | 2 | 0,2501 | 23,72 | SSLSVPYVIVPLK |  |
|  |  |  |  |  |  | 735,7177 | 2 | -0,3518 | 21,33 | IPIEDGSGEVVLSR |  |
|  |  |  |  |  |  | 828,5656 | 2 | 0,2565 | 38,63 | TVMVNIENPEGIPVK | Oxidation (M) |
|  |  |  |  |  |  | 895,6843 | 2 | 0,4977 | 95,23 | DICEEQVNSLPGSITK |  |
|  |  |  |  |  |  | 940,2972 | 2 | 0,6137 | 72,2 | EYVLPSFEVIVEPTEK |  |
|  |  |  |  |  |  | 1087,361 | 2 | 0,6344 | 89,85 | ILLQGTPVAQMTEDAVDAER | Oxidation (M) |
|  |  |  |  |  |  | 1099,772 | 2 | 0,4074 | 97,38 | VPVAVQGEDTVQSLTQGDGVAK |  |
| 14 | P01859 | Immunoglobulin heavy constant gamma 2 OS=Homo sapiens OX=9606 GN=IGHG2 PE=1 SV=2 | 543 | 36505 | 5 | 581,3208 | 2 | 0,0047 | 47,46 | NQVSLTCLVK |  |
|  |  |  |  |  |  | 643,8583 | 2 | 0,0354 | 40,95 | EPQVYTLPPSR |  |
|  |  |  |  |  |  | 644,3432 | 2 | 0,0277 | 39,76 | GPSVFPLAPCSR |  |
|  |  |  |  |  |  | 712,3712 | 2 | 0,0255 | 83,74 | STSESTAALGCLVK |  |
|  |  |  |  |  |  | 961,5033 | 2 | 0,1105 | 41,6 | TTPPMLDSDGSFFLYSK | Oxidation (M) |
| 15 | P01876 | Immunoglobulin heavy constant alpha 1 OS=Homo sapiens OX=9606 GN=IGHA1 PE=1 SV=2 | 506 | 38486 | 7 | 601,5259 | 1 | 0,1591 | 25,11 | DVLVR |  |
|  |  |  |  |  |  | 466,3753 | 2 | 0,1975 | 30,45 | TPLTATLSK |  |
|  |  |  |  |  |  | 470,7144 | 2 | -0,0632 | 41,9 | SAVQGPPER |  |
|  |  |  |  |  |  | 607,4738 | 2 | 0,3078 | 56,35 | WLQGSQELPR |  |
|  |  |  |  |  |  | 688,2507 | 2 | -0,1257 | 48,66 | TFTCTAAYPESK |  |
|  |  |  |  |  |  | 771,1248 | 2 | 0,5144 | 86,46 | DASGVTFTWTPSSGK |  |
|  |  |  |  |  |  | 918,7487 | 2 | 0,5403 | 88,4 | QEPSQGTTTFAVTSILR |  |
| 16 | P01861 | Immunoglobulin heavy constant gamma 4 OS=Homo sapiens OX=9606 GN=IGHG4 PE=1 SV=1 | 491 | 36431 | 4 | 581,3208 | 2 | 0,0047 | 47,46 | NQVSLTCLVK |  |
|  |  |  |  |  |  | 644,3432 | 2 | 0,0277 | 39,76 | GPSVFPLAPCSR |  |
|  |  |  |  |  |  | 712,3712 | 2 | 0,0255 | 83,74 | STSESTAALGCLVK |  |
|  |  |  |  |  |  | 951,5062 | 2 | 0,0772 | 41,4 | TTPPVLDSDGSFFLYSR |  |
| 17 | P0CG04 | Immunoglobulin lambda constant 1 OS=Homo sapiens OX=9606 GN=IGLC1 PE=1 SV=1 | 287 | 11512 | 4 | 864,1694 | 1 | -0,2073 | 28,89 | TVAPTECS |  |
|  |  |  |  |  |  | 856,8155 | 2 | 0,8645 | 46,24 | SYSCQVTHEGSTVEK |  |
|  |  |  |  |  |  | 872,6925 | 2 | 0,5189 | 82,12 | YAASSYLSLTPEQWK |  |
|  |  |  |  |  |  | 1022,308 | 2 | 0,5692 | 61,74 | ANPTVTLFPPSSEELQANK |  |
|  |  |  |  |  |  | 665,671 | 2 | 0,6086 | 73,36 | NLDLDSIIAEVK |  |
| 18 | P00450 | Ceruloplasmin OS=Homo sapiens OX=9606 GN=CP PE=1 SV=1 | 224 | 122983 | 5 | 602,1183 | 2 | -0,2983 | 53,06 | EYTDASFTNR |  |
|  |  |  |  |  |  | 686,6108 | 2 | 0,4513 | 37,72 | GAYPLSIEPIGVR |  |
|  |  |  |  |  |  | 716,697 | 2 | 0,7481 | 51,31 | QSEDSTFYLGER |  |
|  |  |  |  |  |  | 736,1846 | 2 | 0,6362 | 60,49 | EVGPTNADPVCLAK |  |
|  |  |  |  |  |  | 952,7147 | 2 | 0,6017 | 58,43 | NNEGTYYSPNYNPQSR |  |
| 19 | P01871 | Immunoglobulin heavy constant mu OS=Homo sapiens OX=9606 GN=IGHM PE=1 SV=4 | 210 | 50093 | 11 | 388,2435 | 2 | 0,0337 | 20,96 | GFPSVLR |  |
|  |  |  |  |  |  | 432,0132 | 2 | 0,4947 | 38,78 | VTSTLTIK |  |
|  |  |  |  |  |  | 451,0205 | 2 | 0,5036 | 38,7 | VSVFVPPR |  |
|  |  |  |  |  |  | 455,2661 | 2 | 0,1036 | 41,68 | DGFFGNPR |  |
|  |  |  |  |  |  | 625,5986 | 2 | 0,5542 | 64,56 | LICQATGFSPR |  |
|  |  |  |  |  |  | 639,5946 | 2 | 0,472 | 55,26 | YAATSQVLLPSK |  |
|  |  |  |  |  |  | 694,1486 | 2 | 0,4544 | 24,71 | NVPLPVIAELPPK |  |
|  |  |  |  |  |  | 512,0943 | 3 | 0,6011 | 28,15 | DVMQGTDEHVVCK | Oxidation (M) |
|  |  |  |  |  |  | 809,0915 | 2 | 0,4021 | 60,28 | YVTSAPMPEPQAPGR | Oxidation (M) |
|  |  |  |  |  |  | 809,6966 | 2 | 0,578 | 83,27 | QVGSGVTTDQVQAEAK |  |
|  |  |  |  |  |  | 573,1619 | 3 | 0,6087 | 42,25 | FTCTVTHTDLPSPLK |  |
| 20 | P05155 | Plasma protease C1 inhibitor OS=Homo sapiens OX=9606 GN=SERPING1 PE=1 SV=2 | 180 | 55347 | 6 | 455,7802 | 2 | 0,0903 | 36,23 | TLYSSSPR |  |
|  |  |  |  |  |  | 558,5775 | 2 | -0,4418 | 61,14 | LLDSLPSDTR |  |
|  |  |  |  |  |  | 593,4283 | 2 | 0,1504 | 37,06 | FQPTLLTLPR |  |
|  |  |  |  |  |  | 633,3243 | 2 | 0,9631 | 51,82 | TNLESILSYPK |  |
|  |  |  |  |  |  | 758,2395 | 2 | 0,7641 | 50,13 | VTTSQDMLSIMEK | 2 Oxidation (M) |
|  |  |  |  |  |  | 805,1465 | 2 | -0,4921 | 62 | LEDMEQALSPSVFK | Oxidation (M) |
| 21 | P00734 | Prothrombin OS=Homo sapiens OX=9606 GN=F2 PE=1 SV=2 | 170 | 71475 | 3 | 626,6146 | 2 | 0,5641 | 75,25 | ETAASLLQAGYK |  |
|  |  |  |  |  |  | 781,751 | 2 | 0,7665 | 43,2 | TATSEYQTFFNPR |  |
|  |  |  |  |  |  | 1077,836 | 2 | -0,2751 | 101,88 | NPDSSTTGPWCYTTDPTVR |  |
| 22 | P02790 | Hemopexin OS=Homo sapiens OX=9606 GN=HPX PE=1 SV=2 | 160 | 52385 | 3 | 571,5725 | 2 | 0,5489 | 54,42 | GGYTLVSGYPK |  |
|  |  |  |  |  |  | 611,0422 | 2 | 0,4714 | 23,75 | NFPSPVDAAFR |  |
|  |  |  |  |  |  | 748,5486 | 2 | 0,4112 | 23,01 | YYCFQGNQFLR |  |
| 23 | P02743 | Serum amyloid P-component OS=Homo sapiens OX=9606 GN=APCS PE=1 SV=2 | 149 | 25485 | 5 | 497,4928 | 2 | 0,4532 | 47,82 | DNELLVYK |  |
|  |  |  |  |  |  | 579,051 | 2 | 0,4951 | 52,19 | VGEYSLYIGR |  |
|  |  |  |  |  |  | 584,1323 | 2 | 0,6733 | 41,59 | QGYFVEAQPK |  |
|  |  |  |  |  |  | 697,4553 | 2 | 0,2076 | 28,59 | IVLGQEQDSYGGK |  |
|  |  |  |  |  |  | 703,3942 | 2 | -0,8887 | 25,93 | AYSLFSYNTQGR |  |
| 24 | P0DOX2 | Immunoglobulin alpha-2 heavy chain OS=Homo sapiens OX=9606 PE=1 SV=2 | 108 | 49816 | 6 | 601,5259 | 1 | 0,1591 | 25,11 | DVLVR |  |
|  |  |  |  |  |  | 623,2529 | 1 | -0,0982 | 22,12 | FTISR |  |
|  |  |  |  |  |  | 470,7646 | 2 | -0,9467 | 23 | SAVEGPPER |  |
|  |  |  |  |  |  | 607,4738 | 2 | 0,3078 | 56,35 | WLQGSQELPR |  |
|  |  |  |  |  |  | 659,574 | 2 | -0,4324 | 66,49 | AEDTAVYYCAR |  |
|  |  |  |  |  |  | 678,1908 | 2 | 0,696 | 40,93 | NTVYLQMNSLR | Oxidation (M) |
| 25 | P08519 | Apolipoprotein(a) OS=Homo sapiens OX=9606 GN=LPA PE=1 SV=1 | 108 | 514737 | 4 | 506,842 | 2 | 0,1709 | 20,11 | GSFSTTVTGR |  |
|  |  |  |  |  |  | 666,6709 | 2 | -0,3197 | 35,31 | TPENYPNAGLTR |  |
|  |  |  |  |  |  | 749,7548 | 2 | 0,8232 | 52,6 | NPDAVAAPYCYTR |  |
|  |  |  |  |  |  | 989,6285 | 2 | 0,3665 | 73,2 | TPEYYPNAGLIMNYCR | Oxidation (M) |
| 26 | P02774-2 | Isoform 2 of Vitamin D-binding protein OS=Homo sapiens OX=9606 GN=GC | 104 | 40611 | 3 | 475,6663 | 2 | -0,1358 | 32,73 | LCDNLSTK |  |
|  |  |  |  |  |  | 578,3829 | 2 | 0,133 | 48,59 | LPDATPTELAK |  |
|  |  |  |  |  |  | 1047,256 | 2 | 0,6795 | 77,78 | SLGECCDVEDSTTCFNAK |  |
| 27 | A0A0B4J1X5 | Immunoglobulin heavy variable 3-74 OS=Homo sapiens OX=9606 GN=IGHV3-74 PE=3 SV=1 | 101 | 13002 | 3 | 623,2529 | 1 | -0,0982 | 22,12 | FTISR |  |
|  |  |  |  |  |  | 659,574 | 2 | -0,4324 | 66,49 | AEDTAVYYCAR |  |
|  |  |  |  |  |  | 685,1898 | 2 | 0,6784 | 38,44 | NTLYLQMNSLR | Oxidation (M) |
| 28 | P01042 | Kininogen-1 OS=Homo sapiens OX=9606 GN=KNG1 PE=1 SV=2 | 99 | 72996 | 2 | 626,5972 | 2 | 0,5978 | 55,96 | TVGSDTFYSFK |  |
|  |  |  |  |  |  | 938,3782 | 2 | 0,8711 | 69,43 | YNSQNQSNNQFVLYR |  |
| 29 | P00751 | Complement factor B OS=Homo sapiens OX=9606 GN=CFB PE=1 SV=2 | 92 | 86847 | 3 | 1050,293 | 1 | 0,7778 | 24,81 | LQDEDLGFL |  |
|  |  |  |  |  |  | 922,2706 | 2 | 0,618 | 29,9 | EAGIPEFYDYDVALIK |  |
|  |  |  |  |  |  | 958,1246 | 2 | 0,3947 | 88,83 | FLCTGGVSPYADPNTCR |  |
| 30 | P02751 | Fibronectin OS=Homo sapiens OX=9606 GN=FN1 PE=1 SV=5 | 91 | 275742 | 2 | 731,7468 | 2 | 0,6956 | 90,52 | VPGTSTSATLTGLTR |  |
|  |  |  |  |  |  | 602,0884 | 3 | -0,7046 | 24,14 | GATYNVIVEALKDQQR |  |
| 31 | P01011 | Alpha-1-antichymotrypsin OS=Homo sapiens OX=9606 GN=SERPINA3 PE=1 SV=2 | 67 | 47792 | 2 | 480,7126 | 2 | -0,093 | 54,07 | ADLSGITGAR |  |
|  |  |  |  |  |  | 547,8715 | 2 | 0,1041 | 38,47 | NLAVSQVVHK |  |
| 32 | P04003 | C4b-binding protein alpha chain OS=Homo sapiens OX=9606 GN=C4BPA PE=1 SV=2 | 55 | 69042 | 2 | 565,1239 | 2 | 0,6899 | 23,18 | YTCLPGYVR |  |
|  |  |  |  |  |  | 625,6042 | 2 | 0,5225 | 55,26 | EDVYVVGTVLR |  |
| 33 | P01008 | Antithrombin-III OS=Homo sapiens OX=9606 GN=SERPINC1 PE=1 SV=1 | 53 | 53025 | 2 | 703,606 | 2 | 0,4539 | 52,89 | EVPLNTIIFMGR | Oxidation (M) |
|  |  |  |  |  |  | 1134,378 | 3 | -0,5386 | 21,87 | ELTPEVLQEWLDELEEMMLVVHMPRFR | 2 Oxidation (M) |

**Table S2.7** PEG-MWCNTs-wtDAAO Hard Corona R1

| **Prot. Number** | | **Accession Number** | **Protein Name** | **Mascot Score** | **Mr** | **N° pep** | **m/z** | **z** | **Pep. error** | **Pep. Score** | **Pep. sequence** | **Pep. Modification** |
| --- | --- | --- | --- | --- | --- | --- | --- | --- | --- | --- | --- | --- |
| 1 | P02768 | | Serum albumin OS=Homo sapiens OX=9606 GN=ALB PE=1 SV=2 | 5655 | 71317 | 29 | 673,352 | 1 | -0,026 | 27,72 | AWAVAR |  |
|  |  | |  |  |  |  | 386,6339 | 2 | -0,1781 | 44,1 | AACLLPK |  |
|  |  | |  |  |  |  | 395,2994 | 2 | 0,1198 | 24,53 | LVTDLTK |  |
|  |  | |  |  |  |  | 440,7817 | 2 | 0,115 | 39,48 | AEFAEVSK |  |
|  |  | |  |  |  |  | 927,6086 | 1 | 0,1152 | 28,01 | YLYEIAR |  |
|  |  | |  |  |  |  | 467,2341 | 2 | -0,0577 | 52,78 | LCTVATLR |  |
|  |  | |  |  |  |  | 470,7659 | 2 | 0,0762 | 29,78 | DDNPNLPR |  |
|  |  | |  |  |  |  | 476,3705 | 2 | 0,2919 | 20,87 | DLGEENFK |  |
|  |  | |  |  |  |  | 480,6784 | 2 | -0,213 | 37,11 | FQNALLVR |  |
|  |  | |  |  |  |  | 984,4033 | 1 | -0,0851 | 26,51 | TYETTLEK |  |
|  |  | |  |  |  |  | 500,7944 | 2 | -0,0222 | 36,23 | QTALVELVK |  |
|  |  | |  |  |  |  | 1013,199 | 1 | -0,3999 | 45,35 | LVAASQAALGL |  |
|  |  | |  |  |  |  | 509,4262 | 2 | 0,3086 | 32,61 | SLHTLFGDK |  |
|  |  | |  |  |  |  | 537,9702 | 2 | 0,3906 | 28,75 | LDELRDEGK |  |
|  |  | |  |  |  |  | 564,6983 | 2 | -0,3093 | 23,38 | KQTALVELVK |  |
|  |  | |  |  |  |  | 570,0107 | 2 | 0,5163 | 46,29 | CCTESLVNR |  |
|  |  | |  |  |  |  | 575,0878 | 2 | -0,4467 | 37,82 | LVNEVTEFAK |  |
|  |  | |  |  |  |  | 613,6091 | 2 | -0,3942 | 39,25 | FKDLGEENFK |  |
|  |  | |  |  |  |  | 679,41 | 2 | -0,8169 | 52,93 | AVMDDFAAFVEK | Oxidation (M) |
|  |  | |  |  |  |  | 686,2279 | 2 | -0,1182 | 51,45 | AAFTECCQAADK |  |
|  |  | |  |  |  |  | 722,4249 | 2 | 0,2006 | 41,74 | YICENQDSISSK |  |
|  |  | |  |  |  |  | 749,8992 | 2 | 0,2126 | 77,12 | TCVADESAENCDK |  |
|  |  | |  |  |  |  | 756,2401 | 2 | -0,3699 | 31,11 | VPQVSTPTLVEVSR |  |
|  |  | |  |  |  |  | 776,6327 | 2 | -0,3396 | 24,65 | CCAAADPHECYAK |  |
|  |  | |  |  |  |  | 547,312 | 3 | -0,0163 | 55,23 | KVPQVSTPTLVEVSR |  |
|  |  | |  |  |  |  | 820,4927 | 2 | 0,1957 | 34,61 | DVFLGMFLYEYAR | Oxidation (M) |
|  |  | |  |  |  |  | 820,5273 | 2 | 0,1095 | 73,2 | KVPQVSTPTLVEVSR |  |
|  |  | |  |  |  |  | 820,6973 | 2 | 0,6048 | 61,53 | DVFLGMFLYEYAR | Oxidation (M) |
|  |  | |  |  |  |  | 547,4697 | 3 | 0,4569 | 50,35 | KVPQVSTPTLVEVSR |  |
|  |  | |  |  |  |  | 829,1074 | 2 | -0,5451 | 31,97 | QNCELFEQLGEYK |  |
|  |  | |  |  |  |  | 956,1945 | 2 | 0,45 | 33,95 | RPCFSALEVDETYVPK |  |
|  |  | |  |  |  |  | 1023,259 | 2 | 0,4152 | 54,26 | VFDEFKPLVEEPQNLIK |  |
| 2 | P04114 | | Apolipoprotein B-100 OS=Homo sapiens OX=9606 GN=APOB PE=1 SV=2 | 1524 | 516651 | 39 | 415,0432 | 2 | 0,6217 | 34,82 | LGNNPVSK |  |
|  |  | |  |  |  |  | 462,5093 | 2 | 0,528 | 22,57 | QSFDLSVK |  |
|  |  | |  |  |  |  | 463,3558 | 2 | 0,2596 | 48,15 | GMALFGEGK | Oxidation (M) |
|  |  | |  |  |  |  | 481,2904 | 2 | 0,0422 | 39,73 | LDVTTSIGR |  |
|  |  | |  |  |  |  | 500,2574 | 2 | 0,026 | 26,86 | MGLAFESTK | Oxidation (M) |
|  |  | |  |  |  |  | 508,364 | 2 | 0,1749 | 53 | VSTAFVYTK |  |
|  |  | |  |  |  |  | 515,033 | 2 | 0,4488 | 26,72 | LSNVLQQVK |  |
|  |  | |  |  |  |  | 1039,584 | 1 | -0,0558 | 34,46 | LAPGELTIIL |  |
|  |  | |  |  |  |  | 523,1169 | 2 | -0,3774 | 35,83 | IPSVQINFK |  |
|  |  | |  |  |  |  | 556,0143 | 2 | 0,4964 | 30,3 | VPQTDMTFR | Oxidation (M) |
|  |  | |  |  |  |  | 593,9114 | 2 | 0,2093 | 45,57 | SNTVASLHTEK |  |
|  |  | |  |  |  |  | 599,113 | 2 | 0,636 | 74,57 | NMEVSVATTTK | Oxidation (M) |
|  |  | |  |  |  |  | 622,2197 | 2 | 0,7956 | 50,53 | ATGVLYDYVNK |  |
|  |  | |  |  |  |  | 643,8646 | 2 | -0,0095 | 67,16 | NTLELSNGVIVK |  |
|  |  | |  |  |  |  | 654,9647 | 2 | -0,7776 | 41,04 | EVGTVLSQVYSK |  |
|  |  | |  |  |  |  | 654,9688 | 2 | 0,2468 | 38,09 | GFEPTLEALFGK |  |
|  |  | |  |  |  |  | 655,2247 | 2 | -0,2576 | 24,84 | EVGTVLSQVYSK |  |
|  |  | |  |  |  |  | 678,1739 | 2 | 0,6371 | 56,2 | YGMVAQVTQTLK | Oxidation (M) |
|  |  | |  |  |  |  | 678,7026 | 2 | -0,4157 | 26,73 | LPYTIITTPPLK |  |
|  |  | |  |  |  |  | 681,3361 | 2 | 0,9582 | 56,61 | INNQLTLDSNTK |  |
|  |  | |  |  |  |  | 694,0514 | 2 | 0,3117 | 36,07 | IAELSATAQEIIK |  |
|  |  | |  |  |  |  | 700,2888 | 2 | -0,234 | 69,19 | TLADLTLLDSPIK |  |
|  |  | |  |  |  |  | 715,5988 | 2 | -0,5987 | 47,67 | ALVEQGFTVPEIK |  |
|  |  | |  |  |  |  | 722,4561 | 2 | -0,7762 | 26,59 | LQSTTVMNPYMK | 2 Oxidation (M) |
|  |  | |  |  |  |  | 727,3673 | 2 | 0,9217 | 34,55 | LNGEIQALELPQK |  |
|  |  | |  |  |  |  | 753,4221 | 2 | 0,0564 | 32,99 | IGQDGISTSATTNLK |  |
|  |  | |  |  |  |  | 779,8824 | 2 | -0,0382 | 44,24 | ITENDIQIALDDAK |  |
|  |  | |  |  |  |  | 786,1105 | 2 | 0,3515 | 55,59 | TLQGIPQMIGEVIR | Oxidation (M) |
|  |  | |  |  |  |  | 791,4704 | 2 | 0,1394 | 72,8 | AVSMPSFSILGSDVR | Oxidation (M) |
|  |  | |  |  |  |  | 796,5071 | 2 | 0,138 | 81,04 | VLLDQLGTTISFER |  |
|  |  | |  |  |  |  | 808,1565 | 2 | 0,5807 | 65,68 | MYQMDIQQELQR | 2 Oxidation (M) |
|  |  | |  |  |  |  | 808,8356 | 2 | -0,2255 | 74,15 | TSSFALNLPTLPEVK |  |
|  |  | |  |  |  |  | 813,3305 | 2 | 0,865 | 90,88 | YEVDQQIQVLMDK | Oxidation (M) |
|  |  | |  |  |  |  | 829,477 | 2 | 0,0713 | 27,1 | SVSDGIAALDLNAVANK |  |
|  |  | |  |  |  |  | 834,2059 | 2 | 0,4784 | 30,41 | GIISALLVPPETEEAK |  |
|  |  | |  |  |  |  | 874,1594 | 2 | 0,4207 | 47,67 | VNWEEEAASGLLTSLK |  |
|  |  | |  |  |  |  | 874,3368 | 2 | 0,7602 | 39,43 | IEGNLIFDPNNYLPK |  |
|  |  | |  |  |  |  | 900,2664 | 2 | 0,6285 | 70,98 | VNQNLVYESGSLNFSK |  |
|  |  | |  |  |  |  | 982,3182 | 2 | 0,5723 | 54,21 | TILGTMPAFEVSLQALQK | Oxidation (M) |
|  |  | |  |  |  |  | 678,4269 | 3 | 0,3092 | 59,02 | IHSGSFQSQVELSNDQEK |  |
| 3 | P01834 | | Immunoglobulin kappa constant OS=Homo sapiens OX=9606 GN=IGKC PE=1 SV=2 | 860 | 11929 | 5 | 751,677 | 2 | -0,4117 | 72,08 | DSTYSLSSTLTLSK |  |
|  |  | |  |  |  |  | 899,4586 | 2 | 0,0147 | 47,13 | SGTASVVCLLNNFYPR |  |
|  |  | |  |  |  |  | 626,3137 | 3 | 0,9997 | 20,99 | VYACEVTHQGLSSPVTK |  |
|  |  | |  |  |  |  | 973,4105 | 2 | -0,2132 | 35,97 | TVAAPSVFIFPPSDEQLK |  |
|  |  | |  |  |  |  | 1068,732 | 2 | 0,4888 | 113,5 | VDNALQSGNSQESVTEQDSK |  |
| 4 | P0DOX5 | | Immunoglobulin gamma-1 heavy chain OS=Homo sapiens OX=9606 PE=1 SV=2 | 795 | 49925 | 7 | 837,8199 | 1 | -0,6833 | 23,89 | ALPAPIEK |  |
|  |  | |  |  |  |  | 425,9698 | 2 | -0,4968 | 36,17 | DTLMISR | Oxidation (M) |
|  |  | |  |  |  |  | 581,28 | 2 | -0,077 | 28,96 | NQVSLTCLVK |  |
|  |  | |  |  |  |  | 593,5699 | 2 | -0,514 | 46,04 | GPSVFPLAPSSK |  |
|  |  | |  |  |  |  | 643,9558 | 2 | 0,2304 | 35,13 | EPQVYTLPPSR |  |
|  |  | |  |  |  |  | 661,3245 | 2 | -0,0364 | 76,97 | STSGGTAALGCLVK |  |
|  |  | |  |  |  |  | 937,1014 | 2 | -0,7262 | 31,37 | TTPPVLDSDGSFFLYSK |  |
| 5 | P0DOX7 | | Immunoglobulin kappa light chain OS=Homo sapiens OX=9606 PE=1 SV=1 | 574 | 23650 | 5 | 751,677 | 2 | -0,4117 | 72,08 | DSTYSLSSTLTLSK |  |
|  |  | |  |  |  |  | 899,4586 | 2 | 0,0147 | 47,13 | SGTASVVCLLNNFYPR |  |
|  |  | |  |  |  |  | 626,3137 | 3 | 0,9997 | 20,99 | VYACEVTHQGLSSPVTK |  |
|  |  | |  |  |  |  | 1002,323 | 2 | 0,5905 | 48,76 | GTVAAPSVFIFPPSDEQLK |  |
|  |  | |  |  |  |  | 1068,732 | 2 | 0,4888 | 113,5 | VDNALQSGNSQESVTEQDSK |  |
| 6 | A0M8Q6 | | Immunoglobulin lambda constant 7 OS=Homo sapiens OX=9606 GN=IGLC7 PE=1 SV=3 | 300 | 11418 | 2 | 872,4025 | 2 | -0,0611 | 58,26 | YAASSYLSLTPEQWK |  |
|  |  | |  |  |  |  | 993,5496 | 2 | 0,074 | 74,42 | AAPSVTLFPPSSEELQANK |  |
| 7 | P01009 | | Alpha-1-antitrypsin OS=Homo sapiens OX=9606 GN=SERPINA1 PE=1 SV=3 | 247 | 46878 | 4 | 427,1337 | 2 | 0,7664 | 35,87 | SASLHLPK |  |
|  |  | |  |  |  |  | 508,3612 | 2 | 0,1006 | 61,78 | SVLGQLGITK |  |
|  |  | |  |  |  |  | 556,0463 | 2 | 0,4811 | 48,89 | LSITGTYDLK |  |
|  |  | |  |  |  |  | 917,7568 | 2 | 0,5835 | 94,69 | VFSNGADLSGVTEEAPLK |  |
| 8 | P01024 | | Complement C3 OS=Homo sapiens OX=9606 GN=C3 PE=1 SV=2 | 223 | 188569 | 10 | 512,8679 | 2 | 0,2154 | 33,61 | FISLGEACK |  |
|  |  | |  |  |  |  | 542,1265 | 2 | -0,3124 | 52,24 | GYTQQLAFR |  |
|  |  | |  |  |  |  | 556,2312 | 2 | 0,8285 | 26,97 | VLLDGVQNPR |  |
|  |  | |  |  |  |  | 577,2191 | 2 | 0,8228 | 25,91 | FISLGEACKK |  |
|  |  | |  |  |  |  | 736,2036 | 2 | 0,6201 | 22,89 | IPIEDGSGEVVLSR |  |
|  |  | |  |  |  |  | 828,7084 | 2 | 0,5422 | 25,63 | TVMVNIENPEGIPVK | Oxidation (M) |
|  |  | |  |  |  |  | 829,6152 | 2 | 0,4593 | 57,23 | AGDFLEANYMNLQR | Oxidation (M) |
|  |  | |  |  |  |  | 852,244 | 2 | 0,688 | 72,47 | VFLDCCNYITELR |  |
|  |  | |  |  |  |  | 940,0721 | 2 | 0,1635 | 67,39 | EYVLPSFEVIVEPTEK |  |
|  |  | |  |  |  |  | 1107,186 | 2 | -0,6389 | 65,75 | EDIPPADLSDQVPDTESETR |  |
| 9 | P02647 | | Apolipoprotein A-I OS=Homo sapiens OX=9606 GN=APOA1 PE=1 SV=1 | 190 | 30759 | 6 | 524,2537 | 2 | -0,0137 | 55,22 | LSPLGEEMR | Oxidation (M) |
|  |  | |  |  |  |  | 608,7235 | 2 | 0,8182 | 32,97 | ATEHLSTLSEK |  |
|  |  | |  |  |  |  | 627,1404 | 2 | 0,6527 | 38,45 | VQPYLDDFQK |  |
|  |  | |  |  |  |  | 701,1085 | 2 | 0,5405 | 84,33 | DYVSQFEGSALGK |  |
|  |  | |  |  |  |  | 732,3781 | 2 | 0,8973 | 49,09 | VKDLATVYVDVLK |  |
|  |  | |  |  |  |  | 806,9785 | 2 | 0,1644 | 85,18 | LLDNWDSVTSTFSK |  |
| 10 | P0CG04 | | Immunoglobulin lambda constant 1 OS=Homo sapiens OX=9606 GN=IGLC1 PE=1 SV=1 | 172 | 11512 | 2 | 872,4025 | 2 | -0,0611 | 58,26 | YAASSYLSLTPEQWK |  |
|  |  | |  |  |  |  | 1022,166 | 2 | 0,2855 | 72,79 | ANPTVTLFPPSSEELQANK |  |
| 11 | P01859 | | Immunoglobulin heavy constant gamma 2 OS=Homo sapiens OX=9606 GN=IGHG2 PE=1 SV=2 | 152 | 36505 | 5 | 425,9698 | 2 | -0,4968 | 36,17 | DTLMISR | Oxidation (M) |
|  |  | |  |  |  |  | 581,28 | 2 | -0,077 | 28,96 | NQVSLTCLVK |  |
|  |  | |  |  |  |  | 643,9558 | 2 | 0,2304 | 35,13 | EPQVYTLPPSR |  |
|  |  | |  |  |  |  | 712,5006 | 2 | 0,2842 | 63,95 | STSESTAALGCLVK |  |
|  |  | |  |  |  |  | 961,8304 | 2 | 0,7648 | 34,36 | TTPPMLDSDGSFFLYSK | Oxidation (M) |
| 12 | P01876 | | Immunoglobulin heavy constant alpha 1 OS=Homo sapiens OX=9606 GN=IGHA1 PE=1 SV=2 | 150 | 38486 | 2 | 771,1083 | 2 | 0,4816 | 72,31 | DASGVTFTWTPSSGK |  |
|  |  | |  |  |  |  | 918,6786 | 2 | 0,4001 | 88,03 | QEPSQGTTTFAVTSILR |  |
| 13 | P01861 | | Immunoglobulin heavy constant gamma 4 OS=Homo sapiens OX=9606 GN=IGHG4 PE=1 SV=1 | 148 | 36431 | 4 | 425,9698 | 2 | -0,4968 | 36,17 | DTLMISR | Oxidation (M) |
|  |  | |  |  |  |  | 581,28 | 2 | -0,077 | 28,96 | NQVSLTCLVK |  |
|  |  | |  |  |  |  | 712,5006 | 2 | 0,2842 | 63,95 | STSESTAALGCLVK |  |
|  |  | |  |  |  |  | 951,7108 | 2 | 0,4864 | 57,54 | TTPPVLDSDGSFFLYSR |  |
| 14 | P00738 | | Haptoglobin OS=Homo sapiens OX=9606 GN=HP PE=1 SV=1 | 145 | 45861 | 2 | 602,3164 | 2 | -0,0113 | 60,85 | VTSIQDWVQK |  |
|  |  | |  |  |  |  | 673,1199 | 2 | -0,4132 | 38,46 | SCAVAEYGVYVK |  |
| 15 | P0DOX6 | | Immunoglobulin mu heavy chain OS=Homo sapiens OX=9606 PE=1 SV=2 | 125 | 64244 | 2 | 639,5253 | 2 | 0,3333 | 67,52 | YAATSQVLLPSK |  |
|  |  | |  |  |  |  | 808,8934 | 2 | 0,0058 | 62,67 | YVTSAPMPEPQAPGR | Oxidation (M) |
| 16 | P0C0L4 | | Complement C4-A OS=Homo sapiens OX=9606 GN=C4A PE=1 SV=2 | 119 | 194261 | 2 | 771,5046 | 2 | 0,1851 | 95,96 | VLSLAQEQVGGSPEK |  |
|  |  | |  |  |  |  | 1242,372 | 2 | 0,4369 | 50,97 | VTASDPLDTLGSEGALSPGGVASLLR |  |
| 17 | P02787 | | Serotransferrin OS=Homo sapiens OX=9606 GN=TF PE=1 SV=3 | 108 | 79294 | 3 | 642,7087 | 2 | 0,8411 | 27,1 | EGYYGYTGAFR |  |
|  |  | |  |  |  |  | 748,1897 | 2 | 0,6424 | 71,63 | MYLGYEYVTAIR | Oxidation (M) |
|  |  | |  |  |  |  | 815,6217 | 2 | 0,4202 | 61,73 | EDPQTFYYAVAVVK |  |
| 18 | P01023 | | Alpha-2-macroglobulin OS=Homo sapiens OX=9606 GN=A2M PE=1 SV=3 | 104 | 164613 | 4 | 524,108 | 2 | 0,6207 | 47,27 | FEVQVTVPK |  |
|  |  | |  |  |  |  | 606,1609 | 2 | 0,6719 | 36,87 | LPPNVVEESAR |  |
|  |  | |  |  |  |  | 628,595 | 2 | 0,5397 | 30,06 | AIGYLNTGYQR |  |
|  |  | |  |  |  |  | 923,0657 | 2 | 0,0873 | 56,16 | LLIYAVLPTGDVIGDSAK |  |
| 19 | P19823 | | Inter-alpha-trypsin inhibitor heavy chain H2 OS=Homo sapiens OX=9606 GN=ITIH2 PE=1 SV=2 | 100 | 106853 | 2 | 792,1949 | 2 | 0,5279 | 72,39 | IQPSGGTNINEALLR |  |
|  |  | |  |  |  |  | 1061,757 | 2 | 0,3782 | 60,01 | VVNNSPQPQNVVFDVQIPK |  |
| 20 | O14686 | | Histone-lysine N-methyltransferase 2D OS=Homo sapiens OX=9606 GN=KMT2D PE=1 SV=2 | 52 | 599575 | 2 | 654,1255 | 1 | -0,2678 | 23,13 | APLTPR |  |
|  |  | |  |  |  |  | 476,4765 | 2 | 0,4927 | 51,67 | IYEEQNR |  |

**Table S2.8** PEG-MWCNTs-wtDAAO Hard Corona R2

| **Prot. Number** | **Accession Number** | **Protein Name** | **Mascot Score** | **Mr** | **N° pep** | **m/z** | **z** | **Pep. error** | **Pep. Score** | **Pep. sequence** | **Pep. Modification** |
| --- | --- | --- | --- | --- | --- | --- | --- | --- | --- | --- | --- |
| 1 | P02768 | Serum albumin OS=Homo sapiens OX=9606 GN=ALB PE=1 SV=2 | 4670 | 71317 | 27 | 673,4532 | 1 | 0,0752 | 23,1 | AWAVAR |  |
|  |  |  |  |  |  | 387,0811 | 2 | 0,7164 | 31,99 | AACLLPK |  |
|  |  |  |  |  |  | 395,1843 | 2 | -0,1103 | 22,89 | LVTDLTK |  |
|  |  |  |  |  |  | 440,4673 | 2 | -0,5137 | 35,35 | AEFAEVSK |  |
|  |  |  |  |  |  | 464,3887 | 2 | 0,2767 | 27,82 | YLYEIAR |  |
|  |  |  |  |  |  | 467,206 | 2 | -0,1139 | 22,07 | LCTVATLR |  |
|  |  |  |  |  |  | 470,4076 | 2 | -0,6402 | 22,05 | DDNPNLPR |  |
|  |  |  |  |  |  | 480,7163 | 2 | -0,1373 | 38,69 | FQNALLVR |  |
|  |  |  |  |  |  | 492,5424 | 2 | -0,4108 | 23,65 | TYETTLEK |  |
|  |  |  |  |  |  | 500,9041 | 2 | 0,1972 | 54,95 | QTALVELVK |  |
|  |  |  |  |  |  | 1013,207 | 1 | -0,3924 | 30,8 | LVAASQAALGL |  |
|  |  |  |  |  |  | 509,6686 | 2 | 0,7935 | 38,99 | SLHTLFGDK |  |
|  |  |  |  |  |  | 538,0383 | 2 | 0,5268 | 39,41 | LDELRDEGK |  |
|  |  |  |  |  |  | 564,4845 | 2 | -0,7369 | 23,09 | KQTALVELVK |  |
|  |  |  |  |  |  | 571,5458 | 2 | 0,3904 | 20,07 | KLVAASQAALGL |  |
|  |  |  |  |  |  | 1149,348 | 1 | -0,2669 | 26,24 | LVNEVTEFAK |  |
|  |  |  |  |  |  | 613,6523 | 2 | -0,3078 | 29,88 | FKDLGEENFK |  |
|  |  |  |  |  |  | 679,41 | 2 | -0,8169 | 52,93 | AVMDDFAAFVEK | Oxidation (M) |
|  |  |  |  |  |  | 686,5273 | 2 | 0,4806 | 38,54 | AAFTECCQAADK |  |
|  |  |  |  |  |  | 722,4583 | 2 | 0,2673 | 81,83 | YICENQDSISSK |  |
|  |  |  |  |  |  | 725,8218 | 2 | 0,108 | 55,38 | ETYGEMADCCAK | Oxidation (M) |
|  |  |  |  |  |  | 749,9197 | 2 | 0,2536 | 83,57 | TCVADESAENCDK |  |
|  |  |  |  |  |  | 756,2401 | 2 | -0,3699 | 31,11 | VPQVSTPTLVEVSR |  |
|  |  |  |  |  |  | 820,1992 | 2 | -0,5466 | 45,88 | KVPQVSTPTLVEVSR |  |
|  |  |  |  |  |  | 820,3975 | 2 | 0,0052 | 78,2 | DVFLGMFLYEYAR | Oxidation (M) |
|  |  |  |  |  |  | 547,269 | 3 | -0,1454 | 49,87 | KVPQVSTPTLVEVSR |  |
|  |  |  |  |  |  | 820,4639 | 2 | 0,138 | 44,42 | DVFLGMFLYEYAR | Oxidation (M) |
|  |  |  |  |  |  | 820,4756 | 2 | 0,0063 | 59,52 | KVPQVSTPTLVEVSR |  |
|  |  |  |  |  |  | 820,7706 | 2 | 0,7515 | 46,01 | DVFLGMFLYEYAR | Oxidation (M) |
|  |  |  |  |  |  | 547,5162 | 3 | 0,5964 | 55,4 | KVPQVSTPTLVEVSR |  |
|  |  |  |  |  |  | 820,8063 | 2 | 0,8228 | 70,41 | DVFLGMFLYEYAR | Oxidation (M) |
|  |  |  |  |  |  | 547,5444 | 3 | 0,681 | 50,72 | KVPQVSTPTLVEVSR |  |
|  |  |  |  |  |  | 829,2213 | 2 | -0,3174 | 33,24 | QNCELFEQLGEYK |  |
|  |  |  |  |  |  | 1022,903 | 2 | -0,2965 | 68,96 | VFDEFKPLVEEPQNLIK |  |
| 2 | P04114 | Apolipoprotein B-100 OS=Homo sapiens OX=9606 GN=APOB PE=1 SV=2 | 1336 | 516651 | 41 | 422,7883 | 2 | 0,0807 | 36,95 | IGVELTGR |  |
|  |  |  |  |  |  | 426,2421 | 2 | -0,0328 | 25,93 | LHVAGNLK |  |
|  |  |  |  |  |  | 476,0997 | 2 | 0,698 | 34,63 | FVTQAEGAK |  |
|  |  |  |  |  |  | 481,1851 | 2 | -0,1684 | 38,15 | LDVTTSIGR |  |
|  |  |  |  |  |  | 508,6695 | 2 | 0,7858 | 39,39 | VSTAFVYTK |  |
|  |  |  |  |  |  | 509,3594 | 2 | 0,1177 | 35,69 | LATALSLSNK |  |
|  |  |  |  |  |  | 1039,379 | 1 | -0,2606 | 29,96 | LAPGELTIIL |  |
|  |  |  |  |  |  | 523,329 | 2 | 0,0466 | 33,19 | IPSVQINFK |  |
|  |  |  |  |  |  | 556,1312 | 2 | 0,7303 | 37,75 | VPQTDMTFR | Oxidation (M) |
|  |  |  |  |  |  | 576,347 | 2 | 0,1177 | 32,92 | LDFSSQADLR |  |
|  |  |  |  |  |  | 598,7641 | 2 | -0,0618 | 53,52 | NMEVSVATTTK | Oxidation (M) |
|  |  |  |  |  |  | 618,1797 | 2 | -0,2494 | 49,88 | ENFAGEATLQR |  |
|  |  |  |  |  |  | 621,7062 | 2 | -0,2314 | 35,3 | ATGVLYDYVNK |  |
|  |  |  |  |  |  | 643,4794 | 2 | -0,78 | 55,13 | NTLELSNGVIVK |  |
|  |  |  |  |  |  | 655,3324 | 2 | 0,9741 | 44,22 | GFEPTLEALFGK |  |
|  |  |  |  |  |  | 655,5079 | 2 | 0,3088 | 26,76 | EVGTVLSQVYSK |  |
|  |  |  |  |  |  | 661,4656 | 2 | -0,75 | 27,13 | NPNGYSFSIPVK |  |
|  |  |  |  |  |  | 677,9446 | 2 | 0,1784 | 77,78 | YGMVAQVTQTLK | Oxidation (M) |
|  |  |  |  |  |  | 678,8404 | 2 | -0,1402 | 51,99 | LPYTIITTPPLK |  |
|  |  |  |  |  |  | 694,0584 | 2 | 0,3256 | 57,68 | IAELSATAQEIIK |  |
|  |  |  |  |  |  | 700,294 | 2 | -0,2236 | 70,5 | TLADLTLLDSPIK |  |
|  |  |  |  |  |  | 715,6071 | 2 | -0,5821 | 50,39 | ALVEQGFTVPEIK |  |
|  |  |  |  |  |  | 723,1158 | 2 | 0,5433 | 42,54 | LQSTTVMNPYMK | 2 Oxidation (M) |
|  |  |  |  |  |  | 753,7235 | 2 | 0,6592 | 66,51 | IGQDGISTSATTNLK |  |
|  |  |  |  |  |  | 763,2986 | 2 | 0,9884 | 47,17 | LSNDMMGSYAEMK | 3 Oxidation (M) |
|  |  |  |  |  |  | 768,2376 | 2 | -0,3241 | 33,36 | CVQSTKPSLMIQK | Oxidation (M) |
|  |  |  |  |  |  | 779,6269 | 2 | -0,5492 | 56,16 | ITENDIQIALDDAK |  |
|  |  |  |  |  |  | 785,9933 | 2 | 0,1172 | 47,07 | TLQGIPQMIGEVIR | Oxidation (M) |
|  |  |  |  |  |  | 796,8091 | 2 | 0,742 | 52,96 | VLLDQLGTTISFER |  |
|  |  |  |  |  |  | 807,813 | 2 | -0,1062 | 72 | MYQMDIQQELQR | 2 Oxidation (M) |
|  |  |  |  |  |  | 809,3294 | 2 | 0,7622 | 58,01 | TSSFALNLPTLPEVK |  |
|  |  |  |  |  |  | 813,2877 | 2 | 0,7795 | 90,94 | YEVDQQIQVLMDK | Oxidation (M) |
|  |  |  |  |  |  | 815,2545 | 2 | -0,2447 | 75,02 | MTSNFPVDLSDYPK | Oxidation (M) |
|  |  |  |  |  |  | 829,6216 | 2 | 0,3604 | 67,12 | SVSDGIAALDLNAVANK |  |
|  |  |  |  |  |  | 873,8148 | 2 | -0,2837 | 39,85 | IEGNLIFDPNNYLPK |  |
|  |  |  |  |  |  | 917,5678 | 2 | 0,1537 | 65,29 | ATFQTPDFIVPLTDLR |  |
|  |  |  |  |  |  | 937,7178 | 2 | 0,3748 | 45,85 | FSVPAGIVIPSFQALTAR |  |
|  |  |  |  |  |  | 944,9241 | 2 | 0,9041 | 58,5 | LLLQMDSSATAYGSTVSK | Oxidation (M) |
|  |  |  |  |  |  | 982,2557 | 2 | 0,4473 | 62,1 | TILGTMPAFEVSLQALQK | Oxidation (M) |
|  |  |  |  |  |  | 678,5809 | 3 | 0,7712 | 74,46 | IHSGSFQSQVELSNDQEK |  |
|  |  |  |  |  |  | 1233,909 | 2 | 0,4367 | 65,72 | IADFELPTIIVPEQTIEIPSIK |  |
| 3 | P01834 | Immunoglobulin kappa constant OS=Homo sapiens OX=9606 GN=IGKC PE=1 SV=2 | 798 | 11929 | 4 | 751,5946 | 2 | -0,5765 | 72,41 | DSTYSLSSTLTLSK |  |
|  |  |  |  |  |  | 973,3529 | 2 | -0,3284 | 77,33 | TVAAPSVFIFPPSDEQLK |  |
|  |  |  |  |  |  | 701,6627 | 3 | 0,8455 | 22,07 | RTVAAPSVFIFPPSDEQLK |  |
|  |  |  |  |  |  | 1068,922 | 2 | 0,8685 | 68,49 | VDNALQSGNSQESVTEQDSK |  |
| 4 | P0DOX5 | Immunoglobulin gamma-1 heavy chain OS=Homo sapiens OX=9606 PE=1 SV=2 | 255 | 49925 | 6 | 838,697 | 1 | 0,1938 | 23,7 | ALPAPIEK |  |
|  |  |  |  |  |  | 581,6128 | 2 | 0,5887 | 40,32 | NQVSLTCLVK |  |
|  |  |  |  |  |  | 593,9306 | 2 | 0,2073 | 44,53 | GPSVFPLAPSSK |  |
|  |  |  |  |  |  | 644,2333 | 2 | 0,7854 | 23,39 | EPQVYTLPPSR |  |
|  |  |  |  |  |  | 937,0339 | 2 | 0,0905 | 25,4 | EPQVYTLPPSRDELTK |  |
|  |  |  |  |  |  | 937,3024 | 2 | -0,3244 | 61,8 | TTPPVLDSDGSFFLYSK |  |
| 5 | P01009 | Alpha-1-antitrypsin OS=Homo sapiens OX=9606 GN=SERPINA1 PE=1 SV=3 | 187 | 46878 | 3 | 555,9501 | 2 | 0,2888 | 53,13 | LSITGTYDLK |  |
|  |  |  |  |  |  | 917,7885 | 2 | 0,6467 | 85,37 | VFSNGADLSGVTEEAPLK |  |
|  |  |  |  |  |  | 1145,812 | 2 | -0,514 | 49,03 | GTEAAGAMFLEAIPMSIPPEVK | 2 Oxidation (M) |
| 6 | P01024 | Complement C3 OS=Homo sapiens OX=9606 GN=C3 PE=1 SV=2 | 125 | 188569 | 6 | 402,7431 | 2 | 0,0328 | 41 | SVQLTEK |  |
|  |  |  |  |  |  | 542,7618 | 2 | 0,9582 | 58,69 | GYTQQLAFR |  |
|  |  |  |  |  |  | 556,2312 | 2 | 0,8285 | 26,97 | VLLDGVQNPR |  |
|  |  |  |  |  |  | 701,6627 | 2 | 0,483 | 28,24 | SSLSVPYVIVPLK |  |
|  |  |  |  |  |  | 828,8713 | 2 | 0,8681 | 38,98 | TVMVNIENPEGIPVK | Oxidation (M) |
|  |  |  |  |  |  | 1107,485 | 2 | -0,0415 | 79,16 | EDIPPADLSDQVPDTESETR |  |
| 7 | P02787 | Serotransferrin OS=Homo sapiens OX=9606 GN=TF PE=1 SV=3 | 59 | 79294 | 2 | 748,0805 | 2 | 0,424 | 23,81 | MYLGYEYVTAIR | Oxidation (M) |
|  |  |  |  |  |  | 815,3961 | 2 | -0,0309 | 61,51 | EDPQTFYYAVAVVK |  |

**Table S2.9** PEG-MWCNTs-mDAAO Soft Corona R1

| **Prot. Number** | **Accession Number** | **Protein Name** | **Mascot Score** | **Mr** | **N° pep** | **m/z** | **z** | **Pep. error** | **Pep. Score** | **Pep. sequence** | **Pep. Modification** |
| --- | --- | --- | --- | --- | --- | --- | --- | --- | --- | --- | --- |
| 1 | P02768 | Serum albumin OS=Homo sapiens OX=9606 GN=ALB PE=1 SV=2 | 5003 | 71317 | 33 | 673,4569 | 1 | 0,0788 | 27,99 | AWAVAR |  |
|  |  |  |  |  |  | 386,6495 | 2 | -0,1467 | 36,29 | AACLLPK |  |
|  |  |  |  |  |  | 395,3233 | 2 | 0,1676 | 32,25 | LVTDLTK |  |
|  |  |  |  |  |  | 880,4476 | 1 | 0,0066 | 44,81 | AEFAEVSK |  |
|  |  |  |  |  |  | 464,177 | 2 | -0,1467 | 35,08 | YLYEIAR |  |
|  |  |  |  |  |  | 467,1992 | 2 | -0,1275 | 50,45 | LCTVATLR |  |
|  |  |  |  |  |  | 470,7488 | 2 | 0,0421 | 20,1 | DDNPNLPR |  |
|  |  |  |  |  |  | 476,3233 | 2 | 0,1975 | 37,19 | DLGEENFK |  |
|  |  |  |  |  |  | 480,6515 | 2 | -0,2667 | 42,44 | FQNALLVR |  |
|  |  |  |  |  |  | 984,3047 | 1 | -0,1837 | 25 | TYETTLEK |  |
|  |  |  |  |  |  | 500,8355 | 2 | 0,06 | 69,71 | QTALVELVK |  |
|  |  |  |  |  |  | 1013,323 | 1 | -0,276 | 20,56 | LVAASQAALGL |  |
|  |  |  |  |  |  | 509,6619 | 2 | 0,7802 | 33,09 | SLHTLFGDK |  |
|  |  |  |  |  |  | 528,7239 | 2 | 0,8522 | 43,44 | KYLYEIAR |  |
|  |  |  |  |  |  | 537,6526 | 2 | -0,2447 | 37,03 | LDELRDEGK |  |
|  |  |  |  |  |  | 564,9125 | 2 | 0,119 | 45,37 | KQTALVELVK |  |
|  |  |  |  |  |  | 569,8276 | 2 | 0,1501 | 52,02 | CCTESLVNR |  |
|  |  |  |  |  |  | 571,3203 | 2 | -0,0606 | 36,4 | KLVAASQAALGL |  |
|  |  |  |  |  |  | 1149,317 | 1 | -0,2985 | 24,45 | LVNEVTEFAK |  |
|  |  |  |  |  |  | 614,1925 | 2 | 0,7726 | 43,82 | FKDLGEENFK |  |
|  |  |  |  |  |  | 1358,436 | 1 | -0,1934 | 25,78 | AVMDDFAAFVEK | Oxidation (M) |
|  |  |  |  |  |  | 686,1638 | 2 | -0,2464 | 51,73 | AAFTECCQAADK |  |
|  |  |  |  |  |  | 718,0692 | 2 | 0,5976 | 44,12 | ETYGEMADCCAK |  |
|  |  |  |  |  |  | 722,2873 | 2 | -0,0746 | 62,13 | YICENQDSISSK |  |
|  |  |  |  |  |  | 725,6762 | 2 | -0,1833 | 60,08 | ETYGEMADCCAK | Oxidation (M) |
|  |  |  |  |  |  | 749,5515 | 2 | -0,4827 | 100,74 | TCVADESAENCDK |  |
|  |  |  |  |  |  | 750,416 | 2 | 0,1929 | 32,62 | ADDKETCFAEEGK |  |
|  |  |  |  |  |  | 756,5246 | 2 | 0,1991 | 69,96 | VPQVSTPTLVEVSR |  |
|  |  |  |  |  |  | 516,5948 | 3 | 0,9733 | 37,34 | LKECCEKPLLEK |  |
|  |  |  |  |  |  | 776,7653 | 2 | -0,0745 | 22,13 | CCAAADPHECYAK |  |
|  |  |  |  |  |  | 547,1503 | 3 | -0,5015 | 23,88 | KVPQVSTPTLVEVSR |  |
|  |  |  |  |  |  | 829,5206 | 2 | 0,2812 | 41,85 | QNCELFEQLGEYK |  |
|  |  |  |  |  |  | 682,6561 | 3 | 0,8585 | 39,76 | VFDEFKPLVEEPQNLIK |  |
|  |  |  |  |  |  | 997,6755 | 3 | 0,6727 | 42,27 | SHCIAEVENDEMPADLPSLAADFVESK | Oxidation (M) |
| 2 | P02787 | Serotransferrin OS=Homo sapiens OX=9606 GN=TF PE=1 SV=3 | 1029 | 79294 | 16 | 368,4015 | 2 | 0,3921 | 43,13 | GDVAFVK |  |
|  |  |  |  |  |  | 438,0963 | 2 | 0,7436 | 35,12 | DSAHGFLK |  |
|  |  |  |  |  |  | 461,3552 | 2 | 0,2685 | 50,18 | DDTVCLAK |  |
|  |  |  |  |  |  | 490,0448 | 2 | 0,5932 | 66,55 | DGAGDVAFVK |  |
|  |  |  |  |  |  | 499,3219 | 2 | 0,1594 | 43,14 | ASYLDCIR |  |
|  |  |  |  |  |  | 501,0733 | 2 | 0,6407 | 22,52 | YLGEEYVK |  |
|  |  |  |  |  |  | 606,1493 | 2 | -0,256 | 37,62 | DSGFQMNQLR | Oxidation (M) |
|  |  |  |  |  |  | 625,4296 | 2 | 0,2461 | 69,89 | SASDLTWDNLK |  |
|  |  |  |  |  |  | 642,3089 | 2 | 0,0414 | 53,85 | EGYYGYTGAFR |  |
|  |  |  |  |  |  | 678,1265 | 2 | 0,615 | 64,37 | DYELLCLDGTR |  |
|  |  |  |  |  |  | 708,2479 | 2 | -0,2315 | 66,36 | SVIPSDGPSVACVK |  |
|  |  |  |  |  |  | 789,3199 | 2 | -0,0251 | 126,25 | FDEFFSEGCAPGSK |  |
|  |  |  |  |  |  | 815,6078 | 2 | 0,3924 | 67,4 | EDPQTFYYAVAVVK |  |
|  |  |  |  |  |  | 862,0837 | 2 | 0,3994 | 54,79 | LCMGSGLNLCEPNNK | Oxidation (M) |
|  |  |  |  |  |  | 863,5183 | 2 | 0,2624 | 81,08 | IECVSAETTEDCIAK |  |
|  |  |  |  |  |  | 606,7693 | 3 | 0,4889 | 21,84 | EGTCPEAPTDECKPVK |  |
| 3 | P0DOX5 | Immunoglobulin gamma-1 heavy chain OS=Homo sapiens OX=9606 PE=1 SV=2 | 762 | 49925 | 5 | 581,2833 | 2 | -0,0702 | 51,91 | NQVSLTCLVK |  |
|  |  |  |  |  |  | 593,8447 | 2 | 0,0354 | 67,29 | GPSVFPLAPSSK |  |
|  |  |  |  |  |  | 643,9664 | 2 | 0,2517 | 23,67 | EPQVYTLPPSR |  |
|  |  |  |  |  |  | 661,4422 | 2 | 0,1991 | 84,46 | STSGGTAALGCLVK |  |
|  |  |  |  |  |  | 937,5858 | 2 | 0,2425 | 33,15 | TTPPVLDSDGSFFLYSK |  |
| 4 | P01834 | Immunoglobulin kappa constant OS=Homo sapiens OX=9606 GN=IGKC PE=1 SV=2 | 690 | 11929 | 2 | 973,8828 | 2 | 0,7314 | 69,54 | TVAAPSVFIFPPSDEQLK |  |
|  |  |  |  |  |  | 1068,438 | 2 | -0,0998 | 142,47 | VDNALQSGNSQESVTEQDSK |  |
| 5 | P04114 | Apolipoprotein B-100 OS=Homo sapiens OX=9606 GN=APOB PE=1 SV=2 | 662 | 516651 | 24 | 431,4492 | 2 | 0,4082 | 22,13 | ITLPDFR |  |
|  |  |  |  |  |  | 500,6819 | 2 | 0,8749 | 32,52 | MGLAFESTK | Oxidation (M) |
|  |  |  |  |  |  | 506,7581 | 2 | -0,1311 | 56,27 | TGISPLALIK |  |
|  |  |  |  |  |  | 518,1016 | 2 | 0,6693 | 26,04 | DNVFDGLVR |  |
|  |  |  |  |  |  | 1039,473 | 1 | -0,1666 | 31,94 | LAPGELTIIL |  |
|  |  |  |  |  |  | 563,3284 | 2 | 0,0522 | 53,12 | LIDVISMYR | Oxidation (M) |
|  |  |  |  |  |  | 570,6576 | 2 | 0,7711 | 62,89 | EVYGFNPEGK |  |
|  |  |  |  |  |  | 599,0139 | 2 | 0,4378 | 43,6 | NMEVSVATTTK | Oxidation (M) |
|  |  |  |  |  |  | 615,0143 | 2 | 0,495 | 68,03 | NSEEFAAAMSR | Oxidation (M) |
|  |  |  |  |  |  | 622,1333 | 2 | 0,6229 | 59,81 | ATGVLYDYVNK |  |
|  |  |  |  |  |  | 635,1082 | 2 | 0,4882 | 25,32 | DLKVEDIPLAR |  |
|  |  |  |  |  |  | 644,2085 | 2 | 0,6783 | 73,36 | NTLELSNGVIVK |  |
|  |  |  |  |  |  | 655,3427 | 2 | -0,0218 | 53,25 | EVGTVLSQVYSK |  |
|  |  |  |  |  |  | 662,2976 | 2 | 0,914 | 44,07 | NPNGYSFSIPVK |  |
|  |  |  |  |  |  | 677,8306 | 2 | -0,0497 | 33,15 | YGMVAQVTQTLK | Oxidation (M) |
|  |  |  |  |  |  | 727,03 | 2 | 0,2472 | 36,58 | LNGEIQALELPQK |  |
|  |  |  |  |  |  | 753,7556 | 2 | 0,7234 | 89,77 | IGQDGISTSATTNLK |  |
|  |  |  |  |  |  | 791,2914 | 2 | 0,8204 | 92,03 | SGSSTASWIQNVDTK |  |
|  |  |  |  |  |  | 791,7014 | 2 | 0,6014 | 90,37 | AVSMPSFSILGSDVR | Oxidation (M) |
|  |  |  |  |  |  | 801,5763 | 2 | 0,292 | 63,18 | VSALLTPAEQTGTWK |  |
|  |  |  |  |  |  | 808,2169 | 2 | 0,7014 | 60,58 | MYQMDIQQELQR | 2 Oxidation (M) |
|  |  |  |  |  |  | 961,2155 | 2 | 0,4831 | 66,51 | VIGNMGQTMEQLTPELK | 2 Oxidation (M) |
|  |  |  |  |  |  | 1077,716 | 2 | 0,4998 | 105,81 | YTYNYEAESSSGVPGTADSR |  |
|  |  |  |  |  |  | 1372,053 | 2 | 0,6722 | 22,17 | ESMLKTTLTAFGFASADLIEIGLEGK |  |
| 6 | P01859 | Immunoglobulin heavy constant gamma 2 OS=Homo sapiens OX=9606 GN=IGHG2 PE=1 SV=2 | 437 | 36505 | 5 | 581,2833 | 2 | -0,0702 | 51,91 | NQVSLTCLVK |  |
|  |  |  |  |  |  | 643,9664 | 2 | 0,2517 | 23,67 | EPQVYTLPPSR |  |
|  |  |  |  |  |  | 644,1939 | 2 | -0,271 | 29,06 | GPSVFPLAPCSR |  |
|  |  |  |  |  |  | 644,2112 | 2 | 0,7412 | 33,22 | EPQVYTLPPSR |  |
|  |  |  |  |  |  | 712,5875 | 2 | 0,4581 | 70,67 | STSESTAALGCLVK |  |
|  |  |  |  |  |  | 961,6705 | 2 | 0,445 | 53,29 | TTPPMLDSDGSFFLYSK | Oxidation (M) |
| 7 | P01024 | Complement C3 OS=Homo sapiens OX=9606 GN=C3 PE=1 SV=2 | 339 | 188569 | 11 | 532,4207 | 2 | 0,2889 | 52,16 | DSCVGSLVVK |  |
|  |  |  |  |  |  | 570,3291 | 2 | 0,1102 | 25,41 | FYYIYNEK |  |
|  |  |  |  |  |  | 576,9968 | 2 | 0,3816 | 28,46 | QPSSAFAAFVK |  |
|  |  |  |  |  |  | 613,2789 | 2 | 0,9536 | 54,34 | YYTYLIMNK | Oxidation (M) |
|  |  |  |  |  |  | 621,8927 | 2 | 0,127 | 25,36 | QPVPGQQMTLK | Oxidation (M) |
|  |  |  |  |  |  | 641,4469 | 2 | 0,2908 | 26,23 | ENEGFTVTAEGK |  |
|  |  |  |  |  |  | 645,4374 | 2 | 0,2594 | 63,35 | SGSDEVQVGQQR |  |
|  |  |  |  |  |  | 650,5302 | 2 | -0,5348 | 26,94 | ACEPGVDYVYK |  |
|  |  |  |  |  |  | 828,4679 | 2 | 0,0612 | 74,53 | TVMVNIENPEGIPVK | Oxidation (M) |
|  |  |  |  |  |  | 619,2804 | 3 | -0,0289 | 49,72 | SEETKENEGFTVTAEGK |  |
|  |  |  |  |  |  | 1099,76 | 2 | 0,3828 | 121,09 | VPVAVQGEDTVQSLTQGDGVAK |  |
| 8 | P01876 | Immunoglobulin heavy constant alpha 1 OS=Homo sapiens OX=9606 GN=IGHA1 PE=1 SV=2 | 259 | 38486 | 7 | 448,871 | 2 | 0,2723 | 37,3 | YLTWASR |  |
|  |  |  |  |  |  | 466,3847 | 2 | 0,2163 | 50,7 | TPLTATLSK |  |
|  |  |  |  |  |  | 471,1931 | 2 | 0,8942 | 31,05 | SAVQGPPER |  |
|  |  |  |  |  |  | 607,5021 | 2 | 0,3645 | 42,56 | WLQGSQELPR |  |
|  |  |  |  |  |  | 688,3995 | 2 | 0,1718 | 52,3 | TFTCTAAYPESK |  |
|  |  |  |  |  |  | 771,1364 | 2 | 0,5377 | 82,91 | DASGVTFTWTPSSGK |  |
|  |  |  |  |  |  | 918,6055 | 2 | 0,2539 | 79,03 | QEPSQGTTTFAVTSILR |  |
| 9 | P01023 | Alpha-2-macroglobulin OS=Homo sapiens OX=9606 GN=A2M PE=1 SV=3 | 221 | 164613 | 10 | 509,8269 | 2 | 0,0533 | 40,23 | ATVLNYLPK |  |
|  |  |  |  |  |  | 524,1745 | 2 | 0,7536 | 58,61 | FEVQVTVPK |  |
|  |  |  |  |  |  | 552,3403 | 2 | 0,0679 | 39,44 | SSGSLLNNAIK |  |
|  |  |  |  |  |  | 561,1429 | 2 | 0,6425 | 23,07 | SIYKPGQTVK |  |
|  |  |  |  |  |  | 575,1171 | 2 | 0,6057 | 47,61 | QGIPFFGQVR |  |
|  |  |  |  |  |  | 605,789 | 2 | -0,0719 | 47,91 | LPPNVVEESAR |  |
|  |  |  |  |  |  | 628,3731 | 2 | 0,096 | 49,89 | AIGYLNTGYQR |  |
|  |  |  |  |  |  | 637,0286 | 2 | 0,377 | 59,56 | VTAAPQSVCALR |  |
|  |  |  |  |  |  | 638,5543 | 2 | 0,5339 | 21,38 | VGFYESDVMGR | Oxidation (M) |
|  |  |  |  |  |  | 698,2636 | 2 | 0,84 | 81,36 | NEDSLVFVQTDK |  |
| 10 | P00738 | Haptoglobin OS=Homo sapiens OX=9606 GN=HP PE=1 SV=1 | 219 | 45861 | 5 | 460,7611 | 2 | 0,0524 | 39,99 | GSFPWQAK |  |
|  |  |  |  |  |  | 490,8342 | 2 | 0,1662 | 64,92 | VGYVSGWGR |  |
|  |  |  |  |  |  | 645,7482 | 2 | -0,2413 | 62,69 | DIAPTLTLYVGK |  |
|  |  |  |  |  |  | 862,7606 | 2 | 0,6998 | 82,42 | YVMLPVADQDQCIR | Oxidation (M) |
|  |  |  |  |  |  | 730,3474 | 3 | 0,9749 | 43,72 | SPVGVQPILNEHTFCAGMSK | Oxidation (M) |
| 11 | P01009 | Alpha-1-antitrypsin OS=Homo sapiens OX=9606 GN=SERPINA1 PE=1 SV=3 | 151 | 46878 | 4 | 445,0162 | 2 | 0,5215 | 27,57 | AVLTIDEK |  |
|  |  |  |  |  |  | 505,0069 | 2 | 0,5068 | 20,17 | QINDYVEK |  |
|  |  |  |  |  |  | 508,3577 | 2 | 0,0936 | 56,08 | SVLGQLGITK |  |
|  |  |  |  |  |  | 556,0455 | 2 | 0,4796 | 51,31 | LSITGTYDLK |  |
| 12 | P10909 | Clusterin OS=Homo sapiens OX=9606 GN=CLU PE=1 SV=1 | 111 | 53031 | 2 | 645,2155 | 2 | 0,7857 | 58,44 | ELDESLQVAER |  |
|  |  |  |  |  |  | 882,114 | 2 | 0,3932 | 37,44 | EILSVDCSTNNPSQAK |  |
| 13 | A0M8Q6 | Immunoglobulin lambda constant 7 OS=Homo sapiens OX=9606 GN=IGLC7 PE=1 SV=3 | 106 | 11418 | 2 | 872,5646 | 2 | 0,2631 | 72,82 | YAASSYLSLTPEQWK |  |
|  |  |  |  |  |  | 993,6497 | 2 | 0,2742 | 60,02 | AAPSVTLFPPSSEELQANK |  |
| 14 | P0DOX2 | Immunoglobulin alpha-2 heavy chain OS=Homo sapiens OX=9606 PE=1 SV=2 | 106 | 49816 | 3 | 448,871 | 2 | 0,2723 | 37,3 | YLTWASR |  |
|  |  |  |  |  |  | 607,5021 | 2 | 0,3645 | 42,56 | WLQGSQELPR |  |
|  |  |  |  |  |  | 660,1824 | 2 | 0,7844 | 63,02 | AEDTAVYYCAR |  |
| 15 | P0DOX6 | Immunoglobulin mu heavy chain OS=Homo sapiens OX=9606 PE=1 SV=2 | 76 | 64244 | 2 | 639,4846 | 2 | 0,252 | 78,3 | YAATSQVLLPSK |  |
|  |  |  |  |  |  | 809,2263 | 2 | 0,6715 | 27,73 | YVTSAPMPEPQAPGR | Oxidation (M) |
| 16 | P14923 | Junction plakoglobin OS=Homo sapiens OX=9606 GN=JUP PE=1 SV=3 | 75 | 82434 | 2 | 544,6852 | 2 | 0,7122 | 45,78 | LNYGIPAIVK |  |
|  |  |  |  |  |  | 714,8408 | 2 | 0,8705 | 60,41 | ALMGSPQLVAAVVR | Oxidation (M) |
| 17 | P00751-2 | Isoform 2 of Complement factor B OS=Homo sapiens OX=9606 GN=CFB | 71 | 69969 | 2 | 368,4015 | 2 | 0,3961 | 21,96 | SGTNTKK |  |
|  |  |  |  |  |  | 638,3567 | 2 | 0,0442 | 70,57 | YGLVTYATYPK |  |
| 18 | P02790 | Hemopexin OS=Homo sapiens OX=9606 GN=HPX PE=1 SV=2 | 65 | 52385 | 2 | 571,5764 | 2 | 0,5566 | 65,13 | GGYTLVSGYPK |  |
|  |  |  |  |  |  | 579,6063 | 2 | -0,2653 | 20,86 | DYFMPCPGR | Oxidation (M) |
| 19 | Q96P63 | Serpin B12 OS=Homo sapiens OX=9606 GN=SERPINB12 PE=1 SV=1 | 50 | 46646 | 2 | 838,7727 | 2 | 0,648 | 48,46 | ADLTGISPSPNLYLSK |  |
|  |  |  |  |  |  | 1052,755 | 2 | 0,3648 | 22,07 | HKNIFFSPLSLSAALGMVR | Oxidation (M) |

**Table S2.10** PEG-MWCNTs-mDAAO Soft Corona R2

| **Prot. Number** | **Accession Number** | **Protein Name** | | **Mascot Score** | **Mr** | **N° pep** | **m/z** | **z** | **Pep. error** | **Pep. Score** | **Pep. sequence** | **Pep. Modification** |
| --- | --- | --- | --- | --- | --- | --- | --- | --- | --- | --- | --- | --- |
| 1 | P02768 | | Serum albumin OS=Homo sapiens OX=9606 GN=ALB PE=1 SV=2 | 5172 | 71317 | 29 | 772,2756 | 1 | -0,1629 | 23,39 | AACLLPK |  |
|  |  | |  |  |  |  | 789,3745 | 1 | -0,0971 | 29,88 | LVTDLTK |  |
|  |  | |  |  |  |  | 880,2279 | 1 | -0,2131 | 41,74 | AEFAEVSK |  |
|  |  | |  |  |  |  | 463,8422 | 2 | -0,8162 | 44,7 | YLYEIAR |  |
|  |  | |  |  |  |  | 467,2629 | 2 | -0,0001 | 47,57 | LCTVATLR |  |
|  |  | |  |  |  |  | 470,5692 | 2 | -0,3172 | 26,33 | DDNPNLPR |  |
|  |  | |  |  |  |  | 480,4237 | 2 | -0,7223 | 26,73 | FQNALLVR |  |
|  |  | |  |  |  |  | 984,3238 | 1 | -0,1646 | 24,5 | TYETTLEK |  |
|  |  | |  |  |  |  | 500,7168 | 2 | -0,1773 | 61,88 | QTALVELVK |  |
|  |  | |  |  |  |  | 1013,175 | 1 | -0,4242 | 31,28 | LVAASQAALGL |  |
|  |  | |  |  |  |  | 509,2164 | 2 | -0,1108 | 31,4 | SLHTLFGDK |  |
|  |  | |  |  |  |  | 538,0317 | 2 | 0,5135 | 28,07 | LDELRDEGK |  |
|  |  | |  |  |  |  | 564,8404 | 2 | -0,0252 | 59,83 | KQTALVELVK |  |
|  |  | |  |  |  |  | 569,5344 | 2 | -0,4364 | 32,39 | CCTESLVNR |  |
|  |  | |  |  |  |  | 571,1374 | 2 | -0,4263 | 64,78 | KLVAASQAALGL |  |
|  |  | |  |  |  |  | 1149,138 | 1 | -0,4768 | 29,04 | LVNEVTEFAK |  |
|  |  | |  |  |  |  | 409,5199 | 3 | -0,0601 | 20,81 | FKDLGEENFK |  |
|  |  | |  |  |  |  | 679,4536 | 2 | -0,7297 | 62,01 | AVMDDFAAFVEK | Oxidation (M) |
|  |  | |  |  |  |  | 686,1346 | 2 | -0,3048 | 64,68 | AAFTECCQAADK |  |
|  |  | |  |  |  |  | 722,0789 | 2 | -0,4916 | 63,31 | YICENQDSISSK |  |
|  |  | |  |  |  |  | 725,765 | 2 | -0,0057 | 45,58 | ETYGEMADCCAK | Oxidation (M) |
|  |  | |  |  |  |  | 749,655 | 2 | -0,2757 | 89,61 | TCVADESAENCDK |  |
|  |  | |  |  |  |  | 756,2754 | 2 | -0,2993 | 70,68 | VPQVSTPTLVEVSR |  |
|  |  | |  |  |  |  | 776,5388 | 2 | -0,5274 | 23,7 | CCAAADPHECYAK |  |
|  |  | |  |  |  |  | 811,9702 | 2 | -0,8544 | 24,02 | DVFLGMFLYEYAR |  |
|  |  | |  |  |  |  | 820,2364 | 2 | -0,4721 | 60,34 | KVPQVSTPTLVEVSR |  |
|  |  | |  |  |  |  | 820,3115 | 2 | -0,1667 | 49,22 | DVFLGMFLYEYAR | Oxidation (M) |
|  |  | |  |  |  |  | 547,2374 | 3 | -0,2402 | 34,69 | KVPQVSTPTLVEVSR |  |
|  |  | |  |  |  |  | 828,9301 | 2 | -0,8997 | 23,74 | QNCELFEQLGEYK |  |
|  |  | |  |  |  |  | 637,5944 | 3 | -0,1631 | 22,67 | RPCFSALEVDETYVPK |  |
|  |  | |  |  |  |  | 1023,042 | 2 | -0,0181 | 75,71 | VFDEFKPLVEEPQNLIK |  |
| 2 | P0DOX5 | | Immunoglobulin gamma-1 heavy chain OS=Homo sapiens OX=9606 PE=1 SV=2 | 1474 | 49925 | 9 | 851,3586 | 1 | -0,0705 | 21,63 | DTLMISR | Oxidation (M) |
|  |  | |  |  |  |  | 581,169 | 2 | -0,299 | 45,1 | NQVSLTCLVK |  |
|  |  | |  |  |  |  | 593,627 | 2 | -0,3999 | 33,9 | GPSVFPLAPSSK |  |
|  |  | |  |  |  |  | 643,6361 | 2 | -0,409 | 30,01 | EPQVYTLPPSR |  |
|  |  | |  |  |  |  | 661,3353 | 2 | -0,0147 | 79,91 | STSGGTAALGCLVK |  |
|  |  | |  |  |  |  | 937,2404 | 2 | -0,4484 | 50,5 | TTPPVLDSDGSFFLYSK |  |
|  |  | |  |  |  |  | 625,1744 | 3 | 0,5384 | 25,25 | EPQVYTLPPSRDELTK |  |
|  |  | |  |  |  |  | 937,2711 | 2 | -0,3869 | 45,55 | TTPPVLDSDGSFFLYSK |  |
|  |  | |  |  |  |  | 982,8507 | 2 | -0,4047 | 20,26 | QVQLVQSGGGVVQPGRSLR |  |
|  |  | |  |  |  |  | 713,9055 | 3 | 0,6744 | 45,95 | TPEVTCVVVDVSHEDPEVK |  |
| 3 | P02787 | | Serotransferrin OS=Homo sapiens OX=9606 GN=TF PE=1 SV=3 | 1077 | 79294 | 18 | 735,3124 | 1 | -0,0911 | 26,22 | GDVAFVK |  |
|  |  | |  |  |  |  | 437,703 | 2 | -0,043 | 35,35 | DSAHGFLK |  |
|  |  | |  |  |  |  | 461,4876 | 2 | 0,5334 | 38,96 | DDTVCLAK |  |
|  |  | |  |  |  |  | 483,0483 | 2 | 0,5569 | 64,44 | APNHAVVTR |  |
|  |  | |  |  |  |  | 489,7667 | 2 | 0,0371 | 80,15 | DGAGDVAFVK |  |
|  |  | |  |  |  |  | 499,0427 | 2 | -0,3991 | 38,77 | ASYLDCIR |  |
|  |  | |  |  |  |  | 500,778 | 2 | 0,0501 | 28,49 | YLGEEYVK |  |
|  |  | |  |  |  |  | 584,054 | 2 | 0,5095 | 30,68 | HQTVPQNTGGK |  |
|  |  | |  |  |  |  | 606,2681 | 2 | -0,0185 | 50,19 | DSGFQMNQLR | Oxidation (M) |
|  |  | |  |  |  |  | 637,5616 | 2 | 0,4625 | 41,49 | HSTIFENLANK |  |
|  |  | |  |  |  |  | 642,0022 | 2 | -0,572 | 59,65 | EGYYGYTGAFR |  |
|  |  | |  |  |  |  | 708,2428 | 2 | -0,2416 | 51,58 | SVIPSDGPSVACVK |  |
|  |  | |  |  |  |  | 747,8967 | 2 | 0,0565 | 76,95 | MYLGYEYVTAIR | Oxidation (M) |
|  |  | |  |  |  |  | 766,3201 | 2 | -0,055 | 31,59 | CSTSSLLEACTFR |  |
|  |  | |  |  |  |  | 789,3741 | 2 | 0,0833 | 69,26 | FDEFFSEGCAPGSK |  |
|  |  | |  |  |  |  | 815,324 | 2 | -0,1752 | 58,37 | EDPQTFYYAVAVVK |  |
|  |  | |  |  |  |  | 863,4108 | 2 | 0,0473 | 98,51 | IECVSAETTEDCIAK |  |
|  |  | |  |  |  |  | 1096,161 | 2 | 0,3106 | 113,26 | IMNGEADAMSLDGGFVYIAGK | 2 Oxidation (M) |
| 4 | P01834 | | Immunoglobulin kappa constant OS=Homo sapiens OX=9606 GN=IGKC PE=1 SV=2 | 1020 | 11929 | 3 | 751,9272 | 2 | 0,0888 | 33,96 | DSTYSLSSTLTLSK |  |
|  |  | |  |  |  |  | 973,2133 | 2 | -0,6077 | 51,94 | TVAAPSVFIFPPSDEQLK |  |
|  |  | |  |  |  |  | 1068,253 | 2 | -0,4706 | 45,69 | VDNALQSGNSQESVTEQDSK |  |
| 5 | P01859 | | Immunoglobulin heavy constant gamma 2 OS=Homo sapiens OX=9606 GN=IGHG2 PE=1 SV=2 | 488 | 36505 | 5 | 851,3586 | 1 | -0,0705 | 21,63 | DTLMISR | Oxidation (M) |
|  |  | |  |  |  |  | 581,169 | 2 | -0,299 | 45,1 | NQVSLTCLVK |  |
|  |  | |  |  |  |  | 643,6361 | 2 | -0,409 | 30,01 | EPQVYTLPPSR |  |
|  |  | |  |  |  |  | 712,2809 | 2 | -0,1552 | 64,72 | STSESTAALGCLVK |  |
|  |  | |  |  |  |  | 961,3157 | 2 | -0,2648 | 64,65 | TTPPMLDSDGSFFLYSK | Oxidation (M) |
| 6 | P01861 | | Immunoglobulin heavy constant gamma 4 OS=Homo sapiens OX=9606 GN=IGHG4 PE=1 SV=1 | 393 | 36431 | 4 | 851,3586 | 1 | -0,0705 | 21,63 | DTLMISR | Oxidation (M) |
|  |  | |  |  |  |  | 581,169 | 2 | -0,299 | 45,1 | NQVSLTCLVK |  |
|  |  | |  |  |  |  | 712,2809 | 2 | -0,1552 | 64,72 | STSESTAALGCLVK |  |
|  |  | |  |  |  |  | 951,6089 | 2 | 0,2825 | 77,75 | TTPPVLDSDGSFFLYSR |  |
| 7 | P04114 | | Apolipoprotein B-100 OS=Homo sapiens OX=9606 GN=APOB PE=1 SV=2 | 281 | 516651 | 13 | 507,087 | 2 | 0,5267 | 36,88 | TGISPLALIK |  |
|  |  | |  |  |  |  | 508,198 | 2 | -0,1572 | 23,59 | VSTAFVYTK |  |
|  |  | |  |  |  |  | 514,923 | 2 | 0,2289 | 46,72 | LSNVLQQVK |  |
|  |  | |  |  |  |  | 523,5905 | 2 | 0,5696 | 38,26 | IPSVQINFK |  |
|  |  | |  |  |  |  | 598,8834 | 2 | 0,1768 | 48,96 | NMEVSVATTTK | Oxidation (M) |
|  |  | |  |  |  |  | 622,2871 | 2 | 0,9305 | 36,46 | ATGVLYDYVNK |  |
|  |  | |  |  |  |  | 655,6101 | 2 | 0,5131 | 57,84 | EVGTVLSQVYSK |  |
|  |  | |  |  |  |  | 678,5648 | 2 | -0,6915 | 58,35 | LPYTIITTPPLK |  |
|  |  | |  |  |  |  | 680,7672 | 2 | -0,1795 | 63,01 | INNQLTLDSNTK |  |
|  |  | |  |  |  |  | 694,033 | 2 | 0,2748 | 66,42 | IAELSATAQEIIK |  |
|  |  | |  |  |  |  | 791,652 | 2 | 0,5026 | 80,53 | AVSMPSFSILGSDVR | Oxidation (M) |
|  |  | |  |  |  |  | 808,0297 | 2 | 0,3271 | 20,01 | MYQMDIQQELQR | 2 Oxidation (M) |
|  |  | |  |  |  |  | 829,5208 | 2 | 0,1588 | 79,3 | SVSDGIAALDLNAVANK |  |
| 8 | P01009 | | Alpha-1-antitrypsin OS=Homo sapiens OX=9606 GN=SERPINA1 PE=1 SV=3 | 250 | 46878 | 4 | 444,9769 | 2 | 0,4428 | 22,32 | AVLTIDEK |  |
|  |  | |  |  |  |  | 555,3987 | 2 | -0,814 | 31,29 | LSITGTYDLK |  |
|  |  | |  |  |  |  | 917,3415 | 2 | -0,2471 | 112,68 | VFSNGADLSGVTEEAPLK |  |
|  |  | |  |  |  |  | 1146,087 | 2 | 0,0378 | 88 | GTEAAGAMFLEAIPMSIPPEVK | 2 Oxidation (M) |
| 9 | P01024 | | Complement C3 OS=Homo sapiens OX=9606 GN=C3 PE=1 SV=2 | 224 | 188569 | 8 | 502,2643 | 2 | 0,9746 | 23,14 | TGLQEVEVK |  |
|  |  | |  |  |  |  | 612,7614 | 2 | -0,0813 | 39,88 | YYTYLIMNK | Oxidation (M) |
|  |  | |  |  |  |  | 645,3149 | 2 | 0,0146 | 65,18 | SGSDEVQVGQQR |  |
|  |  | |  |  |  |  | 650,6446 | 2 | -0,3058 | 24,99 | ACEPGVDYVYK |  |
|  |  | |  |  |  |  | 701,0764 | 2 | -0,6896 | 33,36 | SSLSVPYVIVPLK |  |
|  |  | |  |  |  |  | 735,5068 | 2 | -0,7734 | 57,91 | IPIEDGSGEVVLSR |  |
|  |  | |  |  |  |  | 756,7026 | 2 | 0,5764 | 74,45 | LVAYYTLIGASGQR |  |
|  |  | |  |  |  |  | 828,1565 | 2 | -0,5616 | 36,13 | TVMVNIENPEGIPVK | Oxidation (M) |
| 10 | P00738 | | Haptoglobin OS=Homo sapiens OX=9606 GN=HP PE=1 SV=1 | 200 | 45861 | 2 | 645,8275 | 2 | -0,0826 | 34,62 | DIAPTLTLYVGK |  |
|  |  | |  |  |  |  | 673,0917 | 2 | -0,4696 | 69,85 | SCAVAEYGVYVK |  |
| 11 | P02647 | | Apolipoprotein A-I OS=Homo sapiens OX=9606 GN=APOA1 PE=1 SV=1 | 191 | 30759 | 12 | 391,1475 | 2 | -0,1437 | 25,79 | AHVDALR |  |
|  |  | |  |  |  |  | 448,8531 | 2 | 0,2153 | 29,56 | LHELQEK |  |
|  |  | |  |  |  |  | 506,6975 | 2 | -0,1909 | 22,94 | AKPALEDLR |  |
|  |  | |  |  |  |  | 524,2562 | 2 | -0,0087 | 26,9 | LSPLGEEMR | Oxidation (M) |
|  |  | |  |  |  |  | 578,98 | 2 | -0,6745 | 27,07 | LEALKENGGAR |  |
|  |  | |  |  |  |  | 608,1817 | 2 | -0,2654 | 39,1 | ATEHLSTLSEK |  |
|  |  | |  |  |  |  | 618,3108 | 2 | -0,0739 | 61,26 | DLATVYVDVLK |  |
|  |  | |  |  |  |  | 434,4628 | 3 | -0,2747 | 41,75 | THLAPYSDELR |  |
|  |  | |  |  |  |  | 668,0916 | 2 | -0,4701 | 20,56 | QEMSKDLEEVK |  |
|  |  | |  |  |  |  | 690,8719 | 2 | 0,0208 | 29,12 | VQPYLDDFQKK |  |
|  |  | |  |  |  |  | 693,7024 | 2 | -0,3176 | 52,55 | VSFLSALEEYTK |  |
|  |  | |  |  |  |  | 606,1138 | 3 | 0,476 | 21,87 | DSGRDYVSQFEGSALGK |  |
| 12 | P01023 | | Alpha-2-macroglobulin OS=Homo sapiens OX=9606 GN=A2M PE=1 SV=3 | 170 | 164613 | 7 | 509,6364 | 2 | -0,3277 | 46,39 | ATVLNYLPK |  |
|  |  | |  |  |  |  | 552,1755 | 2 | -0,2617 | 42,48 | SSGSLLNNAIK |  |
|  |  | |  |  |  |  | 575,643 | 2 | -0,2985 | 62,64 | SASNMAIVDVK | Oxidation (M) |
|  |  | |  |  |  |  | 605,7292 | 2 | -0,1914 | 49,65 | LPPNVVEESAR |  |
|  |  | |  |  |  |  | 628,3887 | 2 | 0,1271 | 53,31 | AIGYLNTGYQR |  |
|  |  | |  |  |  |  | 636,8268 | 2 | -0,0265 | 42,83 | VTAAPQSVCALR |  |
|  |  | |  |  |  |  | 741,0652 | 2 | 0,4897 | 42,21 | DMYSFLEDMGLK | 2 Oxidation (M) |
| 13 | P01876 | | Immunoglobulin heavy constant alpha 1 OS=Homo sapiens OX=9606 GN=IGHA1 PE=1 SV=2 | 159 | 38486 | 3 | 466,2276 | 2 | -0,0979 | 22,44 | TPLTATLSK |  |
|  |  | |  |  |  |  | 688,4525 | 2 | 0,2779 | 55,39 | TFTCTAAYPESK |  |
|  |  | |  |  |  |  | 918,6446 | 2 | 0,3322 | 63,04 | QEPSQGTTTFAVTSILR |  |
| 14 | P01011 | | Alpha-1-antichymotrypsin OS=Homo sapiens OX=9606 GN=SERPINA3 PE=1 SV=2 | 79 | 47792 | 2 | 531,4692 | 2 | 0,3435 | 29,76 | EIGELYLPK |  |
|  |  | |  |  |  |  | 608,4548 | 2 | 0,1717 | 78,55 | ITLLSALVETR |  |

**Table S2.11** PEG-MWCNTs-mDAAO Hard Corona R1

| **Prot. Number** | **Accession Number** | **Protein Name** | | **Mascot Score** | **Mr** | **N° pep** | **m/z** | **z** | **Pep. error** | **Pep. Score** | **Pep. sequence** | **Pep. Modification** |
| --- | --- | --- | --- | --- | --- | --- | --- | --- | --- | --- | --- | --- |
| 1 | P02768 | | Serum albumin OS=Homo sapiens OX=9606 GN=ALB PE=1 SV=2 | 5817 | 71317 | 31 | 673,4067 | 1 | 0,0287 | 28,38 | AWAVAR |  |
|  |  | |  |  |  |  | 772,4061 | 1 | -0,0324 | 23,55 | AACLLPK |  |
|  |  | |  |  |  |  | 395,4724 | 2 | 0,4658 | 37,3 | LVTDLTK |  |
|  |  | |  |  |  |  | 440,6285 | 2 | -0,1914 | 25,43 | AEFAEVSK |  |
|  |  | |  |  |  |  | 464,2584 | 2 | 0,0161 | 41,24 | YLYEIAR |  |
|  |  | |  |  |  |  | 467,3152 | 2 | 0,1044 | 37,63 | LCTVATLR |  |
|  |  | |  |  |  |  | 470,6526 | 2 | -0,1502 | 38,04 | DDNPNLPR |  |
|  |  | |  |  |  |  | 476,2713 | 2 | 0,0936 | 38,52 | DLGEENFK |  |
|  |  | |  |  |  |  | 481,1008 | 2 | 0,6318 | 45,14 | FQNALLVR |  |
|  |  | |  |  |  |  | 984,3877 | 1 | -0,1007 | 23,19 | TYETTLEK |  |
|  |  | |  |  |  |  | 500,618 | 2 | -0,3749 | 39,92 | QTALVELVK |  |
|  |  | |  |  |  |  | 1013,264 | 1 | -0,3353 | 37,9 | LVAASQAALGL |  |
|  |  | |  |  |  |  | 509,4008 | 2 | 0,2579 | 45,39 | SLHTLFGDK |  |
|  |  | |  |  |  |  | 537,8801 | 2 | 0,2104 | 33,08 | LDELRDEGK |  |
|  |  | |  |  |  |  | 564,9556 | 2 | 0,2052 | 52,21 | KQTALVELVK |  |
|  |  | |  |  |  |  | 569,8748 | 2 | 0,2443 | 51,75 | CCTESLVNR |  |
|  |  | |  |  |  |  | 571,3269 | 2 | -0,0474 | 44,85 | KLVAASQAALGL |  |
|  |  | |  |  |  |  | 1149,52 | 1 | -0,0952 | 32,26 | LVNEVTEFAK |  |
|  |  | |  |  |  |  | 613,9015 | 2 | 0,1905 | 41,56 | FKDLGEENFK |  |
|  |  | |  |  |  |  | 679,7248 | 2 | -0,1873 | 77,55 | AVMDDFAAFVEK | Oxidation (M) |
|  |  | |  |  |  |  | 686,1287 | 2 | -0,3166 | 64,2 | AAFTECCQAADK |  |
|  |  | |  |  |  |  | 717,7093 | 2 | -0,122 | 39,21 | ETYGEMADCCAK |  |
|  |  | |  |  |  |  | 722,3265 | 2 | 0,0038 | 65,69 | YICENQDSISSK |  |
|  |  | |  |  |  |  | 726,0277 | 2 | 0,5198 | 50,77 | ETYGEMADCCAK | Oxidation (M) |
|  |  | |  |  |  |  | 749,7013 | 2 | -0,1831 | 110,51 | TCVADESAENCDK |  |
|  |  | |  |  |  |  | 500,8358 | 3 | 0,8611 | 46,8 | ADDKETCFAEEGK |  |
|  |  | |  |  |  |  | 756,3882 | 2 | -0,0736 | 20,01 | VPQVSTPTLVEVSR |  |
|  |  | |  |  |  |  | 820,4667 | 2 | 0,1436 | 65,73 | DVFLGMFLYEYAR | Oxidation (M) |
|  |  | |  |  |  |  | 547,4141 | 3 | 0,2899 | 51,96 | KVPQVSTPTLVEVSR |  |
|  |  | |  |  |  |  | 828,9237 | 2 | -0,9125 | 39,21 | QNCELFEQLGEYK |  |
|  |  | |  |  |  |  | 637,8908 | 3 | 0,726 | 47,23 | RPCFSALEVDETYVPK |  |
|  |  | |  |  |  |  | 997,6602 | 3 | 0,6266 | 58,97 | SHCIAEVENDEMPADLPSLAADFVESK | Oxidation (M) |
| 2 | P0DOX5 | | Immunoglobulin gamma-1 heavy chain OS=Homo sapiens OX=9606 PE=1 SV=2 | 1517 | 49925 | 7 | 419,7391 | 2 | -0,0322 | 20,66 | ALPAPIEK |  |
|  |  | |  |  |  |  | 425,9138 | 2 | -0,6089 | 25,33 | DTLMISR | Oxidation (M) |
|  |  | |  |  |  |  | 581,3823 | 2 | 0,1277 | 40,47 | NQVSLTCLVK |  |
|  |  | |  |  |  |  | 593,8695 | 2 | 0,0851 | 37,26 | GPSVFPLAPSSK |  |
|  |  | |  |  |  |  | 643,816 | 2 | -0,0492 | 62,37 | EPQVYTLPPSR |  |
|  |  | |  |  |  |  | 661,4204 | 2 | 0,1555 | 87,99 | STSGGTAALGCLVK |  |
|  |  | |  |  |  |  | 937,613 | 2 | 0,297 | 66,71 | TTPPVLDSDGSFFLYSK |  |
| 3 | P01024 | | Complement C3 OS=Homo sapiens OX=9606 GN=C3 PE=1 SV=2 | 1013 | 188569 | 25 | 389,0012 | 2 | 0,5285 | 39,28 | GVFVLNK |  |
|  |  | |  |  |  |  | 480,3794 | 2 | 0,2108 | 34,47 | EALKLEEK |  |
|  |  | |  |  |  |  | 513,1065 | 2 | 0,6924 | 44,46 | FISLGEACK |  |
|  |  | |  |  |  |  | 532,5293 | 2 | 0,5061 | 36,85 | DSCVGSLVVK |  |
|  |  | |  |  |  |  | 542,5377 | 2 | 0,51 | 53,71 | GYTQQLAFR |  |
|  |  | |  |  |  |  | 555,965 | 2 | 0,2962 | 52,82 | VLLDGVQNPR |  |
|  |  | |  |  |  |  | 570,1388 | 2 | -0,2705 | 21,94 | FYYIYNEK |  |
|  |  | |  |  |  |  | 577,1183 | 2 | 0,6246 | 33,01 | QPSSAFAAFVK |  |
|  |  | |  |  |  |  | 596,6392 | 2 | 0,6859 | 39,26 | SDDKVTLEER |  |
|  |  | |  |  |  |  | 405,0718 | 3 | 0,5174 | 20,17 | VTIKPAPETEK |  |
|  |  | |  |  |  |  | 621,8585 | 2 | 0,0587 | 36,45 | QPVPGQQMTLK | Oxidation (M) |
|  |  | |  |  |  |  | 641,6072 | 2 | 0,6114 | 59,42 | ENEGFTVTAEGK |  |
|  |  | |  |  |  |  | 645,4534 | 2 | 0,2914 | 50,47 | SGSDEVQVGQQR |  |
|  |  | |  |  |  |  | 668,6606 | 2 | 0,5833 | 64,23 | APSTWLTAYVVK |  |
|  |  | |  |  |  |  | 685,8769 | 2 | 0,0152 | 52,61 | TIYTPGSTVLYR |  |
|  |  | |  |  |  |  | 470,0251 | 3 | 0,4398 | 31,26 | VSHSEDDCLAFK |  |
|  |  | |  |  |  |  | 735,7664 | 2 | -0,2543 | 46,74 | IPIEDGSGEVVLSR |  |
|  |  | |  |  |  |  | 756,3882 | 2 | -0,0524 | 117,18 | LVAYYTLIGASGQR |  |
|  |  | |  |  |  |  | 828,5757 | 2 | 0,2768 | 72,6 | TVMVNIENPEGIPVK | Oxidation (M) |
|  |  | |  |  |  |  | 829,421 | 2 | 0,0709 | 23,02 | AGDFLEANYMNLQR | Oxidation (M) |
|  |  | |  |  |  |  | 844,8716 | 2 | 0,8887 | 61 | SYTVAIAGYALAQMGR | Oxidation (M) |
|  |  | |  |  |  |  | 1087,01 | 2 | -0,0678 | 135,31 | ILLQGTPVAQMTEDAVDAER | Oxidation (M) |
|  |  | |  |  |  |  | 1099,334 | 2 | -0,4688 | 144,42 | VPVAVQGEDTVQSLTQGDGVAK |  |
|  |  | |  |  |  |  | 1107,674 | 2 | 0,3362 | 96,68 | EDIPPADLSDQVPDTESETR |  |
|  |  | |  |  |  |  | 919,3605 | 3 | 0,7764 | 44,01 | EGVQKEDIPPADLSDQVPDTESETR |  |
| 4 | P02787 | | Serotransferrin OS=Homo sapiens OX=9606 GN=TF PE=1 SV=3 | 856 | 79294 | 11 | 489,7439 | 2 | -0,0085 | 79,95 | DGAGDVAFVK |  |
|  |  | |  |  |  |  | 501,0218 | 2 | 0,5377 | 25,78 | YLGEEYVK |  |
|  |  | |  |  |  |  | 606,4866 | 2 | 0,4185 | 48,08 | DSGFQMNQLR | Oxidation (M) |
|  |  | |  |  |  |  | 625,599 | 2 | 0,5848 | 66,95 | SASDLTWDNLK |  |
|  |  | |  |  |  |  | 642,2093 | 2 | -0,1577 | 45,44 | EGYYGYTGAFR |  |
|  |  | |  |  |  |  | 708,5159 | 2 | 0,3046 | 47,84 | SVIPSDGPSVACVK |  |
|  |  | |  |  |  |  | 747,9739 | 2 | 0,2108 | 68,88 | MYLGYEYVTAIR | Oxidation (M) |
|  |  | |  |  |  |  | 766,8217 | 2 | 0,9481 | 94,8 | CSTSSLLEACTFR |  |
|  |  | |  |  |  |  | 815,3889 | 2 | -0,0455 | 91,19 | EDPQTFYYAVAVVK |  |
|  |  | |  |  |  |  | 862,224 | 2 | 0,68 | 64,76 | LCMGSGLNLCEPNNK | Oxidation (M) |
|  |  | |  |  |  |  | 1096,353 | 2 | 0,6949 | 98,09 | IMNGEADAMSLDGGFVYIAGK | 2 Oxidation (M) |
| 5 | P01860 | | Immunoglobulin heavy constant gamma 3 OS=Homo sapiens OX=9606 GN=IGHG3 PE=1 SV=2 | 791 | 42287 | 6 | 419,7391 | 2 | -0,0322 | 20,66 | ALPAPIEK |  |
|  |  | |  |  |  |  | 425,9138 | 2 | -0,6089 | 25,33 | DTLMISR | Oxidation (M) |
|  |  | |  |  |  |  | 581,3823 | 2 | 0,1277 | 40,47 | NQVSLTCLVK |  |
|  |  | |  |  |  |  | 643,816 | 2 | -0,0492 | 62,37 | EPQVYTLPPSR |  |
|  |  | |  |  |  |  | 644,2135 | 2 | -0,2317 | 35,2 | GPSVFPLAPCSR |  |
|  |  | |  |  |  |  | 644,2743 | 2 | 0,8674 | 23,94 | EPQVYTLPPSR |  |
|  |  | |  |  |  |  | 644,624 | 2 | 0,5893 | 49,37 | GPSVFPLAPCSR |  |
|  |  | |  |  |  |  | 661,4204 | 2 | 0,1555 | 87,99 | STSGGTAALGCLVK |  |
| 6 | P04114 | | Apolipoprotein B-100 OS=Homo sapiens OX=9606 GN=APOB PE=1 SV=2 | 751 | 516651 | 26 | 388,5968 | 2 | -0,2472 | 21,17 | QLKEMK |  |
|  |  | |  |  |  |  | 473,1304 | 2 | 0,7124 | 36,28 | LTLDIQNK |  |
|  |  | |  |  |  |  | 481,4105 | 2 | 0,2824 | 56,26 | LDVTTSIGR |  |
|  |  | |  |  |  |  | 500,4512 | 2 | 0,4136 | 40,87 | MGLAFESTK | Oxidation (M) |
|  |  | |  |  |  |  | 507,2431 | 2 | 0,8388 | 37,69 | TGISPLALIK |  |
|  |  | |  |  |  |  | 508,9477 | 2 | -0,7056 | 31,4 | LATALSLSNK |  |
|  |  | |  |  |  |  | 515,1564 | 2 | 0,6957 | 52,47 | LSNVLQQVK |  |
|  |  | |  |  |  |  | 1039,408 | 1 | -0,2318 | 34,39 | LAPGELTIIL |  |
|  |  | |  |  |  |  | 570,5121 | 2 | 0,4801 | 20,35 | EVYGFNPEGK |  |
|  |  | |  |  |  |  | 599,0016 | 2 | 0,4132 | 53,97 | NMEVSVATTTK | Oxidation (M) |
|  |  | |  |  |  |  | 601,6517 | 2 | 0,6427 | 63,16 | LTISEQNIQR |  |
|  |  | |  |  |  |  | 614,9917 | 2 | 0,4499 | 54,9 | NSEEFAAAMSR | Oxidation (M) |
|  |  | |  |  |  |  | 618,5443 | 2 | 0,4799 | 69,75 | ENFAGEATLQR |  |
|  |  | |  |  |  |  | 634,7733 | 2 | -0,1815 | 29,54 | DLKVEDIPLAR |  |
|  |  | |  |  |  |  | 636,6879 | 2 | 0,6843 | 68,97 | SVSLPSLDPASAK |  |
|  |  | |  |  |  |  | 640,9717 | 2 | 0,2152 | 39,49 | TEVIPPLIENR |  |
|  |  | |  |  |  |  | 670,0566 | 2 | 0,4702 | 35,03 | ESQLPTVMDFR | Oxidation (M) |
|  |  | |  |  |  |  | 678,0656 | 2 | 0,4204 | 75,69 | YGMVAQVTQTLK | Oxidation (M) |
|  |  | |  |  |  |  | 681,015 | 2 | 0,4212 | 34,22 | SEYQADYESLR |  |
|  |  | |  |  |  |  | 722,9745 | 2 | 0,2606 | 34,14 | LQSTTVMNPYMK | 2 Oxidation (M) |
|  |  | |  |  |  |  | 753,4277 | 2 | 0,0676 | 50,25 | IGQDGISTSATTNLK |  |
|  |  | |  |  |  |  | 791,6827 | 2 | 0,564 | 79,02 | AVSMPSFSILGSDVR | Oxidation (M) |
|  |  | |  |  |  |  | 808,0438 | 2 | 0,3552 | 44,88 | MYQMDIQQELQR | 2 Oxidation (M) |
|  |  | |  |  |  |  | 815,6962 | 2 | 0,6387 | 88,11 | MTSNFPVDLSDYPK | Oxidation (M) |
|  |  | |  |  |  |  | 829,6576 | 2 | 0,4324 | 20,68 | SVSDGIAALDLNAVANK |  |
|  |  | |  |  |  |  | 961,1647 | 2 | 0,3815 | 32,03 | VIGNMGQTMEQLTPELK | 2 Oxidation (M) |
| 7 | P01859 | | Immunoglobulin heavy constant gamma 2 OS=Homo sapiens OX=9606 GN=IGHG2 PE=1 SV=2 | 659 | 36505 | 6 | 425,9138 | 2 | -0,6089 | 25,33 | DTLMISR | Oxidation (M) |
|  |  | |  |  |  |  | 581,3823 | 2 | 0,1277 | 40,47 | NQVSLTCLVK |  |
|  |  | |  |  |  |  | 643,816 | 2 | -0,0492 | 62,37 | EPQVYTLPPSR |  |
|  |  | |  |  |  |  | 644,2135 | 2 | -0,2317 | 35,2 | GPSVFPLAPCSR |  |
|  |  | |  |  |  |  | 644,2743 | 2 | 0,8674 | 23,94 | EPQVYTLPPSR |  |
|  |  | |  |  |  |  | 644,624 | 2 | 0,5893 | 49,37 | GPSVFPLAPCSR |  |
|  |  | |  |  |  |  | 712,0803 | 2 | -0,5564 | 40,9 | STSESTAALGCLVK |  |
|  |  | |  |  |  |  | 961,5835 | 2 | 0,2709 | 35,67 | TTPPMLDSDGSFFLYSK | Oxidation (M) |
| 8 | P01023 | | Alpha-2-macroglobulin OS=Homo sapiens OX=9606 GN=A2M PE=1 SV=3 | 583 | 164613 | 14 | 414,7704 | 2 | 0,0762 | 32,93 | SDIAPVAR |  |
|  |  | |  |  |  |  | 509,7856 | 2 | -0,0293 | 44,2 | ATVLNYLPK |  |
|  |  | |  |  |  |  | 524,1583 | 2 | 0,7213 | 54,79 | FEVQVTVPK |  |
|  |  | |  |  |  |  | 552,2557 | 2 | -0,1013 | 45,52 | SSGSLLNNAIK |  |
|  |  | |  |  |  |  | 558,8612 | 2 | 0,1103 | 33,41 | QTVSWAVTPK |  |
|  |  | |  |  |  |  | 575,9995 | 2 | 0,4145 | 33,56 | SASNMAIVDVK | Oxidation (M) |
|  |  | |  |  |  |  | 606,0814 | 2 | 0,513 | 57,76 | LPPNVVEESAR |  |
|  |  | |  |  |  |  | 628,5754 | 2 | 0,5007 | 38,31 | AIGYLNTGYQR |  |
|  |  | |  |  |  |  | 636,8286 | 2 | -0,0229 | 60,73 | VTAAPQSVCALR |  |
|  |  | |  |  |  |  | 638,5371 | 2 | 0,4995 | 60,27 | VGFYESDVMGR | Oxidation (M) |
|  |  | |  |  |  |  | 697,866 | 2 | 0,0449 | 101,04 | NEDSLVFVQTDK |  |
|  |  | |  |  |  |  | 741,1225 | 2 | 0,6043 | 51,91 | DMYSFLEDMGLK | 2 Oxidation (M) |
|  |  | |  |  |  |  | 857,5572 | 2 | 0,2708 | 78,73 | SSSNEEVMFLTVQVK | Oxidation (M) |
|  |  | |  |  |  |  | 942,7499 | 2 | 0,4448 | 66,5 | VSVQLEASPAFLAVPVEK |  |
| 9 | P0DOX7 | | Immunoglobulin kappa light chain OS=Homo sapiens OX=9606 PE=1 SV=1 | 560 | 23650 | 4 | 545,3002 | 2 | 0,0396 | 35,25 | ASSLESGVPSR |  |
|  |  | |  |  |  |  | 752,2028 | 2 | 0,6398 | 68,81 | DSTYSLSSTLTLSK |  |
|  |  | |  |  |  |  | 954,7835 | 2 | -0,3371 | 100 | DIQMTQSPSTLSASVGDR | Oxidation (M) |
|  |  | |  |  |  |  | 1068,414 | 2 | -0,1476 | 68,61 | VDNALQSGNSQESVTEQDSK |  |
| 10 | P01834 | | Immunoglobulin kappa constant OS=Homo sapiens OX=9606 GN=IGKC PE=1 SV=2 | 539 | 11929 | 3 | 752,2028 | 2 | 0,6398 | 68,81 | DSTYSLSSTLTLSK |  |
|  |  | |  |  |  |  | 973,743 | 2 | 0,4518 | 36,67 | TVAAPSVFIFPPSDEQLK |  |
|  |  | |  |  |  |  | 1068,414 | 2 | -0,1476 | 68,61 | VDNALQSGNSQESVTEQDSK |  |
| 11 | P01861 | | Immunoglobulin heavy constant gamma 4 OS=Homo sapiens OX=9606 GN=IGHG4 PE=1 SV=1 | 515 | 36431 | 6 | 425,9138 | 2 | -0,6089 | 25,33 | DTLMISR | Oxidation (M) |
|  |  | |  |  |  |  | 581,3823 | 2 | 0,1277 | 40,47 | NQVSLTCLVK |  |
|  |  | |  |  |  |  | 644,2135 | 2 | -0,2317 | 35,2 | GPSVFPLAPCSR |  |
|  |  | |  |  |  |  | 712,0803 | 2 | -0,5564 | 40,9 | STSESTAALGCLVK |  |
|  |  | |  |  |  |  | 946,9624 | 2 | 0,0229 | 52,53 | EPQVYTLPPSQEEMTK | Oxidation (M) |
|  |  | |  |  |  |  | 951,7167 | 2 | 0,4981 | 66,64 | TTPPVLDSDGSFFLYSR |  |
| 12 | P01871 | | Immunoglobulin heavy constant mu OS=Homo sapiens OX=9606 GN=IGHM PE=1 SV=4 | 460 | 50093 | 8 | 388,3406 | 2 | 0,2279 | 21,62 | GFPSVLR |  |
|  |  | |  |  |  |  | 432,0056 | 2 | 0,4794 | 32,13 | VTSTLTIK |  |
|  |  | |  |  |  |  | 450,7645 | 2 | -0,0084 | 37,15 | VSVFVPPR |  |
|  |  | |  |  |  |  | 625,6389 | 2 | 0,6348 | 65,36 | LICQATGFSPR |  |
|  |  | |  |  |  |  | 639,6941 | 2 | 0,6709 | 81,57 | YAATSQVLLPSK |  |
|  |  | |  |  |  |  | 694,1041 | 2 | 0,3655 | 28,76 | NVPLPVIAELPPK |  |
|  |  | |  |  |  |  | 808,947 | 2 | 0,1129 | 76,49 | YVTSAPMPEPQAPGR | Oxidation (M) |
|  |  | |  |  |  |  | 809,4885 | 2 | 0,1618 | 38,79 | QVGSGVTTDQVQAEAK |  |
| 13 | P01876 | | Immunoglobulin heavy constant alpha 1 OS=Homo sapiens OX=9606 GN=IGHA1 PE=1 SV=2 | 334 | 38486 | 7 | 410,0378 | 2 | 0,6641 | 31,17 | VAAEDWK |  |
|  |  | |  |  |  |  | 466,2172 | 2 | -0,1188 | 41,02 | TPLTATLSK |  |
|  |  | |  |  |  |  | 470,8961 | 2 | 0,3003 | 35,81 | SAVQGPPER |  |
|  |  | |  |  |  |  | 607,3019 | 2 | -0,0359 | 56 | WLQGSQELPR |  |
|  |  | |  |  |  |  | 688,6709 | 2 | 0,7147 | 56,66 | TFTCTAAYPESK |  |
|  |  | |  |  |  |  | 770,9368 | 2 | 0,1385 | 83,11 | DASGVTFTWTPSSGK |  |
|  |  | |  |  |  |  | 918,61 | 2 | 0,2629 | 45,97 | QEPSQGTTTFAVTSILR |  |
| 14 | P00738 | | Haptoglobin OS=Homo sapiens OX=9606 GN=HP PE=1 SV=1 | 186 | 45861 | 8 | 429,9886 | 2 | 0,4768 | 43,91 | QLVEIEK |  |
|  |  | |  |  |  |  | 490,9457 | 2 | 0,3892 | 59,58 | VGYVSGWGR |  |
|  |  | |  |  |  |  | 531,1327 | 2 | 0,7053 | 27,94 | VMPICLPSK | Oxidation (M) |
|  |  | |  |  |  |  | 574,0996 | 2 | 0,6493 | 27,39 | HYEGSTVPEK |  |
|  |  | |  |  |  |  | 602,638 | 2 | 0,6319 | 65,45 | VTSIQDWVQK |  |
|  |  | |  |  |  |  | 425,6882 | 3 | 0,4126 | 29,75 | HYEGSTVPEKK |  |
|  |  | |  |  |  |  | 645,6937 | 2 | -0,3502 | 57,6 | DIAPTLTLYVGK |  |
|  |  | |  |  |  |  | 854,8045 | 2 | 0,7824 | 28,31 | YVMLPVADQDQCIR |  |
| 15 | P0DOX2 | | Immunoglobulin alpha-2 heavy chain OS=Homo sapiens OX=9606 PE=1 SV=2 | 178 | 49816 | 6 | 410,0378 | 2 | 0,6641 | 31,17 | VAAEDWK |  |
|  |  | |  |  |  |  | 470,8961 | 2 | -0,6837 | 33,27 | SAVEGPPER |  |
|  |  | |  |  |  |  | 607,3019 | 2 | -0,0359 | 56 | WLQGSQELPR |  |
|  |  | |  |  |  |  | 660,0936 | 2 | 0,6068 | 74,44 | AEDTAVYYCAR |  |
|  |  | |  |  |  |  | 678,1143 | 2 | 0,5429 | 52,14 | NTVYLQMNSLR | Oxidation (M) |
|  |  | |  |  |  |  | 926,702 | 2 | 0,4521 | 101,45 | QEPSQGTTTYAVTSILR |  |
| 16 | P02649 | | Apolipoprotein E OS=Homo sapiens OX=9606 GN=APOE PE=1 SV=1 | 171 | 36246 | 3 | 749,3692 | 2 | -0,0708 | 73,61 | AATVGSLAGQPLQER |  |
|  |  | |  |  |  |  | 811,2347 | 2 | 0,6646 | 103,03 | VQAAVGTSAAPVPSDNH |  |
|  |  | |  |  |  |  | 866,3356 | 2 | 0,8196 | 53,31 | SELEEQLTPVAEETR |  |
| 17 | P0DOY2 | | Immunoglobulin lambda constant 2 OS=Homo sapiens OX=9606 GN=IGLC2 PE=1 SV=1 | 156 | 11458 | 2 | 496,0567 | 2 | 0,5958 | 61,22 | AGVETTTPSK |  |
|  |  | |  |  |  |  | 993,4547 | 2 | -0,1157 | 53,79 | AAPSVTLFPPSSEELQANK |  |
| 18 | P04004 | | Vitronectin OS=Homo sapiens OX=9606 GN=VTN PE=1 SV=1 | 151 | 55069 | 3 | 438,4338 | 2 | 0,387 | 46,91 | QPQFISR |  |
|  |  | |  |  |  |  | 711,7546 | 2 | -0,1517 | 70,18 | FEDGVLDPDYPR |  |
|  |  | |  |  |  |  | 561,7997 | 3 | 0,6141 | 26,94 | DWHGVPGQVDAAMAGR | Oxidation (M) |
| 19 | P01011 | | Alpha-1-antichymotrypsin OS=Homo sapiens OX=9606 GN=SERPINA3 PE=1 SV=2 | 145 | 47792 | 3 | 481,0059 | 2 | 0,4936 | 63,18 | ADLSGITGAR |  |
|  |  | |  |  |  |  | 531,6047 | 2 | 0,6145 | 35,72 | EIGELYLPK |  |
|  |  | |  |  |  |  | 954,6874 | 2 | 0,4078 | 116,83 | AVLDVFEEGTEASAATAVK |  |
| 20 | P0C0L4-2 | | Isoform 2 of Complement C4-A OS=Homo sapiens OX=9606 GN=C4A | 140 | 189125 | 5 | 485,4941 | 2 | 0,477 | 44,19 | VEYGFQVK |  |
|  |  | |  |  |  |  | 518,7611 | 2 | 0,968 | 41,79 | LGQYASPTAK |  |
|  |  | |  |  |  |  | 558,1147 | 2 | 0,6006 | 85,88 | VGDTLNLNLR |  |
|  |  | |  |  |  |  | 630,5568 | 2 | 0,4762 | 42,86 | EMSGSPASGIPVK |  |
|  |  | |  |  |  |  | 673,9598 | 2 | 0,2028 | 61,77 | LNMGITDLQGLR | Oxidation (M) |
| 21 | P02647 | | Apolipoprotein A-I OS=Homo sapiens OX=9606 GN=APOA1 PE=1 SV=1 | 121 | 30759 | 3 | 524,142 | 2 | -0,2372 | 29,59 | LSPLGEEMR | Oxidation (M) |
|  |  | |  |  |  |  | 627,0059 | 2 | 0,3836 | 41,75 | VQPYLDDFQK |  |
|  |  | |  |  |  |  | 701,2612 | 2 | 0,846 | 96,4 | DYVSQFEGSALGK |  |
| 22 | P01009 | | Alpha-1-antitrypsin OS=Homo sapiens OX=9606 GN=SERPINA1 PE=1 SV=3 | 119 | 46878 | 4 | 444,9094 | 2 | 0,3078 | 37,83 | AVLTIDEK |  |
|  |  | |  |  |  |  | 504,9924 | 2 | 0,478 | 32,01 | QINDYVEK |  |
|  |  | |  |  |  |  | 555,7862 | 2 | -0,039 | 53,44 | LSITGTYDLK |  |
|  |  | |  |  |  |  | 946,7085 | 2 | 0,5541 | 36,66 | DTEEEDFHVDQVTTVK |  |
| 23 | P02751 | | Fibronectin OS=Homo sapiens OX=9606 GN=FN1 PE=1 SV=5 | 118 | 275742 | 3 | 556,1782 | 2 | 0,8066 | 32,48 | STTPDITGYR |  |
|  |  | |  |  |  |  | 772,6513 | 2 | 0,5315 | 57,95 | SYTITGLQPGTDYK |  |
|  |  | |  |  |  |  | 1084,621 | 2 | 0,1843 | 77,85 | ITYGETGGNSPVQEFTVPGSK |  |
| 24 | Q03591 | | Complement factor H-related protein 1 OS=Homo sapiens OX=9606 GN=CFHR1 PE=1 SV=2 | 112 | 38766 | 3 | 564,1085 | 2 | -0,294 | 31,59 | TGESAEFVCK |  |
|  |  | |  |  |  |  | 691,6433 | 2 | 0,6014 | 42,82 | EIMENYNIALR | Oxidation (M) |
|  |  | |  |  |  |  | 753,2181 | 2 | -0,2651 | 66,99 | ITCTEEGWSPTPK |  |
| 25 | P00734 | | Prothrombin OS=Homo sapiens OX=9606 GN=F2 PE=1 SV=2 | 112 | 71475 | 2 | 626,7026 | 2 | 0,7401 | 64,55 | ETAASLLQAGYK |  |
|  |  | |  |  |  |  | 781,7805 | 2 | 0,8256 | 26,77 | TATSEYQTFFNPR |  |
| 26 | A0A0B4J1X5 | | Immunoglobulin heavy variable 3-74 OS=Homo sapiens OX=9606 GN=IGHV3-74 PE=3 SV=1 | 102 | 13002 | 2 | 660,0936 | 2 | 0,6068 | 74,44 | AEDTAVYYCAR |  |
|  |  | |  |  |  |  | 685,1239 | 2 | 0,5465 | 62,64 | NTLYLQMNSLR | Oxidation (M) |
| 27 | P04003 | | C4b-binding protein alpha chain OS=Homo sapiens OX=9606 GN=C4BPA PE=1 SV=2 | 82 | 69042 | 2 | 625,6461 | 2 | 0,6063 | 54,75 | EDVYVVGTVLR |  |
|  |  | |  |  |  |  | 730,6379 | 2 | 0,5767 | 60,26 | LMQCLPNPEDVK | Oxidation (M) |
| 28 | A0A075B6S6 | | Immunoglobulin kappa variable 2D-30 OS=Homo sapiens OX=9606 GN=IGKV2D-30 PE=3 SV=1 | 74 | 13321 | 2 | 652,6148 | 2 | 0,6058 | 74,22 | FSGSGSGTDFTLK |  |
|  |  | |  |  |  |  | 583,8777 | 3 | 0,7219 | 20,92 | LIYKVSNWDSGVPDR |  |
| 29 | P02790 | | Hemopexin OS=Homo sapiens OX=9606 GN=HPX PE=1 SV=2 | 68 | 52385 | 2 | 571,5367 | 2 | 0,4774 | 20,99 | GGYTLVSGYPK |  |
|  |  | |  |  |  |  | 579,9502 | 2 | 0,4224 | 21,86 | DYFMPCPGR | Oxidation (M) |
| 30 | P01008 | | Antithrombin-III OS=Homo sapiens OX=9606 GN=SERPINC1 PE=1 SV=1 | 61 | 53025 | 2 | 933,3624 | 2 | 0,8179 | 60,61 | EQLQDMGLVDLFSPEK | Oxidation (M) |
|  |  | |  |  |  |  | 625,7504 | 3 | 0,364 | 27,47 | ATEDEGSEQKIPEATNR |  |
| 31 | P00450 | | Ceruloplasmin OS=Homo sapiens OX=9606 GN=CP PE=1 SV=1 | 58 | 122983 | 4 | 509,1913 | 2 | -0,0883 | 22,96 | QYTDSTFR |  |
|  |  | |  |  |  |  | 602,7007 | 2 | 0,8666 | 39,99 | EYTDASFTNR |  |
|  |  | |  |  |  |  | 686,6628 | 2 | 0,5552 | 40,68 | GAYPLSIEPIGVR |  |
|  |  | |  |  |  |  | 716,7082 | 2 | 0,7704 | 33,27 | QSEDSTFYLGER |  |
| 32 | P08603 | | Complement factor H OS=Homo sapiens OX=9606 GN=CFH PE=1 SV=4 | 53 | 143680 | 2 | 578,6184 | 2 | 0,6945 | 23,25 | TGESVEFVCK |  |
|  |  | |  |  |  |  | 691,6433 | 2 | 0,6014 | 42,82 | EIMENYNIALR | Oxidation (M) |

**Table S2.12** PEG-MWCNTs-mDAAO Hard Corona R2

| **Prot. Number** | **Accession Number** | **Protein Name** | **Mascot Score** | **Mr** | **N° pep** | **m/z** | **z** | **Pep. error** | **Pep. Score** | **Pep. sequence** | **Pep. Modification** |
| --- | --- | --- | --- | --- | --- | --- | --- | --- | --- | --- | --- |
| 1 | P02768 | Serum albumin OS=Homo sapiens OX=9606 GN=ALB PE=1 SV=2 | 5369 | 71317 | 28 | 387,0041 | 2 | 0,5624 | 33,44 | AACLLPK |  |
|  |  |  |  |  |  | 395,2328 | 2 | -0,0132 | 21,95 | LVTDLTK |  |
|  |  |  |  |  |  | 440,4436 | 2 | -0,5611 | 35,99 | AEFAEVSK |  |
|  |  |  |  |  |  | 464,2474 | 2 | -0,0058 | 40,76 | YLYEIAR |  |
|  |  |  |  |  |  | 467,3136 | 2 | 0,1014 | 31,99 | LCTVATLR |  |
|  |  |  |  |  |  | 470,5095 | 2 | -0,4366 | 24,39 | DDNPNLPR |  |
|  |  |  |  |  |  | 480,6984 | 2 | -0,1729 | 21,44 | FQNALLVR |  |
|  |  |  |  |  |  | 492,4825 | 2 | -0,5306 | 22,25 | TYETTLEK |  |
|  |  |  |  |  |  | 500,8833 | 2 | 0,1556 | 48,67 | QTALVELVK |  |
|  |  |  |  |  |  | 1013,225 | 1 | -0,3741 | 20,44 | LVAASQAALGL |  |
|  |  |  |  |  |  | 509,4149 | 2 | 0,2861 | 45,47 | SLHTLFGDK |  |
|  |  |  |  |  |  | 537,6404 | 2 | -0,269 | 23,14 | LDELRDEGK |  |
|  |  |  |  |  |  | 565,044 | 2 | 0,3821 | 20,89 | KQTALVELVK |  |
|  |  |  |  |  |  | 569,3077 | 2 | -0,8899 | 29,47 | CCTESLVNR |  |
|  |  |  |  |  |  | 571,4031 | 2 | 0,105 | 52,96 | KLVAASQAALGL |  |
|  |  |  |  |  |  | 574,8916 | 2 | -0,8391 | 70,1 | LVNEVTEFAK |  |
|  |  |  |  |  |  | 613,859 | 2 | 0,1056 | 58,85 | FKDLGEENFK |  |
|  |  |  |  |  |  | 679,4157 | 2 | -0,8057 | 74,71 | AVMDDFAAFVEK | Oxidation (M) |
|  |  |  |  |  |  | 686,1562 | 2 | -0,2617 | 60,19 | AAFTECCQAADK |  |
|  |  |  |  |  |  | 721,9758 | 2 | -0,6976 | 65,75 | YICENQDSISSK |  |
|  |  |  |  |  |  | 725,642 | 2 | -0,2517 | 71,66 | ETYGEMADCCAK | Oxidation (M) |
|  |  |  |  |  |  | 749,796 | 2 | 0,0062 | 70,71 | TCVADESAENCDK |  |
|  |  |  |  |  |  | 756,3466 | 2 | -0,1568 | 24,41 | VPQVSTPTLVEVSR |  |
|  |  |  |  |  |  | 547,0573 | 3 | -0,7804 | 33,5 | KVPQVSTPTLVEVSR |  |
|  |  |  |  |  |  | 820,5218 | 2 | 0,2539 | 72,19 | DVFLGMFLYEYAR | Oxidation (M) |
|  |  |  |  |  |  | 547,351 | 3 | 0,1005 | 56,82 | KVPQVSTPTLVEVSR |  |
|  |  |  |  |  |  | 820,5811 | 2 | 0,3725 | 59,36 | DVFLGMFLYEYAR | Oxidation (M) |
|  |  |  |  |  |  | 547,4036 | 3 | 0,2584 | 34,16 | KVPQVSTPTLVEVSR |  |
|  |  |  |  |  |  | 820,6365 | 2 | 0,4832 | 60 | DVFLGMFLYEYAR | Oxidation (M) |
|  |  |  |  |  |  | 547,4334 | 3 | 0,3479 | 63,1 | KVPQVSTPTLVEVSR |  |
|  |  |  |  |  |  | 828,995 | 2 | -0,7699 | 56,24 | QNCELFEQLGEYK |  |
|  |  |  |  |  |  | 637,8105 | 3 | 0,4852 | 34,81 | RPCFSALEVDETYVPK |  |
|  |  |  |  |  |  | 1023 | 2 | -0,1021 | 71,59 | VFDEFKPLVEEPQNLIK |  |
| 2 | P0DOX5 | Immunoglobulin gamma-1 heavy chain OS=Homo sapiens OX=9606 PE=1 SV=2 | 1203 | 49925 | 8 | 623,4728 | 1 | 0,1216 | 24,16 | FTISR |  |
|  |  |  |  |  |  | 419,679 | 2 | -0,1525 | 21,35 | ALPAPIEK |  |
|  |  |  |  |  |  | 581,5127 | 2 | 0,3885 | 44,41 | NQVSLTCLVK |  |
|  |  |  |  |  |  | 593,565 | 2 | -0,5239 | 51,33 | GPSVFPLAPSSK |  |
|  |  |  |  |  |  | 643,6747 | 2 | -0,3318 | 22,02 | EPQVYTLPPSR |  |
|  |  |  |  |  |  | 661,1847 | 2 | -0,3159 | 75,54 | STSGGTAALGCLVK |  |
|  |  |  |  |  |  | 937,014 | 2 | 0,0507 | 41,18 | EPQVYTLPPSRDELTK |  |
|  |  |  |  |  |  | 937,1101 | 2 | -0,7089 | 48,89 | TTPPVLDSDGSFFLYSK |  |
|  |  |  |  |  |  | 937,1243 | 2 | 0,2711 | 42,42 | EPQVYTLPPSRDELTK |  |
|  |  |  |  |  |  | 937,4842 | 2 | 0,0394 | 51,61 | TTPPVLDSDGSFFLYSK |  |
| 3 | P01009 | Alpha-1-antitrypsin OS=Homo sapiens OX=9606 GN=SERPINA1 PE=1 SV=3 | 776 | 46878 | 6 | 444,6284 | 2 | -0,2542 | 31,3 | AVLTIDEK |  |
|  |  |  |  |  |  | 504,5914 | 2 | -0,3241 | 23,48 | QINDYVEK |  |
|  |  |  |  |  |  | 508,423 | 2 | 0,224 | 46,16 | SVLGQLGITK |  |
|  |  |  |  |  |  | 555,5508 | 2 | -0,5097 | 46,58 | LSITGTYDLK |  |
|  |  |  |  |  |  | 917,7406 | 2 | 0,551 | 92,25 | VFSNGADLSGVTEEAPLK |  |
|  |  |  |  |  |  | 1146,1 | 2 | 0,0622 | 71,35 | GTEAAGAMFLEAIPMSIPPEVK | 2 Oxidation (M) |
| 4 | P01024 | Complement C3 OS=Homo sapiens OX=9606 GN=C3 PE=1 SV=2 | 739 | 188569 | 12 | 531,6807 | 2 | -0,1344 | 57,4 | ADIGCTPGSGK |  |
|  |  |  |  |  |  | 556,082 | 2 | 0,5302 | 69,71 | VLLDGVQNPR |  |
|  |  |  |  |  |  | 570,4282 | 2 | 0,3084 | 28,62 | FYYIYNEK |  |
|  |  |  |  |  |  | 612,6881 | 2 | -0,2279 | 49,21 | YYTYLIMNK | Oxidation (M) |
|  |  |  |  |  |  | 645,4221 | 2 | 0,2289 | 68,22 | SGSDEVQVGQQR |  |
|  |  |  |  |  |  | 685,7109 | 2 | -0,3168 | 74,02 | TIYTPGSTVLYR |  |
|  |  |  |  |  |  | 736,2802 | 2 | 0,7732 | 81,44 | IPIEDGSGEVVLSR |  |
|  |  |  |  |  |  | 756,3466 | 2 | -0,1357 | 66,48 | LVAYYTLIGASGQR |  |
|  |  |  |  |  |  | 828,6744 | 2 | 0,4743 | 102,54 | TVMVNIENPEGIPVK | Oxidation (M) |
|  |  |  |  |  |  | 940,2539 | 2 | 0,527 | 102,31 | EYVLPSFEVIVEPTEK |  |
|  |  |  |  |  |  | 1086,989 | 2 | -0,1093 | 112,51 | ILLQGTPVAQMTEDAVDAER | Oxidation (M) |
|  |  |  |  |  |  | 1107,987 | 2 | 0,9629 | 82,86 | EDIPPADLSDQVPDTESETR |  |
| 5 | P01834 | Immunoglobulin kappa constant OS=Homo sapiens OX=9606 GN=IGKC PE=1 SV=2 | 465 | 11929 | 2 | 973,6713 | 2 | 0,3083 | 80,42 | TVAAPSVFIFPPSDEQLK |  |
|  |  |  |  |  |  | 1068,526 | 2 | 0,0758 | 138,46 | VDNALQSGNSQESVTEQDSK |  |
| 6 | P02787 | Serotransferrin OS=Homo sapiens OX=9606 GN=TF PE=1 SV=3 | 423 | 79294 | 6 | 490,0669 | 2 | 0,6375 | 66,24 | DGAGDVAFVK |  |
|  |  |  |  |  |  | 500,8787 | 2 | 0,2516 | 37,6 | YLGEEYVK |  |
|  |  |  |  |  |  | 606,3622 | 2 | 0,1697 | 46,53 | DSGFQMNQLR | Oxidation (M) |
|  |  |  |  |  |  | 642,287 | 2 | -0,0024 | 53,85 | EGYYGYTGAFR |  |
|  |  |  |  |  |  | 748,1412 | 2 | 0,5454 | 76,89 | MYLGYEYVTAIR | Oxidation (M) |
|  |  |  |  |  |  | 815,4625 | 2 | 0,1019 | 67,63 | EDPQTFYYAVAVVK |  |
| 7 | P01859 | Immunoglobulin heavy constant gamma 2 OS=Homo sapiens OX=9606 GN=IGHG2 PE=1 SV=2 | 385 | 36505 | 5 | 581,5127 | 2 | 0,3885 | 44,41 | NQVSLTCLVK |  |
|  |  |  |  |  |  | 643,6747 | 2 | -0,3318 | 22,02 | EPQVYTLPPSR |  |
|  |  |  |  |  |  | 712,0956 | 2 | -0,5257 | 27,13 | STSESTAALGCLVK |  |
|  |  |  |  |  |  | 961,0889 | 2 | 0,2333 | 56,37 | EPQVYTLPPSREEMTK | Oxidation (M) |
|  |  |  |  |  |  | 961,4342 | 2 | -0,0277 | 46,47 | TTPPMLDSDGSFFLYSK | Oxidation (M) |
| 8 | P01871 | Immunoglobulin heavy constant mu OS=Homo sapiens OX=9606 GN=IGHM PE=1 SV=4 | 381 | 50093 | 5 | 431,6147 | 2 | -0,3023 | 25,07 | VTSTLTIK |  |
|  |  |  |  |  |  | 450,9878 | 2 | 0,4382 | 24,32 | VSVFVPPR |  |
|  |  |  |  |  |  | 639,6717 | 2 | 0,6263 | 66,43 | YAATSQVLLPSK |  |
|  |  |  |  |  |  | 809,057 | 2 | 0,333 | 76,33 | YVTSAPMPEPQAPGR | Oxidation (M) |
|  |  |  |  |  |  | 809,4426 | 2 | 0,0701 | 63,1 | QVGSGVTTDQVQAEAK |  |
| 9 | P01861 | Immunoglobulin heavy constant gamma 4 OS=Homo sapiens OX=9606 GN=IGHG4 PE=1 SV=1 | 327 | 36431 | 4 | 581,5127 | 2 | 0,3885 | 44,41 | NQVSLTCLVK |  |
|  |  |  |  |  |  | 712,0956 | 2 | -0,5257 | 27,13 | STSESTAALGCLVK |  |
|  |  |  |  |  |  | 946,8778 | 2 | -0,1463 | 26,79 | EPQVYTLPPSQEEMTK | Oxidation (M) |
|  |  |  |  |  |  | 951,4943 | 2 | 0,0533 | 71,09 | TTPPVLDSDGSFFLYSR |  |
| 10 | P01023 | Alpha-2-macroglobulin OS=Homo sapiens OX=9606 GN=A2M PE=1 SV=3 | 319 | 164613 | 10 | 414,8773 | 2 | 0,29 | 28,17 | SDIAPVAR |  |
|  |  |  |  |  |  | 509,6341 | 2 | -0,3322 | 45,03 | ATVLNYLPK |  |
|  |  |  |  |  |  | 575,8837 | 2 | 0,1828 | 22,91 | SASNMAIVDVK | Oxidation (M) |
|  |  |  |  |  |  | 606,0769 | 2 | 0,5039 | 37,04 | LPPNVVEESAR |  |
|  |  |  |  |  |  | 638,1871 | 2 | -0,2004 | 21,2 | VGFYESDVMGR | Oxidation (M) |
|  |  |  |  |  |  | 698,0802 | 2 | 0,4733 | 70,35 | NEDSLVFVQTDK |  |
|  |  |  |  |  |  | 475,3129 | 3 | 0,1185 | 24,77 | SIYKPGQTVKFR |  |
|  |  |  |  |  |  | 740,5752 | 2 | -0,4903 | 33,26 | DMYSFLEDMGLK | 2 Oxidation (M) |
|  |  |  |  |  |  | 923,4985 | 2 | 0,953 | 95,95 | LLIYAVLPTGDVIGDSAK |  |
|  |  |  |  |  |  | 1031,491 | 2 | 0,8808 | 28,57 | LLLQQVSLPELPGEYSMK | Oxidation (M) |
| 11 | P00738 | Haptoglobin OS=Homo sapiens OX=9606 GN=HP PE=1 SV=1 | 181 | 45861 | 4 | 405,2905 | 2 | 0,195 | 31,13 | DYAEVGR |  |
|  |  |  |  |  |  | 574,0209 | 2 | 0,492 | 25,69 | HYEGSTVPEK |  |
|  |  |  |  |  |  | 645,7319 | 2 | -0,2738 | 52,65 | DIAPTLTLYVGK |  |
|  |  |  |  |  |  | 673,3961 | 2 | 0,1393 | 82,02 | SCAVAEYGVYVK |  |
| 12 | P01876 | Immunoglobulin heavy constant alpha 1 OS=Homo sapiens OX=9606 GN=IGHA1 PE=1 SV=2 | 163 | 38486 | 3 | 470,6853 | 2 | -0,1213 | 40,72 | SAVQGPPER |  |
|  |  |  |  |  |  | 688,4614 | 2 | 0,2957 | 27,52 | TFTCTAAYPESK |  |
|  |  |  |  |  |  | 918,1572 | 2 | -0,6426 | 42,05 | QEPSQGTTTFAVTSILR |  |
| 13 | P01011 | Alpha-1-antichymotrypsin OS=Homo sapiens OX=9606 GN=SERPINA3 PE=1 SV=2 | 151 | 47792 | 2 | 608,4972 | 2 | 0,2564 | 83,99 | ITLLSALVETR |  |
|  |  |  |  |  |  | 954,579 | 2 | 0,191 | 100,63 | AVLDVFEEGTEASAATAVK |  |
| 14 | P0DOY2 | Immunoglobulin lambda constant 2 OS=Homo sapiens OX=9606 GN=IGLC2 PE=1 SV=1 | 132 | 11458 | 2 | 496,1622 | 2 | 0,8069 | 54,04 | AGVETTTPSK |  |
|  |  |  |  |  |  | 993,6171 | 2 | 0,209 | 67,23 | AAPSVTLFPPSSEELQANK |  |
| 15 | P02790 | Hemopexin OS=Homo sapiens OX=9606 GN=HPX PE=1 SV=2 | 70 | 52385 | 2 | 571,3145 | 2 | 0,0329 | 56,75 | GGYTLVSGYPK |  |
|  |  |  |  |  |  | 611,0674 | 2 | 0,5216 | 33,58 | NFPSPVDAAFR |  |
| 16 | P0C0L5 | Complement C4-B OS=Homo sapiens OX=9606 GN=C4B PE=1 SV=2 | 68 | 194170 | 4 | 518,6584 | 2 | 0,7626 | 22,33 | LGQYASPTAK |  |
|  |  |  |  |  |  | 557,9172 | 2 | 0,2057 | 68,75 | VGDTLNLNLR |  |
|  |  |  |  |  |  | 638,4912 | 2 | 0,3502 | 30,19 | EMSGSPASGIPVK | Oxidation (M) |
|  |  |  |  |  |  | 518,4526 | 3 | 0,5217 | 22,35 | VEASISKASSFLGEK |  |
| 17 | Q9NPI6 | mRNA-decapping enzyme 1A OS=Homo sapiens OX=9606 GN=DCP1A PE=1 SV=3 | 65 | 63410 | 2 | 683,5989 | 1 | 0,2518 | 22 | SAPSGHK |  |
|  |  |  |  |  |  | 535,3182 | 3 | -0,8892 | 24,94 | RSASPYHGFTIVNR |  |
| 18 | P04114 | Apolipoprotein B-100 OS=Homo sapiens OX=9606 GN=APOB PE=1 SV=2 | 64 | 516651 | 4 | 1039,742 | 1 | 0,1019 | 26,82 | LAPGELTIIL |  |
|  |  |  |  |  |  | 557,2998 | 2 | 0,0129 | 20,61 | LAAYLMLMR | 2 Oxidation (M) |
|  |  |  |  |  |  | 614,9659 | 2 | 0,3984 | 66,29 | NSEEFAAAMSR | Oxidation (M) |
|  |  |  |  |  |  | 635,0282 | 2 | 0,3283 | 31,01 | DLKVEDIPLAR |  |
