# Supplementary material for: The D-amino acid oxidase-carbon nanotubes: evaluation of cytotoxicity and biocompatibility of a potential anticancer nanosystem
Source: 3 Biotech. 2023 Jun 19;13(7):243. doi: 10.1007/s13205-023-03568-1 (PMC10279611; doi:10.1007/s13205-023-03568-1)
Supplement: Supplementary file 3 — Supplementary file3 (DOCX 40 KB) [file 13205_2023_3568_MOESM3_ESM.docx]

**Table S3.1** PEG-MWCNTs Soft Corona

| **Accession Number** | **Protein Name** | **Mascot Score** | **Mr** | **N° peptides** |
| --- | --- | --- | --- | --- |
| P02768 | Serum albumin OS=Homo sapiens OX=9606 GN=ALB PE=1 SV=2 | 11758 | 71317 | 42 |
| P02647 | Apolipoprotein A-I OS=Homo sapiens OX=9606 GN=APOA1 PE=1 SV=1 | 2012 | 30759 | 20 |
| P02787 | Serotransferrin OS=Homo sapiens OX=9606 GN=TF PE=1 SV=3 | 1775 | 79294 | 25 |
| P01024 | Complement C3 OS=Homo sapiens OX=9606 GN=C3 PE=1 SV=2 | 1693 | 188569 | 30 |
| P01834 | Immunoglobulin kappa constant OS=Homo sapiens OX=9606 GN=IGKC PE=1 SV=2 | 1345 | 11929 | 5 |
| P01009 | Alpha-1-antitrypsin OS=Homo sapiens OX=9606 GN=SERPINA1 PE=1 SV=3 | 1338 | 46878 | 9 |
| P00738 | Haptoglobin OS=Homo sapiens OX=9606 GN=HP PE=1 SV=1 | 1240 | 45861 | 11 |
| P01023 | Alpha-2-macroglobulin OS=Homo sapiens OX=9606 GN=A2M PE=1 SV=3 | 1061 | 164613 | 22 |
| P0DOX5 | Immunoglobulin gamma-1 heavy chain OS=Homo sapiens OX=9606 PE=1 SV=2 | 1052 | 49925 | 7 |
| P0DOX7 | Immunoglobulin kappa light chain OS=Homo sapiens OX=9606 PE=1 SV=1 | 1012 | 23650 | 7 |
| P04114 | Apolipoprotein B-100 OS=Homo sapiens OX=9606 GN=APOB PE=1 SV=2 | 937 | 516651 | 32 |
| P01860 | Immunoglobulin heavy constant gamma 3 OS=Homo sapiens OX=9606 GN=IGHG3 PE=1 SV=2 | 722 | 42287 | 8 |
| P0DOY2 | Immunoglobulin lambda constant 2 OS=Homo sapiens OX=9606 GN=IGLC2 PE=1 SV=1 | 585 | 11458 | 5 |
| P01859 | Immunoglobulin heavy constant gamma 2 OS=Homo sapiens OX=9606 GN=IGHG2 PE=1 SV=2 | 549 | 36505 | 7 |
| P01861 | Immunoglobulin heavy constant gamma 4 OS=Homo sapiens OX=9606 GN=IGHG4 PE=1 SV=1 | 512 | 36431 | 6 |
| P01042 | Kininogen-1 OS=Homo sapiens OX=9606 GN=KNG1 PE=1 SV=2 | 467 | 72996 | 10 |
| P01876 | Immunoglobulin heavy constant alpha 1 OS=Homo sapiens OX=9606 GN=IGHA1 PE=1 SV=2 | 455 | 38486 | 8 |
| P0CG04 | Immunoglobulin lambda constant 1 OS=Homo sapiens OX=9606 GN=IGLC1 PE=1 SV=1 | 366 | 11512 | 4 |
| P05155 | Plasma protease C1 inhibitor OS=Homo sapiens OX=9606 GN=SERPING1 PE=1 SV=2 | 347 | 55347 | 5 |
| P0C0L4 | Complement C4-A OS=Homo sapiens OX=9606 GN=C4A PE=1 SV=2 | 221 | 194261 | 5 |
| P00450 | Ceruloplasmin OS=Homo sapiens OX=9606 GN=CP PE=1 SV=1 | 200 | 122983 | 5 |
| P02790 | Hemopexin OS=Homo sapiens OX=9606 GN=HPX PE=1 SV=2 | 197 | 52385 | 5 |
| P10909 | Clusterin OS=Homo sapiens OX=9606 GN=CLU PE=1 SV=1 | 175 | 53031 | 4 |
| P01011 | Alpha-1-antichymotrypsin OS=Homo sapiens OX=9606 GN=SERPINA3 PE=1 SV=2 | 173 | 47792 | 2 |
| P08603 | Complement factor H OS=Homo sapiens OX=9606 GN=CFH PE=1 SV=4 | 127 | 143680 | 6 |
| P01871 | Immunoglobulin heavy constant mu OS=Homo sapiens OX=9606 GN=IGHM PE=1 SV=4 | 104 | 50093 | 2 |

**Table S3.2** PEG-MWCNTs Hard Corona

| **Accession Number** | **Protein Name** | **Mascot Score** | **Mr** | **N° peptides** |
| --- | --- | --- | --- | --- |
| P02768 | Serum albumin OS=Homo sapiens OX=9606 GN=ALB PE=1 SV=2 | 8190 | 71317 | 34 |
| P04114 | Apolipoprotein B-100 OS=Homo sapiens OX=9606 GN=APOB PE=1 SV=2 | 3417 | 516651 | 77 |
| P02647 | Apolipoprotein A-I OS=Homo sapiens OX=9606 GN=APOA1 PE=1 SV=1 | 2044 | 30759 | 20 |
| P01024 | Complement C3 OS=Homo sapiens OX=9606 GN=C3 PE=1 SV=2 | 1340 | 188569 | 24 |
| P01834 | Immunoglobulin kappa constant OS=Homo sapiens OX=9606 GN=IGKC PE=1 SV=2 | 1073 | 11929 | 5 |
| P01009 | Alpha-1-antitrypsin OS=Homo sapiens OX=9606 GN=SERPINA1 PE=1 SV=3 | 952 | 46878 | 11 |
| P0DOX5 | Immunoglobulin gamma-1 heavy chain OS=Homo sapiens OX=9606 PE=1 SV=2 | 906 | 49925 | 9 |
| P0DOX7 | Immunoglobulin kappa light chain OS=Homo sapiens OX=9606 PE=1 SV=1 | 824 | 23650 | 6 |
| P00738 | Haptoglobin OS=Homo sapiens OX=9606 GN=HP PE=1 SV=1 | 754 | 45861 | 13 |
| P01859 | Immunoglobulin heavy constant gamma 2 OS=Homo sapiens OX=9606 GN=IGHG2 PE=1 SV=2 | 426 | 36505 | 8 |
| P01876 | Immunoglobulin heavy constant alpha 1 OS=Homo sapiens OX=9606 GN=IGHA1 PE=1 SV=2 | 413 | 38486 | 6 |
| P01023 | Alpha-2-macroglobulin OS=Homo sapiens OX=9606 GN=A2M PE=1 SV=3 | 406 | 164613 | 13 |
| P0C0L4 | Complement C4-A OS=Homo sapiens OX=9606 GN=C4A PE=1 SV=2 | 383 | 194261 | 4 |
| P01861 | Immunoglobulin heavy constant gamma 4 OS=Homo sapiens OX=9606 GN=IGHG4 PE=1 SV=1 | 379 | 36431 | 5 |
| P02787 | Serotransferrin OS=Homo sapiens OX=9606 GN=TF PE=1 SV=3 | 378 | 79294 | 7 |
| P0DOY2 | Immunoglobulin lambda constant 2 OS=Homo sapiens OX=9606 GN=IGLC2 PE=1 SV=1 | 332 | 11458 | 4 |
| P27169 | Serum paraoxonase/arylesterase 1 OS=Homo sapiens OX=9606 GN=PON1 PE=1 SV=3 | 247 | 39877 | 4 |
| Q03591 | Complement factor H-related protein 1 OS=Homo sapiens OX=9606 GN=CFHR1 PE=1 SV=2 | 219 | 38766 | 4 |
| P0DOX8 | Immunoglobulin lambda-1 light chain OS=Homo sapiens OX=9606 PE=1 SV=1 | 201 | 23101 | 4 |
| P01011 | Alpha-1-antichymotrypsin OS=Homo sapiens OX=9606 GN=SERPINA3 PE=1 SV=2 | 191 | 47792 | 4 |
| P0DOX2 | Immunoglobulin alpha-2 heavy chain OS=Homo sapiens OX=9606 PE=1 SV=2 | 168 | 49816 | 5 |
| P02747 | Complement C1q subcomponent subunit C OS=Homo sapiens OX=9606 GN=C1QC PE=1 SV=3 | 152 | 25985 | 2 |
| P02649 | Apolipoprotein E OS=Homo sapiens OX=9606 GN=APOE PE=1 SV=1 | 134 | 36246 | 3 |
| P01019 | Angiotensinogen OS=Homo sapiens OX=9606 GN=AGT PE=1 SV=1 | 84 | 53406 | 3 |

**Table S3.3** PEG-MWCNTs-wtDAAO Soft Corona

| **Accession Number** | **Protein Name** | **Mascot Score** | **Mr** | **N° peptides** |
| --- | --- | --- | --- | --- |
| P02768 | Serum albumin OS=Homo sapiens OX=9606 GN=ALB PE=1 SV=2 | 12080 | 71317 | 46 |
| P04114 | Apolipoprotein B-100 OS=Homo sapiens OX=9606 GN=APOB PE=1 SV=2 | 3531 | 516651 | 82 |
| P02787 | Serotransferrin OS=Homo sapiens OX=9606 GN=TF PE=1 SV=3 | 1864 | 79294 | 25 |
| P01834 | Immunoglobulin kappa constant OS=Homo sapiens OX=9606 GN=IGKC PE=1 SV=2 | 1375 | 11929 | 5 |
| P0DOX5 | Immunoglobulin gamma-1 heavy chain OS=Homo sapiens OX=9606 PE=1 SV=2 | 1350 | 49925 | 9 |
| P0DOX7 | Immunoglobulin kappa light chain OS=Homo sapiens OX=9606 PE=1 SV=1 | 1322 | 23650 | 7 |
| P01009 | Alpha-1-antitrypsin OS=Homo sapiens OX=9606 GN=SERPINA1 PE=1 SV=3 | 1244 | 46878 | 9 |
| P01024 | Complement C3 OS=Homo sapiens OX=9606 GN=C3 PE=1 SV=2 | 1095 | 188569 | 23 |
| P00738 | Haptoglobin OS=Homo sapiens OX=9606 GN=HP PE=1 SV=1 | 1077 | 45861 | 17 |
| P01023 | Alpha-2-macroglobulin OS=Homo sapiens OX=9606 GN=A2M PE=1 SV=3 | 764 | 164613 | 25 |
| P0DOY2 | Immunoglobulin lambda constant 2 OS=Homo sapiens OX=9606 GN=IGLC2 PE=1 SV=1 | 753 | 11458 | 5 |
| P01860 | Immunoglobulin heavy constant gamma 3 OS=Homo sapiens OX=9606 GN=IGHG3 PE=1 SV=2 | 749 | 42287 | 7 |
| P02647 | Apolipoprotein A-I OS=Homo sapiens OX=9606 GN=APOA1 PE=1 SV=1 | 609 | 30759 | 14 |
| P01859 | Immunoglobulin heavy constant gamma 2 OS=Homo sapiens OX=9606 GN=IGHG2 PE=1 SV=2 | 553 | 36505 | 7 |
| P01861 | Immunoglobulin heavy constant gamma 4 OS=Homo sapiens OX=9606 GN=IGHG4 PE=1 SV=1 | 514 | 36431 | 6 |
| P01876 | Immunoglobulin heavy constant alpha 1 OS=Homo sapiens OX=9606 GN=IGHA1 PE=1 SV=2 | 506 | 38486 | 7 |
| P00450 | Ceruloplasmin OS=Homo sapiens OX=9606 GN=CP PE=1 SV=1 | 224 | 122983 | 5 |
| P01871 | Immunoglobulin heavy constant mu OS=Homo sapiens OX=9606 GN=IGHM PE=1 SV=4 | 210 | 50093 | 11 |
| P05155 | Plasma protease C1 inhibitor OS=Homo sapiens OX=9606 GN=SERPING1 PE=1 SV=2 | 180 | 55347 | 6 |
| P00734 | Prothrombin OS=Homo sapiens OX=9606 GN=F2 PE=1 SV=2 | 170 | 71475 | 3 |
| P02790 | Hemopexin OS=Homo sapiens OX=9606 GN=HPX PE=1 SV=2 | 160 | 52385 | 3 |
| P02743 | Serum amyloid P-component OS=Homo sapiens OX=9606 GN=APCS PE=1 SV=2 | 159 | 25485 | 3 |
| P01011 | Alpha-1-antichymotrypsin OS=Homo sapiens OX=9606 GN=SERPINA3 PE=1 SV=2 | 153 | 47792 | 3 |
| P0DOX2 | Immunoglobulin alpha-2 heavy chain OS=Homo sapiens OX=9606 PE=1 SV=2 | 140 | 49816 | 5 |
| A0A0B4J1X5 | Immunoglobulin heavy variable 3-74 OS=Homo sapiens OX=9606 GN=IGHV3-74 PE=3 SV=1 | 119 | 13002 | 2 |
| P00751 | Complement factor B OS=Homo sapiens OX=9606 GN=CFB PE=1 SV=2 | 119 | 86847 | 2 |
| P02774-2 | Isoform 2 of Vitamin D-binding protein OS=Homo sapiens OX=9606 GN=GC | 104 | 40611 | 3 |
| P01042 | Kininogen-1 OS=Homo sapiens OX=9606 GN=KNG1 PE=1 SV=2 | 99 | 72996 | 2 |
| P04003 | C4b-binding protein alpha chain OS=Homo sapiens OX=9606 GN=C4BPA PE=1 SV=2 | 85 | 69042 | 3 |

**Table S3.4** PEG-MWCNTs-wtDAAO Hard Corona

| **Accession Number** | **Protein Name** | **Mascot Score** | **Mr** | **N° peptides** |
| --- | --- | --- | --- | --- |
| P02768 | Serum albumin OS=Homo sapiens OX=9606 GN=ALB PE=1 SV=2 | 5655 | 71317 | 29 |
| P04114 | Apolipoprotein B-100 OS=Homo sapiens OX=9606 GN=APOB PE=1 SV=2 | 1524 | 516651 | 39 |
| P01834 | Immunoglobulin kappa constant OS=Homo sapiens OX=9606 GN=IGKC PE=1 SV=2 | 860 | 11929 | 5 |
| P0DOX5 | Immunoglobulin gamma-1 heavy chain OS=Homo sapiens OX=9606 PE=1 SV=2 | 795 | 49925 | 7 |
| P01009 | Alpha-1-antitrypsin OS=Homo sapiens OX=9606 GN=SERPINA1 PE=1 SV=3 | 247 | 46878 | 4 |
| P01024 | Complement C3 OS=Homo sapiens OX=9606 GN=C3 PE=1 SV=2 | 223 | 188569 | 10 |
| P02787 | Serotransferrin OS=Homo sapiens OX=9606 GN=TF PE=1 SV=3 | 108 | 79294 | 3 |

**Table S3.5** PEG-MWCNTs-mDAAO Soft Corona

| **Accession Number** | **Protein Name** | **Mascot Score** | **Mr** | **N° peptides** |
| --- | --- | --- | --- | --- |
| P02768 | Serum albumin OS=Homo sapiens OX=9606 GN=ALB PE=1 SV=2 | 5172 | 71317 | 29 |
| P0DOX5 | Immunoglobulin gamma-1 heavy chain OS=Homo sapiens OX=9606 PE=1 SV=2 | 1474 | 49925 | 9 |
| P02787 | Serotransferrin OS=Homo sapiens OX=9606 GN=TF PE=1 SV=3 | 1077 | 79294 | 18 |
| P01834 | Immunoglobulin kappa constant OS=Homo sapiens OX=9606 GN=IGKC PE=1 SV=2 | 1020 | 11929 | 3 |
| P04114 | Apolipoprotein B-100 OS=Homo sapiens OX=9606 GN=APOB PE=1 SV=2 | 662 | 516651 | 24 |
| P01859 | Immunoglobulin heavy constant gamma 2 OS=Homo sapiens OX=9606 GN=IGHG2 PE=1 SV=2 | 488 | 36505 | 5 |
| P01024 | Complement C3 OS=Homo sapiens OX=9606 GN=C3 PE=1 SV=2 | 339 | 188569 | 11 |
| P01876 | Immunoglobulin heavy constant alpha 1 OS=Homo sapiens OX=9606 GN=IGHA1 PE=1 SV=2 | 259 | 38486 | 7 |
| P01009 | Alpha-1-antitrypsin OS=Homo sapiens OX=9606 GN=SERPINA1 PE=1 SV=3 | 250 | 46878 | 4 |
| P01023 | Alpha-2-macroglobulin OS=Homo sapiens OX=9606 GN=A2M PE=1 SV=3 | 221 | 164613 | 10 |
| P00738 | Haptoglobin OS=Homo sapiens OX=9606 GN=HP PE=1 SV=1 | 219 | 45861 | 5 |

**Table S3.6** PEG-MWCNTs-mDAAO Hard Corona

| **Accession Number** | **Protein Name** | **Mascot Score** | **Mr** | **N° peptides** |
| --- | --- | --- | --- | --- |
| P02768 | Serum albumin OS=Homo sapiens OX=9606 GN=ALB PE=1 SV=2 | 5817 | 71317 | 31 |
| P0DOX5 | Immunoglobulin gamma-1 heavy chain OS=Homo sapiens OX=9606 PE=1 SV=2 | 1517 | 49925 | 7 |
| P01024 | Complement C3 OS=Homo sapiens OX=9606 GN=C3 PE=1 SV=2 | 1013 | 188569 | 25 |
| P02787 | Serotransferrin OS=Homo sapiens OX=9606 GN=TF PE=1 SV=3 | 856 | 79294 | 11 |
| P01009 | Alpha-1-antitrypsin OS=Homo sapiens OX=9606 GN=SERPINA1 PE=1 SV=3 | 776 | 46878 | 6 |
| P04114 | Apolipoprotein B-100 OS=Homo sapiens OX=9606 GN=APOB PE=1 SV=2 | 751 | 516651 | 26 |
| P01859 | Immunoglobulin heavy constant gamma 2 OS=Homo sapiens OX=9606 GN=IGHG2 PE=1 SV=2 | 659 | 36505 | 6 |
| P01023 | Alpha-2-macroglobulin OS=Homo sapiens OX=9606 GN=A2M PE=1 SV=3 | 583 | 164613 | 14 |
| P01834 | Immunoglobulin kappa constant OS=Homo sapiens OX=9606 GN=IGKC PE=1 SV=2 | 539 | 11929 | 3 |
| P01861 | Immunoglobulin heavy constant gamma 4 OS=Homo sapiens OX=9606 GN=IGHG4 PE=1 SV=1 | 515 | 36431 | 6 |
| P01871 | Immunoglobulin heavy constant mu OS=Homo sapiens OX=9606 GN=IGHM PE=1 SV=4 | 460 | 50093 | 8 |
| P01876 | Immunoglobulin heavy constant alpha 1 OS=Homo sapiens OX=9606 GN=IGHA1 PE=1 SV=2 | 334 | 38486 | 7 |
| P00738 | Haptoglobin OS=Homo sapiens OX=9606 GN=HP PE=1 SV=1 | 186 | 45861 | 8 |
| P0DOY2 | Immunoglobulin lambda constant 2 OS=Homo sapiens OX=9606 GN=IGLC2 PE=1 SV=1 | 156 | 11458 | 2 |
| P01011 | Alpha-1-antichymotrypsin OS=Homo sapiens OX=9606 GN=SERPINA3 PE=1 SV=2 | 151 | 47792 | 2 |
| P02790 | Hemopexin OS=Homo sapiens OX=9606 GN=HPX PE=1 SV=2 | 70 | 52385 | 2 |
